# Identifying usual food choices with avocados in a clinical trial cohort of overweight and obese adults in Australia

**DOI:** 10.1371/journal.pone.0279567

**Published:** 2023-01-26

**Authors:** Vivienne X. Guan, Elizabeth P. Neale, Yasmine C. Probst

**Affiliations:** 1 School of Medical, Indigenous and Health Sciences, Faculty of Science, Medicine and Health, University of Wollongong, Wollongong, New South Wales, Australia; 2 Illawarra Health and Medical Research Institute, Wollongong, New South Wales, Australia; Washington State University, UNITED STATES

## Abstract

**Background:**

Consumption of avocados has been suggested to be beneficial for weight control, however, limited research is available about the related food choices. Understanding the food choices associated with avocados at meal occasions may further aid behavioural strategies to lose weight. The present study used a systematic approach to develop an avocado-specific food database, with the aim to explore food choices related to avocados at meal occasions as reported by overweight and obese volunteers in weight loss clinical trials.

**Methods:**

The avocado-specific database was based on AUSNUT 2011–13 food composition database structure and was developed via a systematic approach, which determined the avocado content of Australian foods and beverages. Baseline usual food intake data was retrospectively pooled from four food-based clinical trials (n = 758). The Apriori algorithm of association rules, a two-step descriptive method was used to identify food choices associated with avocados at different meal occasions using a nested hierarchical food group classification system.

**Results:**

The avocado database identified 34 avocados and avocado-containing foods and beverages. The proportion of avocado consumers in the pooled cohort was 51.3% (n = 389), with an average avocado intake of 21.57 ± 36.75 grams per day. Avocados were found to be closely related to other food groups at all of the food group levels at main meal occasions. A total of 68 food items containing avocados were identified for avocado consumers at the breakfast, lunch and dinner meals.

**Conclusion:**

The avocado specific database provides a snapshot of the foods and beverages which contain avocados. Enumerating the full range of food choices in relation to avocado consumption should provide examples of food choices that people might consider in their efforts to increase their avocado consumption.

## Introduction

The prevalence of overweight and obesity continue to increase as a global epidemic, tripling since 1975 [1, 2]. The current prevalence of overweight and obesity among adults is 67% in Australia [3] and 52% worldwide [2]. Overweight and obesity is a risk factor for many chronic diseases and is associated with increased mortality [4]. For example, in Australia, 46% of persons with hypertensive heart disease, 25% with coronary heart disease, and 22% of the stroke burden were attributed to overweight and obesity alone [5].

Consuming an energy-restricted pattern of eating is an effective strategy to lose weight [6]. However, adherence to energy-restrictions appears to be poor in individuals who are overweight and obese [7]. Overweight and obese individuals who already adhere well to dietary recommendations prior to a weight loss intervention may experience difficulties with adhering to energy restrictions compared to those who do not [8]. Furthermore, the evidence suggests that approximately half of participants who participated in a weight loss trial regained their weight after the intervention period [9]. Although the restriction of energy intake tends to be the most important factor to lose weight, evidence is now emerging to suggest that the quality and quantity of macronutrients may play a critical role in weight loss via regulation of satiety in the short-term [10]. It may indicate that food choices in relation to achieving dietary goals for weight loss may be a critical factor in adherence to an energy-restricted diet for weight loss.

Avocados are a rich source of key nutrients including monounsaturated fatty acids, fibre, potassium, magnesium, and phytochemicals [11, 12]. Epidemiological evidence suggests that avocado consumers tend to have significantly lower body weight and body mass index (BMI) than non-consumers [13–15]. Isocaloric substitution of foods with avocados in a lunch meal occasion resulted in an increased post-ingestive satiety during a 5-h period in overweight and obese adults [16]. Moreover, research has suggested that the higher monounsaturated fat and dietary fiber content of avocados may contribute to weight loss by changing the composition of the gut microbiota, thereby stimulating satiety-signalling [17]. Thus, consuming avocados may assist with weight management in this population.

Understanding food choice decisions at meals is important prior to designing a dietary intervention for weight loss. Food choice events are colloquially labelled as meal occasions (e.g. breakfast, lunch, dinner, or snacks) [18]. During meal occasions, individual foods and/or multi-ingredient foods, such as mixed dishes are eaten [19, 20]. Thus, meal patterns appear to be closely linked to eating habits [21] implying that the translation of dietary recommendations to practice requires identification of the actual foods that make up the meals [22]. Understanding how avocados are consumed at meal occasions can be a crucial element toward developing dietary strategies to include avocados in a pattern of eating for weight loss though little research has been conducted in this area. In addition, avocados may be consumed as single-ingredient foods as raw avocados, or as ingredients within multi-ingredient foods, such as sushi and guacamole [23, 24]. To accurately estimate avocado intake, the amount of avocado contained within these multi-ingredient foods needs to be known. The present study used a systematic approach to develop an avocado-specific database for this purpose. Created as an expansion to the Australian food composition database AUSNUT 2011–13, this research aimed to explore the food choices in relation to avocados at meal occasions as reported by a sample of overweight and obese volunteers in weight loss trials.

## Materials and methods

### Development of the avocado database

The Australian food composition database AUSNUT 2011–13 was expanded to include data on avocados and avocado-containing foods. AUSNUT 2011–13 contains data for 5,740 foods and beverages which were derived from results of the 2011–12 National Nutrition and Physical Activity Survey (NNPAS), as part of the 2011–13 Australian Health Survey (AHS). The methods for the development of the avocado database were based on previous methods used to develop a similar database for nuts [25].

AUSNUT 2011–13 contains two foods related to whole avocados (‘Avocado, raw’, and ‘Avocado, cooked, with or without fat’) as well as several mixed dishes containing avocados (for example: ‘Sushi, chicken & avocado, with seaweed’), however, the variety of avocado is not specified in the database. Hass avocados are reported to be the most common avocado variety consumed across Australia (e.g., 80% of 2019/20 production, 70,016 tones) [26]. Further, to create the nutrient data for this food item the majority of nutrient data were derived from a composite of eight samples of Hass avocados purchased in five Australian states [23]. Therefore, it can be assumed that the Hass avocado is closely related to the AUSNUT 2011–13 entry for the whole avocado [27]. Although avocado oil is sold in Australia, it is not specified in the AUSNUT 2011–13 database. Therefore, the present avocado database represents avocados as whole food item only.

To identify avocados and avocado-containing products two databases were used, the AUSNUT 2011–13 Food Details File (which contains non-nutrient information such as food derivation, food description and food sampling for all the foods listed in the AUSNUT 2011–13 database) [23] and the AUSNUT 2011–13 Food Recipe File (which provides the ingredient lists and weights of individual ingredients for many of the foods found in the AUSNUT 2011–13 database) [24].

Food name, derivation, description, and the inclusion and exclusion criteria of each food in the AUSNUT 2011–13 Food Details File were initially reviewed by one investigator (VG) to determine if it contained avocados. The ingredient list of the AUSNUT 2011–13 Food Recipe File was then reviewed to identify avocado-containing foods. The identified avocado and avocado-containing food items were assessed via an iterative process with the investigator (VG) and a senior investigator (YP) ascertaining omitted food items containing avocados in the AUSNUT 2011–13 database.

Foods and products with no avocado present in the food name, derivation, description, or the inclusion and exclusion criteria of each food in the AUSNUT 2011–13 Food Details File and the ingredients lists outlined in the AUSNUT 2011–13 Food Recipe File were regarded as not containing avocados (0 g avocado per 100 g food). The proportion of avocado of avocado-containing foods were determined by using the AUSNUT 2011–13 Food Recipe File [24]. The avocado content of avocado-containing products was calculated using the equation. For example, the avocado content of the food ‘Dip, avocado or guacamole, homemade’ was calculated as 81.14g/100g by dividing the weight of ‘Avocado, raw’ (300g) by the weight of the final product (369.74g), then multiplying by 100 to be presented as gram avocados per 100 g food.


$$Avocado\;content\;(g/100g)=\frac{Weight\;of\;avocado\;(x\;grams)}{Weight\;of\;avocado-containing\;products\;(y\;grams)}\times 100$$

For those foods that were identified from the AUSNUT 2011–13 Food Detail File, but did not report ingredients listed in the AUSNUT 2011–13 Food Recipe File, a similar food or product was used to estimate the avocado content based on professional judgement. For example, in the case of the food ‘Sushi, California roll, commercial’, the avocado content of the food was estimated based on a similar food item ‘Sushi, tuna & avocado, with seaweed’.

Following the development of the database, the number of avocado and avocado-containing products contained in the database were identified. The presence of avocado and avocado-containing products according to the AUSNUT 2011–13 food group classification system was then explored. The mean avocado content for avocado and/or avocado-containing products was calculated at each food group level (major, sub-major and minor), using SPSS (IBM Corp, 2019, version 25).

### Study participants

Usual food choice combinations with avocados in four previously published food-based clinical trial studies were explored [28–31]. All studies were registered with the Australian Clinical Trials Registry (ACTRN12608000453381 https://www.anzctr.org.au/Trial/Registration/TrialReview.aspx?id=83115, ACTRN12608000425392 https://www.anzctr.org.au/Trial/Registration/TrialReview.aspx?id=82844, ACTRN12610000784011 https://www.anzctr.org.au/Trial/Registration/TrialReview.aspx?id=335943 and ANZCTRN 12614000581662 https://www.anzctr.org.au/Trial/Registration/TrialReview.aspx?id=366400). Ethics approval was obtained for the initial studies and for this investigation by the University of Wollongong/Illawarra Shoalhaven Local Health District Human Research Ethics Committee (HE04/326, HE07/323, HE10/192 and HE13/189). The data from the four studies was linked and retrospectively pooled for analysis as a baseline cohort [32]. The detailed process and characteristics of study participants has been described elsewhere [32]. In brief, the original studies were food-based randomised controlled trials for weight loss, conducted between 2005 and 2015 at the University of Wollongong, Australia [28–31]. All study participants were recruited from the Illawarra, a major coastal region 70 km south of Sydney, Australia.

### Dietary intake data and food intake data preparation

A validated dietitian-administered diet history interview [33] was used to derive self-reported food intake data during the four trials. Participants were asked to recall their intakes during the interview, reflecting on usual food consumption at participant defined meal occasions. Commonly omitted food items were assessed by using a food checklist during the interview. The nested hierarchical food group classification system from the AUSNUT 2011–13 food composition database was used for the analyses of this study [34]. Food intake data was grouped into participant-defined meals, which were labelled as breakfast, lunch and dinner. Other meals such as morning tea, afternoon tea, desserts, extras and snacks, were grouped into an ‘other’ meal occasion. To prevent undue duplication, repeated food items at each food group level within the meals were removed from the dataset to ensure that each food group was only included once for each meal occasion at each food level.

In order to determine the avocado consumption, the developed avocado-specific food composition database was applied to the dietary intake data. In the AUSNUT 2011–13, a semi-structured naming system is applied to differentiate between the food items and describe the nature of food [35]. For example, ‘Hamburger, bread roll, beef patty, with avocado & cheese, takeaway & homemade’ and ‘Hamburger, bread roll, beef patty, with avocado, cheese & salad, takeaway & homemade’. To aid in the analyses of food combinations with avocados, the same type of avocado-containing foods with different ingredients were aggregated to one food item (such as hamburger, Mexican nachos, sushi and salad). For example, ‘Hamburger, bread roll, beef patty, with avocado & cheese, takeaway & homemade’ and ‘Hamburger, bread roll, beef patty, with avocado, cheese & salad, takeaway & homemade’ were aggregated together as ‘Hamburger’. Non avocado-containing foods were kept as originally assigned in the food groups.

### Statistical analysis

The Apriori algorithm of association rules was used to identify food groups associated with avocado consumption, using RStudio, version 1.0.44 (incorporating R, version 3.2.5; The R Foundation for Statistical Computing, Vienna, Austria) [36]. The algorithm is a two-step descriptive method of creating rules to determine associations between items in a dataset [37, 38]. The association rules are presented to indicate that if the antecedent food items are reported, the consequent food items are also reported. In the present study, the consequent food items were defined as avocados and avocado containing food items. This method has been used to explore food choice combinations at meals in other studies [32, 39–43]. In the first step, a set of frequent food items containing avocados was generated, which is determined by a frequency threshold which is referred to as support and represents the percentage of the records containing the identified frequent food item sets [37, 38]. In the second step, the frequent food items sets are used to generate association rules. Constraints include support, confidence and lift, which are applied to identify the association rules [37, 38, 44]. The support is the percentage of participant food records containing both precursor and consequent items (in the case, avocados or avocado-containing foods). The confidence is calculated as a percentage of participant food records containing both items divided by the percentage of participant food records only containing precursor items. The final constraint referred to as lift (the confidence of a rule divided by the percentage of participant food records only containing avocados or avocado-containing foods [44]) was used to identify the association rules. A lift >1 indicates that antecedent food item(s) and avocado or avocado containing foods are more likely to depend on each other [44].

In the present analyses, the threshold of the possible food group combinations at events (meals) was set as one quarter of the study participants in the cohort [45]. In other words, at least 25% of participants would need to have reported a specific combination of food groups in relation to avocado or avocado-containing foods for the food combination to be reported. The default value for the Apriori algorithm within the R software (0.80) was used for the values of confidence [36]. At each event, many closely related food groups tend to be identified in the dataset, contributed by inherent variability related to food intakes. Thus, redundant closely related food groups were removed to minimize unnecessary complexity by comparing closely related food groups at meal occasions [32, 43, 44, 46].

## Results

### Avocado database

A total of 34 (0.6%) of the 5740 foods and beverages contained in AUSNUT 2011–13 were identified as avocado or avocado-containing foods. Of those foods, the avocado content of 32 items was calculated using a recipe-based approach. The remaining two foods were whole avocados, ‘Avocado, raw’ and ‘Avocado, cooked, with or without fat’. The avocado database is presented in S1 **Table**.

Avocados or avocado-containing foods were found in four of the 24 major AUSNUT 2011–13 food groups (**Table 1)**. Most avocado and avocado-containing foods (64.7% of all avocado-containing foods, n = 22) were found in the ‘Cereal based products and dishes’. Seven (20.6%) avocado-containing food products were found in the ’Vegetable products and dishes’ food group; three (8.8%) were found in the ’Savory sauces and condiments’ good group; and two (5.1%) were found in the ’Fish and seafood products and dishes’ food group. The mean avocado content of avocado-containing foods in the major food groups was 8.7g/100g (standard deviation (SD): 2.81) for ‘Cereal based products and dishes’, 36.85g/100g (SD: 43.66) for ‘Vegetable products and dishes’, 32.0g/100g (SD: 42.79) for ‘Savoury sauces and condiments’ and 10.2g/100g (SD: 7.21) for ‘Fish and seafood products and dishes’.

**Table 1:** An overview of the avocado content (grams avocado per 100 g food) at food group levels* in which avocado-containing products were found.

AUSNUT2011-13 food group	Number of avocado-containing foods in food group (n = 34)	Proportion of avocado-containing foods in food group (%)	Mean avocado content of avocado-containing foods in food group (g/100g)	Highest avocado content in food group (g/100g)
**Cereal based products and dishes**	**22**	**64.70**	**8.77**	**15.70**
Mixed dishes where cereal is the major ingredient	22	64.70	8.77	15.70
*Burgers*, *saturated fat ≤5 g/100 g*	*2*	*5*.*90*	*5*.*66*	*6*.*57*
*Taco and tortilla-based dishes*, *saturated fat ≤5 g/100 g*	*8*	*23*.*50*	*7*.*91*	*9*.*24*
*Taco and tortilla-based dishes*, *saturated fat >5 g/100 g*	*4*	*11*.*80*	*12*.*63*	*15*.*70*
*Sushi*, *all types*	*8*	*23*.*50*	*8*.*49*	*10*.*00*
**Fish and seafood products and dishes**	**2**	**5.90**	**10.21**	**15.31**
Fin fish (excluding commercially sterile)	1	2.90	5.11	5.11
*Fin fish*, *fresh*, *frozen*	*1*	*2*.*90*	*5*.*11*	*5*.*11*
Mixed dishes with fish or seafood as the major component	1	2.90	15.31	15.31
*Mixed seafood dishes with crustacea*, *molluscs or other seafood products as the major component*	*1*	*2*.*90*	*15*.*31*	*15*.*31*
**Savoury sauces and condiments**	**3**	**8.80**	**32.01**	**81.14**
Dips	3	8.80	32.01	81.14
*Vegetable based dips*	*2*	*5*.*90*	*46*.*57*	*81*.*14*
*Other dips*	*1*	*2*.*90*	*2*.*90*	*2*.*90*
**Vegetable products and dishes**	**7**	**20.60**	**36.85**	**100.00**
Other fruiting vegetables	2	5.90	100.00	100.00
*Other fruiting vegetables*	*2*	*5*.*90*	*100*.*00*	*100*.*00*
Dishes where vegetable is the major component	5	14.70	11.59	23.00
*Salads*, *vegetable based*	*5*	*14*.*70*	*11*.*59*	*23*.*00*

* Major food groups shown in bold; sub-major food groups shown in underline; minor food groups shown in italics

### Usual food choice combinations with avocado

Data for a total of 758 participants were analysed (**Table 2**). The proportion of participants who were consuming any avocado was 51.3% (n = 389) with the average avocado intake among avocado consumers 21.57 ± 36.75 grams per day. A total of 596 food items containing avocados were identified for avocado consumers. The proportion of participants who reported ‘avocado, raw’ was 62.1% (n = 370). There were 33.4% (n = 130) of participants who consumed avocados during at least two meal occasions. The average avocado intake at breakfast was 2.07 ± 7.22 grams per day (n = 53, 8.9%), 12.18 ± 17.80 for lunch (n = 295, 49.5%), 6.02 ± 13.62 for dinner (n = 207, 34.7%) and 1.31 ± 8.37 for other meals (n = 41, 6.9%). Details of the food items containing avocado at meal occasions are shown in **Table 3**.

**Table 2:** Participant characteristics of the pooled data set*.

	All (n = 758)	Male (n = 201, 27%)	Female (n = 557, 73%)
**Age (years)**	46 (38, 51)	45 (38, 51.5)	46 (39, 51)
**Height (m)**	1.67 (1.62, 1.73)	1.77 (1.73, 1.83)	1.65 (1.60, 1.69)
**Weight (kg)**	88.40 (79.08, 99.73)	101.10 (93.20, 109.35)	84.50 (76.65, 94.10)
**Body mass idex (kg/m** ^**2**^ **)**	31.39 (28.76, 34.61)	31.78 (29.62, 34.85)	31.12 (28.41, 34.59)
**Energy (MJ)**	9.01 (7.54, 11.03)	11.34 (9.10, 13.6)	8.59 (7.23, 10.09)

* Medians and interquartile ranges. Abbreviations: n: number of participants; kg: kilograms; m: meters and MJ: megajoules

**Table 3:** Food items containing avocados at meal occasions.

Avocado items at meal occasions	n (%)
**Breakfast**	**53 (8.9)**
Avocado, raw	53 (8.89)
**Lunch**	**295 (49.5)**
Avocado, raw	194 (32.55)
Dip	3 (0.5)
Prawn cocktail	1 (0.17)
Salad	44 (7.38)
Sushi	53 (8.89)
**Dinner**	**207 (34.7)**
Avocado, raw	110 (18.46)
Dip	5 (0.84)
Mexican nachos	11 (1.85)
Mexican wrap	1 (0.17)
Salad	69 (11.58)
Sushi	11 (1.85)
**Others meals**	**41 (6.9)**
Avocado, raw	13 (2.18)
Dip	21 (3.52)
Salad	4 (0.67)
Sushi	3 (0.5)

* Total of food items containing avocados at meal occasions were bolded. Abbreviations: n: number of participants

**Fig 1:**
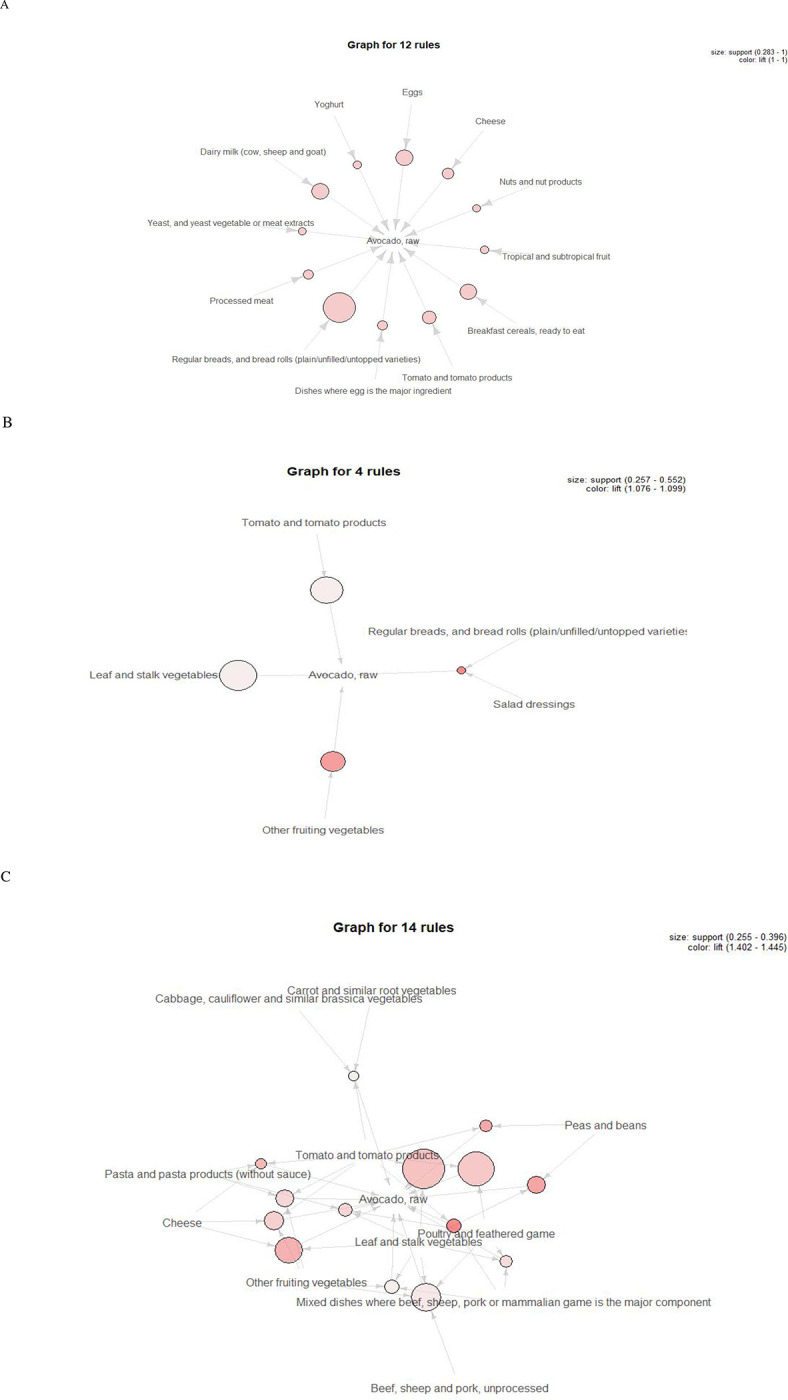
Visualization of the food choice associations with avocados at the sub-major food group level A. Breakfast (n = 12). B. Lunch (n = 4). C. Dinner (n = 14). Arrows represents closely related food groups relationships. The size of the sphere shows the value of support. The intensity of the colour indicates the value of lift.

‘Avocado, raw’ was the only avocado or avocado-containing food item that was found to be closely related to other food groups at all of the food group levels during main meal occasions. A total of 68 food items containing avocados were identified for avocado consumers. A total of 30 clusters of food choices were found in relation to avocados at breakfast (n = 12), lunch (n = 4), and dinner (n = 14) meal occasions at the sub-major food group level (**Fig 1**).

At the breakfast meal occasion, the top three food choices related to ‘Avocado, raw’ were ‘Cereals and cereal products’ (100%), ‘Milk products and dishes’ (81%) and ‘Egg products and dishes’ (77%) at the major food group level. At the sub-major food group level, the top three food choices related to ‘Avocado, raw’ were ‘Regular breads, and bread rolls (plain/unfilled/untopped varieties)’ (100%), ‘Dairy milk (cow, sheep and goat)’ (57%) and ‘Eggs’ (57%). At the minor food groups level, the top three food choices related to ‘Avocado, raw’ were ‘Eggs, chicken’ (57%), ‘Breads, and bread rolls, mixed grain, mandatorily fortified’ (45%) and ‘Tomato’ (45%).

At the lunch meal occasion, vegetables were closely related to ‘Avocado, raw’, such as ‘Leaf and stalk vegetables’ (n = 144), ‘Tomato and tomato products’ (n = 132), and ‘Other fruiting vegetables’ (n = 110). At the sub-major food group level, when the food combination of ‘Regular breads, and bread rolls (plain/unfilled/untopped varieties)’ and ‘Salad dressings’ was reported, 82% of the time ‘Avocado, raw’ was also reported (n = 67).

At the dinner meal occasion, no food choice association was identified at the major group level. At the sub-major food group, a total of 14 food choice combinations with avocados were identified. For example, among 40% of the avocado consumers (n = 76), if the food choice combination of ‘Leaf and stalk vegetables’ and ‘Tomato and tomato products’ was reported, ‘Avocado, raw’ was also reported. Moreover, 83% of the time when the food combination of ‘Mixed dishes where beef, sheep, pork or mammalian game is the major component’ and ‘Tomato and tomato products’ was reported, ‘Avocado, raw’ was also reported (n = 53).

## Discussion

To our knowledge, this study is the first to develop an avocado specific food database to estimate the usual avocado intakes from overweight and obese adult participants. By applying the avocado specific food composition database, the present analysis allowed us to explore food choices for avocados that were more closely related to food consumed at meal occasions by overweight and obese adults. This provides a positioning for avocados in relation to other food groups at different meal occasions, which can be used to support food guidance and dietary behaviour change strategies.

The present study highlighted the importance of developing an avocado specific database to accurately estimate habitual avocado intake. The proportion of avocado consumers (defined as consuming the whole avocado only) in the US population (≥ 19 years of age) was suggested to be approximately 2% (n = 347), which was identified from one 24-hour dietary recall based on several cycles of NHANES (2001–2008) [14]. The average avocado intake of these avocado consumers was 70.1 (SD: 5.4) g/day [14]. Moreover, based on dietary intake data from a food frequency questionnaire (FFQ) in the Adventist Health Study (n = 21,165), 58% of study participants were avocado consumers. In this study, avocado consumption meant consuming avocado and/or guacamole [13]. The median avocado consumption among the avocado consumers was 2.3 (range: 1.1, 120.1) g/day [13]. The discrepancy between the present analysis and these findings may be due to the dietary assessment methods and the food composition data used to estimate avocado intake. Dietary assessment methods such as diet history interview and FFQs are used to estimate usual intake or habitual intake while dietary intake data from a single 24-h dietary recall is not reflective of habitual avocado intake. Avocados may be consumed episodically and may, therefore, be reported as zero on a particular day. In addition, in the present analysis, avocados were also found in multi-ingredient foods, such as sushi, hamburger and Mexican style dishes. The avocado intake appears to be underestimated by assessing the whole avocado alone or using single-day dietary intake data. Thus, to facilitate accurate estimation of avocado intake, a systematic approach using habitual dietary intake data was required to include specific information for avocado and avocado-containing foods, including multi-ingredient foods.

Consuming avocados is suggested to be beneficial for weight control and weight loss [13, 14, 16, 17, 47]. Understanding food choices in relation to avocados is a pivotal step to designing dietary prescriptions and strategies that include avocados for weight loss. Research has suggested that in the short-term, the most critical factor in the success of weight loss tends to be the restriction of energy intake [6, 10]. However, adults who lost excess body weight through energy restriction typically experience a complex and slow process of behaviour change that is influenced by many interacting factors [48]. For example, in terms of food choices that comply with an energy-restricted diet, food literacy and skills are required to incorporate dietary recommendations into everyday food choices [49–51]. Less research is available about how such food choices can be operationalised and implemented in daily practice.

Our findings may assist in developing dietary prescriptions or strategies for better food choices with avocados to assist in behaviour change at the individual level for weight loss. The present results suggest that foods accompanying avocados were chosen differently at main meals in overweight and obese participants. These findings are aligned with a previous study which has demonstrated that overweight and obese study participants tend to choose foods differently at different meal occasions [43]. For example, walnuts are more likely to be consumed as snacks [32]. Meal can function as an organisational unit for food items including avocados for the broader aspects of achieving dietary goals for weight loss. Meals provide insights into how and in what combinations foods are consumed and whether dietary change is needed to comply with dietary prescriptions. This change may include realistic food group substitutions. Moreover, such a function tends to take into account the impact of convenience and flexibility of food preparation. The identified avocado-containing foods and food choice combinations with avocados in the present study suggest that avocados are consumed through foods that are more ‘open’ to variation or those that are ‘closed’ [52]. Open structures allow for adding or substituting of foods with the whole avocado freely [52]. For example, whole avocados tended to be eaten at the breakfast meal occasion. If eggs or tomato were reported, then avocados were also reported. Knowing such a relationship may assist in the planning of how to increase the frequency of avocado consumption. Conversely, closed structures represent that avocados are one of the ingredients in multi-ingredient foods, such as sushi and Mexican style foods [52]. These tend to offer less flexibility to include avocados beyond these multi-ingredient foods. Thus, the identified food choices with avocados provide practical examples to aid in articulating specific and easy-to-implement dietary advice including avocado food selections at each meal occasion for weight loss.

There are several strengths and limitations to the present study. The present study was the first to identify the food choices associated with avocados by using a data mining method and a nested hierarchical food grouping system. The dietary intake data used in this study was derived from a diet history interview. The diet history interview uses an open-ended interview approach and probes to encourage participants to describe their usual food consumption from the first meal of the day to the end of the day [53]. Although such information did focus on food choices at self-defined meals, the richness of food intake data allowed us to capture food choices associated with avocados at meal occasions [54], which may not be captured when using other dietary assessment methods, such as FFQs. There are some limitations to be acknowledged. For the purpose of the pooled data, it was assumed that all dietary data was collected in the same manner. While the dietitians involved in each of the clinical trials were all trained, it cannot be guaranteed that the procedures followed across an 11 year period remained identical. The matching of the food items in the pooled analysis was conducted by one researcher. While this was guided by a pre-constructed matching file, there may have been variations to the data over time which required subjective judgments to be made. The threshold of 25% of participants having each combination means that less common food associations may not have been captured. This specification was set for this specific analysis and should be determined based upon the data characteristics and planned outcomes of the study. The recipes described in the AUSNUT 2011–13 may differ from the recipes used by the individuals when preparing foods in the home (thereby altering avocado content of some multi-ingredient foods). Although the AUSNUT 2011–13 is the most recent survey specific Australian food composition database, at the time of the current study, the information within the AUSNUT 2011–13 database is becoming dated. The Australian food market is constantly changing. Newly developed avocado derived foods, such as avocado oil are not included in the database. Nevertheless, studies that have explored how avocados are consumed in the diet are considerably less frequent. The pooled data analysis of clinical trials offers new opportunities to complement the current studies for weight loss by providing translational evidence toward practice.

## Conclusions

In conclusion, the developed avocado specific database provides a snapshot of the foods and beverages which contain avocados. Food choice analyses offer an example of food choices in relation to avocados made by overweight and obese volunteers. We applied a descriptive data mining tool (the Apriori algorithm of association rules) and nested hierarchical food grouping system to examine the food choices that were associated with avocados at different meal occasions; providing data at a much deeper level. Enumerating the full range of food choices in relation to avocado consumption should provide examples of food choices that people might consider in their efforts to increase their avocado consumption.

## Supporting information

S1 Checklist STROBE-nut: An extension of the STROBE statement for nutritional epidemiology. (DOCX)

S1 Table Database for the estimation of the avocado content of Australian foods (AUSNUT 2011–13 expansion). (PDF)
