# Supplementary material for: Identifying usual food choices with avocados in a clinical trial cohort of overweight and obese adults in Australia
Source: PLoS One. 2023 Jan 26;18(1):e0279567. doi: 10.1371/journal.pone.0279567 (PMC9879481; doi:10.1371/journal.pone.0279567)
Supplement: S1 Table — (PDF) [file pone.0279567.s002.pdf]

Supplementary Table: Database for the estimation of the avocado content of Australian foods (AUSNUT 2011-13 expansion)

Food ID	Survey ID	Food Name	content %
13B20299	23504003	Dip, not further defined	0.03
02F40413	13514023	Sushi, not further defined	0.03
13A12630	24904032	Salad, not further defined	0.01
02F30284	13505033	Hamburger, bread roll, beef patty, with avocado, cheese & salad, takeaway & homemade	0.05
05D10384	13514022	Sushi, vegetable, with seaweed	0.05
05A10803	15101111	Tuna, tartare (tartar)	0.05
02F30266	13505032	Hamburger, bread roll, beef patty, with avocado & cheese, takeaway & homemade	0.07
13A12597	24904009	Salad, garden, no added dressing	0.07
02F40426	13507022	Mexican wrap, beef, with cheese, guacamole, salad & salsa	0.07
02F40427	13507030	Mexican wrap, chicken, with cheese, guacamole, salad & salsa	0.07
02F40465	13507035	Mexican wrap, fish, with cheese, guacamole, salad & salsa	0.07
02F40425	13507025	Mexican wrap, beef & bean, with cheese, guacamole, salad & salsa	0.07
02F40461	13507014	Mexican wrap, bean, with cheese, guacamole, salad & salsa	0.07
02F40462	13507036	Mexican wrap, with cheese, guacamole, salad & salsa	0.09
02F40423	13507021	Mexican wrap, beef, with cheese, guacamole & salsa	0.09
02F40424	13507029	Mexican wrap, chicken, with cheese, guacamole & salsa	0.09
02F40223	13514003	Sushi, California roll, commercial	0.10
05D10383	13514005	Sushi, chicken & avocado, with seaweed	0.10
05D10388	13514006	Sushi, chicken & avocado, without seaweed	0.10
05D10377	13514015	Sushi, salmon & avocado, with seaweed	0.10
05D10380	13514020	Sushi, tuna & avocado, with seaweed	0.10
05D10400	13514021	Sushi, tuna & avocado, without seaweed	0.10
02F40329	13508004	Mexican nachos, corn chips, beef, with cheese, guacamole & salsa	0.11
02F40331	13508006	Mexican nachos, corn chips, beef & bean, with cheese, guacamole & salsa	0.11
13A12588	24904008	Salad, fennel, no added dressing	0.12
13B10276	23502001	Dip, avocado or guacamole, commercial	0.12
02F40333	13508002	Mexican nachos, corn chips, beans, with cheese, guacamole & salsa	0.13
13A12144	24904010	Salad, garden, added avocado, no added dressing	0.15
05C10223	15603005	Prawn cocktail	0.15
02F40463	13508007	Mexican nachos, corn chips, with cheese, guacamole, salad & salsa	0.16
13A12591	24904034	Mixed salad vegetables, additional vegetables, for use in garden salads	0.23

13B10262	23502002	Dip, avocado or guacamole, homemade	0.81
13A11674	24705001	Avocado, raw	1.00
13A12424	24705002	Avocado, cooked, with or without fat	1.00
10F40019	31103001	Beef, extract, bonox	0.00
13A12001	31302001	Basil, dried	0.00
10E10113	31302002	Cardamom, seeds, ground	0.00
10E10098	31302003	Chilli (chili) powder	0.00
10E10107	31302004	Chilli (chili), dried, ground	0.00
10E10099	31302005	Cinnamon, dried, ground	0.00
10E10106	31302006	Cloves, dried, ground	0.00
10E10100	31302007	Coriander seed, dried, ground	0.00
10E10093	31302008	Cumin (cummin) seed, dried, ground	0.00
10E10096	31302009	Curry powder	0.00
10E10112	31302010	Fenugreek seed	0.00
10E10101	31302011	Ginger, dried, ground	0.00
10E10115	31302012	Herbs, mixed, dried	0.00
10E10095	31302013	Mustard powder	0.00
10E10102	31302014	Nutmeg, dried, ground	0.00
10E10105	31302015	Oregano or marjoram, dried	0.00
10E10110	31302016	Paprika, dry powder	0.00
10E10097	31302017	Pepper, ground, black or white	0.00
10E10116	31302018	Rosemary, dried	0.00
10E10111	31302019	Sage, dried	0.00
10F60105	31301001	Salt substitute, potassium chloride	0.00
10F60107	31301002	Salt, cooking	0.00
10F60104	31301003	Salt, flavoured	0.00
10F60102	31301004	Salt, rock	0.00
10F60103	31301005	Salt, sea	0.00
10F60101	31301006	Salt, table, iodised	0.00
10F60097	31301007	Salt, table, non-iodised	0.00
10F60112	31301008	Salt, not further defined	0.00
10F60108	31303001	Seasoning mix, chilli-based, for tacos	0.00
10E10114	31302020	Spice, mixed or all spice	0.00
10F60111	31303002	Stock, dry powder or cube	0.00
10F60113	31304001	Stock, liquid, all flavours (except fish), prepared from commercial powder or cube	0.00
10C10559	31304002	Stock, liquid, all flavours (except fish), homemade from basic ingredients	0.00
10C10501	31304003	Stock, liquid, fish, commercial	0.00
10C10500	31304004	Stock, liquid, fish, homemade from basic ingredients	0.00
10F60114	31303003	Stock, dry powder or cube, reduced salt	0.00
10F60115	31304005	Stock, liquid, all flavours, reduced salt, prepared from commercial powder or cube	0.00
10E10103	31302021	Thyme, dried, ground	0.00
10E10104	31302022	Turmeric, dried, ground	0.00
14B10174	31501001	Baking powder, dry powder	0.00
14B10166	31501002	Baking soda (bicarbonate), dry powder	0.00

14B10167	31501003	Cream of tartar, dry powder	0.00
10F60109	31502001	Gelatine, all types	0.00
13B20222	31103002	Lecithin, soy, granules	0.00
10F60110	31401001	Vanilla, artificial	0.00
10F30010	31101001	Yeast, bakers, compressed	0.00
10F30011	31101002	Yeast, dry powder	0.00
14B10182	31503012	Caffeine	0.00
14B10172	31503001	Calcium	0.00
14B10173	31503002	Fibre	0.00
14B10168	31503003	Folic acid	0.00
14B10179	31503004	Iodine	0.00
14B10181	31503005	Maltodextrin	0.00
14B10176	31503006	Oligosaccharide	0.00
14B10180	31503007	Thiamin	0.00
14B10169	31503008	Beta-carotene	0.00
14B10170	31503009	Vitamin C	0.00
14B10171	31503010	Vitamin E	0.00
01A10079	29101003	Beer, lager or ale style (alcohol 5% v/v & above)	0.00
01A10082	29101004	Beer, lager or ale style (alcohol 4.6% v/v)	0.00
01A10094	29102002	Beer, lager or ale style, mid strength (alcohol 3.5% v/v)	0.00
01A10085	29102003	Beer, lager or ale style, reduced alcohol or light beer (alcohol 2.5% v/v)	0.00
01A10097	29101001	Beer, flavoured (alcohol 4% v/v)	0.00
01A10081	29101002	Beer, home-brewed, full strength	0.00
01A10086	29102001	Beer, home-brewed, reduced alcohol, mid-strength, or light beer	0.00
01A10084	29101005	Beer, stout (alcohol 6% v/v)	0.00
01A10092	29101006	Beer, not further defined	0.00
01A30161	29505001	Bitters	0.00
01A30139	29301001	Brandy	0.00
01A30170	29301002	Brandy, cooked	0.00
01A30166	29501001	Cointreau	0.00
01A30148	29401001	Cider, apple, alcoholic, draught style	0.00
01A30149	29401002	Cider, apple, alcoholic, dry style	0.00
01A30150	29401003	Cider, apple, alcoholic, sweet style	0.00
01A30163	29401004	Cider, apple, alcoholic, not further defined	0.00
01A30182	29402001	Cider, pear (perry), alcoholic	0.00
01A30154	29505003	Ethanol 100%	0.00
01A30141	29301003	Gin	0.00
01A30145	29501002	Liqueur, advocaat	0.00
01A30146	29501003	Liqueur, cream-based, coffee flavour	0.00
01A30147	29501004	Liqueur, cream-based, non-coffee flavours	0.00
01A30186	29501005	Liqueur, 25% v/v alcohol, not cream based	0.00
01A20126	29505002	Mirin	0.00
01A30142	29301004	Ouzo	0.00
01A20118	29203001	Port	0.00
01A30143	29301005	Rum, dark & light coloured	0.00

01A20116	29203002	Sherry, dry style (~ 1% sugars)	0.00
01A20117	29203003	Sherry, sweet style (~11% sugars)	0.00
01A20127	29203004	Sherry, not further defined	0.00
01A30175	29301006	Tequila	0.00
01A30144	29301007	Vodka	0.00
01A30140	29301008	Whisky or scotch	0.00
01A30191	29301009	Spirit, not further defined	0.00
01A30183	29502001	Cocktail, cream or milk based	0.00
01A30189	29502002	Cocktail, fruit juice based	0.00
01A30190	29502003	Cocktail, soft drink based	0.00
01A30155	29504001	Mixed drink, gin & tonic, commercial, pre-mixed	0.00
01A30152	29503001	Mixed drink, rum & cola, pre-mixed	0.00
01A30188	29503002	Mixed drink, spirit & energy drink	0.00
01A30192	29502004	Mixed drink, spirit & non-cola flavoured soft drink, home-mixed	0.00
01A30167	29502005	Mixed drink, spirit & soda, home-mixed	0.00
01A30165	29502006	Mixed drink, spirit & tonic, home-mixed	0.00
01A30177	29502007	Mixed drink, vodka & fruit juice, home-mixed	0.00
01A30158	29504002	Mixed drink, vodka & non-cola flavoured soft drink, commercial, pre-mixed	0.00
01A30153	29502008	Mixed drink, whisky or scotch & ice	0.00
01A30159	29503003	Mixed drink, whisky or scotch & regular cola, commercial, pre-mixed	0.00
01A30164	29502009	Mixed drink, whisky or scotch & regular cola, home-mixed	0.00
01A30187	29502010	Mixed drink, whisky or scotch & diet soft drink	0.00
01A30160	29504003	Mixed drink, whisky or scotch & dry, commercial, pre-mixed	0.00
01A20129	29502011	Mixed drink, wine & regular cola soft drink, home-mixed	0.00
01A20122	29204001	Wine cooler, wine & fruit juice blend, all flavours	0.00
01A20107	29201001	Wine, red	0.00
01A20120	29204002	Wine, red, reduced alcohol	0.00
01A20109	29201002	Wine, red, sparkling	0.00
01A20110	29201003	Wine, red, cooked	0.00
01A20108	29201004	Wine, rose	0.00
01A20111	29202001	Wine, white, dry style (sugars content < 1%)	0.00
01A20112	29202002	Wine, white, medium dry style (~ 1% sugars)	0.00
01A20113	29202003	Wine, white, medium sweet style (~ 2.5% sugars)	0.00
01A20119	29204003	Wine, white, reduced alcohol	0.00
01A20115	29202005	Wine, white, cooked	0.00
01A20125	29202004	Wine, white, not further defined	0.00
01A20114	29202006	Wine, white, sparkling	0.00
01A20121	29205002	Wine, white, sparkling, de-alcoholised	0.00
01A20123	29202007	Wine, white, sweet dessert style	0.00
01A20128	29201005	Wine, not further defined	0.00
01B10322	11804001	Cocoa powder	0.00

01B10523	11804002	Beverage base, banana flavour (Nesquik brand)	0.00
01B10319	11804003	Beverage base, chocolate flavour, unfortified (Nesquik brand)	0.00
01B10316	11802001	Beverage base, chocolate flavour, added vitamins A & B3 & Fe	0.00
01B10315	11802002	Beverage base, chocolate flavour, added vitamins A, B1, B2, C, D & folate, Ca & Fe (Milo)	0.00
01B10525	11802003	Beverage base, chocolate flavour, added vitamins A, B1, B2, B3, C, D & folate & Ca (Aktavite)	0.00
01B10318	11804004	Beverage base, drinking chocolate, unfortified	0.00
01B10323	11804005	Beverage base, malted milk powder, unfortified	0.00
01B10317	11802005	Beverage base, malted milk powder, added vitamins A, B1, B2, B3, B6, B12, biotin, C, D E & folate, Ca & Zn	0.00
01B10557	11802004	Beverage base, malt chocolate flavour, added vitamins A, B1, B2, C, D & folate, Ca & Fe (Milo malt)	0.00
01B10522	11804006	Beverage base, strawberry flavour, unfortified (Nesquik brand)	0.00
01B10524	11804007	Beverage base, strawberry flavour, from drinking straw, containing added sugar & intense sweetener, unfortified	0.00
01B10328	11803001	Beverage, chocolate flavour, from cocoa powder, with cows milk	0.00
01B10329	11803002	Beverage, chocolate flavour, from drinking chocolate, with regular fat cows milk	0.00
01B10553	11803003	Beverage, chocolate flavour, from drinking chocolate, with reduced fat cows milk	0.00
01B10554	11803004	Beverage, chocolate flavour, from drinking chocolate, with skim cows milk	0.00
01B10555	11803005	Beverage, chocolate flavour, from drinking chocolate, with soy milk	0.00
01B10564	11803006	Beverage, chocolate flavour, from drinking chocolate, with water	0.00
01B10327	11801001	Beverage, chocolate flavour, from original Milo powder, with cows milk	0.00
01B10563	11801002	Beverage, chocolate flavour, from original Milo powder, with soy milk	0.00
01B10560	11801003	Beverage, chocolate flavour, from Ovaltine powder, with cows milk	0.00
01B10486	11106002	Chai latte, dry powder mix containing milk solids & sugar	0.00
01B10487	11101008	Chai latte, prepared from chai mix powder & water, no added milk	0.00
01B10488	11102002	Chai latte, prepared from chai mix powder & regular fat cows milk	0.00
01B10489	11102003	Chai latte, prepared from chai mix powder & reduced fat cows milk	0.00
01B10490	11102004	Chai latte, prepared from chai mix powder & skim cows milk	0.00
01B10491	11102005	Chai latte, prepared from chai mix powder & soy milk	0.00
01B10303	11205001	Coffee, instant, dry powder or granules	0.00

01B10301	11201002	Coffee, black, from instant coffee powder, without milk	0.00
01B10396	11202001	Coffee, white, from instant coffee powder, made up with regular fat cows milk	0.00
01B10405	11202002	Coffee, white, from instant coffee powder, made up with cows milk not further defined	0.00
01B10306	11205002	Coffee, instant, dry powder or granules, decaffeinated	0.00
01B10305	11203002	Coffee, black, from instant coffee powder, decaffeinated, without milk	0.00
01B10479	11204001	Coffee, white, from instant coffee powder, decaffeinated, made up with cows milk not further defined	0.00
01B10325	11201001	Coffee, espresso style, without milk	0.00
01B10471	11203001	Coffee, espresso style, decaffeinated, without milk	0.00
01B10402	11202039	Coffee, from ground coffee beans, with regular fat coffee whitener	0.00
01B10302	11201006	Coffee, from instant or ground beans, Turkish, no milk, added sugar	0.00
01B10363	11202003	Coffee, cappuccino, from ground coffee beans, with regular fat cows milk	0.00
01B10399	11202004	Coffee, cappuccino, from ground coffee beans, double shot, with regular fat cows milk	0.00
01B10364	11202005	Coffee, cappuccino, from ground coffee beans, with reduced fat cows milk	0.00
01B10470	11202006	Coffee, cappuccino, from ground coffee beans, double shot, with reduced fat cows milk	0.00
01B10365	11202007	Coffee, cappuccino, from ground coffee beans, with skim cows milk	0.00
01B10394	11202008	Coffee, cappuccino, from ground coffee beans, double shot, with skim cows milk	0.00
01B10366	11202009	Coffee, cappuccino, from ground coffee beans, with cows milk not further defined	0.00
01B10367	11202010	Coffee, cappuccino, from ground coffee beans, with regular fat soy milk	0.00
01B10368	11202011	Coffee, cappuccino, from ground coffee beans, with reduced fat soy milk	0.00
01B10468	11202012	Coffee, cappuccino, from ground coffee beans, with rice milk	0.00
01B10339	11202013	Coffee, flat white or latte, from ground coffee beans, with regular fat cows milk	0.00
01B10406	11202014	Coffee, flat white or latte, from ground coffee beans, double shot, with regular fat cows milk	0.00
01B10340	11202015	Coffee, flat white or latte, from ground coffee beans, with reduced fat cows milk	0.00
01B10401	11202016	Coffee, flat white or latte, from ground coffee beans, double shot, with reduced fat cows milk	0.00
01B10342	11202017	Coffee, flat white or latte, from ground coffee beans, with skim cows milk	0.00
01B10412	11202018	Coffee, flat white or latte, from ground coffee beans, double shot, with skim cows milk	0.00

01B10375	11202019	Coffee, flat white or latte, from ground coffee beans, with cows milk not further defined	0.00
01B10343	11202020	Coffee, flat white or latte, from ground coffee beans, with regular fat soy milk	0.00
01B10354	11202021	Coffee, flat white or latte, from ground coffee beans, with reduced fat soy milk	0.00
01B10410	11202022	Coffee, cappuccino, flat white or latte, from ground coffee beans, double shot, with cows milk not further defined	0.00
01B10409	11202023	Coffee, cappuccino, flat white or latte, from ground coffee beans, double shot, with soy milk not further defined	0.00
01B10466	11202024	Coffee, cappuccino, flat white or latte, from ground coffee beans, half shot, with regular fat cows milk	0.00
01B10467	11202025	Coffee, cappuccino, flat white or latte, from ground coffee beans, half shot, with reduced fat cows milk	0.00
01B10421	11202026	Coffee, cappuccino, flat white or latte, from ground coffee beans, half shot, with skim cows milk	0.00
01B10420	11202027	Coffee, cappuccino, flat white or latte, from ground coffee beans, half shot, with soy milk not further defined	0.00
01B10377	11204002	Coffee, cappuccino, flat white or latte, from ground coffee beans, decaffeinated, with regular fat cows milk	0.00
01B10378	11204003	Coffee, cappuccino, flat white or latte, from ground coffee beans, decaffeinated, with reduced fat cows milk	0.00
01B10469	11204004	Coffee, cappuccino, flat white or latte, from ground coffee beans, decaffeinated, with skim cows milk	0.00
01B10474	11204005	Coffee, cappuccino, flat white or latte, from ground coffee beans, decaffeinated, with soy milk not further defined	0.00
01B10344	11202028	Coffee, macchiato, from ground coffee beans, with regular fat cows milk	0.00
01B10345	11202029	Coffee, macchiato, from ground coffee beans, with reduced fat cows milk	0.00
01B10346	11202030	Coffee, macchiato, from ground coffee beans, with skim cows milk	0.00
01B10359	11202031	Coffee, macchiato, from ground coffee beans, with cows milk not further defined	0.00
01B10358	11202032	Coffee, macchiato, from ground coffee beans, with soy milk not further defined	0.00
01B10379	11204006	Coffee, macchiato, from ground coffee beans, decaffeinated, with regular fat cows milk	0.00
01B10380	11204007	Coffee, macchiato, from ground coffee beans, decaffeinated, with reduced fat cows milk	0.00
01B10348	11202033	Coffee, mocha, from ground coffee beans, with regular fat cows milk	0.00
01B10349	11202034	Coffee, mocha, from ground coffee beans, with reduced fat cows milk	0.00

01B10350	11202035	Coffee, mocha, from ground coffee beans, with skim cows milk	0.00
01B10362	11202036	Coffee, mocha, from ground coffee beans, with cows milk not further defined	0.00
01B10478	11202037	Coffee, mocha, from ground coffee beans, double shot, with cows milk not further defined	0.00
01B10361	11202038	Coffee, mocha, from ground coffee beans, with soy milk not further defined	0.00
01B10381	11204008	Coffee, mocha, from ground coffee beans, decaffeinated, with regular fat cows milk	0.00
01B10382	11204009	Coffee, mocha, from ground coffee beans, decaffeinated, with reduced fat cows milk	0.00
01B10300	11201003	Coffee, long black style, from ground coffee beans, without milk	0.00
01B10398	11201004	Coffee, long black style, from ground coffee beans, double shot, without milk	0.00
01B10475	11201005	Coffee, long black style, from ground coffee beans, half shot, without milk	0.00
01B10304	11203003	Coffee, long black style, from ground beans, decaffeinated, without milk	0.00
01B10307	11207001	Coffee substitute, cereal beverage, dry powder or granules	0.00
01B10494	11206001	Coffee, prepared from coffee substitute powder & water, no added milk	0.00
01B10309	11207002	Coffee & chicory essence, concentrate	0.00
01B10308	11209001	Coffee mix, with beverage whitener & sugar, dry powder	0.00
01B10416	11208001	Coffee, prepared from coffee mix with sugar & whitener, no added milk	0.00
01B10422	11209002	Coffee mix, with beverage whitener & intense sweetener, dry powder	0.00
01B10417	11208002	Coffee, prepared from coffee mix with intense sweetener & whitener, no added milk	0.00
01B10483	11209003	Coffee mix, with beverage whitener & sugar, decaffeinated, dry powder	0.00
01B10484	11208003	Coffee, prepared from decaffeinated coffee mix with sugar & whitener, no added milk	0.00
01B10418	11208004	Coffee, prepared from coffee mix, not further defined, no added milk	0.00
01B10310	11209004	Coffee & milk concentrate	0.00
01B10562	11208005	Coffee, prepared from coffee & milk concentrate, no added milk	0.00
01B10324	11209005	Coffee whitener, dry powder	0.00
01B10544	11202040	Coffee, not further defined	0.00
01B20380	11403009	Cordial base, 25% citrus fruit juice (orange & lemon), regular	0.00
01B20382	11403010	Cordial base, 40% citrus fruit juice (orange, lemon or lime), regular	0.00
01B20381	11403011	Cordial base, 25% non-citrus fruit juice (apple & berry), regular	0.00

01B20383	11403012	Cordial base, 40% non-citrus fruit juice (apple & berry), regular	0.00
01B20433	11403001	Cordial base, apple & berry, regular	0.00
01B20379	11403002	Cordial base, blackcurrant juice, regular	0.00
01B20477	11403003	Cordial base, fruit cup or tropical, regular	0.00
01B20505	11403004	Cordial base, ginger, regular	0.00
12A10099	11403005	Cordial base, grenadine syrup	0.00
01B20431	11403006	Cordial base, lemon, regular	0.00
01B20403	11403007	Cordial base, lime or green, regular	0.00
01B20430	11403008	Cordial base, orange or orange & mango, regular	0.00
01B20384	11404007	Cordial base, 25% citrus fruit juice (orange or lemon), intense sweetened or diet	0.00
01B20385	11404001	Cordial base, apple & berry, intense sweetened or diet	0.00
01B20512	11404002	Cordial base, blackcurrant juice, intense sweetened or diet	0.00
01B20479	11404003	Cordial base, fruit cup or tropical, intense sweetened or diet	0.00
01B20424	11404004	Cordial base, lemon, intense sweetened or diet	0.00
01B20432	11404005	Cordial base, lime or green, intense sweetened or diet	0.00
01B20422	11404006	Cordial base, orange or orange & mango, intense sweetened or diet	0.00
01B20411	11401019	Cordial, 25% citrus fruit juice, regular, recommended dilution	0.00
01B20413	11401020	Cordial, 40% citrus fruit juice, regular, recommended dilution	0.00
01B20412	11401021	Cordial, 25% non-citrus fruit juice, regular, recommended dilution	0.00
01B20414	11401022	Cordial, 40% non-citrus fruit juice, regular, recommended dilution	0.00
01B20494	11401001	Cordial, apple & berry, regular, stronger than recommended dilution	0.00
01B20439	11401002	Cordial, apple & berry, regular, recommended dilution	0.00
01B20472	11401003	Cordial, apple & berry, regular, weaker than recommended dilution	0.00
01B20475	11401004	Cordial, blackcurrant juice, regular, stronger than recommended dilution	0.00
01B20410	11401005	Cordial, blackcurrant juice, regular, recommended dilution	0.00
01B20476	11401006	Cordial, blackcurrant juice, regular, weaker than recommended dilution	0.00
01B20493	11401007	Cordial, fruit cup or tropical, regular, stronger than recommended dilution	0.00
01B20458	11401008	Cordial, fruit cup or tropical, regular, recommended dilution	0.00
01B20473	11401009	Cordial, fruit cup or tropical, regular, weaker than recommended dilution	0.00
01B20506	11401010	Cordial, ginger, regular, recommended dilution	0.00
01B20435	11401011	Cordial, lemon, regular, recommended dilution	0.00

01B20474	11401012	Cordial, lemon, regular, weaker than recommended dilution	0.00
01B20492	11401013	Cordial, lime or green, regular, stronger than recommended dilution	0.00
01B20417	11401014	Cordial, lime or green, regular, recommended dilution	0.00
01B20460	11401015	Cordial, lime or green, regular, weaker than recommended dilution	0.00
01B20471	11401016	Cordial, orange or orange & mango, regular, stronger than recommended dilution	0.00
01B20434	11401017	Cordial, orange or orange & mango, regular, recommended dilution	0.00
01B20448	11401018	Cordial, orange or orange & mango, regular, weaker than recommended dilution	0.00
01B20416	11402001	Cordial, apple & berry, intense sweetened or diet, recommended dilution	0.00
01B20513	11402002	Cordial, blackcurrant juice, intense sweetened or diet, recommended dilution	0.00
01B20415	11402007	Cordial, 25% citrus fruit juice, intense sweetened or diet, recommended dilution	0.00
01B20480	11402003	Cordial, fruit cup or tropical, intense sweetened, recommended dilution	0.00
01B20437	11402004	Cordial, lemon, intense sweetened or diet, recommended dilution	0.00
01B20438	11402005	Cordial, lime or green, intense sweetened or diet, recommended dilution	0.00
01B20436	11402006	Cordial, orange or orange & mango, intense sweetened or diet, recommended dilution	0.00
01B20378	11309001	Fruit drink, from dry base, regular, recommended dilution	0.00
01B20497	11308001	Fruit drink, aloe vera, commercial	0.00
01B20440	11307001	Fruit drink, apple juice, commercial	0.00
01B20407	11307002	Fruit drink, 25% apple juice, commercial	0.00
01B20372	11307003	Fruit drink, 35% apple juice, commercial, added vitamin C	0.00
01B20449	11307004	Fruit drink, apple & blackcurrant, commercial	0.00
01B20488	11307005	Fruit drink, apple & mango, commercial	0.00
01B20404	11307006	Fruit drink, apricot juice, commercial	0.00
01B30322	11307007	Fruit drink, blackcurrant, commercial	0.00
01B20442	11307008	Fruit drink, cranberry, commercial	0.00
01B20443	11307009	Fruit drink, cranberry & blackcurrant, commercial	0.00
01B20496	11307010	Fruit drink, guava, commercial	0.00
01B20373	11307011	Fruit drink, lemon or lime juice, commercial	0.00
01B30376	11307012	Fruit drink, mango, commercial	0.00
01B20408	11307014	Fruit drink, 25% orange juice, commercial	0.00
01B20419	11307015	Fruit drink, 25% orange juice, commercial, added vitamin C	0.00
01B20374	11307016	Fruit drink, 35% orange juice, commercial, added vitamin C	0.00
01B20441	11307013	Fruit drink, orange juice, commercial	0.00

01B20375	11307017	Fruit drink, orange & mango juices, commercial	0.00
01B20376	11307018	Fruit drink, pineapple juice, commercial	0.00
01B20377	11307019	Fruit drink, tropical style, commercial	0.00
01B20490	32401001	Juice, infant, apple or pear, commercial	0.00
01B20491	32401002	Juice, infant, orange or tropical, commercial	0.00
01B30392	11304001	Juice, aloe vera, commercial	0.00
01B30321	11301002	Juice, apple, commercial, added vitamin C	0.00
01B30355	11301003	Juice, apple, commercial, no added vitamin C	0.00
01B30356	11301001	Juice, apple, commercial	0.00
01B30344	11303001	Juice, apple, commercial, added vitamins C, E & folate & fibre	0.00
01B30371	11302001	Juice, apple, home squeezed	0.00
01B30350	11301005	Juice, apple, sparkling, commercial, added vitamin C	0.00
01B30348	11301006	Juice, apple, sparkling, commercial, no added vitamin C	0.00
01B30357	11301004	Juice, apple, sparkling, commercial	0.00
01B30384	11302002	Juice, apple & berries, home squeezed	0.00
01B30337	11301007	Juice, apple & blackcurrant, commercial	0.00
01B30366	11301008	Juice, apple & forest fruits, commercial	0.00
01B30365	11301009	Juice, apple & mango, commercial	0.00
01B30333	11304002	Juice, carrot, commercial	0.00
01B30372	11305001	Juice, carrot, home squeezed	0.00
01B30334	11304003	Juice, celery, commercial	0.00
01B30342	11303002	Juice, fruit blend, 70% apple juice & 30% orange, grape & other juices, commercial, added vitamins A, C & folate	0.00
01B30341	11303003	Juice, fruit blend, 60% orange juice & 40% apple & pineapple juices, commercial, added Ca & vitamin C	0.00
01B30361	11303004	Juice, fruit blend, 60% orange juice & 40% apple & pineapple juices, commercial, added vitamin C & folate	0.00
01B30363	11303005	Juice, fruit blend, 60% orange juice & 40% apple & pineapple juices, commercial, added fibre & vitamin C	0.00
01B30375	11303006	Juice, fruit blend, 60% orange juice, 20% apple juice & other juices, commercial, added vitamins A, C & E	0.00
01B30393	11301010	Juice, fruit blend, 45% orange juice & 55% apple, pineapple & other juices, commercial	0.00
01B30373	11303007	Juice, fruit blend, with added vitamin A & C & herbal extracts	0.00
01B30396	11303008	Juice, fruit blend, breakfast, commercial, not further defined	0.00
01B30379	11302003	Juice, fruit blend, home squeezed	0.00
01B30381	11306001	Juice, fruit & vegetable blend, apple, berries, beetroot & carrot, commercial	0.00
01B30374	11306002	Juice, fruit & vegetable blend, apple, carrot, mango, orange, pineapple & passionfruit, commercial, added fibre & vitamin C	0.00

01B30386	11306003	Juice, fruit & vegetable blend, home squeezed, not further defined	0.00
01B30399	11301011	Juice, goji, commercial	0.00
01B30323	11301012	Juice, grape, commercial	0.00
01B30347	29205001	Juice, grape, sparkling, white, non-alcoholic, commercial	0.00
01B30378	11301013	Juice, grape, commercial, concentrate	0.00
01B30324	11301014	Juice, grapefruit, commercial	0.00
01B30325	11301015	Juice, lemon, commercial	0.00
01B30367	11302004	Juice, lemon, home squeezed	0.00
01B30326	11302005	Juice, lime, commercial or home squeezed	0.00
01B30354	11301018	Juice, orange, commercial, fresh, no added vitamin C	0.00
01B30328	11301017	Juice, orange, commercial, fresh, added vitamin C	0.00
01B30353	11301019	Juice, orange, commercial, shelf stable, added vitamin C	0.00
01B30351	11301020	Juice, orange, commercial, sweetened, added vitamin C	0.00
01B30358	11301016	Juice, orange, commercial	0.00
01B30343	11303009	Juice, orange, commercial, added Ca & vitamins A, C & folate	0.00
01B30352	11301021	Juice, orange, commercial, concentrate only	0.00
01B30327	11302006	Juice, orange, home squeezed	0.00
01B30340	11301022	Juice, orange & mango, commercial	0.00
01B30330	11301023	Juice, pear, commercial	0.00
01B30331	11301025	Juice, pineapple, commercial, unsweetened	0.00
01B30332	11301026	Juice, pineapple, commercial, sweetened	0.00
01B30359	11301024	Juice, pineapple, commercial	0.00
01B30380	11301027	Juice, pomegranate, commercial	0.00
01B30397	11301028	Juice, prune, commercial	0.00
01B30335	11304005	Juice, tomato, commercial, added salt	0.00
01B30336	11304006	Juice, tomato, commercial, sweetened, added salt	0.00
01B30360	11304004	Juice, tomato, commercial	0.00
01B30364	11301029	Juice, tropical, commercial	0.00
01B30349	11304007	Juice, vegetable blend, tomato, carrot, celery & parsley, commercial	0.00
01B30385	11305002	Juice, vegetable blend, home squeezed	0.00
01B30398	11304009	Juice, wheatgrass, commercial	0.00
01B30387	11301030	Juice, fruit, commercial, not further defined	0.00
01B30401	11301035	Juice, fruit, commercial, not further defined	0.00
01B30389	11304008	Juice, vegetable, commercial, not further defined	0.00
01B30388	11301031	Juice, commercial, not further defined	0.00
01B20401	11702001	Mineral water, natural, plain or unflavoured	0.00
01B20392	11505001	Mineral water, citrus flavoured, regular	0.00
01B20453	11505002	Mineral water, non-citrus flavoured, regular	0.00
01B20393	11506001	Mineral water, flavoured, intense sweetened or diet	0.00
01B20390	11503001	Soft drink, cola flavour, regular	0.00
01B20462	11503002	Soft drink, cola flavour, regular, with ice	0.00

01B20391	11503003	Soft drink, cola flavour, regular, decaffeinated	0.00
01B20399	11504001	Soft drink, cola flavour, intense sweetened or diet	0.00
01B20465	11504002	Soft drink, cola flavour, intense sweetened or diet, with added ice	0.00
01B20409	11504003	Soft drink, cola flavour, intense sweetened or diet, decaffeinated	0.00
01B20420	11501001	Soft drink, creaming soda, regular	0.00
01B20425	11502001	Soft drink, creaming soda, intense sweetened or diet	0.00
01B20455	11501002	Soft drink, dry ginger ale or ginger beer, regular	0.00
01B20456	11502002	Soft drink, dry ginger ale or ginger beer, intense sweetened or diet	0.00
01B20504	11603001	Soft drink, energy drink, Hype	0.00
01B20503	11603002	Soft drink, energy drink, Monster	0.00
01B20470	11603003	Soft drink, energy drink, Mother	0.00
01B20467	11603004	Soft drink, energy drink, Red Bull	0.00
01B20502	11603005	Soft drink, energy drink, Red Eye	0.00
01B20507	11603006	Soft drink, energy drink, Rockstar	0.00
01B20469	11603007	Soft drink, energy drink, V	0.00
01B20402	11603008	Soft drink, energy drink, not further defined	0.00
01B20468	11604001	Soft drink, energy drink, intense sweetened, Red Bull	0.00
01B20501	11604002	Soft drink, energy drink, intense sweetened, V	0.00
01B20510	11604003	Soft drink, energy drink, intense sweetened, XS sugar-free	0.00
01B20515	11501003	Soft drink, grass jelly, all flavours	0.00
01B20406	11501004	Soft drink, lemon flavour, regular	0.00
01B20428	11502003	Soft drink, lemon flavour, intense sweetened or diet	0.00
01B20459	11501005	Soft drink, lemon, lime & bitters, regular	0.00
01B20386	11501006	Soft drink, lemonade, regular	0.00
01B20464	11501007	Soft drink, lemonade, regular, with added ice	0.00
01B20395	11502004	Soft drink, lemonade, intense sweetened or diet	0.00
01B20451	11501008	Soft drink, lime flavour, regular	0.00
01B20405	11501009	Soft drink, orange flavour, regular	0.00
01B20463	11501010	Soft drink, orange flavour, regular, with added ice	0.00
01B20429	11502005	Soft drink, orange flavour, intense sweetened or diet	0.00
01B20396	11502008	Soft drink, other fruit flavours, intense sweetened or diet	0.00
01B20421	11501011	Soft drink, passionfruit flavour, regular	0.00
01B20426	11502006	Soft drink, passionfruit flavour, intense sweetened or diet	0.00
01B20423	11501012	Soft drink, raspberry flavour, regular	0.00
01B20427	11502007	Soft drink, raspberry flavour, intense sweetened or diet	0.00
01B20511	11501017	Soft drink, ready to drink, added glucose & vitamin C (Lucozade)	0.00
01B20457	11501013	Soft drink, sarsaparilla, regular	0.00
01B20388	11501014	Soft drink, tea flavour, regular	0.00
01B10540	11104001	Iced tea, homemade, unsweetened	0.00
01B20389	11501015	Soft drink, tonic water, regular	0.00

01B20398	11502009	Soft drink, tonic water, intense sweetened or diet	0.00
01B20508	11501016	Soft drink, not further defined	0.00
01B20521	11501020	Soft drink, not further defined	0.00
01B20484	11503004	Soft drink, frozen, cola flavour, regular	0.00
01B20485	11501018	Soft drink, frozen, non-cola flavours, regular	0.00
01B20486	11501019	Soft drink, frozen, regular, not further defined	0.00
01B20499	11602001	Sports drink, dry powder, all flavours	0.00
01B20500	11601003	Sports drink, prepared from dry powder, weaker than standard dilution, all flavours	0.00
01B20394	11601001	Sports drink, ready to drink, all flavours	0.00
01B20498	11601002	Sports drink, ready to drink, sugar free, all flavours	0.00
01B10485	11106001	Tea, chai, instant dry powder	0.00
01B10392	11101006	Tea, chai, plain, without milk	0.00
01B10482	11101007	Tea, chai, flavoured, without milk	0.00
01B10390	11101003	Tea, green, plain, without milk	0.00
01B10391	11101004	Tea, green, flavoured, without milk	0.00
01B10334	11105001	Tea, herbal, chamomile, without milk	0.00
01B10333	11105002	Tea, herbal, lemon, without milk	0.00
01B10332	11105003	Tea, herbal, mint, without milk	0.00
01B10299	11105004	Tea, herbal, other, without milk	0.00
01B10404	11101005	Tea, jasmine, plain, without milk	0.00
01B10298	11101001	Tea, regular, black, brewed from leaf or teabags, plain, without milk	0.00
01B10330	11101002	Tea, regular, black, brewed from leaf or teabags, flavoured, without milk	0.00
01B10480	11102001	Tea, regular, white, brewed from leaf or teabags, with cows milk not further defined	0.00
01B10397	11103001	Tea, decaffeinated, black, brewed from leaf or teabags, plain, without milk	0.00
01B10419	11701001	Water, bore	0.00
01B10312	11702002	Water, bottled, still	0.00
01B20400	11702003	Water, bottled, carbonated or soda water	0.00
01B10521	11703001	Water, bottled, with added sugar, vitamins & minerals	0.00
01B10389	11701002	Water, filtered	0.00
01B10386	11701003	Water, ice	0.00
01B10387	11701004	Water, rainwater or tank water	0.00
01B10311	11701005	Water, tap	0.00
02C10127	13205001	Biscuit, savoury, corn cake, plain, salted	0.00
02C10147	13205002	Biscuit, savoury, corn cake, multigrain, salted	0.00
02C10116	13204001	Biscuit, savoury, rice cake, from brown rice, plain	0.00
02C10141	13204002	Biscuit, savoury, rice cake, from brown rice, flavoured	0.00
02C10132	13204005	Biscuit, savoury, rice cracker, cheese based flavours	0.00
02C10130	13204008	Biscuit, savoury, rice cracker, other flavours	0.00
02C10131	13204004	Biscuit, savoury, rice cracker, plain	0.00
02C10133	13204007	Biscuit, savoury, rice cracker, sour cream based flavours	0.00
02C10129	13204006	Biscuit, savoury, rice cracker, seaweed flavoured	0.00

02C10152	13204009	Biscuit, savoury, rice cracker, from brown rice, all flavours	0.00
02C10153	13204010	Biscuit, savoury, rice cracker, not further defined	0.00
02C10124	13202001	Biscuit, savoury, from white wheat flour, cheese-flavoured	0.00
02C10149	13205003	Biscuit, savoury, corn, crispbread, puffed & toasted	0.00
02C10125	13202002	Biscuit, savoury, from white wheat flour, other flavours	0.00
02C10121	13203001	Biscuit, savoury, from rye flour, crispbread	0.00
02C10148	13203002	Biscuit, savoury, from rye flour, crispbread, with added grains (buckwheat, linseed, soy, sesame)	0.00
02C10120	13201001	Biscuit, savoury, from wheat flour, crispbread, puffed & toasted	0.00
02C10136	13201002	Biscuit, savoury, from wheat flour, crispbread, puffed & toasted, reduced fat	0.00
02C10122	13202005	Biscuit, savoury, from white wheat flour, plain snack cracker style	0.00
02C10151	13201003	Biscuit, savoury, from white wheat flour, plain snack cracker style, reduced fat	0.00
02C10117	13202003	Biscuit, savoury, from white wheat flour, flaky cracker style	0.00
02C10157	13202004	Biscuit, savoury, from white wheat flour, flatbread style	0.00
02C10118	13201004	Biscuit, savoury, from white wheat flour, Salada style	0.00
02C10135	13201005	Biscuit, savoury, from white wheat flour, Salada style, reduced fat	0.00
02C10128	13202006	Biscuit, savoury, from white wheat flour, stick, salted	0.00
02C10163	13201006	Biscuit, savoury, from white wheat flour, wafer style	0.00
02C10119	13201007	Biscuit, savoury, from white wheat flour, water cracker style	0.00
02C10150	13202007	Biscuit, savoury, from white wheat flour, plain, not further defined	0.00
02C10113	13201008	Biscuit, savoury, from wholemeal wheat flour	0.00
02C10114	13201009	Biscuit, savoury, from wholemeal wheat flour, added grains	0.00
02C10123	13201010	Biscuit, savoury, from wholemeal wheat flour, crispbread	0.00
02C10138	13201011	Biscuit, savoury, from wholemeal wheat flour, Salada style, high fibre	0.00
02C10115	13201012	Biscuit, savoury, from wholemeal wheat flour & rye flour, crispbread, puffed	0.00
02C10162	13204011	Biscuit, savoury, rice flour, peanut cracker	0.00
02C10160	13201013	Biscuit, savoury, not further defined	0.00
02C10164	26401001	Cracker, with cheddar cheese	0.00
02C10165	26401002	Cracker, with cheese not further defined	0.00
02C10161	26401011	Snack pack, savoury, corn & rice biscuit with salsa	0.00
02C10146	26401012	Snack pack, savoury, rice cracker with reduced fat cheddar cheese	0.00

02C10145	26401013	Snack pack, savoury, white wheat flour biscuit with cheese	0.00
02C10155	26401014	Snack pack, savoury, white wheat flour biscuit with processed cheese spread	0.00
02C10159	26401015	Snack pack, savoury, white wheat flour biscuit with processed vegemite flavoured cheese spread	0.00
05D10415	15602008	Snack pack, savoury, white wheat flour biscuit with tuna	0.00
02C10137	26401016	Snack pack, savoury, wholemeal wheat flour biscuit with processed cheese spread	0.00
12C10474	27304001	Biscuit filling, cream style, chocolate flavoured, commercial	0.00
12C10473	27304002	Biscuit filling, cream style, vanilla flavoured, commercial	0.00
12C10476	27304003	Biscuit filling, icing style, vanilla flavoured, commercial	0.00
02C20340	13102001	Biscuit, sweet, almond spekulatius (butter almond cookie), commercial	0.00
02C20283	13101001	Biscuit, sweet, Anzac style, commercial	0.00
02C20313	13101002	Biscuit, sweet, Anzac style, homemade from basic ingredients, fat not further defined	0.00
02C20335	13102002	Biscuit, sweet, biscotti (almond bread), commercial	0.00
02C20318	13106001	Biscuit, sweet, biscuit base, caramel filling, chocolate-coated, commercial	0.00
02C20319	13106002	Biscuit, sweet, biscuit base, mint filling, chocolate-coated, commercial	0.00
02C20324	13101003	Biscuit, sweet, Butternut Snap style, commercial	0.00
02C20308	13105001	Biscuit, sweet, chocolate chip, commercial	0.00
02C20323	13105002	Biscuit, sweet, chocolate chip, reduced fat, commercial	0.00
02C20315	13105003	Biscuit, sweet, chocolate chip, homemade from basic ingredients, fat not further defined	0.00
02C20346	13105004	Biscuit, sweet, chocolate chip & caramel, commercial	0.00
02C20307	13105005	Biscuit, sweet, chocolate chip & nut, commercial	0.00
02C20284	13101004	Biscuit, sweet, chocolate flavoured, commercial	0.00
02C20373	13101005	Biscuit, sweet, chocolate flavoured, homemade from basic ingredients, fat not further defined	0.00
02C20326	13101006	Biscuit, sweet, chocolate flavoured, bite-size shell, unfilled for pods recipes, commercial	0.00
02C20329	13106003	Biscuit, sweet, chocolate flavoured, bite-size shell, filled with layers of caramel & chocolate, commercial	0.00
02C20352	13105006	Biscuit, sweet, gluten free, chocolate flavoured with chocolate chips	0.00
02C20390	13101007	Biscuit, sweet, fortune cookie, commercial	0.00
02C20296	13102003	Biscuit, sweet, fruit-filled, commercial	0.00
02C20321	13102004	Biscuit, sweet, fruit-filled, commercial, reduced fat	0.00
02C20294	13102009	Biscuit, sweet, with dried fruit, commercial	0.00
02C20295	13102010	Biscuit, sweet, with dried fruit & icing, commercial	0.00
02C20285	13101008	Biscuit, sweet, ginger flavoured, commercial	0.00
02C20336	13101009	Biscuit, sweet, gingerbread, homemade from basic ingredients, fat not further defined, uniced	0.00

02C20387	13103001	Biscuit, sweet, gingerbread, homemade from basic ingredients, fat not further defined, iced	0.00
02C20374	13101010	Biscuit, sweet, honey jumble, homemade from basic ingredients, fat not further defined, uniced	0.00
02C20375	13103002	Biscuit, sweet, honey jumble, homemade from basic ingredients, fat not further defined, iced	0.00
02C20298	13103003	Biscuit, sweet, jam-filled, commercial	0.00
02C20322	13103004	Biscuit, sweet, jam-filled, reduced fat, commercial	0.00
02C20345	13103005	Biscuit, sweet, jam-filled, homemade from basic ingredients, fat not further defined	0.00
02C20391	13104001	Biscuit, sweet, macaron (almond based), filled	0.00
02C20299	13102006	Biscuit, sweet, macaroon (coconut based), commercial	0.00
02C20388	13102007	Biscuit, sweet, macaroon (coconut based), homemade from basic ingredients	0.00
02C20301	13103006	Biscuit, sweet, marshmallow filling, commercial	0.00
02C20311	13105007	Biscuit, sweet, marshmallow filling, chocolate-coated, commercial	0.00
02C20288	13101011	Biscuit, sweet, oatmeal, commercial	0.00
02C20287	13101012	Biscuit, sweet, plain, commercial	0.00
02C20300	13103007	Biscuit, sweet, plain, with icing, commercial	0.00
02C20366	13101013	Biscuit, sweet, plain, homemade from basic ingredients, butter or dairy blend	0.00
02C20364	13101014	Biscuit, sweet, plain, homemade from basic ingredients, fat not further defined	0.00
02C20370	13103008	Biscuit, sweet, plain, homemade from basic ingredients, fat not further defined, iced	0.00
02C20309	13105008	Biscuit, sweet, plain, chocolate-coated, commercial	0.00
02C20306	13107001	Biscuit, sweet, plain, carob-coated, commercial	0.00
02C20353	13101015	Biscuit, sweet, plain, sponge finger, commercial	0.00
02C20357	13102005	Biscuit, sweet, gluten free, non-chocolate flavour	0.00
02C10143	13204003	Biscuit, savoury, rice cake, from brown rice, carob coated	0.00
02C20293	13102008	Biscuit, sweet, with coconut, commercial	0.00
02C20342	13105009	Biscuit, sweet, with coconut, chocolate base, commercial	0.00
02C20291	13102012	Biscuit, sweet, with nuts, commercial	0.00
02C20383	13102013	Biscuit, sweet, with nuts, reduced fat, commercial	0.00
02C20371	13102014	Biscuit, sweet, with nuts, homemade from basic ingredients, fat not further defined	0.00
02C20367	13102015	Biscuit, sweet, with peanuts, homemade from basic ingredients, fat not further defined	0.00
02C20292	13102011	Biscuit, sweet, with dried fruit & nuts, commercial	0.00
02C20325	13104002	Biscuit, sweet, sandwich, butternut snap biscuit, chocolate cream filling, commercial	0.00
02C20316	13106004	Biscuit, sweet, sandwich, cream filling, chocolate-coated, commercial	0.00
02C20354	13106005	Biscuit, sweet, sandwich, cream & caramel filling, chocolate-coated, commercial	0.00

02C20303	13104005	Biscuit, sweet, sandwich, cream & jam filling, commercial	0.00
02C20302	13104003	Biscuit, sweet, sandwich, chocolate flavour biscuit, cream filling, commercial	0.00
02C20377	13104004	Biscuit, sweet, sandwich, chocolate flavoured shortbread style biscuit, chocolate icing filling, commercial	0.00
02C20304	13104008	Biscuit, sweet, sandwich, vanilla flavour biscuit, cream filling, commercial	0.00
02C20356	13104009	Biscuit, sweet, sandwich, vanilla flavour biscuit, cream filling, white chocolate coated, commercial	0.00
02C20331	13104006	Biscuit, sweet, sandwich, shortbread style biscuit, icing filling, commercial	0.00
02C20333	13104007	Biscuit, sweet, sandwich, shortbread style biscuit, icing filling, homemade from basic ingredients	0.00
02C20334	13103009	Biscuit, sweet, sandwich, syrup wafer, commercial	0.00
02C20305	13104011	Biscuit, sweet, sandwich, wafer layers, cream filling, non-chocolate flavours, commercial	0.00
02C20320	13104010	Biscuit, sweet, sandwich, wafer layers, cream filling, chocolate flavoured, commercial	0.00
02C20351	13106006	Biscuit, sweet, sandwich, wafer layers, cream filling, chocolate flavoured, chocolate coated, commercial	0.00
02C20286	13101016	Biscuit, sweet, shortbread style, commercial	0.00
02C20314	13101017	Biscuit, sweet, shortbread style, homemade from basic ingredients, butter or dairy blend	0.00
02C20330	13101018	Biscuit, sweet, shortbread style, with custard powder, commercial	0.00
02C20332	13101019	Biscuit, sweet, shortbread style, with custard powder, homemade from basic ingredients, fat not further defined	0.00
02C20297	13101020	Biscuit, sweet, sugar-glazed pastry, commercial	0.00
02C20347	13101021	Biscuit, sweet, wafer style	0.00
02C20289	13101022	Biscuit, sweet, wheatmeal, commercial	0.00
02C20359	13105010	Biscuit, sweet, wheatmeal, chocolate-coated, commercial	0.00
02C20382	13106007	Biscuit, sweet, chocolate, commercial, not further defined	0.00
02C20339	13104013	Biscuit, sweet, cream assorted, commercial, not further defined	0.00
02C20349	13101023	Biscuit, sweet, plain assorted, commercial, not further defined	0.00
02C20344	13104012	Biscuit, sweet, sandwich, wafer layers, cream filling, commercial, not further defined	0.00
02C20380	13101024	Biscuit, sweet, not further defined	0.00
02C20312	13101025	Biscuit mix, sweet, dry mix	0.00
07B20032	32202001	Infant rusk, teething	0.00
02C20290	13101026	Cone, wafer style, for ice cream	0.00
02C20327	13101027	Cone, waffle style, for ice cream	0.00
02C20350	13101028	Cone, for ice cream, not further defined	0.00

02B10607	12201001	Bagel, from white flour, commercial	0.00
02B10598	12201002	Bagel, from white flour, commercial, toasted	0.00
02B10723	12305001	Bagel, fruit, commercial	0.00
02B10739	12305002	Bagel, fruit, commercial, toasted	0.00
02B10641	12201003	Breadcrumbs, white	0.00
02B10610	12302001	Bread, chapatti, commercial	0.00
02B10796	12214001	Bread, corn	0.00
02B10793	12201004	Bread, croutons, commercial or homemade	0.00
02B10746	12201005	Bread, damper, from white flour, commercial	0.00
02B10747	12201006	Bread, damper, from white flour, commercial, toasted	0.00
02B10599	12203001	Bread, damper, from white flour, homemade from basic ingredients	0.00
02B10815	12203024	Bread, damper, from white flour, homemade from basic ingredients	0.00
02B10808	12202011	Bread, damper, from white Jackaroo flour, added vitamins & minerals, homemade from basic ingredients	0.00
02B10709	12207001	Bread, damper, from wholemeal flour, commercial	0.00
02B10710	12207002	Bread, damper, from wholemeal flour, commercial, toasted	0.00
02B10708	12209001	Bread, damper, from wholemeal flour, homemade from basic ingredients	0.00
02B10611	12302002	Bread, flat (pita or Lebanese), white, commercial	0.00
02B10721	12302003	Bread, flat (pita or Lebanese), white, commercial, toasted	0.00
02B10612	12302004	Bread, flat (pita or Lebanese), wholemeal, commercial	0.00
02B10722	12302005	Bread, flat (pita or Lebanese), wholemeal, commercial, toasted	0.00
02B10788	12303001	Bread, flat wrap, corn, commercial	0.00
02B10789	12303002	Bread, flat wrap, corn, commercial, toasted	0.00
02B10717	12302006	Bread, flat wrap, white, commercial	0.00
02B10718	12302007	Bread, flat wrap, white, commercial, toasted	0.00
02B10802	12302008	Bread, flat wrap or tortilla, mixed grain	0.00
02B10803	12302009	Bread, flat wrap or tortilla, mixed grain, toasted	0.00
02B10792	12303003	Bread, flat wrap or tortilla, rice, commercial	0.00
02B10730	12303004	Bread, flat wrap or tortilla, rye, commercial	0.00
02B10731	12303005	Bread, flat wrap or tortilla, rye, commercial, toasted	0.00
02B10719	12302010	Bread, flat wrap or tortilla, wholemeal, commercial	0.00
02B10720	12302011	Bread, flat wrap or tortilla, wholemeal, commercial, toasted	0.00
02B10786	12302015	Bread, flat, not further defined, commercial	0.00
02B10787	12302016	Bread, flat, not further defined, commercial, toasted	0.00
02B10760	12303006	Bread, tortilla, corn, commercial	0.00
02B10761	12303007	Bread, tortilla, corn, commercial, toasted	0.00
02B10790	12302013	Bread, tortilla, white, commercial	0.00
02B10791	12302014	Bread, tortilla, white, commercial, toasted	0.00
02B10798	12214009	Bread, tortilla, for use in Mexican recipes	0.00
02B10660	12201007	Bread, focaccia, plain, commercial	0.00

02B10661	12201008	Bread, focaccia, plain, commercial, toasted	0.00
02B10633	12210001	Bread, from rye flour, dark, commercial	0.00
02B10634	12210002	Bread, from rye flour, dark, commercial, toasted	0.00
02B10635	12210003	Bread, from rye flour, light, commercial	0.00
02B10636	12210004	Bread, from rye flour, light, commercial, toasted	0.00
02B10744	12210007	Bread, from rye flour, added grains, commercial	0.00
02B10745	12210008	Bread, from rye flour, added grains, commercial, toasted	0.00
02B10780	12212001	Bread, from rye flour, organic, commercial	0.00
02B10781	12212002	Bread, from rye flour, organic, commercial, toasted	0.00
02B10756	12210005	Bread, from rye flour, sour dough, commercial	0.00
02B10757	12210006	Bread, from rye flour, sour dough, commercial, toasted	0.00
02B10784	12212003	Bread, from rye flour, homemade from basic ingredients	0.00
02B10785	12212004	Bread, from rye flour, homemade from basic ingredients, toasted	0.00
02B10690	12201009	Bread, from spelt flour, commercial	0.00
02B10691	12201010	Bread, from spelt flour, commercial, toasted	0.00
02B10603	12201011	Bread, from white flour, commercial	0.00
02B10604	12201012	Bread, from white flour, commercial, toasted	0.00
02B10613	12202001	Bread, from white flour, commercial, added calcium	0.00
02B10614	12202002	Bread, from white flour, commercial, added calcium, toasted	0.00
02B10617	12202003	Bread, from white flour, commercial, added fibre	0.00
02B10618	12202004	Bread, from white flour, commercial, added fibre, toasted	0.00
02B10713	12202005	Bread, from white flour, commercial, added fibre & vitamins B1, B3, B6 & E, Fe & Zn	0.00
02B10714	12202006	Bread, from white flour, commercial, added fibre & vitamins B1, B3, B6 & E, Fe & Zn, toasted	0.00
02B10619	12202007	Bread, from white flour, commercial, added iron	0.00
02B10620	12202008	Bread, from white flour, commercial, added iron, toasted	0.00
02B10623	12202009	Bread, from white flour, commercial, added omega-3 polyunsaturates	0.00
02B10624	12202010	Bread, from white flour, commercial, added omega-3 polyunsaturates, toasted	0.00
02B10805	12202012	Bread, from white Jackaroo flour, commercial, added vitamins B1 & folate & Fe	0.00
02B10809	12202013	Bread, from white Jackaroo flour, commercial, added vitamins B1 & folate & Fe, toasted	0.00
02B10687	12201013	Bread, from white flour, commercial, low GI	0.00
02B10688	12201014	Bread, from white flour, commercial, low GI, toasted	0.00
02B10741	12201015	Bread, from white flour, chia seeds, commercial	0.00
02B10742	12201016	Bread, from white flour, chia seeds, commercial, toasted	0.00
02B10668	12201017	Bread, from white flour, ciabatta, commercial	0.00
02B10669	12201018	Bread, from white flour, ciabatta, commercial, toasted	0.00

02B10684	12204013	Bread, from white flour, extra grainy & seeds, commercial	0.00
02B10686	12204014	Bread, from white flour, extra grainy & seeds, commercial, toasted	0.00
02B10666	12201019	Bread, from white flour, French stick or baguette, commercial	0.00
02B10667	12201020	Bread, from white flour, French stick or baguette, commercial, toasted	0.00
02B10670	12201021	Bread, from white flour, pane di casa, commercial	0.00
02B10671	12201022	Bread, from white flour, pane di casa, commercial, toasted	0.00
02B10648	12203003	Bread, from white flour, organic, commercial	0.00
02B10647	12203004	Bread, from white flour, organic, commercial, toasted	0.00
02B10664	12201023	Bread, from white flour, sour dough, commercial	0.00
02B10665	12201024	Bread, from white flour, sour dough, commercial, toasted	0.00
02B10736	12203005	Bread, from white flour, sour dough, homemade from basic ingredients	0.00
02B10737	12203006	Bread, from white flour, sour dough, homemade from basic ingredients, toasted	0.00
02B10595	12203007	Bread, from white flour, homemade from basic ingredients, added salt	0.00
02B10596	12203008	Bread, from white flour, homemade from basic ingredients, added salt, toasted	0.00
02B10749	12203009	Bread, from white flour, commercial, fresh, not further defined	0.00
02B10750	12203010	Bread, from white flour, commercial, toasted, not further defined	0.00
02B10694	12203011	Bread, from white flour, for homemade sandwiches	0.00
02B10813	12203025	Bread, from white flour, for homemade sandwiches	0.00
02B10695	12203012	Bread, from white flour, for homemade sandwiches, toasted	0.00
02B10814	12203026	Bread, from white flour, for homemade sandwiches, toasted	0.00
02B10625	12201025	Bread roll, from white flour, commercial	0.00
02B10626	12201026	Bread roll, from white flour, commercial, toasted	0.00
02B10655	12201027	Bread roll, from white flour, fast food style	0.00
02B10605	12207003	Bread, from wholemeal flour, commercial	0.00
02B10606	12207004	Bread, from wholemeal flour, commercial, toasted	0.00
02B10627	12208001	Bread, from wholemeal flour, commercial, added fibre	0.00
02B10807	12208002	Bread, from wholemeal flour, commercial, added fibre, toasted	0.00
02B10715	12208003	Bread, from wholemeal flour, commercial, added fibre & vitamins B1, B3, B6 & E, Fe & Zn	0.00
02B10716	12208004	Bread, from wholemeal flour, commercial, added fibre & vitamins B1, B3, B6 & E, Fe & Zn, toasted	0.00
02B10656	12208005	Bread, from wholemeal flour, commercial, added iron	0.00
02B10657	12208006	Bread, from wholemeal flour, commercial, added iron, toasted	0.00

02B10658	12208007	Bread, from wholemeal flour, commercial, added omega-3 polyunsaturates	0.00
02B10659	12208008	Bread, from wholemeal flour, commercial, added omega-3 polyunsaturates, toasted	0.00
02B10734	12207005	Bread, from wholemeal flour, added seeds, commercial	0.00
02B10735	12207006	Bread, from wholemeal flour, added seeds, commercial, toasted	0.00
02B10726	12207007	Bread, from wholemeal flour, mixed grain & seeds, commercial	0.00
02B10727	12207008	Bread, from wholemeal flour, mixed grain & seeds, commercial, toasted	0.00
02B10724	12207009	Bread, from wholemeal flour, mixed grain, with quinoa & flaxseeds, commercial	0.00
02B10725	12207010	Bread, from wholemeal flour, mixed grain, with quinoa & flaxseeds, commercial, toasted	0.00
02B10676	12207011	Bread, from wholemeal flour, extra grainy, four seeds, commercial	0.00
02B10677	12207012	Bread, from wholemeal flour, extra grainy, four seeds, commercial, toasted	0.00
02B10681	12207013	Bread, from wholemeal flour, extra grainy & seeds, commercial	0.00
02B10685	12207014	Bread, from wholemeal flour, extra grainy & seeds, commercial, toasted	0.00
02B10678	12207015	Bread, from wholemeal flour, extra grainy & seeds, added oats, commercial	0.00
02B10689	12207016	Bread, from wholemeal flour, extra grainy & seeds, added oats, commercial, toasted	0.00
02B10649	12209002	Bread, from wholemeal flour, organic, commercial	0.00
02B10782	12209003	Bread, from wholemeal flour, organic, commercial, toasted	0.00
02B10751	12209004	Bread, from wholemeal flour, commercial, fresh, not further defined	0.00
02B10752	12209005	Bread, from wholemeal flour, commercial, toasted, not further defined	0.00
02B10600	12209006	Bread, from wholemeal flour, homemade from basic ingredients, added salt	0.00
02B10601	12209007	Bread, from wholemeal flour, homemade from basic ingredients, added salt, toasted	0.00
02B10696	12209008	Bread, from wholemeal flour, for homemade sandwiches	0.00
02B10697	12209009	Bread, from wholemeal flour, for homemade sandwiches, toasted	0.00
02B10711	12207017	Bread, from wholemeal flour, sour dough, commercial	0.00
02B10712	12207018	Bread, from wholemeal flour, sour dough, commercial, toasted	0.00
02B10631	12207021	Bread roll, from wholemeal flour, commercial	0.00
02B10632	12207022	Bread roll, from wholemeal flour, commercial, toasted	0.00
02B10679	12207019	Bread, from white & wholemeal flour, commercial	0.00

02B10680	12207020	Bread, from white & wholemeal flour, commercial, toasted	0.00
02B10646	12203013	Bread, from white or wholemeal flour, organic, commercial	0.00
02B10783	12203014	Bread, from white or wholemeal flour, organic, commercial, toasted	0.00
02B10800	12206001	Bread, from white or wholemeal flour, organic, added grains, commercial	0.00
02B10801	12206002	Bread, from white or wholemeal flour, organic, added grains, commercial, toasted	0.00
02F30204	12307001	Bread, garlic or herb, commercial, cooked	0.00
02F30313	12307002	Bread, garlic or herb, homemade, cooked	0.00
02B10644	12213001	Bread, gluten free, commercial	0.00
02B10645	12213002	Bread, gluten free, commercial, toasted	0.00
02B10758	12213003	Bread, gluten free, with added grains, commercial	0.00
02B10759	12213004	Bread, gluten free, with added grains, commercial, toasted	0.00
02B10597	12213005	Bread, gluten free, homemade from basic ingredients	0.00
02B10602	12213006	Bread, gluten free, homemade from basic ingredients, toasted	0.00
02B10733	12203002	Bread, flat, injera	0.00
02B10811	12203027	Bread, johnny (jonny) cake, from white flour, homemade from basic ingredients	0.00
02B10650	12203015	Bread, Naan, commercial	0.00
02B10740	12203016	Bread, Naan, homemade	0.00
02B10637	12204001	Bread, mixed grain, commercial	0.00
02B10638	12204002	Bread, mixed grain, commercial, toasted	0.00
02B10682	12204003	Bread, mixed grain, added seeds, commercial	0.00
02B10683	12204004	Bread, mixed grain, added seeds, commercial, toasted	0.00
02B10706	12204005	Bread, mixed grain, extra grainy & seeds, added pumpkin seeds, commercial	0.00
02B10707	12204006	Bread, mixed grain, extra grainy & seeds, added pumpkin seeds, commercial, toasted	0.00
02B10702	12204007	Bread, mixed grain, French stick or baguette, commercial	0.00
02B10703	12204008	Bread, mixed grain, French stick or baguette, commercial, toasted	0.00
02B10753	12204009	Bread, mixed grain, commercial, fresh, not further defined	0.00
02B10754	12204010	Bread, mixed grain, commercial, toasted, not further defined	0.00
02B10674	12206003	Bread, mixed grain, homemade from basic ingredients, added salt	0.00
02B10675	12206004	Bread, mixed grain, homemade from basic ingredients, added salt, toasted	0.00
02B10698	12206005	Bread, mixed grain, for homemade sandwiches	0.00
02B10699	12206006	Bread, mixed grain, for homemade sandwiches, toasted	0.00

02B10700	12204011	Bread, mixed grain, for sandwiches purchased from a cafe, takeaway shop or canteen	0.00
02B10701	12204012	Bread, mixed grain, for sandwiches purchased from a cafe, takeaway shop or canteen, toasted	0.00
02B10639	12204017	Bread roll, mixed grain, commercial	0.00
02B10640	12204018	Bread roll, mixed grain, commercial, toasted	0.00
02B10732	12203017	Bread, paratha	0.00
02B10592	12203018	Bread, pizza base, commercial	0.00
02B10652	12203021	Bread, pizza base, homemade from basic ingredients	0.00
02B10653	12203019	Bread, pizza base, thick base, fast food-style	0.00
02B10654	12203020	Bread, pizza base, thin base, fast food-style	0.00
02B10642	12210009	Bread, pumpernickel, commercial	0.00
02B10643	12210010	Bread, pumpernickel, commercial, toasted	0.00
02B10795	12203022	Bread, pumpkin	0.00
02B10799	12203023	Bread, pumpkin, toasted	0.00
02B10651	12302012	Bread, Roti, commercial	0.00
02B10672	12204015	Bread, soy & linseed, commercial	0.00
02B10673	12204016	Bread, soy & linseed, commercial, toasted	0.00
02B10662	12201028	Bread or bread roll, Turkish, plain, commercial	0.00
02B10663	12201029	Bread or bread roll, Turkish, plain, commercial, toasted	0.00
02B10748	12214002	Bread, commercial, fresh, not further defined	0.00
02B10810	12214010	Bread, fresh, not further defined	0.00
02B10755	12214003	Bread, commercial, toasted, not further defined	0.00
02B10812	12214011	Bread, toasted, not further defined	0.00
02B10704	12214004	Bread, homemade from basic ingredients, not further defined	0.00
02B10705	12214005	Bread, homemade from basic ingredients, not further defined, toasted	0.00
02B10729	12214006	Bread roll, commercial, fresh, not further defined	0.00
02B10797	12214007	Bread roll, for use in hamburger or hot dog recipes	0.00
02B10763	12214008	Bread roll, for use in subway sandwich recipes	0.00
02F30206	12304001	Bread or bread roll, topped/mixed with cheese	0.00
02F30207	12304002	Bread or bread roll, topped/mixed with cheese & bacon	0.00
02F30256	12304003	Bread or bread roll, topped/mixed with cheese & frankfurt	0.00
02F30257	12304004	Bread or bread roll, topped/mixed with cheese, meat & vegetables	0.00
02F30213	12304005	Bread or bread roll, topped/mixed with cheese & vegemite	0.00
02F30314	12304006	Bread or bread roll, topped/mixed with cheese & vegetables	0.00
02F30258	12304007	Bread or bread roll, topped/mixed with olives	0.00
02F30255	12304008	Bread or bread roll, topped/mixed with spinach & fetta	0.00
02B10615	12305003	Bread, from wheat flour, commercial, added dried fruit	0.00

02B10616	12305004	Bread, from wheat flour, commercial, added dried fruit, toasted	0.00
02E20102	12305005	Bread, from wheat flour, homemade from basic ingredients, added dried fruit	0.00
02E20103	12305006	Bread, from wheat flour, homemade from basic ingredients, added dried fruit, toasted	0.00
02E20109	12305007	Bun, sweet, chocolate or with chocolate chips, uniced	0.00
02E60369	12306001	Bun, sweet, steamed bun, filled with bean paste	0.00
02E20099	12306002	Bun, sweet, sticky cinnamon, with icing	0.00
02E20105	12306003	Bun, sweet, with custard, iced	0.00
02E20116	12305009	Bun, sweet, with fruit (other than sultanas), uniced	0.00
02E20117	12306005	Bun, sweet, with fruit (other than sultanas), iced	0.00
02E20118	12306006	Bun, sweet, with fruit (other than sultanas) & nuts, iced	0.00
02E20104	12306007	Bun, sweet, with mock cream & jam, uniced	0.00
02E20089	12305008	Bun, sweet, with sultanas, uniced	0.00
02E20090	12306004	Bun, sweet, with sultanas, iced	0.00
02E20121	12306008	Bun, sweet, with taro	0.00
02E20119	12306009	Bun, sweet, not further defined	0.00
02E20100	12307003	French toast, white bread, plain	0.00
02E20106	12307004	French toast, wholemeal bread, plain	0.00
02E20120	12305010	Panettone	0.00
02B20057	13606001	Crumpet, from white flour, commercial	0.00
02B20049	13606002	Crumpet, from white flour, commercial, toasted	0.00
02B20050	13606003	Crumpet, from wholemeal flour, commercial, toasted	0.00
02B20058	12301005	Muffin, English style, mixed grain, commercial, toasted	0.00
02B20053	12301001	Muffin, English style, from white flour, commercial, fresh/untoasted	0.00
02B20051	12301002	Muffin, English style, from white flour, commercial, toasted	0.00
02B20052	12301003	Muffin, English style, from white flour, commercial, added dried fruit, toasted	0.00
02B20056	12301004	Muffin, English style, from wholemeal flour, commercial, toasted	0.00
02F40236	23401001	Stuffing, bread-based, commercial	0.00
02D10290	11805001	Breakfast cereal, beverage, chocolate flavour, added vitamins A, B1, B2, B3, B6, B12, C & folate & Ca	0.00
02D10285	11805003	Breakfast cereal, beverage, non-chocolate flavours, added vitamins A, B1, B2, B3, B6, B12, C & folate & Ca	0.00
02D10344	11805004	Breakfast cereal, beverage, non-chocolate flavours, added vitamins A, B1, B2, B3, B6, B12, C & folate & Ca & Fe	0.00
02D10400	11805005	Breakfast cereal, beverage, non-chocolate flavours, natural sweetener, added vitamins A, B1, B2, B3, B6, B12, C & folate & Ca	0.00
02D10362	11805002	Breakfast cereal, beverage, chocolate flavour, added vitamins A, B1, B2, B3, B6, B12, C & folate & Ca & Fe	0.00
02D10409	11805006	Breakfast cereal, beverage, not further defined	0.00

02D10394	12516001	Breakfast cereal, barley, flakes, honey, added sultanas, unfortified	0.00
02D10296	12502001	Breakfast cereal, crispy corn pillows, honey, added vitamins B1, B2, B3 & folate	0.00
02D10289	12501002	Breakfast cereal, flakes of corn, no added sugar or salt, unfortified	0.00
02D10351	12501001	Breakfast cereal, flakes of corn, unfortified	0.00
02D10401	12502002	Breakfast cereal, flakes of corn, added vitamins B1, B2, B3 & folate	0.00
02D10266	12502003	Breakfast cereal, flakes of corn, added vitamins B1, B2, B3, C & folate, Fe & Zn	0.00
02D10352	12502004	Breakfast cereal, flakes of corn, added vitamins B1, B2, B3, B6, E & folate & Fe	0.00
02D10300	12502005	Breakfast cereal, flakes of corn, added vitamins B1, B2, B3 & folate & Fe	0.00
02D10369	12502006	Breakfast cereal, flakes of corn, with psyllium, added vitamins B1, B2, B3 & folate & Fe	0.00
02D10360	12502007	Breakfast cereal, flakes of corn, not further defined	0.00
02D10282	12502008	Breakfast cereal, flakes of corn, added nuts, added vitamins B1, B2, B3, C & folate, Fe & Zn	0.00
02D10398	12502009	Breakfast cereal, flakes of corn, added nuts, added vitamins B1, B2, B3, B6, folate & E	0.00
02D10372	12502010	Breakfast cereal, flakes of corn, added nuts, added vitamins B1, B2, B3 & folate & Fe	0.00
02D10301	12502011	Breakfast cereal, frosted flakes of corn, added vitamins B1, B2, B3, C, E & folate, Fe & Zn	0.00
02D10288	12501003	Breakfast cereal, puffed or popped corn, no added sugar or salt, unfortified	0.00
02D10406	12502012	Breakfast cereal, corn based, not further defined	0.00
02D10392	12514001	Breakfast cereal, mixed grain (barley & oat), flakes, honey, added fruit, nuts & seeds, unfortified	0.00
02D10393	12511001	Breakfast cereal, mixed grain (barley & soy), flakes, honey, unfortified	0.00
02D10277	12512001	Breakfast cereal, mixed grain (rice & wheat), flakes, added vitamins B1, B2, B3, B6 & folate, Ca, Fe & Zn	0.00
02D10303	12513010	Breakfast cereal, mixed grain (rice & wheat), flakes, almond & honey, vitamins B1, B2, B3, B6 & folate, Ca, Fe & Zn	0.00
02D10302	12515001	Breakfast cereal, mixed grain (rice & wheat), flakes, berries, added vitamins B1, B2, B3, B6 & folate, Ca, Fe & Zn	0.00
02D10309	12515002	Breakfast cereal, mixed grain (rice & wheat), flakes, fruit & nut, added vitamins B1, B2, B3, B6, E & folate, Ca, Fe & Zn	0.00
02D10395	12515003	Breakfast cereal, mixed grain (rice & wheat), flakes, nuts, added vitamins B1, B2 & B3, Ca & Fe	0.00
02D10332	12511002	Breakfast cereal, mixed grain (wheat & corn), flakes or extruded, no added salt, unfortified	0.00

02D10333	12515004	Breakfast cereal, mixed grain (wheat & corn), flakes, berries & sultanas, added vitamins A, B1, B2, B3, B6, E & folate, Ca, Fe & Zn	0.00
02D10334	12515005	Breakfast cereal, mixed grain (wheat & corn), flakes, sultana, apple & currant, added vitamins B1, B2, B3, B6, E & folate, Ca, Fe & Zn	0.00
02D10335	12513001	Breakfast cereal, mixed grain (wheat & corn), extruded, chocolate malt coating, added vitamins B1, B2, B3, B6, C, E & folate, Ca, Fe & Zn	0.00
02D10374	12515006	Breakfast cereal, mixed grain (wheat & oat), clusters, almond, added vitamins B1, B2, B3, B6, C, E & folate, Ca, Fe & Zn	0.00
02D10319	12514002	Breakfast cereal, mixed grain (wheat & oat), flakes, dried fruit, no added salt, unfortified	0.00
02D10376	12515007	Breakfast cereal, mixed grain (wheat & oat), flakes, apple, sultana & cranberry, added vitamins B1, B2, B3, B6, C, E & folate	0.00
02D10278	12515008	Breakfast cereal, mixed grain (wheat & oat), flakes, apricot & sultana, added vitamins B1, B2, B3 & folate & Fe	0.00
02D10307	12515009	Breakfast cereal, mixed grain (wheat & oat), flakes, berries, added vitamins B1, B2, B3 & folate & Fe	0.00
02D10312	12511003	Breakfast cereal, mixed grain (wheat, oat & corn), flakes or extruded, no added salt, unfortified	0.00
02D10324	12515011	Breakfast cereal, mixed grain (wheat, oat & corn), flakes, fruit, added vitamins B1, B2, B3, B6, E & folate, Ca, Fe & Zn	0.00
02D10403	12515012	Breakfast cereal, mixed grain (wheat, oat & corn), flakes, fruit & nuts, added vitamins B1, B2, B3, C & folate & Fe	0.00
02D10325	12515013	Breakfast cereal, mixed grain (wheat, oat & corn), flakes, fruit & nuts, added vitamins B1, B2, B3, B6, E & folate, Ca, Fe & Zn	0.00
02D10348	12515015	Breakfast cereal, mixed grain (wheat, oat & corn), flakes & clusters, pomegranate & berries, added vitamins A, B1, B2, B3, B6, E & folate, Ca, Fe & Zn	0.00
02D10339	12515010	Breakfast cereal, mixed grain (wheat, oat & corn), clusters, honey, nuts, added vitamins B1, B2, B3, E, C & folate, Fe & Zn	0.00
02D10280	12513002	Breakfast cereal, mixed grain (wheat, oat & corn), extruded, added vitamins B1, B2, B3, B6 & C, Ca & Fe	0.00
02D10371	12513003	Breakfast cereal, mixed grain (wheat, oat & corn), extruded, added vitamins B1, B2, B3, B6, C & folate, Fe & Zn	0.00
02D10346	12513004	Breakfast cereal, mixed grain (wheat, oat & corn), extruded, added vitamins B1, B2, B3, B6, C & folate, Ca, Fe & Zn	0.00
02D10413	12513007	Breakfast cereal, mixed grain (wheat, oat & corn), extruded, cocoa coating, added B1, B2, B3, B6, C & folate, Ca, Fe & Zn	0.00

02D10313	12513005	Breakfast cereal, mixed grain (wheat, oat & corn), extruded, fruit flavours, added vitamins B1, B2, B3, C & folate, Fe & Zn	0.00
02D10321	12514003	Breakfast cereal, mixed grain (wheat, rice & oat), flakes, dried fruit, no added salt, unfortified	0.00
02D10337	12514004	Breakfast cereal, mixed grain (wheat, rice & oat), flakes, cashew, almond, hazelnut & coconut, unfortified	0.00
02D10405	12515017	Breakfast cereal, mixed grain (wheat, rice & oat), flakes, dried fruit, added vitamins B1, B2, B3 & E & Fe	0.00
02D10322	12511004	Breakfast cereal, mixed grain (wheat, rice & oat), flakes, honey, unfortified	0.00
02D10375	12512005	Breakfast cereal, mixed grain (wheat, rice & oat), flakes, honey, added vitamins B1, B2, B3 & folate & Fe	0.00
02D10336	12514005	Breakfast cereal, mixed grain (wheat, rice & oat), flakes & clusters, apple & raisins, unfortified	0.00
02D10365	12515014	Breakfast cereal, mixed grain (wheat, rice & oat), flakes & clusters, apple, added vitamins B1, B2, B3, B6, E & folate, Fe & Zn	0.00
02D10318	12515016	Breakfast cereal, mixed grain (wheat, rice & oat), flakes & clusters, sultanas, added vitamins B1, B2, B3, B6, C & folate, Fe & Zn	0.00
02D10380	12513008	Breakfast cereal, mixed grain (wheat, corn & rice), extruded, cocoa coating, added vitamins B1, B2, B3, B6, C, E & folate, Ca, Fe, Mg & Zn	0.00
02D10314	12514006	Breakfast cereal, mixed grain (wheat, corn, rice & oat), flakes, added fruit &/or nuts, no added salt, unfortified	0.00
02D10338	12512004	Breakfast cereal, mixed grain (wheat, corn, rice & oat), flakes, added vitamins B1, B2, B3, B6, E & folate, Ca & Fe	0.00
02D10316	12515021	Breakfast cereal, mixed grain (wheat, corn, rice & oat), flakes, apricot & coconut, added vitamins B1, B2, B3, E & folate, Ca & Fe	0.00
02D10315	12515022	Breakfast cereal, mixed grain (wheat, corn, rice & oat), flakes, berries, added vitamins B1, B2, B3, E & folate, Ca & Fe	0.00
02D10350	12515023	Breakfast cereal, mixed grain (wheat, corn, rice & oat), flakes, fruit, added vitamins B1, B2, B3 & folate & Fe	0.00
02D10345	12515024	Breakfast cereal, mixed grain (wheat, corn, rice & oat), flakes, fruit, added vitamins B1, B2, B3, & folate, Ca & Fe	0.00
02D10279	12515025	Breakfast cereal, mixed grain (wheat, corn, rice & oat), flakes, fruit & nuts, added vitamins B1, B2, B3, C & folate & Fe	0.00
02D10363	12515020	Breakfast cereal, mixed grain (wheat, corn, rice & oat), flakes & clusters, honey & macadamias, added vitamins B1, B2, B3, E & folate, Ca & Fe	0.00
02D10308	12512002	Breakfast cereal, mixed grain (wheat, oat, rice & corn), clusters, added vitamins B1, B2, B3, C & folate, Fe & Zn	0.00

02D10330	12515018	Breakfast cereal, mixed grain (wheat, corn, rice & oat), clusters, apple & almonds, added vitamins B1, B2, B3 & folate & Fe	0.00
02D10343	12515019	Breakfast cereal, mixed grain (wheat, corn, rice & oat), clusters, sultana & berries, added vitamins B1, B2, B3, E & folate, Ca & Fe	0.00
02D10366	12513009	Breakfast cereal, mixed grain (wheat, corn, rice & oat), crispy pillows, cocoa coating, added vitamins B1, B2, B3 & folate	0.00
02D10326	12513006	Breakfast cereal, mixed grain (wheat, corn, rice & oat), extruded, honey, added vitamins B1, B2, B3, B6, C, E & folate, Ca, Fe & Zn	0.00
02D10331	12512003	Breakfast cereal, mixed grain (wheat, corn, rice & oat), extruded, added vitamins B1, B2, B3, B6, C, E & folate, Ca, Fe & Zn	0.00
02D10408	12515026	Breakfast cereal, mixed grain, commercial, not further defined	0.00
02D10368	12504001	Breakfast cereal, flakes of rice, added vitamins B1, B2, B3 & folate & Fe	0.00
02D10286	12503001	Breakfast cereal, puffed or popped rice, no added sugar or salt, unfortified	0.00
02D10402	12504002	Breakfast cereal, puffed or popped rice, added vitamins B1, B2 & B3 & Fe	0.00
02D10367	12504003	Breakfast cereal, puffed or popped rice, added vitamins B1, B3 & folate & Fe	0.00
02D10304	12504004	Breakfast cereal, puffed or popped rice, added vitamins B1, B2, B3 & folate & Fe	0.00
02D10267	12504005	Breakfast cereal, puffed or popped rice, added vitamins B1, B2, B3, C & folate, Fe & Zn	0.00
02D10370	12504006	Breakfast cereal, puffed or popped rice, not further defined	0.00
02D10305	12503002	Breakfast cereal, puffed or popped rice, cocoa coating, no added sugar or salt, unfortified	0.00
02D10383	12503003	Breakfast cereal, puffed or popped rice, cocoa coating, unfortified	0.00
02D10281	12504008	Breakfast cereal, puffed or popped rice, cocoa coating, added vitamins B1, B2, B3, C & folate, Ca, Fe & Zn	0.00
02D10306	12504007	Breakfast cereal, puffed or popped rice, cocoa coating, added vitamins B1, B2, B3 & folate & Fe	0.00
02D10410	12516002	Breakfast cereal, cocoa coating, commercial, not further defined	0.00
02D10404	12505001	Breakfast cereal, wheat bran, flakes, unfortified	0.00
02D10264	12506001	Breakfast cereal, wheat bran, flakes, added vitamins B1, B2 B3, B6, & folate, Fe & Zn	0.00
02D10340	12510001	Breakfast cereal, wheat bran, flakes, honey & almond, added vitamins B1, B2 B3, B6 & folate, Fe & Zn	0.00
02D10391	12510002	Breakfast cereal, wheat bran, flakes, sultanas, added vitamins B1, B2, B3 & folate & Fe	0.00
02D10275	12509001	Breakfast cereal, wheat bran, flakes, sultanas, added vitamins B1, B2, B3, B6 & folate, Fe & Zn	0.00

02D10263	12506002	Breakfast cereal, wheat bran, pellets, added vitamins B1, B2 & folate	0.00
02D10397	12506003	Breakfast cereal, wheat bran, pellets, added vitamins B1, B2, B3 & folate & Fe	0.00
02D10389	12506004	Breakfast cereal, wheat bran, pellets, added vitamins E & folate, Ca & Zn	0.00
02D10373	12506005	Breakfast cereal, wheat bran, pellets, added vitamins A, B1, B2, B3, E & folate, Ca & Fe	0.00
02D10399	12506006	Breakfast cereal, wheat bran, pellets, not further defined	0.00
02D10310	12510003	Breakfast cereal, wheat bran, small biscuit, with sultana paste, added vitamins B1, B2, B3, B6, E & folate, Fe & Zn	0.00
02D10349	12511005	Breakfast cereal, oat & whole wheat, biscuit, added salt, unfortified	0.00
02D10270	12505003	Breakfast cereal, whole wheat, biscuit, no added sugar, unfortified	0.00
02D10396	12505002	Breakfast cereal, whole wheat, biscuit, no added sugar or salt, unfortified	0.00
02D10291	12505004	Breakfast cereal, whole wheat, biscuit, added sugar & salt, unfortified	0.00
02D10268	12506009	Breakfast cereal, whole wheat, biscuit, added vitamins B1, B2, B3 & folate, Fe & Zn	0.00
02D10299	12506007	Breakfast cereal, whole wheat, biscuit, added vitamins B1, B2 & B3	0.00
02D10342	12506008	Breakfast cereal, whole wheat, biscuit, added vitamins B1, B2 & B3, Ca, Fe & Zn	0.00
02D10269	12506010	Breakfast cereal, whole wheat, biscuit, bran, added vitamins B1, B2, B3 & folate & Fe	0.00
02D10311	12512006	Breakfast cereal, whole wheat, biscuit, multigrain, added vitamins B1, B2, B3 & E & Fe	0.00
02D10361	12506011	Breakfast cereal, whole wheat, biscuit, not further defined	0.00
02D10271	12506014	Breakfast cereal, whole wheat, small biscuit, added vitamins B1, B2, B3 & folate	0.00
02D10293	12509003	Breakfast cereal, whole wheat, small biscuit, with berries, added vitamins B1, B2, B3 & folate, Ca & Fe	0.00
02D10292	12509002	Breakfast cereal, whole wheat, small biscuit, with apricot, added vitamins B1, B2, B3 & folate, Ca & Fe	0.00
02D10295	12509004	Breakfast cereal, whole wheat, small biscuit, with blackcurrant paste, added vitamins B1, B2, B3 & folate	0.00
02D10377	12509005	Breakfast cereal, whole wheat, small biscuit, with fruit paste, added vitamins B1, B2, B3 & C & Fe	0.00
02D10341	12507002	Breakfast cereal, whole wheat, small biscuit, with honey, added vitamins B1, B2, B3 & folate, Fe & Zn	0.00
02D10297	12505005	Breakfast cereal, whole wheat, flakes, no added sugar, unfortified	0.00
02D10273	12506012	Breakfast cereal, whole wheat, flakes, added vitamins B1, B2, B3 & folate	0.00

02D10298	12506013	Breakfast cereal, whole wheat, flakes, added vitamins B1, B2, B3, C & folate & Fe	0.00
02D10328	12508001	Breakfast cereal, whole wheat, flakes, added dried fruit &/or nuts, no added sugar, unfortified	0.00
02D10329	12509006	Breakfast cereal, whole wheat, flakes, dried fruit, added vitamins B1, B2, B3, C & folate	0.00
02D10274	12509007	Breakfast cereal, whole wheat, flakes, dried fruit & nuts, added fibre, vitamins B1, B2, B3 & folate, Ca & Fe	0.00
02D10272	12505006	Breakfast cereal, whole wheat, puffed, no added sugar or salt, unfortified	0.00
02D10364	12505007	Breakfast cereal, whole wheat, puffed, honey, unfortified	0.00
02D10347	12507001	Breakfast cereal, whole wheat, puffed, honey, added vitamins B1, B2, B3, B6, E & folate	0.00
02D10407	12506015	Breakfast cereal, wheat based, commercial, not further defined	0.00
02D10412	12516003	Breakfast cereal, not further defined	0.00
02E40122	13102016	Breakfast pastry, sweet, fruit-paste filled, commercial	0.00
02D10387	12514008	Muesli, commercial, toasted, added dried fruit, unfortified	0.00
02D10284	12514009	Muesli, commercial, toasted, added dried fruit & nuts, unfortified	0.00
02D10390	12515027	Muesli, commercial, toasted, added dried fruit & nuts, added vitamins B2, B3, E & folate & Ca	0.00
02D10378	12514010	Muesli, commercial, toasted, added nuts, unfortified	0.00
02D10385	12514011	Muesli, homemade, toasted, added dried fruit	0.00
02D10359	12514012	Muesli, homemade, toasted, added dried fruit & nuts	0.00
02D10357	12514013	Muesli, homemade, toasted, added dried fruit & seeds	0.00
02D10358	12514014	Muesli, homemade, toasted, added dried fruit, nuts & seeds	0.00
02D10382	12514015	Muesli, homemade, toasted, added nuts & seeds	0.00
02D10283	12514016	Muesli, commercial, untoasted or natural style, added dried fruit, unfortified	0.00
02D10388	12514017	Muesli, commercial, untoasted or natural style, added dried fruit, low fat, unfortified	0.00
02D10353	12514018	Muesli, commercial, untoasted or natural style, added dried fruit & nuts, unfortified	0.00
02D10379	12514019	Muesli, commercial, untoasted or natural style, added nuts & seeds	0.00
02D10384	12514020	Muesli, homemade, untoasted or natural, added dried fruit	0.00
02D10355	12514021	Muesli, homemade, untoasted or natural, added dried fruit & nuts	0.00
02D10356	12514022	Muesli, homemade, untoasted or natural, added dried fruit & seeds	0.00
02D10354	12514023	Muesli, homemade, untoasted or natural, added dried fruit, nuts & seeds	0.00
02D10381	12514024	Muesli, homemade, untoasted or natural, added nuts & seeds	0.00

02D10411	12514007	Muesli, commercial, gluten free	0.00
02A10393	12602001	Porridge, rice (congee), cooked with water	0.00
02A10500	12602002	Porridge, brown rice, with added dry fruit, cooked with cows milk	0.00
02A10389	12601001	Porridge, rolled oats, prepared with water	0.00
02A10398	12601002	Porridge, rolled oats, prepared with regular fat cows milk	0.00
02D20030	12601003	Porridge, rolled oats, prepared with reduced fat cows milk	0.00
02D20031	12601004	Porridge, rolled oats, prepared with skim cows milk	0.00
02A10497	12601005	Porridge, rolled oats, prepared with cows milk	0.00
02A10416	12601006	Porridge, rolled oats, prepared with rice milk	0.00
02A10466	12601007	Porridge, rolled oats, prepared with soy milk	0.00
02D20032	12601008	Porridge, rolled oats, prepared with cows milk & water	0.00
02D20037	12601009	Porridge, rolled oats, prepared with soy milk & water	0.00
02D20036	12601010	Porridge, rolled oats, with added fibre & Ca, prepared with cows milk	0.00
02A10455	12601012	Porridge, rolled oats mixed with sugar or honey & other flavours, prepared with cows milk	0.00
02A10435	12601011	Porridge, rolled oats mixed with sugar or honey & other flavours, prepared with water	0.00
02A10465	12601013	Porridge, rolled oats mixed with sugar or honey & other flavours, prepared with soy milk	0.00
02A10467	12601015	Porridge, rolled oats mixed with sugar, flavours & dried fruit, prepared with cows milk	0.00
02A10460	12601014	Porridge, rolled oats mixed with sugar, flavours & dried fruit, prepared with water	0.00
02A10498	12601016	Porridge, rolled oats mixed with sugar, flavours & dried fruit, prepared with soy milk	0.00
09A40065	12602004	Paste, flour mixed with sugar & cows milk	0.00
07B10086	32201001	Infant cereal, added vitamin C & Fe	0.00
07B10087	32201002	Infant cereal, added vitamin C & Fe, prepared with water	0.00
02E10554	13303001	Bread, banana, commercial	0.00
02E10555	13303002	Bread, banana, commercial, toasted	0.00
02E10585	13303003	Bread, banana, homemade	0.00
02E10586	13303004	Bread, banana, homemade, toasted	0.00
02E50322	13306003	Slice, brownie, chocolate, with nuts, commercial	0.00
02E50254	13306004	Slice, brownie, chocolate, with nuts, homemade from basic ingredients, fat not further defined	0.00
02E50253	13306005	Slice, brownie, chocolate, without nuts, homemade from basic ingredients, butter or dairy blend	0.00
02E50296	13306007	Slice, brownie, chocolate, without nuts, homemade from basic ingredients, fat not further defined	0.00
02E50295	13306006	Slice, brownie, chocolate, without nuts, homemade from basic ingredients, with oil	0.00
02E10435	13303005	Cake mix, plain, dry powder	0.00
02E10442	13301001	Cake mix, chocolate flavour, dry powder	0.00

02E10510	13303006	Cake mix, orange & poppy seed, dry powder	0.00
02E10450	13302001	Cake mix, sponge, dry powder	0.00
02E10529	13303007	Cake or cupcake, banana, commercial, uniced	0.00
02E10535	13303008	Cake or cupcake, banana, commercial, iced	0.00
02E10460	13303009	Cake or cupcake, banana, homemade from basic ingredients, butter or dairy blend, uniced	0.00
02E10536	13303010	Cake or cupcake, banana, homemade from basic ingredients, butter or dairy blend, iced	0.00
02E10652	13303011	Cake or cupcake, banana, homemade from basic ingredients, margarine, uniced	0.00
02E10592	13303012	Cake or cupcake, banana, homemade from basic ingredients, oil, uniced	0.00
02E10593	13303013	Cake or cupcake, banana, homemade from basic ingredients, undefined fat, uniced	0.00
02E10595	13303014	Cake or cupcake, banana, homemade from basic ingredients, undefined fat, iced	0.00
02E10604	13303015	Cake, cupcake or muffin, banana, prepared from dry mix, uniced	0.00
02E10436	13301002	Cake or cupcake, black forest (chocolate-cherry), layered, cream-filled, commercial	0.00
02E10597	13301003	Cake or cupcake, black forest (chocolate-cherry), layered, cream-filled, homemade from basic ingredients	0.00
02E10641	13303016	Cake or cupcake, berry, undefined fat, uniced	0.00
02E10469	13303017	Cake or cupcake, berry, reduced fat, commercial, uniced	0.00
02E10568	13303018	Cake, cupcake or muffin, berry, prepared from dry mix, undefined fat, uniced	0.00
02E10649	13306001	Cake or cupcake, berry shortcake, undefined fat	0.00
02E10528	13303019	Cake or cupcake, caramel, uniced	0.00
02E10605	13303020	Cake or cupcake, caramel, iced	0.00
02E10583	13303021	Cake or cupcake, carrot, commercial, uniced	0.00
02E10440	13303022	Cake or cupcake, carrot, commercial, iced	0.00
02E10584	13303023	Cake or cupcake, carrot, commercial, iced, filled with cream	0.00
02E10545	13303024	Cake or cupcake, carrot, homemade from basic ingredients, butter or dairy blend, uniced	0.00
02E10546	13303025	Cake or cupcake, carrot, homemade from basic ingredients, butter or dairy blend, iced	0.00
02E10462	13303026	Cake or cupcake, carrot, homemade from basic ingredients, oil, uniced	0.00
02E10582	13303027	Cake or cupcake, carrot, homemade from basic ingredients, oil, iced	0.00
02E10606	13303028	Cake or cupcake, carrot, homemade from basic ingredients, undefined fat, uniced	0.00
02E10607	13303029	Cake or cupcake, carrot, homemade from basic ingredients, undefined fat, iced	0.00
02E10459	19702001	Cake, cheesecake, biscuit base, chocolate or coffee flavoured cream cheese topping, commercial	0.00

02E10486	19702002	Cake, cheesecake, biscuit base, fruit flavoured cream cheese topping, commercial	0.00
02E10623	19702003	Cake, cheesecake, biscuit base, fruit flavoured cream cheese topping, homemade from basic ingredients	0.00
02E10458	19702004	Cake, cheesecake, biscuit base, plain cream cheese topping, commercial	0.00
02E10503	19702005	Cake, cheesecake, biscuit base, plain cream cheese topping, homemade from basic ingredients	0.00
02E10494	13301004	Cake or cupcake, chocolate, commercial, uniced	0.00
02E10498	13301005	Cake or cupcake, chocolate, commercial, uniced, filled with cream	0.00
02E10495	13301006	Cake or cupcake, chocolate, commercial, sugar based icing	0.00
02E10497	13301007	Cake or cupcake, chocolate, commercial, sugar based icing, filled with cream	0.00
02E10441	13301008	Cake or cupcake, chocolate, homemade from basic ingredients, butter or dairy blend, uniced	0.00
02E10502	13301009	Cake or cupcake, chocolate, homemade from basic ingredients, butter or dairy blend, sugar based icing	0.00
02E10548	13301010	Cake or cupcake, chocolate, homemade from basic ingredients, margarine spread, uniced	0.00
02E10549	13301011	Cake or cupcake, chocolate, homemade from basic ingredients, margarine spread, sugar based icing	0.00
02E10609	13301012	Cake or cupcake, chocolate, homemade from basic ingredients, oil, uniced	0.00
02E10610	13301013	Cake or cupcake, chocolate, homemade from basic ingredients, oil, sugar based icing	0.00
02E10539	13301014	Cake or cupcake, chocolate, homemade from basic ingredients, undefined fat, uniced	0.00
02E10541	13301015	Cake or cupcake, chocolate, homemade from basic ingredients, undefined fat, uniced, filled with cream	0.00
02E10611	13301016	Cake or cupcake, chocolate, homemade from basic ingredients, undefined fat, sugar based icing	0.00
02E10542	13301017	Cake or cupcake, chocolate, homemade from basic ingredients, undefined fat, sugar based icing, filled with cream	0.00
02E10481	13301018	Cake or cupcake, chocolate, homemade from basic ingredients, undefined fat, almond meal, uniced	0.00
02E10543	13301019	Cake, cupcake or muffin, chocolate, prepared from dry mix, undefined fat, uniced	0.00
02E10544	13301020	Cake, cupcake or muffin, chocolate, prepared from dry mix, undefined fat, sugar based icing	0.00
02E10526	13303030	Cake or cupcake, citrus, commercial, uniced	0.00
02E10612	13303031	Cake or cupcake, citrus, commercial, iced	0.00
02E10570	13303032	Cake or cupcake, citrus, homemade from basic ingredients, undefined fat, uniced	0.00
02E10574	13303033	Cake or cupcake, citrus, homemade from basic ingredients, undefined fat, uniced, filled with cream	0.00

02E10573	13303034	Cake or cupcake, citrus, homemade from basic ingredients, undefined fat, iced	0.00
02E10628	13303035	Cake or cupcake, coconut, commercial or homemade, uniced	0.00
02E10626	13301021	Cake or cupcake, coffee, homemade from basic ingredients, undefined fat, uniced	0.00
02E10627	13301022	Cake or cupcake, coffee, homemade from basic ingredients, undefined fat, iced	0.00
02E10516	13303036	Cake or cupcake, date loaf, commercial, uniced	0.00
02E10499	13303037	Cake or cupcake, date loaf, homemade from basic ingredients, undefined fat, uniced	0.00
02E10625	13303038	Cake or cupcake, date loaf, commercial or homemade, undefined fat, iced	0.00
02E10566	13303039	Cake, friand, commercial, uniced	0.00
02E10630	13303040	Cake, friand, homemade from basic ingredients, uniced	0.00
02E10443	13303041	Cake, fruit, dark in colour, commercial, uniced	0.00
02E10444	13303042	Cake, fruit, light in colour, commercial, uniced	0.00
02E10530	13303043	Cake, fruit, commercial, uniced	0.00
02E10631	13303045	Cake, fruit, commercial, iced	0.00
02E10524	13303044	Cake, fruit, commercial, reduced fat, uniced	0.00
02E10445	13303046	Cake, fruit, homemade from basic ingredients, butter or dairy blend, uniced	0.00
02E10550	13303047	Cake, fruit, homemade from basic ingredients, undefined fat, uniced	0.00
02E10632	13303048	Cake, fruit, homemade from basic ingredients, undefined fat, iced	0.00
02E10520	13301023	Cake or cupcake, hazelnut, homemade from basic ingredients, undefined fat, uniced	0.00
02E10489	13303049	Cake or cupcake, honey, commercial or homemade, uniced	0.00
02E10490	13303050	Cake or cupcake, honey, commercial or homemade, uniced, filled with cream	0.00
02E10634	13303051	Cake or cupcake, honey, commercial or homemade, iced	0.00
02E10448	13302016	Cake, lamington, unfilled	0.00
02E10642	13302017	Cake, lamington, filled with jam &/or cream	0.00
02C10144	13403001	Cake, moon, commercial	0.00
02E10523	13301024	Cake or cupcake, mud, caramel or white chocolate, uniced	0.00
02E10532	13301025	Cake or cupcake, mud, caramel or white chocolate, sugar based icing	0.00
02E10456	13301026	Cake or cupcake, mud, dark chocolate, commercial, chocolate ganache icing	0.00
02E10500	13301027	Cake or cupcake, mud, dark chocolate, homemade from basic ingredients, undefined fat, uniced	0.00
02E10464	13301028	Cake or cupcake, mud, dark chocolate, homemade from basic ingredients, undefined fat, chocolate ganache icing	0.00

02E10576	13303052	Cake or cupcake, orange & almond, commercial, uniced	0.00
02E10577	13303053	Cake or cupcake, orange & poppy seed, commercial or homemade, uniced	0.00
02E10579	13303054	Cake or cupcake, orange & poppy seed, commercial or homemade, iced	0.00
02E10511	13303055	Cake, cupcake or muffin, orange & poppy seed, prepared from dry mix, undefined fat, uniced	0.00
02E10515	13303056	Cake, cupcake or muffin, orange & poppy seed, prepared from dry mix, undefined fat, sugar based icing	0.00
02E10639	13303057	Cake, pineapple upside down cake, homemade from basic ingredients, fat not further defined	0.00
02E10438	13303058	Cake or cupcake, plain butter cake, commercial, uniced	0.00
02E10437	13303059	Cake or cupcake, plain butter cake, commercial, iced	0.00
02E10439	13303060	Cake or cupcake, plain butter cake, homemade from basic ingredients, butter or dairy blend, uniced	0.00
02E10508	13303061	Cake or cupcake, plain butter cake, homemade from basic ingredients, butter or dairy blend, iced	0.00
02E10614	13303062	Cake or cupcake, plain butter cake, homemade from basic ingredients, margarine, uniced	0.00
02E10651	13303063	Cake or cupcake, plain butter cake, homemade from basic ingredients, margarine, iced	0.00
02E10615	13303064	Cake or cupcake, plain butter cake, homemade from basic ingredients, oil, uniced	0.00
02E10580	13303065	Cake or cupcake, plain butter cake, homemade from basic ingredients, undefined fat, uniced	0.00
02E10581	13303066	Cake or cupcake, plain butter cake, homemade from basic ingredients, undefined fat, iced	0.00
02E10465	13303068	Cake, cupcake or muffin, plain butter cake, prepared from dry mix, undefined fat, uniced	0.00
02E10514	13303069	Cake, cupcake or muffin, plain butter cake, prepared from dry mix, undefined fat, iced	0.00
02E10613	13303067	Cake or cupcake, plain butter cake, commercial or homemade, iced, cream filling	0.00
02E10558	13301029	Cake, cupcake or muffin, vanilla with chocolate chip, prepared from dry mix, undefined fat, uniced	0.00
02E10635	13303070	Cake or cupcake, plum, homemade from basic ingredients, undefined fat, uniced	0.00
02E10617	13303071	Cake or cupcake, pumpkin, homemade from basic ingredients, undefined fat, uniced	0.00
02E10446	13302002	Cake or cupcake, sponge, plain, commercial, uniced, unfilled	0.00
02E10471	13302003	Cake or cupcake, sponge, plain, commercial, uniced, filled with cream	0.00
02E10492	13302004	Cake or cupcake, sponge, plain, commercial, uniced, filled with jam	0.00
02E10447	13302005	Cake or cupcake, sponge, plain, commercial, uniced, filled with jam & cream	0.00

02E10618	13302006	Cake or cupcake, sponge, plain, commercial, iced, unfilled	0.00
02E10619	13302007	Cake or cupcake, sponge, plain, commercial, iced, filled with cream	0.00
02E10533	13302008	Cake or cupcake, sponge, plain, homemade from basic ingredients, uniced	0.00
02E10621	13302009	Cake or cupcake, sponge, plain, homemade from basic ingredients, uniced, filled with jam &/or cream	0.00
02E10620	13302010	Cake or cupcake, sponge, plain, homemade from basic ingredients, iced, unfilled	0.00
02E10647	13302011	Cake or cupcake, sponge, plain, uniced, filled with fruit & cream	0.00
02E10473	13302012	Cake or cupcake, sponge, chocolate flavoured, commercial, uniced, filled with jam & cream	0.00
02E10616	13302013	Cake or cupcake, sponge, chocolate flavoured, commercial, iced, filled with cream	0.00
02E10463	13302014	Cake or cupcake, sponge, plain, prepared from dry mix, undefined fat, uniced, unfilled	0.00
02E10622	13302015	Cake or cupcake, sponge, plain, prepared from dry mix, undefined fat, iced	0.00
02E10636	13303072	Cake or cupcake, sultana, commercial or homemade from basic ingredients, uniced	0.00
02E10488	13303073	Cake, tea cake, commercial, uniced	0.00
02E10522	13303074	Cake, tea cake, apricot, homemade from basic ingredients, undefined fat, uniced	0.00
02E10461	13303075	Cake, tea cake, cinnamon topped, homemade from basic ingredients, undefined fat, uniced	0.00
02E10644	13303076	Cake, custard filled, with & without icing	0.00
02E10645	13303077	Cake, gluten free, with & without icing	0.00
02E30146	13605003	Doughnut, custard filled	0.00
02E30124	13605004	Doughnut, custard filled, with chocolate icing	0.00
02E30147	13605001	Doughnut, cream filled	0.00
02E30125	13605002	Doughnut, cream filled, with chocolate icing	0.00
02E30115	13605005	Doughnut, custard & cream filled, with chocolate icing	0.00
02E30119	13605006	Doughnut, jam filled, sugar coated	0.00
02E30148	13605007	Doughnut, jam filled, with chocolate icing	0.00
02E30117	13605008	Doughnut, unfilled, with cinnamon & sugar dusting	0.00
02E30116	13605009	Doughnut, unfilled, glazed	0.00
02E30121	13605011	Doughnut, unfilled, with icing (except chocolate flavoured)	0.00
02E30122	13605010	Doughnut, unfilled, with chocolate flavoured icing	0.00
02E10476	13304002	Muffin, cake-style, apple, commercial, uniced	0.00
02E10596	13304003	Muffin, cake-style, apple, homemade from basic ingredients, uniced	0.00
02E10527	13304004	Muffin, cake-style, apricot, homemade from basic ingredients, uniced	0.00
02E10567	13304005	Muffin, cake-style, banana, commercial, uniced	0.00
02E10590	13304006	Muffin, cake-style, banana, homemade from basic ingredients, uniced	0.00

02E10477	13304007	Muffin, cake-style, berry, commercial, uniced	0.00
02E10560	13304008	Muffin, cake-style, berry, homemade from basic ingredients, uniced	0.00
02E10551	13304009	Muffin, cake-style, bran, with or without dried fruit, commercial, uniced	0.00
02E10564	13304010	Muffin, cake-style, bran, with or without dried fruit, homemade from basic ingredients, uniced	0.00
02E10559	13304011	Muffin, cake-style, chocolate, homemade from basic ingredients, uniced	0.00
02E10474	13304012	Muffin, cake-style, chocolate chip, commercial, uniced	0.00
02E10531	13304013	Muffin, cake-style, chocolate chip, homemade from basic ingredients, uniced	0.00
02E10519	13304014	Muffin, cake-style, chocolate, with or without chocolate chip, commercial, uniced	0.00
02E10521	13304015	Muffin, cake-style, coconut & lemon, homemade from basic ingredients, uniced	0.00
02E10478	13304016	Muffin, cake-style, orange & poppy seed, commercial, uniced	0.00
02E10563	13304017	Muffin, cake-style, plain, commercial, uniced	0.00
02E10599	13304018	Muffin, cake-style, raspberry & white chocolate, commercial, uniced	0.00
02E10601	13304019	Muffin, cake-style, raspberry & white chocolate, homemade from basic ingredients, uniced	0.00
02E10638	13304020	Muffin, cake-style, commercial, not further defined	0.00
02E10648	13304021	Muffin, cake-style, savoury, homemade from basic ingredients	0.00
02E30144	13601001	Crepe or pancake, banana, homemade from basic ingredients	0.00
02E30126	13601002	Crepe or pancake, berry, homemade from basic ingredients	0.00
02E30127	13601003	Crepe or pancake, buckwheat flour, homemade from basic ingredients	0.00
02E30134	13601004	Crepe or pancake, buttermilk, homemade from basic ingredients	0.00
02E30142	13601005	Crepe or pancake, chocolate, homemade from basic ingredients	0.00
02E30141	13601006	Crepe or pancake, gluten free, commercial or homemade	0.00
02E30137	13601007	Crepe or pancake, plain, commercial	0.00
02E30112	13601008	Crepe or pancake, plain, dry mix	0.00
02E30114	13601009	Crepe or pancake, plain, prepared from dry mix using water	0.00
02E30111	13601010	Crepe or pancake, plain, white wheat flour, homemade from basic ingredients	0.00
02E30152	13601011	Crepe or pancake, potato, homemade from basic ingredients	0.00
02E30135	13601012	Crepe or pancake, rice based, homemade from basic ingredients	0.00
02E30151	13601013	Crepe or pancake, wholemeal wheat flour, commercial or homemade	0.00

02E30131	13401001	Pancake, Chinese	0.00
02E30120	13602001	Pikelet, plain, commercial	0.00
02E30110	13602002	Pikelet, plain, homemade from basic ingredients	0.00
02F40478	13601015	Crepe or pancake, with butter & syrup, fast food chain	0.00
02E30154	13601014	Crepe or pancake, with savoury filling	0.00
08F11083	18902006	Crepe or pancake, savoury, filled with duck & vegetables, with sauce (Peking duck)	0.00
02E50266	13309001	Pudding, bread & butter, homemade from basic ingredients, butter or dairy blend	0.00
02E50315	13305001	Pudding, chocolate, homemade from basic ingredients	0.00
02E50267	13309002	Pudding, lemon delicious, homemade from basic ingredients	0.00
02E10650	13309003	Pudding, made from cake or bread, with mixed berries & cream	0.00
02E10451	13305002	Pudding, plum, canned, commercial	0.00
02E10483	13305003	Pudding, plum, steamed or boiled, homemade from basic ingredients	0.00
02E10479	13305004	Pudding, self-saucing, chocolate flavoured, prepared from dry mix	0.00
02E10480	13305005	Pudding, self-saucing, other flavours, prepared from dry mix	0.00
02E50311	13305006	Pudding, sticky date, commercial	0.00
02E50252	13305007	Pudding, sticky date, homemade from basic ingredients	0.00
02E50316	13309004	Pudding, vanilla & other non-chocolate flavours, homemade from basic ingredients	0.00
02E20087	13307001	Rock cake, commercial or homemade	0.00
02E20094	13308001	Scone, cheese, commercial	0.00
02E20113	13308002	Scone, cheese, homemade from basic ingredients	0.00
02E20101	13308003	Scone, chocolate, with chocolate chips, commercial	0.00
02E20092	13307002	Scone, date, commercial	0.00
02E20095	13307003	Scone, date, homemade from basic ingredients	0.00
02E20091	13307004	Scone, plain, commercial	0.00
02E20097	13307005	Scone, plain, homemade from basic ingredients	0.00
02E20096	13307006	Scone, pumpkin, commercial	0.00
02E20088	13307007	Scone, pumpkin, homemade from basic ingredients	0.00
02E20098	13307008	Scone, sultana, commercial	0.00
02E20086	13307009	Scone, sultana, homemade from basic ingredients	0.00
02E20112	13307010	Scone, wholemeal, commercial	0.00
02E20111	13307011	Scone, wholemeal, homemade from basic ingredients	0.00
02E50320	13306008	Slice, caramel, commercial	0.00
02E50260	13306009	Slice, caramel, homemade from basic ingredients, fat not further defined	0.00
02E50261	13306010	Slice, cherry, homemade from basic ingredients, fat not further defined	0.00
02E50346	13306011	Slice, chocolate, commercial, chocolate icing	0.00
02E50312	13306012	Slice, chocolate, homemade from basic ingredients, fat not further defined, chocolate icing	0.00

02E50345	13306013	Slice, coconut, with jam, commercial	0.00
02E50264	13306014	Slice, coconut, with jam, homemade from basic ingredients, fat not further defined	0.00
02E50344	13306015	Slice, dried fruit, homemade from basic ingredients, fat not further defined	0.00
02E50244	13306016	Slice, fruit mince, commercial, with icing	0.00
02E50274	13306017	Slice, hedgehog, commercial, chocolate icing	0.00
02E50262	13306018	Slice, hedgehog, homemade from basic ingredients, fat not further defined, chocolate icing	0.00
02E50306	13306019	Slice, jelly, commercial	0.00
02E50307	13306020	Slice, jelly, homemade from basic ingredients, fat not further defined	0.00
02E50300	13306021	Slice, lemon, homemade from basic ingredients, fat not further defined	0.00
02E50308	13306022	Slice, meringue, with jam, homemade from basic ingredients, fat not further defined	0.00
02E50272	13306023	Slice, muesli, with sultanas & apricot, homemade from basic ingredients, fat not further defined	0.00
02E50348	13306024	Slice, nut, homemade from basic ingredients, fat not further defined	0.00
02E50271	13306025	Slice, oat, with caramel, marshmallow & chocolate, homemade from basic ingredients, fat not further defined	0.00
02E50347	13306026	Slice, passionfruit	0.00
02E50241	13306027	Slice, vanilla, commercial, with icing (except chocolate flavoured)	0.00
02E50310	13306028	Slice, vanilla, homemade from basic ingredients, fat not further defined, with icing (except chocolate flavoured)	0.00
02E50349	13306029	Slice, not further defined	0.00
03B10177	17202001	Souffle, chocolate	0.00
03B10178	17202002	Souffle, fruit	0.00
02E50265	19702006	Trifle, homemade from basic ingredients	0.00
02C20361	13603001	Waffle, plain, frozen, commercial	0.00
02C20362	13603002	Waffle, plain, fresh, homemade from basic ingredients	0.00
02C20386	13603003	Waffle, berry, fresh, homemade from basic ingredients	0.00
02E30113	13604008	Yorkshire pudding, homemade from basic ingredients	0.00
02A10430	12101003	BarleyMax, dry, uncooked	0.00
02A10342	12101001	Barley, pearl, uncooked	0.00
02A10343	12101002	Barley, pearl, cooked in water, no added fat or salt	0.00
02A10344	12101004	Buckwheat groats, uncooked	0.00
02A10470	12101005	Buckwheat groats, cooked in water, no added salt	0.00
02A10376	12101006	Bulgur (burghul, burgaul), dry, uncooked	0.00
02A10351	12101007	Bulgur (burghul, burgaul), soaked in water, no added fat or salt	0.00
02A10352	12101008	Bulgur (burghul, burgaul), cooked in water, no added fat or salt	0.00
02A10480	22101002	Seed, chia, cooked, no added salt	0.00
02A10345	12101009	Cornmeal (polenta), uncooked	0.00

02A10392	12101010	Cornmeal (polenta), cooked in water, no added salt	0.00
02A10482	12101011	Cornmeal (polenta), cooked in water & fat, no added salt	0.00
02A10346	12103001	Couscous, uncooked	0.00
02A10394	12103002	Couscous, cooked in water, no added fat or salt	0.00
02A10474	12103003	Couscous, cooked in water & fat, no added salt	0.00
02A10458	12101013	Grains & pasta, for homemade soup recipes	0.00
02A10469	12101012	Grains, boiled, for homemade mixed dishes	0.00
02A10347	12101014	Millet, uncooked	0.00
02A10348	12101015	Millet, boiled in water, no added fat or salt	0.00
02A10356	12101016	Oat bran, unprocessed, uncooked	0.00
02A10349	12101017	Oats, rolled, uncooked	0.00
02A10457	12101018	Oats, rolled, uncooked, added fibre & Ca	0.00
02A10454	12601017	Oats, rolled, mixed with sugar or honey & other flavours, uncooked	0.00
02A10401	12601018	Oats, rolled, mixed with sugar, flavours & dried fruit, uncooked	0.00
02A10431	22102001	Psyllium, dry, uncooked	0.00
02A10444	22102002	Psyllium, cooked in water, no added salt	0.00
02A10479	22102003	Psyllium, cooked in soy milk, no added salt	0.00
02A10405	12101019	Quinoa, uncooked	0.00
02A10445	12101021	Quinoa, cooked in water, no added salt	0.00
02A10477	12101022	Quinoa, cooked in water & fat, no added salt	0.00
02A10446	12101020	Quinoa, cooked in milk, no added salt	0.00
02A10357	12102001	Rice bran, extruded or low processed, uncooked	0.00
02A10499	12602003	Rice, ground brown rice, with added dry fruit, uncooked	0.00
02A20113	12101025	Rye, uncooked	0.00
02A10503	12101026	Rye, grains, cooked in water	0.00
02A10353	12103018	Semolina, uncooked	0.00
02A10448	12103020	Semolina, cooked in water, no added salt	0.00
02A10483	12103019	Semolina, cooked in cows milk, no added salt	0.00
02A10404	12101030	Spelt, uncooked	0.00
02A10449	12101027	Sago, dry, uncooked	0.00
02A10450	12101029	Sago, cooked in water, no added salt	0.00
02A10451	12101028	Sago, cooked in milk, no added salt	0.00
02A10350	12103021	Tapioca, pearl or seed style, uncooked	0.00
02A10375	12103022	Tapioca, seed or pearl style, boiled in water, no added fat or salt	0.00
02A10355	12101031	Wheat bran, unprocessed, uncooked	0.00
02A10354	12101032	Wheat germ	0.00
02A10368	12102002	Rice, white, uncooked	0.00
02A10386	12102003	Rice, white, boiled, no added salt	0.00
02A10408	12102004	Rice, white, steamed or rice cooker, no added salt	0.00
02A10490	12102005	Rice, white, boiled, steamed or microwaved with coconut milk	0.00

02F40255	13511008	Rice, white, fried, no meat or vegetables, oil not further defined, homemade or commercial	0.00
02A10406	12102006	Rice, white, purchased par-cooked or instant, cooked	0.00
02A10371	12102007	Rice, brown, uncooked	0.00
02A10388	12102008	Rice, brown, boiled, no added salt	0.00
02F40256	13511004	Rice, brown, fried, no meat or vegetables, oil not further defined, homemade	0.00
02A10407	12102009	Rice, brown, purchased par-cooked or instant, cooked	0.00
02F40303	12102010	Rice, flavoured, instant dry mix	0.00
02F40233	12102011	Rice, flavoured, prepared from dry mix	0.00
02A10373	12102012	Rice, purchased as parboiled (gold rice), uncooked	0.00
02A10387	12102013	Rice, purchased as parboiled (gold rice), boiled, no added salt	0.00
02A10489	12102014	Rice, red, steamed or rice cooker, no added salt	0.00
02A10378	12101023	Rice, wild, uncooked	0.00
02A10372	12101024	Rice, wild, boiled, no added salt	0.00
02A10414	12102015	Rice, cooked, for making sushi	0.00
02A30016	12103017	Rice paper wrapper, soaked in water	0.00
02F40293	13511001	Paella, white rice, with chicken, chorizo & mixed vegetables	0.00
02F40294	13511002	Paella, white rice, with chicken, chorizo, mixed vegetables & seafood	0.00
02F40295	13511003	Paella, white rice, with mixed vegetables & seafood	0.00
02F40282	13511005	Rice, brown, fried with bacon or ham, egg & mixed vegetables	0.00
02F40281	13511006	Rice, brown, fried with chicken, egg & mixed vegetables	0.00
02F40279	13511007	Rice, brown, fried with mixed vegetables	0.00
02F40283	13511009	Rice, white, fried with bacon or ham, egg & mixed vegetables	0.00
02F40284	13511010	Rice, white, fried with bacon or ham, egg, mixed vegetables & nuts	0.00
02F40222	13511011	Rice, white, fried with bacon or ham, egg, prawns & vegetables	0.00
02F40296	13511012	Rice, white, fried with beef, lamb or pork & mixed vegetables	0.00
02F40280	13511013	Rice, white, fried with chicken, egg & mixed vegetables	0.00
02F40259	13511014	Rice, white, fried with chicken & mixed vegetables	0.00
02F40260	13511015	Rice, white, fried with egg	0.00
02F40258	13511016	Rice, white, fried with egg & mixed vegetables	0.00
02F40418	13511017	Rice, white, fried with egg, mixed vegetables & seafood	0.00
02F40298	13511018	Rice, white, fried with lentils	0.00
02F40257	13511019	Rice, white, fried with mixed vegetables	0.00
02F40419	13511020	Rice, white, fried with mixed vegetables & seafood	0.00
02F40248	13511021	Rice, white, pilaf style, with butter, stock & spices	0.00
02F40254	13511022	Risotto, bacon or ham & mixed vegetables, with cheese	0.00

02F40299	13511023	Risotto, beef, lamb or pork & mixed vegetable, with cheese	0.00
02F40289	13511024	Risotto, chicken & mixed vegetables, with cheese	0.00
02F40288	13511025	Risotto, mixed vegetables, with cheese	0.00
02F40251	13511026	Risotto, mushroom, with cheese	0.00
02F40250	13511027	Risotto, plain, with cheese	0.00
02F40291	13511028	Risotto, prawn & mixed vegetables, with cheese	0.00
02F40285	13511029	Risotto, pumpkin, with cheese	0.00
02A20100	12103005	Flour, arrowroot	0.00
02A20107	25201001	Flour, chick pea (besan)	0.00
02A20108	12103006	Flour, cornflour, from maize starch	0.00
02A20099	12103007	Flour, cornflour, from maize & wheat starch	0.00
02A20112	12103008	Flour, gluten free mix (rice, soy, corn & tapioca)	0.00
02A20106	12103009	Flour, rice	0.00
02A20098	12103010	Flour, rye, wholemeal	0.00
13A20139	25201002	Flour, soya	0.00
02A20110	12103011	Flour, spelt	0.00
02A20103	12103012	Flour, wheat, white, plain	0.00
02A20114	12104003	Flour, wheat, white, plain, added vitamins B1, B2, B3, B6, E & folate & Fe, Mg & Zn (Jackaroo)	0.00
02A20102	12103013	Flour, wheat, white, self-raising	0.00
02A20115	12104004	Flour, wheat, white, self-raising, added vitamins B1, B2, B3, B6, E & folate & Fe, Mg & Zn (Jackaroo)	0.00
02A20101	12104001	Flour, wheat, white, high protein or bread making flour	0.00
02A20109	12104002	Flour, wheat, white, self-raising, added calcium & folate	0.00
02A20104	12103014	Flour, wheat, wholemeal, plain	0.00
02A20105	12103015	Flour, wheat, wholemeal, self-raising	0.00
10F60098	12103016	Gluten, from wheat (vital wheat gluten)	0.00
10F60099	24102001	Starch, potato	0.00
02F30277	13505001	Bacon burger, bread roll, bacon rasher, with cheese, takeaway & homemade	0.00
02F30323	13505002	Bacon burger, bread roll, bacon rasher, with cheese & salad, takeaway & homemade	0.00
02F30335	13505060	Bacon burger, bread roll, bacon rasher, with egg, takeaway & homemade	0.00
02F30279	13505003	Bacon burger, bread roll, bacon rasher, with salad, takeaway & homemade	0.00
02F40469	13507001	Breakfast wrap, white tortilla, bacon, egg , cheese & sauce, fast food chain	0.00
02F30292	13505004	Chicken burger, white roll, chicken breast, with bacon, cheese, egg & sauce, fast food chain-style	0.00
02F30218	13505005	Chicken burger, white roll, chicken breast, with bacon, cheese, lettuce & mayonnaise, fast food chain	0.00
02F30197	13505006	Chicken burger, white roll, chicken breast, with cheese, lettuce & mayonnaise, fast food chain	0.00

02F30234	13505008	Chicken burger, white roll, double chicken breast, with cheese, lettuce, chilli sauce & mayonnaise, fast food chain	0.00
02F30227	13505007	Chicken burger, white roll, chicken breast, with lettuce, tomato & mayonnaise, fast food chain	0.00
02F30235	13505009	Chicken burger, white roll, crumbed chicken breast, with bacon, cheese, lettuce, mayonnaise & BBQ sauce, fast food chain	0.00
02F30293	13505010	Chicken burger, white roll, crumbed chicken breast, with bacon, cheese, lettuce, pineapple & mayonnaise, fast food	0.00
02F30226	13505011	Chicken burger, white roll, crumbed chicken breast, with bacon, cheese, lettuce, tomato & mayonnaise, fast food chain	0.00
02F30224	13505012	Chicken burger, white roll, crumbed chicken breast, with cheese & mayonnaise, fast food chain	0.00
02F30254	13505013	Chicken burger, white roll, crumbed chicken breast, with cheese, lettuce, hash brown, mayonnaise & sauce, fast food	0.00
02F30196	13505014	Chicken burger, white roll, crumbed chicken breast, with cheese, lettuce, onion, tomato, relish & mayonnaise, fast food chain	0.00
02F30188	13505015	Chicken burger, white roll, crumbed chicken breast, with lettuce & mayonnaise, fast food chain	0.00
02F30205	13505021	Chicken roll, white roll, chicken, with mayonnaise, fast food chain	0.00
02F40272	13507002	Chicken wrap, white tortilla, coated chicken breast strips, with salad & mayonnaise, fast food chain	0.00
02F40276	13507003	Chicken wrap, white tortilla, chicken breast strips, with salad & mayonnaise, fast food chain	0.00
02F30271	13505016	Chicken burger, bread roll, chicken fillet or patty, takeaway & homemade	0.00
02F30202	13505017	Chicken burger, bread roll, chicken fillet or patty & salad, takeaway & homemade	0.00
02F30321	13505018	Chicken burger, bread roll, chicken fillet or patty, with bacon & salad, takeaway & homemade	0.00
02F30272	13505019	Chicken burger, bread roll, chicken fillet or patty & cheese, takeaway & homemade	0.00
02F30200	13505020	Chicken burger, bread roll, chicken fillet or patty, with cheese & salad, takeaway & homemade	0.00
02F30329	13503001	Filled bread roll, Asian-style, pork, mayonnaise, pate & vegetables	0.00
02F30301	13503002	Filled bread roll, commercial, bacon & egg, no sauce	0.00
02F30305	13503003	Filled bread roll, commercial, bacon & egg, water based sauce	0.00
02F30307	13503004	Filled bread roll, commercial, bacon & egg, cheese, water based sauce	0.00
02B10769	13503005	Filled bread roll, commercial, beef, no sauce	0.00
02B10772	13503006	Filled bread roll, commercial, beef, water based sauce	0.00

02B10773	13503007	Filled bread roll, commercial, beef, cheese, no sauce	0.00
02B10774	13503008	Filled bread roll, commercial, beef, cheese, oil based sauce	0.00
02B10764	13503009	Filled bread roll, commercial, beef, cheese & water based sauce	0.00
02B10779	13503010	Filled bread roll, commercial, chicken & no sauce	0.00
02B10778	13503011	Filled bread roll, commercial, chicken & oil based sauce	0.00
02B10771	13503012	Filled bread roll, commercial, chicken & water based sauce	0.00
02B10770	13503013	Filled bread roll, commercial, chicken, cheese & no sauce	0.00
02B10768	13503014	Filled bread roll, commercial, chicken, cheese & oil based sauce	0.00
02B10766	13503015	Filled bread roll, commercial, chicken, cheese & water based sauce	0.00
02F30312	13503016	Filled bread roll, commercial, crumbed chicken, no sauce	0.00
02F30310	13503017	Filled bread roll, commercial, crumbed chicken, cheese & no sauce	0.00
02F30311	13503018	Filled bread roll, commercial, crumbed chicken, cheese & oil based sauce	0.00
02F30294	13503019	Filled bread roll, commercial, crumbed chicken, cheese & water based sauce	0.00
02B10775	13503020	Filled bread roll, commercial, ham & salami, cheese, oil based sauce	0.00
02B10776	13503021	Filled bread roll, commercial, ham & salami, cheese, water based sauce	0.00
02B10777	13503022	Filled bread roll, commercial, ham, oil based sauce	0.00
02B10765	13503023	Filled bread roll, commercial, ham, cheese, no sauce	0.00
02F30295	13503024	Filled bread roll, commercial, ham, cheese, oil based sauce	0.00
02F30297	13503025	Filled bread roll, commercial, ham, cheese, water based sauce	0.00
02F30296	13503026	Filled bread roll, commercial, meatball & no sauce	0.00
02B10767	13503027	Filled bread roll, commercial, meatball, cheese, no sauce	0.00
02F30298	13503028	Filled bread roll, commercial, meatball, cheese & oil based sauce	0.00
02F30299	13503029	Filled bread roll, commercial, meatball, cheese & water based sauce	0.00
02F30300	13503030	Filled bread roll, commercial, tuna & water based sauce	0.00
02F30302	13503031	Filled bread roll, commercial, tuna, cheese & no sauce	0.00
02F30303	13503032	Filled bread roll, commercial, tuna, cheese & oil based sauce	0.00
02F30304	13503033	Filled bread roll, commercial, vegetable & water based sauce	0.00
02F30306	13503034	Filled bread roll, commercial, vegetable, cheese & no sauce	0.00

02F30308	13503035	Filled bread roll, commercial, vegetable, cheese & oil based sauce	0.00
02F30309	13503036	Filled bread roll, commercial, vegetable, cheese & water based sauce	0.00
02F30189	13505022	Fish burger, with cheese, fast food chain	0.00
02F30333	13505061	Fish burger, bread roll, with salad & mayonnaise, takeaway & homemade	0.00
02F30332	18703010	Hamburger, no roll, beef patty, with cheese, fast food chain	0.00
02F30330	18703011	Hamburger, no roll, beef patty, with salad, takeaway & homemade	0.00
02F30331	20701002	Hamburger, no roll, vegetable & lentil patty, with salad, takeaway & homemade	0.00
02F30216	13505023	Hamburger, white roll, beef patty, with bacon, beetroot, cheese, egg, lettuce, onion & tomato & tomato sauce, fast food chain	0.00
02F30223	13506001	Hamburger, white roll, beef patty, with bacon, cheese, onion, pickles & sauce, fast food chain	0.00
02F30253	13505024	Hamburger, white roll, beef patty, with bacon, cheese, lettuce, onion rings, tomato, pickles, mayonnaise & sauce, fast food chain	0.00
02F30222	13506003	Hamburger, white roll, 2 beef patties, with bacon, cheese, lettuce, onion, pickles, tomato, mayonnaise & sauce, fast food chain	0.00
02F30211	13506004	Hamburger, white roll, 2 beef patties, with bacon, cheese, lettuce, tomato & mayonnaise, fast food style	0.00
02F30263	13505025	Hamburger, white roll, beef patty, with beetroot, cheese, lettuce, onion, tomato & tomato sauce, fast food chain	0.00
02F30194	13505026	Hamburger, white roll, beef patty, with cheese, lettuce, onion, pickles, tomato, mayonnaise & tomato sauce, fast food chain	0.00
02F30181	13505027	Hamburger, white roll, beef patty, with cheese, lettuce, sauce, fast food chain	0.00
02F30185	13506002	Hamburger, white roll, beef patty, with cheese, onion, pickles & sauce, fast food chain	0.00
02F30225	13506005	Hamburger, white roll, 2 beef patties, with cheese, onion, pickles & sauce, fast food chain	0.00
02F30190	13506006	Hamburger, white roll, beef patty, with cheese, sauce, fast food chain	0.00
02F30193	13505028	Hamburger, white roll, beef patty, with lettuce, onion, pickles & tomato, mayonnaise & sauce, fast food chain	0.00
02F30221	13505030	Hamburger, white roll, 2 beef patties, with lettuce, onion, pickles, tomato, mayonnaise & sauce, fast food chain	0.00
02F30195	13505029	Hamburger, white roll, beef patty, with pickles, tomato sauce & mustard, fast food chain	0.00
02F30264	13505031	Hamburger, bread roll, beef patty, takeaway & homemade	0.00

02F30268	13505034	Hamburger, bread roll, beef patty, with bacon & cheese, takeaway & homemade	0.00
02F30278	13505035	Hamburger, bread roll, beef patty, with bacon, cheese & salad, takeaway & homemade	0.00
02F30267	13505036	Hamburger, bread roll, beef patty, with bacon & salad, takeaway & homemade	0.00
02F30265	13505037	Hamburger, bread roll, beef patty, with cheese, takeaway & homemade	0.00
02F30220	13505038	Hamburger, bread roll, beef patty, with cheese & salad, takeaway & homemade	0.00
02F30184	13505039	Hamburger, bread roll, beef patty, with egg & salad, takeaway & homemade	0.00
02F30179	13505040	Hamburger, bread roll, beef patty, with salad, takeaway & homemade	0.00
02F30326	13505041	Hamburger, bread roll, goat fillet or patty, with salad, takeaway & homemade	0.00
02F30280	13505042	Hamburger, bread roll, lamb fillet or patty, takeaway & homemade	0.00
02F30325	13505043	Hamburger, bread roll, lamb fillet or patty, with cheese, takeaway & homemade	0.00
02F30320	13505044	Hamburger, bread roll, lamb fillet or patty, with cheese & salad, takeaway & homemade	0.00
02F30281	13505045	Hamburger, bread roll, lamb fillet or patty, with salad, takeaway & homemade	0.00
02F30328	13505046	Hamburger, bread roll, processed meat, with onion, takeaway & homemade	0.00
02F30239	13503037	Hot dog, bread roll, frankfurt filling	0.00
02F30262	13503038	Hot dog, bread roll, frankfurt & onion filling	0.00
02F30261	13503039	Hot dog, bread roll, frankfurt, onion & sauce filling	0.00
02F30231	13503040	Hot dog, bread roll, frankfurt & sauce filling	0.00
02F30245	13504001	Hot dog, bread roll, frankfurt, cheese & sauce filling	0.00
02F30252	13504002	Hot dog, bread roll, frankfurt, cheese, onion & sauce filling	0.00
02F30250	13503041	Hot dog, bread roll, frankfurt, onion, meat & sauce filling	0.00
02F30334	13503069	Hot dog, bread roll, vegetarian style frankfurt filling	0.00
02F40308	13503042	Kebab wrap, beef, with salad, takeaway	0.00
02F40353	13503043	Kebab wrap, beef, with salad & sauce, takeaway	0.00
02F40354	13503044	Kebab wrap, beef, with salad, cheese & sauce, takeaway	0.00
02F40307	13503045	Kebab wrap, chicken, with salad, takeaway	0.00
02F40351	13503046	Kebab wrap, chicken, with salad & sauce, takeaway	0.00
02F40352	13503047	Kebab wrap, chicken, with salad, cheese & sauce, takeaway	0.00
02F40468	13503048	Kebab wrap, falafel, with salad & sauce, takeaway	0.00
02F40309	13503049	Kebab wrap, lamb, with salad, takeaway	0.00
02F40348	13503050	Kebab wrap, lamb, with salad & sauce, takeaway	0.00
02F40355	13503051	Kebab wrap, lamb, with salad & sauce, homemade from basic ingredients	0.00

02F40350	13503052	Kebab wrap, lamb, with salad, cheese & sauce, takeaway	0.00
02F40332	13508001	Mexican nachos, corn chips, beans, with cheese & salsa	0.00
02F40328	13508003	Mexican nachos, corn chips, beef, with cheese & salsa	0.00
02F40330	13508005	Mexican nachos, corn chips, beef & bean, with cheese & salsa	0.00
02F40334	13508008	Mexican nachos, corn chips, with cheese & salsa	0.00
02F40429	13508009	Mexican nachos, corn chips, with cheese, salad & salsa	0.00
02F40464	13507005	Mexican nachos, corn chips, with salsa	0.00
02F40430	13507006	Mexican taco, hard shell, bean, with cheese & salsa	0.00
02F40327	13507007	Mexican taco, hard shell, bean, with cheese, salad & salsa	0.00
02F40422	13508010	Mexican taco, hard shell, beef, with cheese & salsa	0.00
02F40323	13507008	Mexican taco, hard shell, beef, with cheese, salad & salsa	0.00
02F40466	13507009	Mexican taco, hard shell, beef, with salad & salsa	0.00
02F40431	13508011	Mexican taco, hard shell, beef & bean, with cheese & salsa	0.00
02F40324	13507010	Mexican taco, hard shell, beef & bean, with cheese, salad & salsa	0.00
02F40325	13507011	Mexican taco, hard shell, chicken, with cheese, salad & salsa	0.00
02F40467	13507012	Mexican taco, hard shell, chicken, with salad & salsa	0.00
02F40326	13507013	Mexican taco, hard shell, chicken & bean, with cheese, salad & salsa	0.00
02F40317	13507015	Mexican wrap, bean, with cheese & salsa	0.00
02F40318	13507016	Mexican wrap, bean, with cheese, salad & salsa	0.00
02F40458	13507017	Mexican wrap, bean, with salad & salsa	0.00
02F40311	13507018	Mexican wrap, beef & bean, with cheese, salad & salsa	0.00
02F40305	13507019	Mexican wrap, beef, with cheese & salsa	0.00
02F40310	13507020	Mexican wrap, beef, with cheese, salad & salsa	0.00
02F40457	13507023	Mexican wrap, beef, with salad & salsa	0.00
02F40320	13507024	Mexican wrap, beef & bean, with cheese & salsa	0.00
02F40459	13507026	Mexican wrap, beef & bean, with salad & salsa	0.00
02F40428	13508012	Mexican wrap, with cheese & salsa	0.00
02F40314	13507027	Mexican wrap, chicken, with cheese & salsa	0.00
02F40316	13507028	Mexican wrap, chicken, with cheese, salad & salsa	0.00
02F40315	13507031	Mexican wrap, chicken, with salad & salsa	0.00
02F40312	13507032	Mexican wrap, chicken & bean, with cheese & salsa	0.00
02F40313	13507033	Mexican wrap, chicken & bean, with cheese, salad & salsa	0.00
02F40460	13507034	Mexican wrap, chicken & bean, with salad & salsa	0.00
02F30192	13505047	Muffin, English style, with bacon, cheese & egg, fast food chain	0.00
02F30238	13505048	Muffin, English style, with bacon & beef sausage patty, fast food chain	0.00

02F30191	13505049	Muffin, English style, with beef sausage patty, cheese & egg, fast food chain	0.00
02F10175	13501001	Pizza, bacon & egg, thick base, BBQ sauce, fast food chain	0.00
02F10174	13501002	Pizza, bacon & egg, thin base, BBQ sauce, fast food chain	0.00
02F10136	13501003	Pizza, cheese & pesto, purchased frozen, uncooked	0.00
02F10145	13502003	Pizza, cheese & tomato, purchased frozen, baked	0.00
02F10146	13502004	Pizza, cheese & tomato, thick base, takeaway style or homemade	0.00
02F10147	13502005	Pizza, cheese & tomato, thin base, takeaway style	0.00
02F10148	13502006	Pizza, cheese & tomato, thick base, fast food chain	0.00
02F10150	13502007	Pizza, cheese & tomato, thin base, fast food chain	0.00
02F10196	13502020	Pizza, cheese & tomato, flat bread base, homemade	0.00
02F10168	13501004	Pizza, chicken & bacon, BBQ sauce, purchased frozen, baked	0.00
02F10166	13501005	Pizza, chicken & bacon, thick base, BBQ sauce, takeaway style or homemade	0.00
02F10167	13501006	Pizza, chicken & bacon, thin base, BBQ sauce, takeaway style	0.00
02F10165	13501007	Pizza, chicken & bacon, thick base, BBQ sauce, fast food chain	0.00
02F10164	13501008	Pizza, chicken & bacon, thin base, BBQ sauce, fast food chain	0.00
02F10188	13501009	Pizza, chicken & vegetable, thick base, takeaway style & homemade	0.00
02F10189	13502014	Pizza, chicken & vegetable, thin base, takeaway style	0.00
02F10192	13502017	Pizza, chicken & vegetable, thin base, fast food chain	0.00
02F10198	13502021	Pizza, chicken & vegetable, flat bread base, homemade	0.00
02F10201	13501010	Pizza, ham & cheese, frozen, baked	0.00
02F10185	13501011	Pizza, ham & cheese, thick base, takeaway style & homemade	0.00
02F10186	13502013	Pizza, ham & cheese, thin base, takeaway style	0.00
02F10191	13502016	Pizza, ham & cheese, thin base, fast food chain	0.00
02F10199	13501012	Pizza, ham & cheese, flat bread base, homemade	0.00
02F10132	13501013	Pizza, ham & pineapple, purchased frozen, baked	0.00
02F10143	13501014	Pizza, ham & pineapple, thick base, takeaway style & homemade	0.00
02F10144	13501015	Pizza, ham & pineapple, thin base, takeaway style	0.00
02F10128	13501016	Pizza, ham & pineapple, thick base, fast food chain	0.00
02F10127	13501017	Pizza, ham & pineapple, thin base, fast food chain	0.00
02F10204	13501018	Pizza, lamb, thick base, takeaway style & homemade	0.00
02F10160	13501019	Pizza, meat lovers, BBQ sauce, purchased frozen, baked	0.00
02F10163	13501020	Pizza, meat lovers, thick base, takeaway style & homemade	0.00
02F10161	13502012	Pizza, meat lovers, thin base, takeaway style	0.00
02F10159	13501021	Pizza, meat lovers, thick base, BBQ sauce, fast food chain	0.00

02F10158	13502011	Pizza, meat lovers, thin base, fast food chain	0.00
02F10155	13502009	Pizza, pepperoni, purchased frozen, baked	0.00
02F10154	13501022	Pizza, pepperoni, thick base, takeaway style & homemade	0.00
02F10153	13502008	Pizza, pepperoni, thin base, takeaway style	0.00
02F10156	13501023	Pizza, pepperoni, thick base, fast food chain	0.00
02F10157	13502010	Pizza, pepperoni, thin base, fast food chain	0.00
02F10200	13502022	Pizza, pepperoni, flat bread base, homemade	0.00
02F10187	13501024	Pizza, prawn & vegetable, thick base, takeaway style & homemade	0.00
02F10190	13502015	Pizza, prawn & vegetable, thin base, takeaway style	0.00
02F10203	13501025	Pizza, prosciutto, thick base, takeaway style & homemade	0.00
02F10173	13501026	Pizza, seafood, thin base, purchased frozen, baked	0.00
02F10171	13501027	Pizza, seafood, thick base, takeaway style & homemade	0.00
02F10172	13501028	Pizza, seafood, thin base, takeaway style	0.00
02F10170	13501029	Pizza, seafood, thick base, fast food chain	0.00
02F10169	13501030	Pizza, seafood, thin base, fast food chain	0.00
02F10131	13502001	Pizza, supreme, purchased frozen, baked	0.00
02F10141	13501031	Pizza, supreme, thick base, takeaway style & homemade	0.00
02F10142	13502002	Pizza, supreme, thin base, takeaway style	0.00
02F10126	13501032	Pizza, supreme, thick base, fast food chain	0.00
02F10125	13501033	Pizza, supreme, thin base, fast food chain	0.00
02F10194	13502018	Pizza, supreme, stuffed crust, fast food chain	0.00
02F10195	13502019	Pizza, supreme, flat bread base, homemade	0.00
02B10728	13501034	Pizza, tomato sauce, thick base, takeaway style or homemade	0.00
02F10151	13501035	Pizza, vegetable, purchased frozen, baked	0.00
02F10139	13501036	Pizza, vegetable, thick base, takeaway style & homemade	0.00
02F10140	13501037	Pizza, vegetable, thin base, takeaway style	0.00
02F10137	13501038	Pizza, vegetable, thick base, fast food chain	0.00
02F10138	13501039	Pizza, vegetable, thin base, fast food chain	0.00
02F10197	13501040	Pizza, vegetable, flat bread base, homemade	0.00
02F10184	13501041	Pizza, vegetable, gluten free base, takeaway style & homemade	0.00
02F20070	13503070	Sandwich or roll, filled with canned spaghetti, toasted	0.00
02F20050	13504003	Sandwich or roll, filled with cheese	0.00
02F20051	13504004	Sandwich or roll, filled with cheese, toasted	0.00
02F20053	13503053	Sandwich or roll, filled with chicken	0.00
02F20054	13503054	Sandwich or roll, filled with chicken & salad	0.00
02F20055	13503055	Sandwich or roll, filled with egg	0.00
02F20057	13503056	Sandwich or roll, filled with ham	0.00
02F20058	13504005	Sandwich or roll, filled with ham & cheese	0.00
02F20046	13503057	Sandwich or roll, filled with ham & cheese, toasted	0.00
02F20059	13503058	Sandwich or roll, filled with ham & salad	0.00

02F20060	13503059	Sandwich or roll, filled with ham & tomato, toasted	0.00
02F20052	13503060	Sandwich or roll, filled with peanut butter	0.00
02F20047	13503061	Sandwich or roll, filled with roast beef or lamb	0.00
02F20049	13503062	Sandwich or roll, filled with roast beef or lamb & salad	0.00
02F20062	13503063	Sandwich or roll, filled with salad	0.00
02F20056	13503064	Sandwich or roll, filled with tuna	0.00
02F20066	13503065	Sandwich or roll, filled with tuna & salad	0.00
02F20048	13503066	Sandwich or roll, filled with vegemite	0.00
02F20061	13504006	Sandwich or roll, filled with vegemite & cheese	0.00
02F20063	13503067	Sandwich or roll, not further defined	0.00
02F20065	13504007	Sandwich or roll, not further defined, toasted	0.00
02F20064	13503068	Wrap, filled, not further defined	0.00
02F30316	13505050	Steak sandwich, bread roll, beef steak, takeaway & homemade	0.00
02F30318	13505051	Steak sandwich, bread roll, beef steak, with cheese, takeaway & homemade	0.00
02F30317	13505052	Steak sandwich, bread roll, beef steak, with cheese & salad, takeaway & homemade	0.00
02F30319	13505053	Steak sandwich, bread roll, beef steak, with salad, takeaway & homemade	0.00
02F30228	13505054	Vegetable burger, bread roll, vegetable patty, with cheese, lettuce, onion, sauce & mayonnaise, fast food chain	0.00
02F30290	13505055	Vegetable or lentil burger, bread roll, vegetable patty, takeaway & homemade	0.00
02F30291	13505056	Vegetable or lentil burger, bread roll, vegetable patty, with cheese, takeaway & homemade	0.00
02F30324	13505057	Vegetable or lentil burger, bread roll, vegetable patty, with cheese & salad, takeaway & homemade	0.00
02F30230	13505058	Vegetable or lentil burger, bread roll, vegetable patty, with salad, takeaway & homemade	0.00
02A30018	12403001	Noodle, buckwheat or soba, dry	0.00
02A30019	12403002	Noodle, buckwheat or soba, boiled, drained	0.00
02A10390	12401001	Noodle, wheat, Asian style, cooked	0.00
02A10418	12401002	Noodle, wheat with egg, plain, dry	0.00
02A10420	12401003	Noodle, wheat with egg, plain, boiled, no added fat	0.00
02A10436	12402004	Noodle, wheat, instant, flavoured, dry, uncooked	0.00
02A10366	12402005	Noodle, wheat, instant, flavoured, boiled, drained	0.00
02A10421	12402006	Noodle, wheat, instant, flavoured, boiled, undrained	0.00
02A10437	12402001	Noodle, wheat, instant, unflavoured, dry, uncooked	0.00
02A10422	12402002	Noodle, wheat, instant, unflavoured, boiled, drained	0.00
02A10423	12402003	Noodle, wheat, instant, unflavoured, boiled, undrained	0.00
02A10427	12402007	Noodle, wheat, instant, flavoured, fried or stir-fried, with or without fat	0.00
02A10424	12402010	Noodle, wheat, instant, low fat, flavoured, boiled, drained	0.00

02A10438	12402011	Noodle, wheat, instant, low fat, flavoured, boiled, undrained	0.00
02A10425	12402008	Noodle, wheat, instant, low fat, unflavoured, boiled, drained	0.00
02A10439	12402009	Noodle, wheat, instant, low fat, unflavoured, boiled, undrained	0.00
02A10496	12402012	Noodle, wheat, instant, not further defined	0.00
02A10377	12403003	Noodle, rice stick, boiled, drained	0.00
02A10410	12401004	Noodle, cooked, not further defined	0.00
02A30017	12401021	Noodles & pasta, boiled, for use in mixed dishes	0.00
02A10396	12403004	Pasta, gluten free, plain, boiled from dry, no added salt	0.00
02A10397	12403005	Pasta, gluten free, plain, boiled from dry, with added salt	0.00
02A10358	12401005	Pasta, white wheat flour, plain, dry	0.00
02A10382	12401006	Pasta, white wheat flour, plain, boiled from dry, no added salt	0.00
02A10359	12401007	Pasta, white wheat flour, plain, boiled from dry, with added salt	0.00
02A10379	12401008	Pasta, white wheat flour, plain, fresh, uncooked	0.00
02A10381	12401009	Pasta, white wheat flour, plain, fresh, boiled, no added salt	0.00
02A10492	12401010	Pasta, white wheat flour, plain, homemade, boiled, no added salt	0.00
02A10493	12401011	Pasta, white wheat flour, plain, homemade, boiled, with added salt	0.00
02A10364	12401012	Pasta, white wheat flour & egg, plain, dry	0.00
02A10385	12401013	Pasta, white wheat flour & egg, plain, boiled, no added salt	0.00
02A10365	12401014	Pasta, white wheat flour & egg, plain, boiled, with added salt	0.00
02A10380	12403006	Pasta, maize flour (corn) based, plain, dry	0.00
02A10363	12403007	Pasta, maize flour (corn) based, plain, cooked	0.00
02A10374	12401015	Pasta, white wheat flour & spinach, plain, dry	0.00
02A10384	12401016	Pasta, white wheat flour & spinach, plain, boiled, no added salt	0.00
02A10362	12401017	Pasta, white wheat flour & spinach, plain, boiled, with added salt	0.00
02A10360	12401018	Pasta, wholemeal wheat flour, plain, dry	0.00
02A10383	12401019	Pasta, wholemeal wheat flour, plain, boiled from dry, no added salt	0.00
02A10361	12401020	Pasta, wholemeal wheat flour, plain, boiled from dry, with added salt	0.00
02F40219	12404001	Pasta, filled with meat, fresh, commercial, boiled, without added sauce	0.00
02F40336	12404002	Pasta, filled with spinach & ricotta, fresh, commercial, boiled, without added sauce	0.00
02F40335	12404003	Pasta, filled with spinach & ricotta, fresh, homemade, boiled, without added sauce	0.00

02F40220	12404004	Pasta, filled with vegetables, fresh, commercial, boiled, without added sauce	0.00
02A10494	12401022	Pasta, unfilled, for commercial pasta recipes	0.00
02A10495	12401023	Pasta, unfilled, for homemade pasta recipes	0.00
02F40245	13509001	Cannelloni, homemade, filled with spinach & ricotta, with commercial based tomato sauce	0.00
02F40337	13509002	Cannelloni, homemade, filled with spinach & ricotta, with homemade tomato based sauce	0.00
02F40338	13509003	Cannelloni, homemade, filled with spinach & ricotta, with homemade tomato based sauce, added meat	0.00
02A10442	13513003	Gnocchi, homemade, potato gnocchi, commercial beef bolognese sauce	0.00
02A10441	13513004	Gnocchi, homemade, potato gnocchi, commercial dairy based sauce	0.00
02A10443	13513005	Gnocchi, homemade, potato gnocchi, homemade beef bolognese sauce	0.00
02A10501	13513002	Gnocchi, commercial or homemade, potato gnocchi, tomato based sauce	0.00
02F40470	13509007	Lasagne (Lasagna), beef, no added vegetables, homemade	0.00
02F40247	13509008	Lasagne (Lasagna), beef, with added vegetables, homemade	0.00
02F40214	13509006	Lasagne (Lasagna), beef, commercial, purchased fresh or frozen, cooked	0.00
02F40304	13509009	Lasagne (Lasagna), chicken, with added vegetables, cheese, homemade	0.00
02F40477	13509083	Lasagne (Lasagna), kangaroo, with added vegetables, homemade	0.00
02F40274	13509010	Lasagne (Lasagna), vegetable, homemade	0.00
02F40246	13509011	Macaroni & cheese, homemade, cooked unfilled pasta, homemade cheese sauce	0.00
02F40360	13509013	Pasta dish, commercial, cooked unfilled pasta, beef bolognese sauce	0.00
02F40361	13509014	Pasta dish, commercial, cooked unfilled pasta, beef bolognese sauce & added vegetables	0.00
02F40267	13509015	Pasta dish, homemade, cooked unfilled pasta, commercial beef bolognese sauce	0.00
02F40356	13509016	Pasta dish, homemade, cooked unfilled pasta, commercial beef bolognese sauce & added vegetables	0.00
02F40358	13509017	Pasta dish, homemade, cooked unfilled pasta, homemade beef bolognese sauce	0.00
02F40266	13509018	Pasta dish, homemade, cooked unfilled pasta, homemade beef bolognese sauce & added vegetables	0.00
02F40385	13510001	Pasta dish, commercial, cooked unfilled pasta, carbonara sauce, added bacon, with or without added vegetables	0.00
02F40386	13509019	Pasta dish, commercial, cooked unfilled pasta, carbonara sauce, added chicken, with or without added vegetables	0.00

02F40269	13510002	Pasta dish, homemade, cooked unfilled pasta, homemade carbonara sauce, added bacon, with or without added vegetables	0.00
02F40410	13510003	Pasta dish, commercial, cooked unfilled pasta, dairy based sauce	0.00
02F40388	13510004	Pasta dish, commercial, cooked unfilled pasta, dairy based sauce, added bacon, with or without added vegetables	0.00
02F40390	13509020	Pasta dish, commercial, cooked unfilled pasta, dairy based sauce, added chicken, with or without added vegetables	0.00
02F40391	13509021	Pasta dish, commercial, cooked unfilled pasta, dairy based sauce, added seafood, with or without added vegetables	0.00
02F40412	13510005	Pasta dish, commercial, cooked unfilled pasta, dairy based sauce, added vegetables	0.00
02F40446	13509022	Pasta dish, homemade, cooked unfilled pasta, commercial dairy based sauce	0.00
02F40268	13509023	Pasta dish, homemade, cooked unfilled pasta, commercial dairy based sauce, added bacon, with or without added vegetables	0.00
02F40265	13509024	Pasta dish, homemade, cooked unfilled pasta, commercial dairy based sauce, added chicken, with or without added vegetables	0.00
02F40453	13509025	Pasta dish, homemade, cooked unfilled pasta, commercial dairy based sauce, added meat, with or without added vegetables	0.00
02F40451	13509026	Pasta dish, homemade, cooked unfilled pasta, commercial dairy based sauce, added seafood, with or without added vegetables	0.00
02F40452	13509027	Pasta dish, homemade, cooked unfilled pasta, commercial dairy based sauce, added vegetables	0.00
02F40401	13510006	Pasta dish, homemade, cooked unfilled pasta, homemade dairy based sauce	0.00
02F40403	13509028	Pasta dish, homemade, cooked unfilled pasta, homemade dairy based sauce, added bacon, with or without added vegetables	0.00
02F40397	13509029	Pasta dish, homemade, cooked unfilled pasta, homemade dairy based sauce, added chicken, with or without added vegetables	0.00
02F40454	13509030	Pasta dish, homemade, cooked unfilled pasta, homemade dairy based sauce, added meat, with or without added vegetables	0.00
02F40398	13509031	Pasta dish, homemade, cooked unfilled pasta, homemade dairy based sauce, added seafood, with or without added vegetables	0.00
02F40400	13509032	Pasta dish, homemade, cooked unfilled pasta, homemade dairy based sauce, added vegetables	0.00
02F40343	13509033	Pasta dish, cooked unfilled pasta, oil based sauce	0.00

02F40345	13509034	Pasta dish, cooked unfilled pasta, oil based sauce, added bacon, with or without added vegetables	0.00
02F40341	13509035	Pasta dish, cooked unfilled pasta, oil based sauce, added chicken, with or without added vegetables	0.00
02F40448	13509036	Pasta dish, cooked unfilled pasta, oil based sauce, added seafood, with or without added vegetables	0.00
02F40342	13509037	Pasta dish, cooked unfilled pasta, oil based sauce, added vegetables	0.00
02F40369	13509038	Pasta dish, commercial, cooked unfilled pasta, tomato based sauce	0.00
02F40382	13509039	Pasta dish, commercial, cooked unfilled pasta, tomato based sauce, added bacon, with or without added vegetables	0.00
02F40370	13509040	Pasta dish, commercial, cooked unfilled pasta, tomato based sauce, added chicken, with or without added vegetables	0.00
02F40371	13509041	Pasta dish, commercial, cooked unfilled pasta, tomato based sauce, added seafood, with or without added vegetables	0.00
02F40383	13509042	Pasta dish, commercial, cooked unfilled pasta, tomato based sauce, added vegetables	0.00
02F40366	13509043	Pasta dish, homemade, cooked unfilled pasta, commercial tomato based sauce	0.00
02F40375	13509044	Pasta dish, homemade, cooked unfilled pasta, commercial tomato based sauce, added bacon, with or without added vegetables	0.00
02F40374	13509045	Pasta dish, homemade, cooked unfilled pasta, commercial tomato based sauce, added chicken, with or without added vegetables	0.00
02F40368	13509046	Pasta dish, homemade, cooked unfilled pasta, commercial tomato based sauce, added seafood, with or without added vegetables	0.00
02F40377	13509047	Pasta dish, homemade, cooked unfilled pasta, commercial tomato based sauce, added vegetables	0.00
02F40270	13509048	Pasta dish, homemade, cooked unfilled pasta, homemade tomato based sauce	0.00
02F40384	13509049	Pasta dish, homemade, cooked unfilled pasta, homemade tomato based sauce, added bacon, with or without added vegetables	0.00
02F40378	13509050	Pasta dish, homemade, cooked unfilled pasta, homemade tomato based sauce, added chicken, with or without added vegetables	0.00
02F40474	13509051	Pasta dish, homemade, cooked unfilled pasta, homemade tomato based sauce, added liver, with or without added vegetables	0.00
02F40379	13509052	Pasta dish, homemade, cooked unfilled pasta, homemade tomato based sauce, added mixed seafood, with or without added vegetables	0.00

02F40380	13509053	Pasta dish, homemade, cooked unfilled pasta, homemade tomato based sauce, added salmon or tuna, with or without added vegetables	0.00
02F40381	13509054	Pasta dish, homemade, cooked unfilled pasta, homemade tomato based sauce, added vegetables	0.00
02F40449	13509055	Pasta dish, cooked unfilled pasta, commercial tomato based sauce, added meat	0.00
02F40450	13509056	Pasta dish, cooked unfilled pasta, homemade tomato based sauce, added meat	0.00
02F40435	13509057	Pasta dish, cooked filled pasta, beef bolognese sauce	0.00
02F40447	13510007	Pasta dish, cooked filled pasta, dairy based sauce	0.00
02F40443	13509058	Pasta dish, cooked filled pasta, dairy based sauce, added bacon, with or without added vegetables	0.00
02F40475	13509059	Pasta dish, cooked filled pasta, dairy based sauce, added meat	0.00
02F40444	13509060	Pasta dish, cooked filled pasta, dairy based sauce, added vegetables	0.00
02F40441	13510008	Pasta dish, cooked filled pasta, oil based sauce	0.00
02F40442	13509061	Pasta dish, cooked filled pasta, oil based sauce, added vegetables	0.00
02F40438	13509062	Pasta dish, cooked filled pasta, tomato based sauce	0.00
02F40436	13509063	Pasta dish, cooked filled pasta, tomato based sauce, added bacon	0.00
02F40439	13509064	Pasta dish, cooked filled pasta, tomato based sauce, added meat	0.00
02F40440	13509065	Pasta dish, cooked filled pasta, tomato based sauce, added meat & vegetables	0.00
02F40437	13509066	Pasta dish, cooked filled pasta, tomato based sauce, added vegetables	0.00
02F40261	15602007	Tuna mornay, homemade, cooked unfilled pasta, homemade white sauce, cheese & breadcrumbs	0.00
02F40476	13509067	Pasta dish, not further defined	0.00
02F40232	13509068	Pasta in cream based sauce, dry mix only	0.00
10A10473	13509069	Pasta in cream based sauce, prepared from dry mix with regular fat milk cows & margarine spread	0.00
10A10463	13509070	Pasta in tomato based sauce, dry mix	0.00
10A10474	13509071	Pasta in tomato based sauce, prepared from dry mix with water & margarine spread	0.00
02F40212	13509072	Spaghetti in meat sauce, canned, regular	0.00
02F40211	13509073	Spaghetti in tomato & cheese sauce, canned, regular	0.00
02F40239	13509074	Spaghetti in tomato & cheese sauce, canned, reduced salt	0.00
02A10391	13513001	Gnocchi, potato, commercially prepared, boiled	0.00
02E40120	13401002	Cannoli, unfilled, fried	0.00
02E50330	13403002	Cannoli, filled with vanilla, ricotta & cream cheese filling	0.00
02E40103	13401003	Croissant, commercial, plain	0.00
02E40112	13403003	Croissant, chocolate filled, commercial	0.00
02E50337	13403004	Danish, custard filled	0.00

02E50246	13403005	Danish, custard & fruit filled	0.00
02E50338	13403006	Danish, custard & pecan filled, commercial	0.00
02E50256	13403010	Eclair or profiterole, cream & custard filled, commercial, chocolate icing	0.00
02E50257	13403008	Eclair or profiterole, custard filled, commercial, chocolate icing	0.00
02E50258	13403007	Eclair or profiterole, cream filled, commercial, chocolate icing	0.00
02E50270	13403011	Eclair or profiterole, mock cream filled, reduced fat, commercial, chocolate icing	0.00
02E50332	13403009	Eclair or profiterole, custard filled, homemade from basic ingredients	0.00
02E50340	13604006	Fritter, banana, fried or deep-fried	0.00
02E50341	13604007	Fritter, pineapple, fried or deep-fried	0.00
02E40105	13401004	Pastry, choux, commercial, baked, unfilled	0.00
02E40104	13401005	Pastry, choux, homemade from basic ingredients, raw	0.00
02E40110	13401006	Pastry, choux, homemade from basic ingredients, baked, unfilled	0.00
02E40094	13401010	Pastry, filo (fillo), commercial, raw	0.00
02E40095	13401011	Pastry, filo (fillo), commercial, baked	0.00
02E40096	13401012	Pastry, puff, with butter, commercial, raw	0.00
02E40109	13401013	Pastry, puff, with butter, commercial, baked	0.00
02E40108	13401014	Pastry, puff, vegetable oil, commercial, raw	0.00
02E40097	13401015	Pastry, puff, vegetable oil, commercial, baked	0.00
02E40111	13401016	Pastry, puff, commercial, raw, not further defined	0.00
02E40098	13401017	Pastry, shortcrust style, commercial, raw	0.00
02E40099	13401018	Pastry, shortcrust style, commercial, baked	0.00
02E40116	13401019	Pastry, shortcrust style, reduced fat, commercial, raw	0.00
02E40117	13401020	Pastry, shortcrust style, homemade from basic ingredients, baked	0.00
02E40100	13401021	Pastry, shortcrust, wholemeal, commercial, raw	0.00
02E40101	13401022	Pastry, shortcrust, wholemeal, commercial, baked	0.00
02E40102	13401023	Pastry, spring roll, homemade from basic ingredients, raw	0.00
02E40113	13401007	Pastry, dumpling wrapper style, raw	0.00
02E40114	13401008	Pastry, gow gee wrapper style, raw	0.00
02E40115	13401009	Pastry, dumpling wrapper, not further defined	0.00
02E40121	13401024	Pastry, for use in quiche recipes, raw	0.00
02E60268	13406001	Chiko roll, takeaway style, deep fried	0.00
08F10919	13406002	Curry puff, beef & vegetable, deep fried, commercial	0.00
08F10918	13406003	Curry puff, beef & vegetable, homemade from basic ingredients	0.00
02E60277	13405001	Dim sim, vegetable & meat filling, purchased frozen, microwaved or steamed	0.00
02E60266	13406004	Dim sim, vegetable &/or meat filling, takeaway style, deep fried	0.00
02E60374	13513008	Dumpling, savoury, plain, homemade from basic ingredients	0.00

02E60323	13406005	Dumpling or wonton, savoury, chicken, fried	0.00
02E60308	13405002	Dumpling or wonton, savoury, chicken, steamed, boiled or microwaved	0.00
02E60282	13406007	Dumpling or wonton, savoury, meat & vegetable filled, takeaway style, cooked	0.00
02E60348	13406006	Dumpling or wonton, savoury, meat, fried	0.00
02E60307	13405003	Dumpling or wonton, savoury, meat, steamed, boiled or microwaved	0.00
02E60326	13405004	Dumpling or wonton, savoury, meat & seafood, steamed, boiled or microwaved	0.00
02E60325	13406008	Dumpling or wonton, savoury, seafood, fried	0.00
02E60309	13405005	Dumpling or wonton, savoury, seafood, steamed, boiled or microwaved	0.00
02E60368	13405006	Dumpling or wonton, savoury, vegetable, steamed, boiled or microwaved	0.00
13B10298	24903001	Fritter, corn, fried or deep-fried	0.00
13B10332	18803001	Fritter, corned beef, fried or deep-fried	0.00
13B10297	24903002	Fritter, zucchini, fried or deep-fried	0.00
08F10910	24903005	Pakora (coated & deep fried vegetables), Indian restaurant-style	0.00
05A10754	13405007	Pastry, filled with salmon, with or without vegetables, homemade from basic ingredients	0.00
02E60279	13405008	Pastry, filled with spinach, commercial, ready to eat	0.00
05A10780	13405009	Pastry, filled with spinach, homemade from basic ingredients	0.00
02E60290	13405010	Pastry, filled with spinach & cheese, from frozen, baked, no added fat	0.00
02E60291	13405011	Pastry, filled with spinach & cheese, commercial, ready to eat	0.00
02E60298	13405012	Pasty, filled with vegetables, commercial, baked	0.00
02E60297	13405013	Pasty, filled with vegetables, homemade from basic ingredients	0.00
02E60274	13405014	Pasty, filled with vegetables & meat, commercial, ready to eat	0.00
02E60327	13405015	Pasty, filled with vegetables & meat, homemade from basic ingredients	0.00
02E60283	13405016	Pie, savoury, chicken & vegetable, commercial	0.00
02E60296	13405017	Pie, savoury, chicken & vegetable, homemade from basic ingredients	0.00
02E60292	13405018	Pie, savoury, meat, commercial	0.00
02E60267	13405019	Pie, savoury, meat, commercial, family size	0.00
02E60293	13405020	Pie, savoury, meat, from frozen, baked or microwaved	0.00
02E60284	13405021	Pie, savoury, meat & cheese, commercial	0.00
02E60351	13405022	Pie, savoury, meat & kidney, commercial	0.00
02E60350	13405023	Pie, savoury, meat, kidney & vegetable filling, homemade from basic ingredients	0.00
02E60324	13405024	Pie, savoury, meat & mushroom, from frozen, baked or microwaved	0.00

02E60349	13405025	Pie, savoury, meat & vegetable filling, homemade from basic ingredients	0.00
02E60322	13405026	Pie, savoury, meat filling, topped with potato, commercial	0.00
02E60299	13405027	Pie, savoury, pumpkin & fetta (feta), homemade from basic ingredients	0.00
05A10747	13405028	Pie, savoury, salmon & vegetable, homemade from basic ingredients	0.00
05A10812	13405029	Pie, savoury, seafood & vegetable, homemade from basic ingredients	0.00
02E60300	13405030	Pie, savoury, spinach & cheese, commercial	0.00
02E60306	13405031	Pie, savoury, vegetable, homemade from basic ingredients	0.00
08F10920	13405032	Pie, savoury, without pastry, meat filling, topped with mashed potato, commercial	0.00
08F10922	13405033	Pie, savoury, without pastry, meat filling, topped with mashed potato & cheese, homemade from basic ingredients	0.00
02E60316	13406009	Prawn toast, white bread, fried	0.00
08F10994	13406010	Samosa, meat & vegetable, deep fried	0.00
08F10911	13406011	Samosa, vegetable, deep fried	0.00
02E60294	13405035	Sausage roll, commercial, ready to eat	0.00
02E60295	13405036	Sausage roll, from frozen, baked or microwaved	0.00
02E60304	13405037	Sausage roll, homemade from basic ingredients, baked	0.00
02E60305	13405038	Sausage roll, chicken, homemade from basic ingredients, baked	0.00
02E60321	13406012	Spring roll, meat &/or vegetable, purchased frozen, baked	0.00
02E60285	13406013	Spring roll, meat &/or vegetable, purchased frozen, deep fried	0.00
02E60286	13406017	Spring roll, meat & vegetable filling, takeaway style, deep fried	0.00
02E60287	13406014	Spring roll, meat filling, takeaway style, deep fried	0.00
02E60288	13406016	Spring roll, vegetable filling, takeaway style, deep fried	0.00
02E60366	13406015	Spring roll, meat & vegetable filling, fried, homemade from basic ingredients	0.00
02E60352	13405039	Spring roll or rice paper roll, chicken & vegetable filling, fresh	0.00
02E60314	13405040	Spring roll or rice paper roll, chicken, prawn & vegetable filling, fresh	0.00
02E60353	13405041	Spring roll or rice paper roll, prawn & vegetable filling, fresh	0.00
02E60289	13405042	Spring roll or rice paper roll, vegetable filling, fresh	0.00
02E60313	13513006	Steamed bun, savoury, plain	0.00
02E60312	13513007	Steamed bun, savoury, pork	0.00
03B10174	17201044	Frittata, cheese/plain, homemade	0.00
03B10168	17201045	Frittata, cheese & bacon, homemade	0.00
03B10167	17201046	Frittata, cheese, bacon & mixed vegetable, homemade	0.00

03B10173	17201047	Frittata, cheese, chicken & mixed vegetable, homemade	0.00
03B10176	17201048	Frittata, cheese, meat & mixed vegetable, homemade	0.00
02E60361	17201049	Frittata, cheese & mixed vegetable, homemade	0.00
03B10172	17201050	Frittata, cheese & salmon or tuna, homemade	0.00
03B10166	17201051	Frittata, cheese & spinach, homemade	0.00
02E60263	13404003	Quiche, Lorraine, commercial, baked	0.00
02E60301	13404004	Quiche, Lorraine, homemade	0.00
02E60360	13404001	Quiche, bacon & vegetables, homemade	0.00
02E60331	13404002	Quiche, chicken & vegetable, homemade	0.00
02E60367	13404005	Quiche, meat & vegetable, homemade	0.00
02E60336	13404006	Quiche, mixed vegetables, homemade	0.00
02E60329	13404007	Quiche, plain, homemade	0.00
02E60317	13404008	Quiche, pumpkin, homemade	0.00
02E60357	13404009	Quiche, salmon or tuna, commercial	0.00
02E60358	13404010	Quiche, salmon or tuna, homemade from basic ingredients	0.00
02E60356	13404011	Quiche, spinach, commercial	0.00
02E60303	13404012	Quiche, spinach, homemade	0.00
02E60354	24903006	Zucchini slice, with bacon	0.00
02E60355	24903007	Zucchini slice, without bacon	0.00
02E50259	13402001	Baklava	0.00
06E10097	16901003	Crumble, apple, baked, homemade	0.00
06E10098	16901004	Crumble, apple & berry, baked, homemade	0.00
06E10147	16901005	Crumble, apple & rhubarb, baked, homemade	0.00
06E10148	16901006	Crumble, stone fruits, baked, homemade	0.00
02E50323	13305008	Dumpling, sweet, soaked in syrup	0.00
02E50248	13402002	Pie, sweet, apple, commercial	0.00
02E50255	13402004	Pie, sweet, apple, from frozen, baked	0.00
02E50298	13402005	Pie, sweet, apple, from frozen, reduced fat, baked	0.00
02E50269	13402006	Pie, sweet, apple, homemade	0.00
02E50289	13402003	Pie, sweet, apple, fast food outlet	0.00
02E50314	13402007	Pie, sweet, apple & berry, from frozen, baked	0.00
02E50324	13402008	Pie, sweet, apple & rhubarb	0.00
02E50247	13402009	Pie, sweet, fruit (apple or apricot), commercial	0.00
02E50285	13402010	Pie, sweet, fruit mince, commercial	0.00
02E50284	13402011	Pie, sweet, fruit mince, homemade	0.00
02E50343	13403012	Pie, sweet, lemon meringue, commercial	0.00
02E50251	13403013	Pie, sweet, lemon meringue, homemade	0.00
02E50292	13402012	Pie, sweet, mixed berry, homemade	0.00
02E50287	13402013	Pie, sweet, pecan, commercial	0.00
02E50268	13403014	Pie, sweet, pumpkin, commercial or homemade	0.00
02E50275	13402014	Pie, sweet, stone fruit, homemade	0.00
02E50325	13402015	Strudel, apple, commercial	0.00
02E50291	13402016	Strudel, apple, homemade	0.00
02E50331	13403015	Tart, caramel, homemade	0.00
02E50280	13403016	Tart, chocolate, homemade	0.00

02E50242	13403017	Tart, custard, commercial	0.00
02E50301	13403018	Tart, custard, homemade	0.00
02E50329	13403019	Tart, frangipane or almond, commercial or homemade	0.00
02E50243	13402017	Tart, jam, commercial	0.00
02E50283	13402018	Tart, jam, homemade	0.00
02E50277	13403020	Tart, lemon or lime, commercial	0.00
02E50279	13403021	Tart, lemon or lime, homemade	0.00
02E50276	13402019	Tart or tarte tatin, apricot, homemade	0.00
02E50327	13402020	Tart or tarte tatin, banana, homemade	0.00
02E50281	13402021	Tart or tarte tatin, pineapple, homemade	0.00
02E50328	13402022	Turnover, apple	0.00
10A10464	23110001	Casserole base, cream style, dry mix	0.00
10A10465	23110002	Casserole base, dry mix	0.00
10F20121	23304001	Dressing, Asian style, with lime juice, chilli and fish sauce	0.00
10F20117	23301001	Dressing, Caesar, commercial	0.00
10F20116	23301002	Dressing, Caesar, homemade	0.00
10F20106	23301003	Dressing, coleslaw, commercial, regular fat	0.00
10F20105	23302001	Dressing, coleslaw, commercial, reduced fat	0.00
10F20123	23302002	Dressing, creamy, commercial, 97% fat free	0.00
10F20125	23303001	Dressing, honey mustard, homemade	0.00
10F20115	23303002	Dressing, lemon vinaigrette, homemade	0.00
10F20124	23303003	Dressing, mustard, homemade	0.00
10F20104	23303004	Dressing, oil & vinegar, commercial, regular fat	0.00
10F20102	23303005	Dressing, oil, vinegar & vegetables/herbs, commercial, regular fat	0.00
10F20120	23301004	Dressing, ranch, commercial	0.00
10F20126	23303006	Dressing, sesame soy, homemade	0.00
10F20099	23301005	Dressing, thousand island, commercial, regular fat	0.00
10F20100	23302003	Dressing, thousand island, commercial, reduced fat	0.00
10F20103	23304002	Dressing, vinegar based, commercial, fat free	0.00
10F20101	23304003	Dressing, vinegar based & vegetables/herbs, commercial, fat free	0.00
10F20114	23303007	Dressing, vinaigrette, homemade	0.00
10A10459	23102001	Gravy powder, dry mix	0.00
10A10457	23101002	Gravy, prepared, commercial	0.00
10A10458	23101001	Gravy, prepared from dry powder with water	0.00
10A10528	23101003	Gravy, prepared from pan-drippings	0.00
10A10549	23102002	Gravy powder, dry mix, reduced salt	0.00
10A10551	23101005	Gravy, prepared, reduced salt, commercial	0.00
10A10550	23101004	Gravy, reduced salt, prepared from dry powder with water	0.00
13A12042	23109001	Horseradish, prepared	0.00
10A10476	23103001	Marinade, Asian style	0.00
10A10493	23103002	Marinade, teriyaki, commercial	0.00
10F20108	23301006	Mayonnaise, commercial, regular fat	0.00
10F20107	23302004	Mayonnaise, commercial, reduced fat	0.00

10F20109	23302005	Mayonnaise, commercial, low fat	0.00
10F20118	23302006	Mayonnaise, commercial, 97% fat free	0.00
10F20111	23301007	Mayonnaise, commercial, not further defined	0.00
10F20112	23301008	Mayonnaise, homemade	0.00
10F20119	23301009	Mayonnaise, soybean oil, homemade	0.00
10E10094	23103003	Mustard, cream style	0.00
10A10453	23109002	Paste, curry, Indian style, commercial	0.00
10A10534	23109010	Paste, curry, commercial, not further defined	0.00
10E10108	23109003	Paste, green curry, commercial	0.00
10E10109	23109004	Paste, green curry, homemade	0.00
10A10497	23109005	Paste, korma, commercial	0.00
10A10478	23109006	Paste, massaman curry, commercial	0.00
10A10501	23109007	Paste, red curry, commercial or homemade	0.00
10A10430	15504011	Paste, shrimp	0.00
10A10495	23109008	Paste, vindaloo, commercial	0.00
10A10517	23109009	Paste, wasabi, commercial	0.00
10A10533	27205001	Sauce, apple, commercial	0.00
10A10504	27205002	Sauce, apple, homemade	0.00
10A10488	23103004	Sauce, apricot, commercial or homemade	0.00
10A10442	23103005	Sauce, barbecue, commercial	0.00
10A10535	23106001	Sauce, barbecue, homemade	0.00
10A10518	23105001	Sauce, basil pesto, homemade	0.00
10A10469	23105002	Sauce, bearnaise, homemade	0.00
10A10436	23103006	Sauce, black bean, commercial	0.00
10A10454	23107001	Sauce, butter chicken, commercial	0.00
10A10526	23108001	Sauce, cheese, homemade with undefined cows milk & fat	0.00
10A10431	23103007	Sauce, chilli (chili), Asian, commercial	0.00
10A10489	23301010	Sauce, cocktail or seafood, commercial or homemade	0.00
10A10441	23103008	Sauce, cranberry, commercial	0.00
10A10552	23108002	Sauce, cream based, homemade from basic ingredients	0.00
10A10450	23107002	Sauce, curry, Asian, commercial	0.00
10A10506	23104001	Sauce, enchilada, commercial	0.00
10A10438	23103009	Sauce, fish, commercial	0.00
10A10539	23103010	Sauce, garlic, commercial	0.00
10A10537	23105003	Sauce, garlic, homemade	0.00
10A10433	23103011	Sauce, hoi sin, commercial	0.00
10A10470	23105004	Sauce, hollandaise, homemade	0.00
10A10508	23103012	Sauce, honey soy, commercial	0.00
10A10542	23103013	Sauce, horseradish, commercial	0.00
10A10530	23103014	Sauce, HP, commercial, regular	0.00
10A10492	23107003	Sauce, lemon chicken, commercial	0.00
10A20006	23103015	Sauce, mint, commercial or homemade	0.00
10A10505	23108003	Sauce, mushroom, homemade	0.00
10A10543	23101006	Sauce, mushroom, commercial, ready to eat	0.00
10A10544	23101007	Sauce, mushroom, prepared from commercial dry mix	0.00

10A10531	23108004	Sauce, mustard, homemade	0.00
10A10434	23103016	Sauce, oyster, commercial	0.00
10A10491	23103017	Sauce, pad Thai, commercial	0.00
10A10456	23107004	Sauce, pasta or simmer, not further defined, commercial, low fat	0.00
10A10460	23103018	Sauce, pasta, basil pesto, commercial	0.00
10A10507	18701023	Sauce, pasta, beef bolognese, commercial	0.00
10A10511	18701024	Sauce, pasta, bolognese, homemade using beef mince & commercial tomato based sauce	0.00
10A10512	18701025	Sauce, pasta, bolognese, homemade using beef mince & homemade tomato based sauce	0.00
10A10514	18701026	Sauce, pasta, bolognese, homemade using beef mince & homemade tomato based sauce with vegetables	0.00
10A10509	23107005	Sauce, pasta, carbonara, commercial	0.00
10A10555	23108005	Sauce, pasta, carbonara, homemade	0.00
10A10449	23107006	Sauce, pasta, cheese or cream-based, commercial	0.00
10A10554	23103019	Sauce, pasta, pesto, for pasta recipes	0.00
10A10525	23105005	Sauce, pasta, rocket pesto, homemade	0.00
10A10448	23104002	Sauce, pasta, tomato-based, commercial	0.00
10A10527	23106002	Sauce, pasta, tomato based, homemade	0.00
10A10553	23104003	Sauce, pasta, tomato pesto, commercial	0.00
10A10545	23103020	Sauce, pepper, commercial	0.00
10A10546	23108006	Sauce, pepper, homemade	0.00
10A10435	23103021	Sauce, plum, commercial	0.00
10A10510	23105006	Sauce, wine reduction, homemade	0.00
10A10462	23104004	Sauce, salsa, tomato-based, commercial	0.00
10A10548	23106003	Sauce, salsa, tomato-based, homemade from basic ingredients	0.00
10A10437	22202006	Sauce, satay, commercial	0.00
10A10540	22202007	Sauce, satay, homemade	0.00
08F10921	18712004	Sauce, savoury meat with vegetables and gravy, commercial	0.00
08F10923	18712005	Sauce, savoury meat with vegetables and gravy, homemade	0.00
10A10452	23107007	Sauce, simmer for chicken, not further defined, commercial	0.00
10A10461	23107008	Sauce, simmer, curry flavoured, commercial	0.00
10A10443	23103022	Sauce, soy, commercial, regular	0.00
10A10446	23103023	Sauce, soy, commercial, reduced salt	0.00
10A10486	23107009	Sauce, stroganoff, commercial	0.00
10A10483	23108007	Sauce, stroganoff, homemade	0.00
10A20004	23103024	Sauce, sweet chilli (chili), commercial	0.00
10A10432	23107010	Sauce, sweet & sour, commercial	0.00
10A10439	23103025	Sauce, tabasco, commercial	0.00
10A10451	23104005	Sauce, taco style, commercial	0.00
10A10479	23301011	Sauce, tartare (tartar), commercial, regular fat	0.00
10A10529	23301012	Sauce, tartare (tartar), homemade	0.00
10A10502	23103026	Sauce, teriyaki, commercial	0.00

10A10444	23104006	Sauce, tomato, commercial, regular	0.00
10A10532	23104007	Sauce, tomato, commercial, reduced salt	0.00
10A10445	23104008	Sauce, tomato, commercial, no added salt	0.00
10A10503	23106004	Sauce, tomato, homemade	0.00
10A10519	23104009	Sauce, tomato, not further defined	0.00
10A10522	23108008	Sauce, white, savoury, homemade with an undefined cows milk & fat	0.00
10A10440	23103027	Sauce, worcestershire, commercial	0.00
10A10541	23104010	Sauce, for use in hamburger recipes	0.00
10F20098	23305001	Vinegar (except balsamic vinegar)	0.00
10F20113	23305002	Vinegar, balsamic	0.00
02F40240	13604001	Batter for coating food, commercial, uncooked	0.00
02F40243	13604002	Batter for coating food, homemade, uncooked	0.00
02F40242	13515001	Breadcrumbs for coating food, commercial, uncooked	0.00
02F40244	13515002	Breadcrumbs for coating food, homemade, uncooked	0.00
02F40263	13604003	Coating, homemade, for chicken & meat, uncooked	0.00
02F40456	13604004	Coating, commercial, for fish & seafood, uncooked	0.00
02F40455	13604005	Coating, homemade, for fish & seafood, uncooked	0.00
02F40241	13604009	Tempura for coating food, commercial, uncooked	0.00
02F40264	13604010	Tempura for coating food, homemade, uncooked	0.00
11B10260	22204001	Chestnut puree, added sugar	0.00
11B10221	22202002	Peanut butter, smooth & crunchy, added sugar & salt	0.00
11B10222	22202003	Peanut butter, smooth & crunchy, added sugar, no added salt	0.00
11B10223	22202004	Peanut butter, smooth & crunchy, no added sugar or salt	0.00
11B10241	22202005	Peanut butter, not further identified	0.00
10F40022	31102001	Spread, yeast, cheesybite	0.00
10F40017	31102002	Spread, yeast, marmite	0.00
10F40018	31102003	Spread, yeast, vegemite, regular	0.00
10F40021	31102004	Spread, yeast, vegemite, my first vegemite	0.00
10F40020	31102005	Spread, vegetable & yeast extract, Promite	0.00
10F40024	31102006	Spread, vegetable & yeast extract, mightymite	0.00
10F40023	31102007	Spread, yeast, not further defined	0.00
09B30019	19406001	Cheese spread, cheddar, regular fat	0.00
09B30018	19406002	Cheese spread, cream cheese, regular fat	0.00
09B30022	19407001	Cheese spread, cream cheese, reduced fat	0.00
09B10192	19401001	Cheese, blended for pizza use, regular fat	0.00
09B10204	19403001	Cheese, bocconcini	0.00
09B10203	19405001	Cheese, brie	0.00
09B10176	19401002	Cheese, blue vein	0.00
09B10184	19405002	Cheese, camembert	0.00
09B10208	19401003	Cheese, cheddar, natural, flavoured	0.00
09B10181	19401004	Cheese, cheddar, natural, plain, regular fat	0.00
09B20072	19402001	Cheese, cheddar, natural, plain, reduced fat (~25%)	0.00
09B20076	19402002	Cheese, cheddar, natural, plain, reduced fat (~ 15%)	0.00
09B20083	19401005	Cheese, cheddar, natural, plain, not further defined	0.00

09B10179	19406003	Cheese, cheddar, processed, regular fat	0.00
09B20077	19407002	Cheese, cheddar, processed, reduced fat (~16%)	0.00
09B20078	19407003	Cheese, cheddar, processed, reduced fat (~8%)	0.00
09B20082	19407004	Cheese, cheddar, processed, reduced fat (3%)	0.00
09B20087	19407005	Cheese, cheddar, processed, reduced fat, not further defined	0.00
09B10198	19406004	Cheese, cheddar, processed, babybel style	0.00
09B10197	19406005	Cheese, cheddar, processed, stick style	0.00
09B10199	19406006	Cheese, processed, stick shape with string texture	0.00
09B20097	19407006	Cheese, processed, with added phytosterols	0.00
09B20084	19406007	Cheese, cheddar, processed, not further defined	0.00
09B20091	19401006	Cheese, cheddar, regular fat, not further defined	0.00
09B20092	19402003	Cheese, cheddar, reduced fat, not further defined	0.00
09B20085	19401007	Cheese, cheddar, not further defined	0.00
09B10185	19401008	Cheese, cheshire	0.00
09B10186	19401009	Cheese, colby style	0.00
09B20071	19403002	Cheese, cottage, regular fat	0.00
09B20088	19404001	Cheese, cottage, reduced fat	0.00
09B10209	19403003	Cheese, cottage or cream, sweet chilli flavoured	0.00
09B10171	19403004	Cheese, cream, plain, regular fat (35% fat)	0.00
09B10195	19404002	Cheese, cream, plain, reduced fat (25% fat)	0.00
09B10196	19404003	Cheese, cream, plain, reduced fat (5% fat)	0.00
09B10194	19403005	Cheese, cream, plain, not further defined	0.00
09B10207	19403006	Cheese, cream, fruit flavoured	0.00
09B10206	19403007	Cheese, cream, herb or spice flavoured	0.00
09B10174	19401010	Cheese, edam	0.00
09B10172	19401011	Cheese, fetta (feta), regular fat	0.00
09B20075	19402004	Cheese, fetta (feta), reduced fat	0.00
09B30021	19401012	Cheese, fetta (feta), not further defined	0.00
09B20079	19401013	Cheese, goat, firm	0.00
09B20080	19401014	Cheese, goat, soft	0.00
09B10187	19401015	Cheese, gloucester style	0.00
09B10177	19401016	Cheese, gouda	0.00
09B10188	19401017	Cheese, haloumi	0.00
09B10189	19401018	Cheese, havarti style	0.00
09B10200	19401019	Cheese, jarlsberg	0.00
09B10173	19401021	Cheese, mozzarella, regular fat	0.00
09B20073	19402005	Cheese, mozzarella, reduced fat	0.00
09B10202	19401022	Cheese, mozzarella, not further defined	0.00
09B10210	19401020	Cheese, mozzarella, buffalo	0.00
09B10178	19403008	Cheese, neufchatel	0.00
09B10175	19401023	Cheese, parmesan, dried, finely grated	0.00
09B10183	19401024	Cheese, parmesan, fresh	0.00
09B10190	19401025	Cheese, pecorino style	0.00
09B10191	19401026	Cheese, provolone style	0.00
09B20074	19404004	Cheese, quark	0.00
09B20070	19403009	Cheese, ricotta, regular fat	0.00

09B20094	19404005	Cheese, ricotta, reduced fat	0.00
09B20086	19403010	Cheese, ricotta, not further defined	0.00
09B10193	19401027	Cheese, romano style	0.00
09B20095	19401028	Cheese, smoked, regular fat	0.00
09B10180	19405003	Cheese, soft, white mould coated, not further defined	0.00
13B20295	20301001	Cheese, soy	0.00
09B10182	19401029	Cheese, swiss	0.00
09B20096	19408001	Cheese, not further defined	0.00
09B20089	19408002	Cheese, for use on sandwiches, not further defined	0.00
09B20098	19408005	Cheese, for use on sandwiches, not further defined	0.00
09B20093	19401030	Cheese, for use in hamburgers & Mexican food	0.00
09B10211	19408004	Cheese, for use in garden salads	0.00
09B10212	19408003	Cheese, for use on crackers or cheese platters	0.00
09A50051	19301004	Cream, rich or double thick	0.00
09A50042	19301001	Cream, pure, 35% fat	0.00
09A50043	19301002	Cream, regular thickened, 35% fat	0.00
09A50044	19301003	Cream, regular thickened, 35% fat, ultra high temperature treated	0.00
09A50049	19302001	Cream, reduced fat (~25%), canned	0.00
09A50053	19302002	Cream, regular thickened, light (~18% fat)	0.00
09A50045	19301005	Cream, whipped, aerosol, regular fat (~28%)	0.00
09A50052	19305001	Cream, imitation or mock (non-dairy)	0.00
09A50059	19305002	Cream, imitation or mock (non-dairy), reduced fat	0.00
09A50055	19306001	Cream, dairy, not further defined	0.00
09A50057	19306002	Cream, dairy, sugar sweetened, whipped, commercial	0.00
09A50058	19306003	Cream, dairy, sugar sweetened, whipped, homemade	0.00
09A50056	19306004	Cream, for use in commercial bakery products, not further defined	0.00
09A50046	19303001	Cream, sour, regular fat	0.00
09A50047	19304001	Cream, sour, light (~18% fat)	0.00
09A50048	19304002	Cream, sour, extra light (>12% fat)	0.00
09A50054	19303002	Cream, sour, not further defined	0.00
09D10218	19502001	Ice cream, Cassata-style dessert	0.00
12D10065	27303001	Gelato or sorbet, fruit or fruit juice, regular fat	0.00
09D10263	19508001	Gelato or sorbet, milk-based, chocolate or coffee flavoured, regular fat	0.00
09D10264	19508002	Gelato or sorbet, milk-based, vanilla & other flavours, regular fat	0.00
09D10283	19501001	Ice cream, all flavours, homemade from basic ingredients	0.00
09D10233	19501002	Ice cream, caramel flavour, regular fat	0.00
09D10244	19503001	Ice cream, caramel flavour, low fat (3% fat)	0.00
09D10259	19501003	Ice cream, caramel, honey & macadamia flavour, premium or rich (~15% fat)	0.00
09D10225	19501004	Ice cream, chocolate or coffee flavour, with or without chocolate chips, regular fat	0.00
09D10226	19503002	Ice cream, chocolate flavour, with or without chocolate chips, low fat (3% fat)	0.00

09D10275	19508003	Ice cream, chocolate flavour, regular fat, added vitamins A, B1, B2, C, D & folate, Ca & Fe	0.00
09D10234	19501005	Ice cream, chocolate & caramel swirl, regular fat	0.00
09D10257	19503003	Ice cream, chocolate & caramel swirl, low fat (3% fat)	0.00
09D10270	19501006	Ice cream, chocolate & honey swirl, with chocolate & nougat, premium or rich (~15% fat)	0.00
09D10239	19502002	Ice cream, mango flavour, regular fat	0.00
09D10235	19501007	Ice cream, mint flavour, regular fat	0.00
09D10236	19503004	Ice cream, mint flavour, low fat (3% fat)	0.00
09D10229	19501008	Ice cream, neopolitan or rainbow flavour, regular fat	0.00
09D10230	19503005	Ice cream, neopolitan or rainbow flavour, low fat (3% fat)	0.00
09D10232	19502003	Ice cream, passionfruit flavour, regular fat	0.00
09D10284	19502004	Ice cream, rum & raisin flavour, regular fat	0.00
09D10227	19501009	Ice cream, strawberry flavour, regular fat	0.00
09D10228	19503006	Ice cream, strawberry flavour, low fat (3% fat)	0.00
09D10243	19501010	Ice cream, vanilla flavour, premium or rich (~15% fat)	0.00
09D10212	19501011	Ice cream, vanilla flavour, regular fat	0.00
09D10222	19503007	Ice cream, vanilla flavour, low fat (3% fat)	0.00
09D10242	19502005	Ice cream, vanilla flavour, soft serve, fast food style	0.00
09D10262	19502006	Ice cream, vanilla flavour, not further defined	0.00
09D10261	19501012	Ice cream, vanilla flavour, with chocolate chip, regular fat	0.00
09D10278	19502007	Ice cream, vanilla flavour, with chocolate chips, reduced fat (6%)	0.00
09D10238	19501013	Ice cream, all other non-chocolate or coffee flavours, with chocolate chips, regular fat	0.00
09D10277	19501014	Ice cream, vanilla flavour, with nuts, regular fat	0.00
09D10240	19501015	Ice cream, vanilla flavour, with sweet biscuit, regular fat	0.00
09D10241	19502008	Ice cream, vanilla flavour, with sweet biscuit, low fat	0.00
09D10269	19501016	Ice cream, vanilla & caramel swirl, regular fat	0.00
09D10260	19501017	Ice cream, vanilla & chocolate or coffee flavour, regular fat	0.00
09D10250	19508005	Ice cream, soft serve, vanilla, with chocolate cream filled biscuit, fast food style	0.00
09D10280	19502009	Ice cream, not further defined	0.00
09D10279	19508004	Ice cream, fried	0.00
09D10281	16901002	Banana split, banana with added ice cream, whipped cream, topping & nuts	0.00
09D10249	19508007	Sundae, vanilla ice cream, caramel topping, fast food style	0.00
09D10276	19508008	Sundae, vanilla ice cream, chocolate topping	0.00
09D10247	19508009	Sundae, vanilla ice cream, chocolate topping, fast food style	0.00
09D10282	19508010	Sundae, vanilla ice cream, non-chocolate topping	0.00
09D10248	19508011	Sundae, vanilla ice cream, strawberry topping, fast food style	0.00

09D10252	19504001	Ice cream, bar, Turkish delight flavour, chocolate coated	0.00
09D10272	19505001	Ice cream, sandwich, vanilla flavour, with chocolate flavour biscuit, regular fat	0.00
09D10273	19504002	Ice cream, sandwich, vanilla flavour, with chocolate flavour biscuit & chocolate coated, regular fat	0.00
09D10254	19504003	Ice cream, stick, chocolate flavoured, chocolate & biscuit crumb coated, regular fat	0.00
09D10213	19504004	Ice cream, stick, flavoured, chocolate & biscuit crumb coated, regular fat	0.00
09D10215	19504005	Ice cream, stick, vanilla flavour, chocolate coated, regular fat	0.00
09D10271	19504006	Ice cream, stick, other non-chocolate flavours, chocolate coated, regular fat	0.00
09D10251	19504007	Ice cream, stick, vanilla flavour, chocolate coated, with almonds, regular fat	0.00
09D10265	19505002	Ice cream, stick, vanilla flavour, fruit ice confection-coated, regular fat	0.00
09D10268	19504008	Ice cream, stick, vanilla flavour, with caramel sauce, chocolate coated, regular fat	0.00
09D10266	19504009	Ice cream, chocolate flavour with confectionery & waffle cone, chocolate coated, regular fat	0.00
09D10221	19504010	Ice cream, vanilla flavour with confectionery & waffle cone, chocolate coated, regular fat	0.00
09D10253	19505003	Ice cream, other flavours with confectionery & waffle cone, chocolate coated, regular fat	0.00
09D10220	19506001	Ice cream, with fruit based ice confection, various flavours, regular fat	0.00
09D10267	19501018	Ice cream cake, not further defined	0.00
09D10224	19505004	Ice confection, stick, milk-based, chocolate flavoured, regular fat	0.00
09D10217	19505005	Ice confection, stick, milk-based, other flavours, regular fat	0.00
12D10049	27303002	Ice confection, stick, water-base, various flavours, regular fat	0.00
12D10050	27303003	Ice confection, stick or tub, fruit juice or fruit flavoured, regular fat	0.00
09D10285	19508006	Ice cream cone, soft serve vanilla ice cream, with wafer cone, fast food style	0.00
09A10228	32102001	Milk, human/breast, mature, fluid	0.00
07A10022	32101001	Infant formula, 6-12 months, prepared with water	0.00
07A10017	32101002	Infant formula, 6-12 months, added omega 3 fatty acids, prepared with water	0.00
07A10021	32103003	Toddler milk, added omega 3 fatty acids, prepared with water	0.00
07A10018	32103001	Toddler milk, regular, prepared with water	0.00
07A10020	32103002	Toddler milk, S26 Gold, prepared with water	0.00
01B10372	19109001	Babyccino, from cows milk	0.00
01B10374	20107001	Babyccino, from soy milk	0.00
09A10195	19210001	Buttermilk, cultured, 2% fat	0.00

09E10065	20106001	Milk, almond, fluid	0.00
09A20030	19106001	Milk, canned, evaporated, regular	0.00
09A20031	19106002	Milk, canned, evaporated, reduced fat (~ 2%)	0.00
09A20032	19106003	Milk, canned, evaporated, skim (<0.5% fat)	0.00
09A20028	19106004	Milk, canned, sweetened, condensed, regular	0.00
09A20029	19106005	Milk, canned, sweetened, condensed, skim (~0.2% fat)	0.00
09A10189	19801007	Milk, cow, fluid, flavoured, chocolate, regular fat	0.00
09A10190	19803003	Milk, cow, fluid, flavoured, chocolate, reduced fat	0.00
09A10239	19803004	Milk, cow, fluid, flavoured, chocolate, reduced fat, added Ca & vitamin D	0.00
09A10226	19803005	Milk, cow, fluid, flavoured, chocolate, reduced fat, added vitamins & minerals	0.00
09A10222	19801008	Milk, cow, fluid, flavoured, chocolate, not further defined	0.00
09A10193	19801009	Milk, cow, fluid, flavoured, coffee, regular fat	0.00
09A10194	19803006	Milk, cow, fluid, flavoured, coffee, reduced fat	0.00
09A10224	19801010	Milk, cow, fluid, flavoured, coffee, not further defined	0.00
09A10191	19802001	Milk, cow, fluid, flavoured, strawberry, regular fat	0.00
09A10192	19804001	Milk, cow, fluid, flavoured, strawberry, reduced fat	0.00
09A10207	19802002	Milk, cow, fluid, flavoured, all other flavours, regular fat	0.00
09A10208	19804002	Milk, cow, fluid, flavoured, all other flavours, reduced fat	0.00
09A10185	19101001	Milk, cow, fluid, regular fat (~3.5%)	0.00
09A10231	19101002	Milk, cow, fluid, regular fat (~3.5%), A2	0.00
09A10216	19101003	Milk, cow, fluid, regular fat (~3.5%), organic	0.00
09A10220	19101004	Milk, cow, fluid, regular fat (~3.5%), raw	0.00
09A10201	19102001	Milk, cow, fluid, regular fat (3.5%), added omega 3 polyunsaturates	0.00
09A10200	19103001	Milk, cow, fluid, reduced fat (1%)	0.00
09A10232	19103002	Milk, cow, fluid, reduced fat (1%), A2	0.00
09A10225	19103003	Milk, cow, fluid, reduced fat (1%), organic	0.00
09A10230	19103004	Milk, cow, fluid, reduced fat (1%), added milk solids	0.00
09A10229	19104001	Milk, cow, fluid, reduced fat (1%), added phytosterols	0.00
09A10238	19104002	Milk, cow, fluid, reduced fat (1%), increased Ca, added Fe & vitamins C & D	0.00
09A10184	19103005	Milk, cow, fluid, reduced fat (~1.5%), increased protein (~4%)	0.00
09A10197	19104003	Milk, cow, fluid, reduced fat (1.5%), added Ca, Mg, Zn & vitamin D	0.00
09A10198	19104004	Milk, cow, fluid, reduced fat (1.5%), added omega 3 polyunsaturates	0.00
09A10196	19104005	Milk, cow, fluid, reduced fat (1.5%), increased Ca, folate & vitamin D	0.00
09A10188	19105001	Milk, cow, fluid, skim (~0.15% fat)	0.00
09A10199	19105002	Milk, cow, fluid, skim (~0.15% fat), added milk solids	0.00
09A10217	19101005	Milk, cow, fluid, lactose free, regular fat (~3.5%)	0.00
09A10218	19103006	Milk, cow, fluid, lactose free, reduced fat (~1%)	0.00

09A10219	19109002	Milk, cow, fluid, lactose free, unfortified, not further defined	0.00
09A10203	19101006	Milk, cow, fluid, regular fat (~3.5%), not further defined	0.00
09A10204	19103007	Milk, cow, fluid, reduced fat (1-2%), not further defined	0.00
09A10205	19105003	Milk, cow, fluid, skim (~0.15% fat), not further defined	0.00
09A10206	19109003	Milk, cow, fluid, unflavoured, not further defined	0.00
09A10242	19109005	Milk, cow, fluid, unflavoured, not further defined	0.00
09A10236	19109004	Milk, dairy or dairy alternative, not further defined, for use in protein drinks	0.00
09A10187	19108001	Milk, goat, fluid, regular fat	0.00
09A30021	19107001	Milk, powder, cow, regular fat	0.00
09A30026	19107003	Milk, powder, cow, regular fat	0.00
09A30024	19101007	Milk, cow, fluid, prepared from dry powder, regular fat, standard dilution	0.00
09A30027	19101009	Milk, cow, fluid, prepared from dry powder, regular fat, standard dilution	0.00
09A30022	19107002	Milk, powder, cow, skim	0.00
09A30028	19107004	Milk, powder, cow, skim	0.00
09A30025	19105004	Milk, cow, fluid, prepared from dry powder, skim, standard dilution	0.00
09A30029	19105006	Milk, cow, fluid, prepared from dry powder, skim, standard dilution	0.00
09A30023	19108002	Milk, powder, goat, regular fat	0.00
09A10213	19101008	Milk & water, regular fat cow's milk & tap water	0.00
09A10214	19103008	Milk & water, reduced fat cow's milk & tap water	0.00
09A10215	19105005	Milk & water, skim cow's milk & tap water	0.00
09A40056	19805001	Drink, cows milk, added egg & sugar (eggnog or egg nog)	0.00
01B10388	11806001	Drink, probiotic, contains milk solids & sugar	0.00
01B10403	11806002	Drink, probiotic, contains milk solids & sugar, intense sweetened	0.00
09A40034	19801006	Iced chocolate, regular fat cows milk, with ice cream & whipped cream	0.00
01B10433	19801001	Iced coffee, regular fat cows milk	0.00
01B10426	19801002	Iced coffee, regular fat cows milk, with added sugar	0.00
01B10424	19801003	Iced coffee, regular fat cows milk, with ice cream	0.00
01B10423	19801004	Iced coffee, regular fat cows milk, with ice cream & whipped cream	0.00
01B10425	19801005	Iced coffee, regular fat cows milk, with whipped cream	0.00
01B10442	19803001	Iced coffee, reduced fat cows milk	0.00
01B10432	19803002	Iced coffee, reduced fat cows milk, with ice cream	0.00
01B10430	20201001	Iced coffee, regular fat soy milk	0.00
09A40059	11104002	Milkshake, bubble tea, non-chocolate or coffee flavour, cows milk, tapioca pearls	0.00
09A40025	19801011	Milkshake, chocolate or coffee flavour, regular fat cows milk, with ice cream	0.00

09A40026	19801012	Milkshake, chocolate or coffee flavour, regular fat cows milk, without ice cream	0.00
09A40045	19803007	Milkshake, chocolate or coffee flavour, reduced fat cows milk, with ice cream	0.00
09A40042	19803008	Milkshake, chocolate or coffee flavour, reduced fat cows milk, without ice cream	0.00
09A40027	19802003	Milkshake, non-chocolate or coffee flavours, regular fat cows milk, with ice cream	0.00
09A40037	19802004	Milkshake, non-chocolate or coffee flavours, regular fat cows milk, without ice cream	0.00
09A40030	19804003	Milkshake, non-chocolate or coffee flavours, reduced fat cows milk, with ice cream	0.00
09A40038	19804004	Milkshake, non-chocolate or coffee flavours, reduced fat cows milk, without ice cream	0.00
09E10066	20201002	Milkshake, non-chocolate or coffee flavours, soy milk, without ice cream	0.00
09A40047	19806001	Smoothie, cows milk, all flavours, added banana	0.00
09A40048	19806002	Smoothie, cows milk, all flavours, added berries	0.00
09A40049	19806003	Smoothie, cows milk, all flavours, added mango	0.00
09A40050	19806004	Smoothie, cows milk, all flavours, added mixed fruit	0.00
09A40052	19806005	Smoothie, cows milk, all flavours, added mixed fruit & egg	0.00
09A40064	19806006	Smoothie, cows milk, all flavours, added mixed fruit, guarana & herbal extracts	0.00
09A40061	19806007	Smoothie, cows milk, all flavours, added mixed fruit & nuts or seeds	0.00
09A40054	11301032	Smoothie, fruit juice based, added berries	0.00
09A40055	11301033	Smoothie, fruit juice based, added mixed fruit	0.00
09A40062	11301034	Smoothie, fruit juice based, added mixed fruit & nuts or seeds	0.00
09E10067	20107002	Smoothie, non-dairy base, all flavours, added mixed fruit	0.00
01B20514	11503005	Spider, cola soft drink & ice cream	0.00
09A10212	19804005	Thickshake, caramel flavour, fast food style	0.00
09A10210	19804006	Thickshake, chocolate flavour, fast food style	0.00
09A10211	19804007	Thickshake, strawberry flavour, fast food style	0.00
09A10209	19804008	Thickshake, vanilla flavour, fast food style	0.00
09A40057	19801013	Thickshake, chocolate or coffee flavour, regular fat cows milk, with ice cream	0.00
09A40058	19802005	Thickshake, non-chocolate or coffee flavour, regular fat cows milk, with ice cream	0.00
09A40063	19801014	Thickshake, all flavours, with cows milk, crushed ice, ice cream & confectionary, fast food style	0.00
09D20121	19602001	Custard, dairy, regular fat, banana, commercial	0.00
09D20122	19602002	Custard, dairy, regular fat, chocolate, commercial	0.00
09D20113	19602003	Custard, dairy, regular fat, vanilla, commercial	0.00
09D20114	19602004	Custard, dairy, reduced fat, vanilla, commercial	0.00
09D20115	19601001	Custard, egg, vanilla, homemade from basic ingredients	0.00

10A20003	12103004	Custard powder, dry mix, commercial	0.00
09D20117	19602005	Custard, dairy, vanilla, prepared from dry mix	0.00
09D20116	19602006	Custard, dairy, vanilla, not further defined	0.00
07D10165	32303001	Custard or yoghurt dessert, infant, dairy, regular fat, fruit flavoured, commercial	0.00
09D20131	19602007	Custard pudding, creme caramel or brulee, regular fat, vanilla, commercial	0.00
09D20119	19601002	Custard pudding or creme brulee, flavoured (except chocolate or coffee), homemade from basic ingredients	0.00
09C10097	19701001	Dairy dessert, chocolate, regular fat	0.00
09D20130	19701002	Dairy dessert, chocolate, regular fat, added vitamins A, B1, B2, C, D & folate, Ca & Fe, commercial	0.00
09C10136	19701003	Dairy dessert, flavours other than chocolate, regular fat	0.00
09D10219	19701004	Fromais frais, berry pieces or flavour, regular fat (5% fat)	0.00
09D10256	19701005	Fromais frais, fruit pieces or flavoured, regular fat (5% fat)	0.00
09C10159	19701006	Fromais frais, vanilla flavoured, regular fat (5% fat)	0.00
09D20129	17202003	Mousse, chocolate, homemade from basic ingredients	0.00
09D20118	19701007	Panna cotta, dairy, vanilla, homemade from basic ingredients	0.00
09D20128	19701008	Pudding, rice, coconut cream, homemade from basic ingredients	0.00
09D20120	19701009	Pudding, rice, dairy, vanilla, commercial	0.00
09D20123	19701010	Pudding, rice, dairy, vanilla, homemade from basic ingredients	0.00
09D20126	19701011	Pudding, rice, dairy, chocolate, commercial	0.00
09D20124	19701012	Pudding, rice, dairy, chocolate, homemade from basic ingredients	0.00
02E10484	19702007	Tiramisu, coffee, homemade from basic ingredients	0.00
09C10107	19201001	Yoghurt, Greek style (~10%), natural	0.00
09C10115	19201002	Yoghurt, Greek style (~8%), natural	0.00
09C10143	19201003	Yoghurt, Greek style, regular fat (~5%), natural	0.00
09C10144	19202001	Yoghurt, Greek style, reduced fat (~2%), natural	0.00
09C10093	19201004	Yoghurt, natural, regular fat (~4%)	0.00
09C10140	19201005	Yoghurt, natural, regular fat (~4%), homemade from basic ingredients	0.00
09C10139	19202002	Yoghurt, natural, reduced fat (~1.5%)	0.00
09C10141	19202003	Yoghurt, natural, reduced fat (~1.5%), homemade from basic ingredients	0.00
09C20042	19203001	Yoghurt, natural, low fat (<0.5%)	0.00
09C10098	19212001	Yoghurt, natural, not further defined	0.00
09C10153	19204002	Yoghurt, natural or Greek, high fat (~8%), added honey or sugar	0.00
09C10147	19204003	Yoghurt, natural or Greek, high fat (~6%), added berry pieces	0.00

09C10148	19204004	Yoghurt, natural or Greek, high fat (~6%), added mango &/or passionfruit pieces	0.00
09C10145	19205007	Yoghurt, natural or Greek, regular fat (~4%), added berry pieces	0.00
09C10118	19205008	Yoghurt, natural or Greek, regular fat (~4%), added mango &/or passionfruit pieces	0.00
09C10155	19205009	Yoghurt, natural or Greek, regular fat (~3.5%), added honey	0.00
09C10157	19206001	Yoghurt, apple & cinnamon flavoured, regular fat (~3%), added cereals	0.00
09C10094	19205001	Yoghurt, apricot, peach or nectarine pieces or flavoured, regular fat (~3%)	0.00
09C10101	19207001	Yoghurt, apricot, peach or nectarine pieces or flavoured, reduced fat (1%)	0.00
09C20043	19208001	Yoghurt, apricot, peach or nectarine pieces or flavoured, low fat (<0.5%)	0.00
09C20048	19209001	Yoghurt, apricot, peach or nectarine pieces or flavoured, low fat (<0.5%), intense sweetened	0.00
09C10109	19205002	Yoghurt, banana pieces or flavoured, regular fat (~3%)	0.00
09C10122	19211001	Yoghurt, banana pieces or flavoured, regular fat (~3%), added omega-3 polyunsaturates	0.00
09C10156	19206002	Yoghurt, banana & honey flavoured, regular fat (~3%), added cereals	0.00
09C20057	19209002	Yoghurt, banana & honey flavoured, low fat (<0.5%), intense sweetened	0.00
09C10095	19205003	Yoghurt, berry pieces or flavoured, regular fat (~3%)	0.00
09C10158	19206003	Yoghurt, berry flavoured, regular fat (~3%), added cereals	0.00
09C10123	19211002	Yoghurt, berry pieces or flavoured, regular fat (~3%), added omega-3 polyunsaturates	0.00
09C10111	19205004	Yoghurt, berry pieces or flavoured, with added fruit juice, reduced fat (~2%)	0.00
09C10168	19211003	Yoghurt, berry pieces or flavoured, reduced fat (2%), added vitamins A, C, E, & omega-3 polyunsaturates	0.00
09C10100	19207002	Yoghurt, berry pieces or flavoured, reduced fat (1%)	0.00
09C10137	19211004	Yoghurt, berry pieces or flavoured, reduced fat (1%), added fibre	0.00
09C20044	19208002	Yoghurt, berry pieces or flavoured, low fat (<0.5%)	0.00
09C20047	19209003	Yoghurt, berry pieces or flavoured, low fat (<0.5%), intense sweetened	0.00
09C20058	19211005	Yoghurt, berry pieces or flavoured, low fat (<0.5%), intense sweetened, added fibre	0.00
09C10164	19207003	Yoghurt, dessert flavoured, reduced fat (~1%)	0.00
09C20049	19209004	Yoghurt, dessert flavoured, low fat (<0.5%), intense sweetened	0.00
09C20061	19211006	Yoghurt, dessert flavoured, low fat (<0.5%), intense sweetened, added fibre	0.00
09C10117	19210002	Yoghurt, drinking style, fruit flavoured, reduced fat (1%)	0.00

09C10128	19210003	Yoghurt, drinking style, vanilla flavoured, reduced fat (1%)	0.00
09C10127	19205005	Yoghurt, flavoured, regular fat (~4%), homemade from basic ingredients	0.00
09C10163	19207004	Yoghurt, flavoured, reduced fat (~1.5%), homemade from basic ingredients	0.00
09C10169	19211007	Yoghurt, fruit pieces or flavoured, reduced fat (~2%), added Ca & vitamin D	0.00
09C10167	19211008	Yoghurt, fruit pieces, reduced fat (2%), added vitamins A, B1, B2, B3, B6, B12, C, D, E, & folate	0.00
09C10131	19204001	Yoghurt, honey flavoured, high fat (~5%)	0.00
09C10135	19207005	Yoghurt, honey flavoured, reduced fat (~2%)	0.00
09C10160	19207006	Yoghurt, honey flavoured, reduced fat (~1%)	0.00
09C10110	19205006	Yoghurt, mango pieces or flavoured, regular fat (~3%)	0.00
09C10102	19207007	Yoghurt, mango pieces or flavoured, reduced fat (1%)	0.00
09C20052	19209005	Yoghurt, mango pieces or flavoured, low fat (<0.5%), intense sweetened	0.00
09C10114	19205010	Yoghurt, passionfruit flavoured, regular fat (~3%)	0.00
09C10103	19207008	Yoghurt, passionfruit flavoured, reduced fat (1%)	0.00
09C10126	19211009	Yoghurt, passionfruit flavoured, reduced fat (1%), added fibre	0.00
09C10119	19207009	Yoghurt, passionfruit flavoured, reduced fat (1%), reduced sugar	0.00
09C20051	19209006	Yoghurt, passionfruit flavoured, low fat (<0.5%), intense sweetened	0.00
09C10130	19204005	Yoghurt, peach, mango & passionfruit pieces or flavoured, high fat (~6%)	0.00
09C10129	19205011	Yoghurt, peach & mango pieces or flavoured, regular fat (~3%)	0.00
09C10133	19207010	Yoghurt, peach & mango pieces or flavoured, reduced fat (2%)	0.00
09C10105	19207011	Yoghurt, peach & mango pieces or flavoured, reduced fat (1%)	0.00
09C10138	19211010	Yoghurt, peach & mango pieces or flavoured, reduced fat (1%), added fibre	0.00
09C20054	19209007	Yoghurt, peach & mango pieces or flavoured, low fat (<0.5%), intense sweetened	0.00
09C10113	19205012	Yoghurt, tropical fruit or fruit salad pieces or flavoured, regular fat (~3%)	0.00
09C10124	19211011	Yoghurt, tropical fruit or fruit salad pieces or flavoured, regular fat (~3%), added omega-3 polyunsaturates	0.00
09C10112	19207012	Yoghurt, tropical fruit or fruit salad pieces or flavoured, with added fruit juice, reduced fat (~2%)	0.00
09C10104	19207013	Yoghurt, tropical fruit or fruit salad pieces or flavoured, reduced fat (1%)	0.00
09C10116	19209008	Yoghurt, tropical fruit or fruit salad pieces or flavoured, low fat (<0.5%), intense sweetened	0.00

09C20062	19211012	Yoghurt, tropical fruit or fruit salad pieces or flavoured, low fat (<0.5%), intense sweetened, added fibre	0.00
09C10096	19205013	Yoghurt, vanilla flavoured, regular fat (~3%)	0.00
09C10121	19211013	Yoghurt, vanilla flavoured, regular fat (~3%), added omega-3 polyunsaturates	0.00
09C10134	19207014	Yoghurt, vanilla flavoured, reduced fat (2%), added berry pieces	0.00
09C10125	19207015	Yoghurt, vanilla flavoured, reduced fat (~1%)	0.00
09C10165	19211014	Yoghurt, vanilla flavoured, reduced fat (~1%), added fibre	0.00
09C20055	19209009	Yoghurt, vanilla flavoured, reduced fat (~1%), intense sweetened	0.00
09C20045	19208003	Yoghurt, vanilla flavoured, low fat (<0.5%)	0.00
09C20050	19209010	Yoghurt, vanilla flavoured, low fat (<0.5%), intense sweetened	0.00
09C20053	19211015	Yoghurt, vanilla flavoured, low fat (<0.5%), with added protein & fibre, intense sweetened	0.00
09C10161	19212002	Yoghurt, not further defined	0.00
09C10166	19108003	Sheep's, milk or yoghurt	0.00
09D10214	19507001	Yoghurt, frozen, berry flavoured, regular fat	0.00
09D10246	19507002	Yoghurt, frozen, tropical or fruit salad flavoured, regular fat	0.00
13B20279	20401001	Ice confection, non-dairy, chocolate or coffee flavour, regular fat	0.00
13B20218	20401002	Ice confection, non-dairy, vanilla flavour, regular fat	0.00
13B20230	25201003	Miso, soyabean paste	0.00
13B20215	20101001	Beverage base, soy, dry powder (not infant food)	0.00
09A10233	20106002	Milk, oat, fluid, unfortified	0.00
09A10234	20106003	Milk, oat, fluid, added calcium	0.00
09A10202	20106004	Milk, rice, fluid, added calcium	0.00
09A10235	20106005	Milk, rice, fluid, protein enriched, added calcium	0.00
13B20216	20101002	Soy beverage, regular fat (~3%), unfortified	0.00
13B20223	20102001	Soy beverage, regular fat (~3%), added Ca	0.00
13B20263	20102002	Soy beverage, regular fat (~3%), added Ca & vitamins A, B2, & B12	0.00
13B20224	20102003	Soy beverage, regular fat (~3%), added Ca & vitamins A, B1, B2 & B12	0.00
13B20255	20102004	Soy beverage, regular fat (~3%), added Ca & vitamins A, B1, B2, B6, B12, & D	0.00
13B20265	20101003	Soy beverage, regular fat (~3%), homemade from basic ingredients, unfortified	0.00
13B20254	20103001	Soy beverage, reduced fat (~1% fat), unfortified	0.00
13B20261	20104001	Soy beverage, reduced fat (~1.5% fat), added Ca	0.00
13B20236	20104002	Soy beverage, reduced fat (~1.5% fat), added vitamins A, B1, B2, B3, B6, B12, C, E, folate & Ca & Fe	0.00
13B20289	20104003	Soy beverage, reduced fat (~1.5% fat), added fibre, Ca & vitamins A, B1, B2, B6 & B12	0.00

13B20264	20104004	Soy beverage, reduced fat (~1% fat), added Ca & vitamins A, B2 & B12	0.00
13B20217	20104005	Soy beverage, reduced fat (~1% fat), added Ca & vitamins A, B1, B2 & B12	0.00
13B20225	20105001	Soy beverage, low fat (~ 0.1%), added Ca & vitamins A, B1, B2 & B12	0.00
13B20240	20107003	Soy beverage, regular fat (~3%), unflavoured, not further defined	0.00
13B20241	20107004	Soy beverage, reduced fat (1-2%), unflavoured, not further defined	0.00
13B20242	20107005	Soy beverage, unflavoured, not further defined	0.00
13B20227	20201003	Soy beverage, chocolate flavoured, regular fat (~3%), added Ca & vitamins A, B1, B2 & B12	0.00
13B20226	20202001	Soy beverage, chocolate flavoured, reduced fat (~ 1.5%), added Ca & vitamins A, B1, B2 & B12	0.00
13B20228	20202002	Soy beverage, coffee flavoured, reduced fat (~ 1.5%), added Ca & vitamins A, B1, B2 & B12	0.00
13B20235	25202009	Falafel, chickpea patty, deep fried, fat not further defined	0.00
08G10025	20601003	Sausage, vegetarian style, raw	0.00
08G10026	20601004	Sausage, vegetarian style, added Fe, Zn and vitamin B12, raw	0.00
08G10027	20601001	Meat alternative, commercial, cooked, no added fat	0.00
08G10028	20601002	Meat alternative, commercial, cooked, fat not further defined	0.00
13B20214	20601006	Tempeh (fermented soy beans), fried	0.00
13B20210	20601007	Tofu (soy bean curd), firm, as purchased	0.00
13B20209	20601008	Tofu (soy bean curd), silken or soft, as purchased	0.00
13B20211	20601009	Tofu (soy bean curd), smoked, as purchased	0.00
13B20251	20601010	Tofu (soy bean curd), fried, stir-fried, grilled or BBQ'd, no added fat	0.00
13B20274	20601011	Tofu (soy bean curd), fried, stir-fried, grilled or BBQ'd, fat not further defined	0.00
13B20213	20601012	Tofu (soy bean curd), burger patty, as purchased	0.00
13B20212	20601005	Soy bean curd skins, dried, rehydrated in boiling water	0.00
13B10283	25202010	Rissole or patty, vegetable or lentil, commercial, baked, fried, grilled or BBQ'd, no added fat	0.00
13B10292	25202011	Rissole or patty, vegetable or lentil, commercial, baked, fried, grilled or BBQ'd, fat not further defined	0.00
13B10295	25202012	Rissole or patty, vegetable or lentil, homemade, baked, fried, grilled or BBQ'd, no added fat	0.00
13B10320	25202013	Rissole or patty, vegetable or lentil, homemade, baked, fried, grilled or BBQ'd, fat not further defined	0.00
08G10029	20601013	Vegetarian meat, bacon, cooked, with or without added fat	0.00
13B20244	20502001	Yoghurt, soy based, apricot or mango flavoured, reduced fat (approx. 1%)	0.00
13B20237	20501001	Yoghurt, soy based, berry flavoured, regular fat (approx. 3%)	0.00

13B20243	20502002	Yoghurt, soy based, berry flavoured, reduced fat (1%)	0.00
13B20219	20502003	Yoghurt, soy based, vanilla flavoured, reduced fat (approx. 1%)	0.00
04A10074	14101001	Butter, plain, salted	0.00
04A10075	14101002	Butter, plain, reduced salt (sodium < 350 mg /100 g)	0.00
04A10076	14101003	Butter, plain, no added salt	0.00
04A10087	14101004	Butter, not further defined	0.00
04A10086	14102002	Butter, garlic or herb, homemade with butter or dairy blend	0.00
04A10094	14101005	Butter, spreadable, regular (~80% fat)	0.00
04A10095	14101006	Butter, spreadable, reduced fat (~60% fat)	0.00
04A10077	14102001	Ghee, clarified butter	0.00
04A10078	14201001	Dairy blend, butter & edible oil spread (~80% fat), sodium 485 mg/100 g	0.00
04A10084	14201002	Dairy blend, butter & edible oil spread (~80% fat), reduced salt (sodium 290 mg/100 g)	0.00
04A10082	14201003	Dairy blend, butter & edible oil spread (70% fat), sodium 485 mg/100 g	0.00
04A10083	14201004	Dairy blend, butter & edible oil spread (70% fat), reduced salt (sodium 280 mg/100 g)	0.00
04A10089	14202001	Dairy blend, butter & edible oil spread, reduced fat (60% fat), sodium 400 mg/100 g	0.00
04A10090	14202002	Dairy blend, butter & edible oil spread, reduced fat (60% fat) & salt (sodium 200 mg/100 g)	0.00
04A10093	14202003	Dairy blend, butter & edible oil spread, reduced fat (40% fat), sodium 510 mg/100 g	0.00
04A10091	14202004	Dairy blend, butter & edible oil spread, reduced fat (40% fat) & sodium 380 mg/100 g	0.00
04A10092	14202005	Dairy blend, butter & edible oil spread, reduced fat (16% fat) & sodium 390 mg/100 g	0.00
04A10098	14201005	Dairy blend, butter & edible oil spread, regular fat (>60% fat), regular salt	0.00
04A10096	14202006	Dairy blend, butter & edible oil spread, reduced fat (<60% fat), regular salt	0.00
04A10097	14202007	Dairy blend, butter & edible oil spread, reduced fat (<60% fat) & salt	0.00
04A10088	14203001	Dairy blend, butter & edible oil spread, not further defined	0.00
04B10077	14301001	Margarine spread, polyunsaturated (70% fat)	0.00
04B10078	14301002	Margarine spread, polyunsaturated (70% fat), reduced salt (sodium = 280 mg/100g)	0.00
04B10090	14301003	Margarine spread, polyunsaturated (70% fat), reduced salt (sodium = 340 mg/100 g), no added milk, added vitamin E	0.00
04B10104	14302001	Margarine spread, polyunsaturated, reduced fat (60% fat), sodium = 790 mg/100 g	0.00
04B10083	14302002	Margarine spread, polyunsaturated, reduced fat (50% fat) & salt (sodium = 360 mg/100 g)	0.00

04B10089	14302003	Margarine spread, polyunsaturated, reduced fat (50% fat) & salt (sodium = 340 mg/100 g), no added milk, added vitamin E	0.00
04B10076	14302004	Margarine spread, polyunsaturated, reduced fat (40% fat), no added salt or milk	0.00
04B10084	14302005	Margarine spread, polyunsaturated, reduced fat (25% fat) & salt (sodium = 360 mg/100 g)	0.00
04B10085	14306001	Margarine spread, polyunsaturated (65% fat), reduced salt (sodium = 360 mg/100 g), added phytosterols	0.00
04B10103	14302006	Margarine spread, polyunsaturated (60% fat), reduced salt (Na=340 mg/100 g), no added milk, added vitamin E & phytosterols	0.00
04B10086	14306002	Margarine spread, polyunsaturated, reduced fat (~40% fat) & salt (sodium = 360 mg/100 g), added phytosterols	0.00
04B10087	14306003	Margarine spread, polyunsaturated, reduced fat (~25% fat) & salt (sodium = 360 mg/100 g), added phytosterols	0.00
04B10094	14301004	Margarine spread, polyunsaturated, regular (>50% fat) & reduced salt (sodium <360 mg)	0.00
04B10095	14301005	Margarine spread, polyunsaturated, regular (>50% fat), not further defined	0.00
04B10096	14302007	Margarine spread, polyunsaturated, reduced fat (<50% fat) & salt (sodium <360 mg)	0.00
04B10080	14307002	Margarine spread, polyunsaturated, not further defined	0.00
04B20106	14303001	Margarine spread, monounsaturated (80% fat), rice bran oil, unfortified	0.00
04B20084	14306004	Margarine spread, monounsaturated (70%), reduced salt (sodium = 350 mg/100 g), added phytosterols	0.00
04B20080	14303002	Margarine spread, monounsaturated (65% fat)	0.00
04B20082	14303003	Margarine spread, monounsaturated (65% fat), reduced salt (sodium = 360mg/100 g)	0.00
04B20101	14303004	Margarine spread, monounsaturated (65% fat), reduced salt (sodium = 300 mg/100 g)	0.00
04B20104	14304001	Margarine spread, monounsaturated, reduced fat (60% fat), no added salt or milk	0.00
04B20097	14304002	Margarine spread, monounsaturated, reduced fat (50% fat)	0.00
04B20100	14304003	Margarine spread, monounsaturated, reduced fat (50% fat) & salt (sodium = 465 mg/100 g)	0.00
04B20096	14304004	Margarine spread, monounsaturated, reduced fat (50% fat) & salt (sodium = 350 mg/100 g)	0.00
04B20105	14304005	Margarine spread, monounsaturated, reduced fat (50% fat) & salt (sodium = 350 mg/100 g), no added milk	0.00
04B20102	14306005	Margarine spread, monounsaturated, reduced fat (45% fat) & salt (sodium = 350 mg/100 g), added phytosterols	0.00

04B20098	14304006	Margarine spread, monounsaturated, reduced fat (30% fat) & salt (sodium = 350 mg/100 g)	0.00
04B20103	14306006	Margarine spread, monounsaturated, reduced fat (25% fat) & salt (sodium = 350 mg/100 g), added vitamin E & phytosterols	0.00
04B20109	14303005	Margarine spread, monounsaturated, regular (>65% fat) & reduced salt (sodium <360 mg)	0.00
04B20110	14303006	Margarine spread, monounsaturated, regular (>65% fat), not further defined	0.00
04B20108	14304007	Margarine spread, monounsaturated, reduced fat (<50% fat) & reduced salt (sodium ~360 mg)	0.00
04B20107	14304008	Margarine spread, monounsaturated, reduced fat (<50% fat), not further defined	0.00
04B20095	14307001	Margarine spread, monounsaturated, not further defined	0.00
04B20086	14303007	Margarine spread, olive oil blend (65% fat), reduced salt (sodium = 360mg/100 g)	0.00
04B10091	14303008	Margarine spread, olive oil blend, (65% fat), reduced salt (sodium = 340 mg/100 g), no added milk, added vitamin E	0.00
04B20087	14304009	Margarine spread, olive oil blend (55% fat), reduced salt (sodium = 350mg/100 g)	0.00
04B10092	14306007	Margarine spread, olive oil blend (50% fat), reduced salt (sodium = 360 mg/100 g), added phytosterols	0.00
04B10093	14307003	Margarine spread, monounsaturated or polyunsaturated, regular fat (>50% fat) & salt	0.00
04B10097	14307004	Margarine spread, monounsaturated or polyunsaturated, regular fat (>50% fat) & reduced salt (sodium <360 mg)	0.00
04B10098	14307005	Margarine spread, monounsaturated or polyunsaturated, regular fat (>50% fat), not further defined	0.00
04B10099	14307006	Margarine spread, monounsaturated or polyunsaturated, reduced fat (<50% fat), regular salt	0.00
04B10100	14307007	Margarine spread, monounsaturated or polyunsaturated, reduced fat (<50% fat) & salt (sodium ~360 mg)	0.00
04B10101	14307008	Margarine spread, monounsaturated or polyunsaturated, reduced fat (<50% fat), not further defined	0.00
04B10102	14307009	Margarine spread, monounsaturated or polyunsaturated, regular salt, not further defined	0.00
04B10105	14307010	Margarine spread, monounsaturated or polyunsaturated, reduced salt, not further defined	0.00
04B10081	14307011	Margarine spread, monounsaturated or polyunsaturated, not further defined	0.00
04D10043	14203002	Fat, butter or dairy blend, for homemade cakes, biscuits & puddings, not further defined	0.00

04D10045	14602002	Fat, butter, dairy blend, ghee or margarine, not further defined, for use in meat, fish, poultry & vegetable recipes	0.00
04B10088	14601001	Fat, butter, dairy blend or margarine spread, not further defined	0.00
04B10106	14601003	Fat, butter, dairy blend or margarine spread, not further defined	0.00
04B10082	14601002	Fat, dairy blend or margarine spread, not further defined	0.00
04B20079	14305001	Margarine, cooking	0.00
04C10106	14402001	Oil, almond	0.00
04C10110	14402002	Oil, blend of monounsaturated vegetable oils	0.00
04C10093	14401001	Oil, blend of polyunsaturated vegetable oils	0.00
04C10096	14402003	Oil, canola	0.00
04C10117	14402004	Oil, canola & red palm blend	0.00
04C10100	14502002	Oil, copha	0.00
04C10105	14401002	Oil, cottonseed	0.00
04C10104	14401003	Oil, grapeseed	0.00
04C10114	14401004	Oil, linseed or flaxseed	0.00
04C10107	14402005	Oil, macadamia	0.00
04C10095	14401005	Oil, maize	0.00
04C10102	14402006	Oil, mustard seed	0.00
04C10094	14402007	Oil, olive	0.00
04C10108	14502003	Oil, palm	0.00
04C10092	14402008	Oil, peanut	0.00
04C10109	14402009	Oil, rice bran	0.00
04C10097	14401006	Oil, safflower	0.00
04C10103	14401007	Oil, sesame	0.00
04C10099	14401008	Oil, soybean	0.00
04C10098	14401009	Oil, sunflower	0.00
04C10115	14401010	Oil, vegetable	0.00
04C10111	14403001	Oil, not further defined	0.00
04C10112	14403002	Oil, not further defined, for use in baked product recipes & deep fried takeaway foods	0.00
04D10039	14501001	Dripping, beef	0.00
04D10038	14503001	Fat, solid, blend of animal & vegetable oils	0.00
04C10101	14502001	Fat, solid, vegetable oil based	0.00
04B20099	14602001	Fat, for frying at home, not further defined	0.00
04D10044	14602003	Fat or oil, not further defined, for use in home cooked meat, fish, poultry & mixed dish recipes	0.00
04B20113	14602004	Fat or oil, not further defined, for use in omelette, scrambled egg & wet cooked vegetables recipes	0.00
04D10046	14602005	Fat or oil, not further defined, for use in homemade cake, biscuit & slice recipes	0.00
04D10047	14602006	Fat or oil, not further defined, for use in homemade cake, biscuit & slice using dry mixes	0.00

04C10116	14602007	Oil, other than olive or canola oils, for use in home cooked meat, fish, poultry & dry cooked vegetable recipes	0.00
04D10048	14602008	Oil, not further defined, for use in wet cooked vegetable recipes	0.00
04D10040	14501002	Lard	0.00
04B20091	14501004	Shortening, commercial, animal fat (for short pastry, pie bases, tarts and flans)	0.00
04B20094	14503002	Shortening, commercial, blend of animal & vegetable fat (for cakes, muffins, shortbreads & biscuits)	0.00
04B20089	14502004	Shortening, commercial, vegetable fat (for bread, buns & yeast doughs)	0.00
04B20092	14502005	Shortening, commercial, vegetable fat (for flaky pastry, pie tops, sausage rolls & danishes)	0.00
04B20093	14502006	Shortening, commercial, vegetable fat (for coatings, creams, icings, confectionery & fillings)	0.00
04D10041	14501003	Suet	0.00
03A10076	17102001	Egg, chicken, whole, omega-3 polyunsaturate enriched, raw	0.00
03A10086	17102002	Egg, chicken, whole, omega-3 polyunsaturate enriched, boiled	0.00
03A10075	17101001	Egg, chicken, whole, raw	0.00
03A10104	17101002	Egg, chicken, whole, baked, no added fat	0.00
03A10136	17101003	Egg, chicken, whole, baked, added fat not further defined	0.00
03A10077	17101004	Egg, chicken, whole, hard-boiled	0.00
03B10078	17201002	Egg, chicken, whole, hard-boiled, mashed with mayonnaise	0.00
03A10110	17201001	Egg, chicken, whole, curried	0.00
03A10078	17101010	Egg, chicken, whole, poached, no added fat	0.00
03A10109	17101011	Egg, chicken, whole, poached, fat not further defined	0.00
03A10124	17101005	Egg, chicken, whole, fried, no fat added	0.00
03A10095	17101006	Egg, chicken, whole, fried, butter or dairy blend	0.00
03A10090	17101007	Egg, chicken, whole, fried, margarine spread	0.00
03A10088	17101008	Egg, chicken, whole, fried, oil not further defined	0.00
03A10089	17101009	Egg, chicken, whole, fried, fat not further defined	0.00
03A10111	17101012	Egg, chicken, whole, cooked, no added fat	0.00
03A10137	17103006	Egg, whole, preserved, cooked	0.00
03A10080	17101013	Egg, chicken, white (albumen) only, raw	0.00
03A10081	17101014	Egg, chicken, white (albumen) only, hard-boiled	0.00
03A10134	17101015	Egg, chicken, white (albumen) only, fried, no added fat	0.00
03A10120	17101016	Egg, chicken, white (albumen) only, fried, fat not further defined	0.00
03A10082	17101017	Egg, chicken, yolk, raw	0.00
03A10083	17101018	Egg, chicken, yolk, hard-boiled	0.00
03A10123	17101019	Egg, chicken, yolk, fried, no fat added	0.00
03A10118	17101020	Egg, chicken, yolk, fried, fat not further defined	0.00

03A10113	17201035	Egg, chicken, yolk, scrambled, with cows milk, no fat added	0.00
03A10135	17201036	Egg, chicken, yolk, scrambled, with cows milk, fat not further defined	0.00
03A10084	17103001	Egg, duck, whole, raw	0.00
03A10087	17103002	Egg, duck, whole, boiled	0.00
03A10133	17103003	Egg, duck, whole, fried, fat not further defined	0.00
03A10115	17201042	Egg, duck, scrambled, with cows milk, no fat added	0.00
03A10116	17201043	Egg, duck, scrambled, with cows milk & fat not further defined	0.00
03A10138	17103007	Egg, emu, whole, raw	0.00
03A10139	17103008	Egg, emu, scrambled, with cows milk, no added fat	0.00
03A10085	17103004	Egg, quail, whole, raw	0.00
03A10131	17103005	Egg, quail, whole, fried, fat not further defined	0.00
03A10125	17201003	Egg, chicken, whole, fried with bacon, with or without added fat	0.00
03A10128	17201004	Egg, chicken, whole, fried with cheese &/or vegetables, with or without added fat	0.00
03A10126	17201005	Egg, chicken, whole, poached with bacon, with or without added fat	0.00
03A10129	17201006	Egg, chicken, whole, poached with cheese &/or vegetables, with or without added fat	0.00
03A10099	17201007	Egg, chicken, scrambled, with cows milk, no fat added	0.00
03B10079	17201008	Egg, chicken, scrambled, with cows milk, cooked with butter or dairy blend	0.00
03A10106	17201009	Egg, chicken, scrambled, with cows milk, cooked with margarine spread	0.00
03A10100	17201010	Egg, chicken, scrambled, with cows milk, cooked with oil not further defined	0.00
03A10102	17201011	Egg, chicken, scrambled, with cows milk, cooked with fat not further defined	0.00
03B10129	17201012	Egg, chicken, scrambled or omelette, with bacon, cooked without added fat	0.00
03B10155	17201013	Egg, chicken, scrambled or omelette, with bacon, cooked with added fat	0.00
03B10102	17201014	Egg, chicken, scrambled or omelette, with bacon & cheese, cooked without added fat	0.00
03B10151	17201015	Egg, chicken, scrambled or omelette, with bacon & cheese, cooked with added fat	0.00
03B10104	17201016	Egg, chicken, scrambled or omelette, with bacon, cheese & vegetables, cooked without added fat	0.00
03B10136	17201017	Egg, chicken, scrambled or omelette, with bacon, cheese & vegetables, cooked with added fat	0.00
03B10126	17201018	Egg, chicken, scrambled or omelette, with bacon & vegetables, cooked without added fat	0.00
03B10154	17201019	Egg, chicken, scrambled or omelette, with bacon & vegetables, cooked with added fat	0.00
03B10181	17201058	Egg, chicken, scrambled or omelette, with canned spaghetti, cooked without added fat	0.00

03B10096	17201020	Egg, chicken, scrambled or omelette, with cheese, cooked without added fat	0.00
03A10107	17201021	Egg, chicken, scrambled or omelette, with cheese, cooked with added fat	0.00
03B10111	17201022	Egg, chicken, scrambled or omelette, with cheese, chicken & vegetables, cooked without added fat	0.00
03B10106	17201023	Egg, chicken, scrambled or omelette, with cheese & salmon or tuna, cooked without added fat	0.00
03B10143	17201024	Egg, chicken, scrambled or omelette, with cheese & vegetables, cooked without added fat	0.00
03B10110	17201025	Egg, chicken, scrambled or omelette, with cheese & vegetables, cooked with added fat	0.00
03B10149	17201026	Egg, chicken, scrambled or omelette, with chicken & vegetables, cooked with added fat	0.00
03A10103	17201027	Egg, chicken, scrambled or omelette, with herbs, cooked without added fat	0.00
03B10087	17201028	Egg, chicken, scrambled or omelette, with herbs, cooked with added fat	0.00
03B10175	17201029	Egg, chicken, scrambled or omelette, with meat & vegetables, cooked with added fat	0.00
03B10153	17201030	Egg, chicken, scrambled or omelette, with salmon or tuna, cooked without added fat	0.00
03B10170	17201031	Egg, chicken, scrambled or omelette, with salmon or tuna & vegetables, cooked without added fat	0.00
03B10156	17201032	Egg, chicken, scrambled or omelette, with salmon or tuna & vegetables, cooked with added fat	0.00
03B10115	17201033	Egg, chicken, scrambled or omelette, with vegetables, fat not further defined	0.00
03B10171	17201034	Egg, chicken, scrambled or omelette, with vegetables, cooked without added fat	0.00
03B10092	17201037	Eggs benedict, poached chicken egg, with bacon or ham & hollandaise sauce	0.00
03B10179	17201038	Eggs benedict, poached chicken egg, bacon or ham & hollandaise sauce, with bread or English muffin	0.00
03B10135	17201039	Eggs benedict, poached chicken egg, with bacon or ham, spinach & hollandaise sauce	0.00
03B10094	17201040	Eggs benedict, poached chicken egg, with smoked salmon, spinach & hollandaise sauce	0.00
03B10180	17201041	Eggs benedict, poached chicken egg, with smoked salmon, spinach & hollandaise sauce, with bread or English muffin	0.00
03B10080	17201052	Omelette, chicken egg, no added fat	0.00
03B10083	17201053	Omelette, chicken egg, cooked with butter or dairy blend	0.00
03B10169	17201054	Omelette, chicken egg, cooked with margarine spread	0.00
03B10082	17201055	Omelette, chicken egg, cooked with oil not further defined	0.00
03B10084	17201056	Omelette, chicken egg, cooked with fat not further defined	0.00

03A20005	17201057	Omelette, prepared from yolk-free egg mix, fried, butter	0.00
06D10506	16101001	Apple, bonza, unpeeled, raw	0.00
06D10500	16802001	Apple, dried	0.00
06D10505	16101002	Apple, fuji, unpeeled, raw	0.00
06D10502	16101003	Apple, golden delicious, unpeeled, raw	0.00
06D10503	16101004	Apple, granny-smith, unpeeled, raw	0.00
06D10522	16101005	Apple, granny-smith, unpeeled, baked, no added fat	0.00
06D10504	16101006	Apple, jonathon, unpeeled, raw	0.00
06D10507	16101007	Apple, pink lady, unpeeled, raw	0.00
06D10501	16101008	Apple, red delicious, unpeeled, raw	0.00
06D10530	16101009	Apple, royal gala, unpeeled, raw	0.00
06D10537	16101010	Apple, green skin, peeled, raw	0.00
06D10509	16101011	Apple, green skin, unpeeled, raw	0.00
06D10536	16101012	Apple, red skin, peeled, raw	0.00
06D10508	16101013	Apple, red skin, unpeeled, raw	0.00
06D10538	16101014	Apple, peeled, raw, not further defined	0.00
06E10102	16101018	Apple, peeled, stewed, sugar sweetened, no added fat	0.00
06D10533	16101015	Apple, unpeeled, raw, not further defined	0.00
06E10099	16101019	Apple, unpeeled, stewed, sugar added, no fat added	0.00
06D10559	16101016	Apple, raw, not further defined	0.00
06D10541	16101017	Apple, baked, no added fat	0.00
06D10560	16102001	Apple, canned or puree	0.00
06D10565	16901001	Apple, toffee coated	0.00
06C10203	16403001	Apricot, raw	0.00
06C10228	16802002	Apricot, dried	0.00
06C10220	16404001	Apricot, canned in pear juice	0.00
06C10205	16404002	Apricot, canned in pear juice, drained	0.00
06C10222	16404004	Apricot, canned in light syrup	0.00
06C10206	16404005	Apricot, canned in light syrup, drained	0.00
06C10221	16404007	Apricot, canned in syrup	0.00
06C10207	16404008	Apricot, canned in syrup, drained	0.00
06C10219	16404010	Apricot, canned in intense sweetened liquid	0.00
06C10204	16404011	Apricot, canned in intense sweetened liquid, drained	0.00
06C20041	16404003	Apricot, canned in pear juice, juice only	0.00
06C20043	16404006	Apricot, canned in light syrup, syrup only	0.00
06C20042	16404009	Apricot, canned in syrup, syrup only	0.00
06C20040	16404012	Apricot, canned in intense sweetened liquid, liquid only	0.00
06E10156	16403002	Apricot, stewed, sugar sweetened, no added fat	0.00
06D10493	16503001	Babaco, peeled, raw	0.00
06D10517	16501001	Banana, cavendish, peeled, raw	0.00
06D10518	16501002	Banana, lady finger or sugar, peeled, raw	0.00
06D10564	16501003	Banana, cooked	0.00
06D10549	16802003	Banana chip	0.00
06A10110	16201001	Blackberry, raw	0.00
06A10116	16201002	Blackberry, purchased frozen	0.00

06A10108	16201003	Blueberry, raw	0.00
06A10115	16201004	Blueberry, purchased frozen	0.00
06A10113	16202001	Blueberry, canned in syrup	0.00
06A10106	16202002	Blueberry, canned in syrup, drained	0.00
06A20015	16202003	Blueberry, canned in syrup, syrup only	0.00
06C10202	16403003	Cherry, raw	0.00
06C10218	16404013	Cherry, black, canned in syrup	0.00
06C10201	16404014	Cherry, black, canned in syrup, drained	0.00
06C20039	16404015	Cherry, black, canned in syrup, syrup only	0.00
06C10238	16802004	Cherry, dried	0.00
12B10081	16804001	Cherry, glace or maraschino	0.00
06A10124	16201005	Cranberry, raw	0.00
06A10107	16802005	Cranberry, dried, sweetened	0.00
06B10097	16303001	Cumquat (kumquat), raw	0.00
06D10499	16801001	Currant, dried	0.00
06D10496	16504001	Custard apple, african pride, peeled, raw	0.00
06C10211	16802006	Date, dried	0.00
06D10570	16504002	Feijoa, raw	0.00
06D10471	16802007	Fig, dried	0.00
06D10470	16503003	Fig, fresh, peeled or unpeeled, raw	0.00
06D10572	16503004	Fig, fresh, peeled or unpeeled, baked	0.00
06E10137	16701001	Fruit salad, apple, banana & berries, homemade	0.00
06E10106	16701002	Fruit salad, apple, banana & mango, homemade	0.00
06E10109	16701003	Fruit salad, apple, banana & melon, homemade	0.00
06E10114	16701004	Fruit salad, apple, banana & orange, homemade	0.00
06E10110	16701005	Fruit salad, apple, banana & stone fruit, homemade	0.00
06E10107	16701006	Fruit salad, apple, banana, berries & other fruit, homemade	0.00
06E10105	16701007	Fruit salad, apple, banana, kiwifruit & other fruit, homemade	0.00
06E10112	16701008	Fruit salad, apple, banana, stone fruit & other fruit homemade	0.00
06E10116	16701009	Fruit salad, apple, berries & orange, homemade	0.00
06E10118	16701010	Fruit salad, apple, melon & orange, homemade	0.00
06E10151	16701011	Fruit salad, apple, melon & stone fruit, homemade	0.00
06E10154	16701012	Fruit salad, banana, berries, melon & other fruit, homemade	0.00
06E10125	16701013	Fruit salad, banana, berries & stone fruit, homemade	0.00
06E10123	16701014	Fruit salad, banana, berries, mango & other fruit, homemade	0.00
06E10124	16701015	Fruit salad, banana, kiwifruit & melon, homemade	0.00
06E10141	16701016	Fruit salad, banana, melon & pineapple, homemade	0.00
06E10139	16701017	Fruit salad, berries & pineapple, homemade	0.00
06E10143	16701018	Fruit salad, berries, cherries, watermelon or other red fruit, homemade	0.00
06E10129	16701019	Fruit salad, berries, mango, stone fruit & other fruit, homemade	0.00

06E10142	16701020	Fruit salad, grape & melon, homemade	0.00
06E10132	16701021	Fruit salad, kiwifruit, melon & orange, homemade	0.00
06E10155	16701022	Fruit salad, melon, pineapple & stone fruit, homemade	0.00
06E10135	16701023	Fruit salad, fresh, tropical style, commercial	0.00
06E10138	16701024	Fruit salad, fresh, tropical style (banana, mango, papaya and other fruits), homemade	0.00
06E10150	16702001	Fruit salad, tropical, canned, drained or undrained	0.00
06E10153	16701025	Fruit salad, fresh fruit & maraschino cherry, homemade or commercial	0.00
06E10152	16701026	Fruit salad, fresh & dried fruit, homemade or commercial	0.00
06E10092	16702008	Fruit salad, canned in heavy syrup	0.00
06E10087	16702009	Fruit salad, canned in heavy syrup, drained	0.00
06E20017	16702010	Fruit salad, canned in heavy syrup, syrup only	0.00
06E10094	16702002	Fruit salad, canned in pear juice	0.00
06E10089	16702003	Fruit salad, canned in pear juice, drained	0.00
06E20019	16702004	Fruit salad, canned in pear juice, juice only	0.00
06E10095	16702005	Fruit salad, canned in pineapple juice	0.00
06E10090	16702006	Fruit salad, canned in pineapple juice, drained	0.00
06E20020	16702007	Fruit salad, canned in pineapple juice, juice only	0.00
06E10093	16702011	Fruit salad, canned in syrup	0.00
06E10088	16702012	Fruit salad, canned in syrup, drained	0.00
06E20018	16702013	Fruit salad, canned in syrup, syrup only	0.00
06E10103	16702014	Fruit salad, canned, not further defined	0.00
06E10096	16701027	Fruit salad, commercial, fresh	0.00
06E10136	16701028	Fruit salad, homemade, fresh, not further defined	0.00
06D10568	16701029	Fruit, fresh, not further defined	0.00
06E10158	16701030	Fruit, puree or stewed, not further defined	0.00
13A12593	16701032	Fruit, for use in garden salad recipes	0.00
06D10561	16802008	Goji berry, dried	0.00
06D10472	16601001	Grape, black muscatel, raw	0.00
06D10475	16601002	Grape, black sultana, raw	0.00
06D10473	16601003	Grape, cornichon, raw	0.00
06D10477	16601004	Grape, red globe, raw	0.00
06D10476	16601005	Grape, thompson seedless or sultana, raw	0.00
06D10474	16601006	Grape, waltham cross, raw	0.00
06D10540	16601007	Grape, raw, not further defined	0.00
06B10079	16303002	Grapefruit, peeled, raw	0.00
06D10487	16504003	Guava, hawaiian, raw	0.00
06D10490	16504004	Jackfruit, peeled, raw	0.00
06D10495	16601009	Kiwifruit, green (hayward), peeled, raw	0.00
06D10553	16601010	Kiwifruit, green (hayward), unpeeled, raw	0.00
06D10531	16601008	Kiwifruit, gold, peeled or unpeeled, raw	0.00
06B10091	16302002	Lemon peel, raw	0.00
06B10080	16302001	Lemon, peeled, raw	0.00
06E10159	16804002	Lemon, preserved	0.00
06B10084	16302003	Lime, peeled, raw	0.00

06D10481	16105001	Loquat, peeled, raw	0.00
06D10482	16504005	Lychee, peeled, raw	0.00
06D10569	16505001	Lychee, peeled, canned	0.00
06B10081	16303003	Mandarin, peeled, raw	0.00
06B10083	16303004	Tangelo, peeled, raw	0.00
06B10082	16303005	Tangerine or tangor, peeled, raw	0.00
06B10092	16304002	Mandarin, canned in syrup	0.00
06B10089	16304003	Mandarin, canned in syrup, drained	0.00
06B20005	16304004	Mandarin, canned in syrup, syrup only	0.00
06B10096	16304001	Mandarin, canned in natural juice	0.00
06D10478	16504006	Mango, peeled, raw	0.00
06D10534	16505002	Mango, pulped, canned	0.00
06D10488	16601011	Melon, honey dew, white skin, peeled, raw	0.00
06D10489	16601012	Melon, honey dew, yellow skin, peeled, raw	0.00
06D10532	16601013	Melon, honey dew, skin not further defined, peeled raw	0.00
06D10497	16601014	Melon, rockmelon (cantaloupe), peeled, raw	0.00
06D10498	16601015	Melon, watermelon, peeled, raw	0.00
06D10555	16601016	Melon, peeled, raw, not further defined	0.00
06D10566	16201007	Mixed berry, cooked	0.00
06A10119	16802009	Mixed berry, dried	0.00
06A10122	16202004	Mixed berry, puree	0.00
06A10118	16201006	Mixed berry, raw, not further defined	0.00
06E10091	16802010	Mixed dried fruit	0.00
06E10133	16802011	Mixed dried fruit, for use in muesli recipes	0.00
06E10134	16802012	Mixed dried fruit, for use in muesli bar recipes	0.00
06D10542	16702015	Mixed fruit, pear & peach, canned in pear juice, drained or undrained	0.00
06E10144	16701031	Mixed fruit platter, not further defined	0.00
06A10102	16201008	Mulberry, raw	0.00
06C10235	16401001	Nectarine, yellow, peeled, raw	0.00
06C10209	16401002	Nectarine, yellow, unpeeled, raw	0.00
06C10229	16401003	Nectarine, white, peeled or unpeeled, raw	0.00
06E10157	16401004	Nectarine, stewed, sugar sweetened, no added fat	0.00
06B10090	16301003	Orange, peeled, raw, not further defined	0.00
06E10146	16301004	Orange, peeled, stewed, no added fat	0.00
06B10095	16301001	Orange, navel (all varieties), peeled, raw	0.00
06B10088	16301002	Orange, valencia, peeled, raw	0.00
06D10479	16504007	Passionfruit, raw	0.00
06D10525	16505003	Passionfruit, pulp, canned	0.00
06D10480	16504008	Pawpaw (papaya), orange flesh, peeled, raw	0.00
06C10231	16401005	Peach, yellow, peeled, raw	0.00
06C10212	16401006	Peach, yellow, unpeeled, raw	0.00
06C10232	16401007	Peach, white, peeled, raw	0.00
06C10230	16401008	Peach, white, unpeeled, raw	0.00
06C10233	16401009	Peach, peeled, raw, not further defined	0.00
06C10234	16401010	Peach, unpeeled, raw, not further defined	0.00

06E10149	16401011	Peach, stewed, sugar sweetened, no added fat	0.00
06C10227	16402001	Peach, canned in pear juice	0.00
06C10216	16402002	Peach, canned in pear juice, drained	0.00
06C10226	16402004	Peach, canned in light syrup	0.00
06C10215	16402005	Peach, canned in light syrup, drained	0.00
06C10225	16402007	Peach, canned in syrup	0.00
06C10214	16402008	Peach, canned in syrup, drained	0.00
06C10223	16402010	Peach, canned in intense sweetened liquid	0.00
06C10208	16402011	Peach, canned in intense sweetened liquid, drained	0.00
06C20048	16402003	Peach, canned in pear juice, juice only	0.00
06C20047	16402006	Peach, canned in light syrup, syrup only	0.00
06C20046	16402009	Peach, canned in syrup, syrup only	0.00
06C20044	16402012	Peach, canned in intense sweetened liquid, liquid only	0.00
06C10236	16402013	Peach, canned, not further defined	0.00
06D10545	16103003	Pear, green, peeled, raw	0.00
06D10543	16103004	Pear, green, unpeeled, raw	0.00
06D10544	16103001	Pear, brown, peeled, raw	0.00
06D10510	16103002	Pear, brown, unpeeled, raw	0.00
06D10511	16103006	Pear, packhams triumph, unpeeled, raw	0.00
06D10512	16103007	Pear, william bartlett, unpeeled, raw	0.00
06D10546	16103008	Pear, peeled, raw, not further defined	0.00
06D10513	16103009	Pear, unpeeled, raw, not further defined	0.00
06D10547	16103010	Pear, raw, not further defined	0.00
06D10529	16104001	Pear, canned in pear juice	0.00
06D10520	16104002	Pear, canned in pear juice, drained	0.00
06D10527	16104004	Pear, canned in light syrup	0.00
06D10515	16104005	Pear, canned in light syrup, drained	0.00
06D10526	16104007	Pear, canned in syrup	0.00
06D10514	16104008	Pear, canned in syrup, drained	0.00
06D10528	16104010	Pear, canned in intense sweetened liquid	0.00
06D10516	16104011	Pear, canned in intense sweetened liquid, drained	0.00
06D20033	16104003	Pear, canned in pear juice, juice only	0.00
06D20032	16104006	Pear, canned in light syrup, syrup only	0.00
06D20031	16104009	Pear, canned in sugar syrup, syrup only	0.00
06D20030	16104012	Pear, canned in intense sweetened liquid, liquid only	0.00
06D10548	16104013	Pear, canned, not further defined	0.00
06E10104	16103011	Pear, stewed, sugar sweetened, no added fat	0.00
06D10521	16103005	Pear, nashi, peeled or unpeeled, raw	0.00
06D10485	16601017	Pepino, peeled, raw	0.00
06D10484	16503005	Persimmon, peeled, raw	0.00
06D10468	16502001	Pineapple (cayenne), peeled, raw	0.00
06D10469	16505004	Pineapple, fresh, cooked in light syrup, drained	0.00
06D10465	16505005	Pineapple, canned in pineapple juice	0.00
06D10464	16505006	Pineapple, canned in pineapple juice, drained	0.00
06D10467	16505008	Pineapple, canned in syrup	0.00
06D10466	16505009	Pineapple, canned in syrup, drained	0.00

06D20028	16505007	Pineapple, canned in pineapple juice, juice only	0.00
06D20029	16505010	Pineapple, canned in syrup, syrup only	0.00
06C10217	16403004	Plum, unpeeled, raw	0.00
06E10101	16403005	Plum, stewed, sugar sweetened, no added fat	0.00
06C10224	16404016	Plum, dark, canned in syrup	0.00
06C10213	16404017	Plum, dark, canned in syrup, drained	0.00
06C20045	16404018	Plum, dark, canned in syrup, syrup only	0.00
06D10562	16101020	Pome fruit, raw, not further defined	0.00
06D10494	16504009	Pomegranate, peeled, raw	0.00
06D10491	16504010	Prickly pear, peeled, raw	0.00
06C10210	16802013	Prune (dried plum)	0.00
06D10523	16105002	Quince, peeled, cooked, sweetened	0.00
06D10552	16801002	Raisin	0.00
06D10483	16504011	Rambutan, raw	0.00
06A10109	16201009	Raspberry, raw	0.00
06A10117	16201010	Raspberry, purchased frozen	0.00
06A10111	16202005	Raspberry, canned in syrup	0.00
06A10104	16202006	Raspberry, canned in syrup, drained	0.00
06A20013	16202007	Raspberry, canned in syrup, syrup only	0.00
06D10524	16601018	Rhubarb, stalk, raw	0.00
06E10100	16601019	Rhubarb, stewed, sugar sweetened, no added fat	0.00
06C10237	16403006	Stone fruit, peeled or unpeeled, raw, not further defined	0.00
06A10103	16201011	Strawberry, raw	0.00
06A10114	16201012	Strawberry, purchased frozen	0.00
06A10112	16202008	Strawberry, canned in syrup	0.00
06A10105	16202009	Strawberry, canned in syrup, drained	0.00
06A20014	16202010	Strawberry, canned in syrup, syrup only	0.00
06A10125	16901007	Strawberry, chocolate coated	0.00
06D10519	16801003	Sultana	0.00
13A20140	16804004	Tamarind, paste, pure, raw	0.00
06D10486	16503006	Tamarillo, peeled, raw	0.00
06D10571	16504012	Tropical fruit, other, raw	0.00
06D10563	16802014	Tropical fruit, dried	0.00
06D10492	16503007	Wax jambu, raw	0.00
07D10164	32301001	Fruit, puree or stewed, infant	0.00
06A10126	16503002	Cheese fruit, raw	0.00
15A10920	24403005	Flower, rosella (native)	0.00
15A10940	16001001	Fruit, wild harvested, raw	0.00
15A10917	16302004	Lime, native, fruit	0.00
15A10936	24001001	Nutgrass (nut grass), peeled, raw	0.00
11B10261	22204023	Pandanus kernel	0.00
15A10919	16601020	Plum, davidson (native), flesh	0.00
15A10918	16601021	Quandong, fruit, flesh	0.00
15A10937	16001002	Saltbush, ruby, fruit	0.00
06C10239	16804003	Plum, salted	0.00
15A10916	22101009	Wattle seed (acacia), ground	0.00

13B20258	25201004	Baked beans, canned in BBQ sauce, regular	0.00
13B20229	25201005	Baked beans, canned in tomato sauce, regular	0.00
13B20233	25201006	Baked beans, canned in tomato sauce, reduced salt	0.00
13B20277	25201009	Baked beans, all flavours (except tomato sauce), reduced salt	0.00
13B20257	25201007	Baked beans, canned in tomato & cheese sauce, regular	0.00
13B20278	25201008	Baked beans, canned in tomato sauce, with sausages, regular	0.00
13B20288	25201010	Baked beans, canned, not further defined	0.00
13A20136	25102002	Bean, cannellini, canned, drained	0.00
13A20122	25101002	Bean, haricot, dried	0.00
13A20123	25101003	Bean, haricot, dried, boiled, microwaved or steamed, drained	0.00
13A20127	25101004	Bean, lima, dried	0.00
13A20128	25101005	Bean, lima, dried, boiled, microwaved or steamed, drained	0.00
13A20138	25102004	Bean, mixed, canned, drained	0.00
13A20124	25101007	Bean, red kidney, dried	0.00
13A20125	25101008	Bean, red kidney, dried, boiled, microwaved or steamed, drained	0.00
13A20126	25102005	Bean, red, kidney, canned, drained	0.00
13A20129	25101009	Bean, soya, dried	0.00
13A20130	25101010	Bean, soya, dried, boiled, microwaved or steamed, drained	0.00
13A20131	25102007	Bean, soya, canned, drained	0.00
13B20231	25102006	Bean, refried, canned	0.00
13A20146	25101011	Chickpea, dried, boiled, microwaved or steamed, drained	0.00
13A20137	25102008	Chickpea, canned, drained	0.00
13A20145	25101020	Legumes, mixed, for use in homemade soup recipes	0.00
13A20134	25101012	Lentil, dried	0.00
13A20135	25101013	Lentil, dried, cooked	0.00
13A20142	25101014	Lentil, green or brown, dried	0.00
13A20144	25101015	Lentil, green or brown, cooked	0.00
13A20141	25101016	Lentil, red, dried	0.00
13A20143	25101017	Lentil, red, cooked	0.00
13A20132	25101018	Pea, split, dried	0.00
13A20133	25101019	Pea, split, dried, boiled, microwaved or steamed, drained	0.00
08A11294	18101001	Beef, for use in kebabs, cooked	0.00
08A11037	18101002	Beef, all cuts, separable fat, raw	0.00
08A11038	18101003	Beef, all cuts, separable fat, grilled or roasted without fat	0.00
08A11082	18101004	Beef, BBQ/grill/fry cuts, fully-trimmed, raw	0.00
08A11083	18101005	Beef, BBQ/grill/fry cuts, fully-trimmed, BBQ'd, grilled or fried, no added fat	0.00
08A11084	18101006	Beef, BBQ/grill/fry cuts, semi-trimmed, raw	0.00

08A11085	18101007	Beef, BBQ/grill/fry cuts, semi-trimmed, BBQ'd, grilled or fried, no added fat	0.00
08A10950	18101020	Beef, blade steak, untrimmed, raw	0.00
08A11060	18101008	Beef, blade steak, separable fat, raw	0.00
08A11066	18101009	Beef, blade steak, separable fat, grilled or BBQ'd, no added fat	0.00
08A10948	18101010	Beef, blade steak, separable lean, raw	0.00
08A10951	18101011	Beef, blade steak, separable lean, grilled or BBQ'd, no added fat	0.00
08A11054	18101012	Beef, blade steak, fully-trimmed, raw	0.00
08A10952	18101013	Beef, blade steak, fully-trimmed, baked, roasted, fried, grilled or BBQ'd, no added fat	0.00
08A11264	18101014	Beef, blade steak, fully-trimmed, baked, roasted, fried, grilled or BBQ'd, fat not further defined	0.00
08A11239	18101015	Beef, blade steak, fully-trimmed, boiled, casseroled, microwaved, poached, steamed or stewed, no added fat	0.00
08A11285	18101016	Beef, blade steak, fully-trimmed, boiled, casseroled, microwaved, poached, steamed or stewed, fat not further defined	0.00
08A10949	18101017	Beef, blade steak, semi-trimmed, raw	0.00
08A10953	18101018	Beef, blade steak, semi-trimmed, baked, roasted, fried, grilled or BBQ'd, no added fat	0.00
08A11139	18101019	Beef, blade steak, semi-trimmed, baked, roasted, fried, grilled or BBQ'd, fat not further defined	0.00
08A10954	18101021	Beef, blade steak, untrimmed, baked, roasted, fried, grilled or BBQ'd, no added fat	0.00
08A11214	18101022	Beef, blade steak, untrimmed, baked, roasted, fried, grilled or BBQ'd, fat not further defined	0.00
08A11295	18101023	Beef, blade steak, untrimmed, boiled, casseroled, microwaved, poached, steamed or stewed, with or without added fat	0.00
08A11072	18101024	Beef, casserole cuts, fully-trimmed, raw	0.00
08A11073	18101025	Beef, casserole cuts, fully-trimmed, boiled, casseroled, microwaved, poached, steamed, or stewed, no added fat	0.00
08A11234	18101026	Beef, casserole cuts, untrimmed, raw	0.00
08A11326	18101027	Beef, casserole cuts, untrimmed, boiled, casseroled, microwaved, poached, steamed or stewed, with or without fat	0.00
08A11067	18101028	Beef, chuck steak, separable fat, raw	0.00
08A11068	18101029	Beef, chuck steak, separable fat, boiled, casseroled, microwaved, poached, steamed, or stewed, no added fat	0.00
08A10955	18101030	Beef, chuck steak, separable lean, raw	0.00
08A10958	18101031	Beef, chuck steak, separable lean, boiled, casseroled, microwaved, poached, steamed, or stewed, no added fat	0.00
08A10956	18101032	Beef, chuck steak, fully-trimmed, raw	0.00

08A11286	18101033	Beef, chuck steak, fully-trimmed, baked, roasted, fried, stir-fried, grilled or BBQ'd, no added fat	0.00
08A11225	18101034	Beef, chuck steak, fully-trimmed, baked, roasted, fried, stir-fried, grilled or BBQ'd, fat not further defined	0.00
08A10959	18101035	Beef, chuck steak, fully-trimmed, boiled, casseroled, microwaved, poached, steamed, or stewed, with or without added fat	0.00
08A10957	18101036	Beef, chuck steak, untrimmed, raw	0.00
08A10960	18101037	Beef, chuck steak, untrimmed, boiled, casseroled, microwaved, poached, steamed, or stewed, with or without added fat	0.00
08A10961	18101038	Beef, diced, separable lean, raw	0.00
08A10963	18101039	Beef, diced, separable lean, fried or stir-fried, no added fat	0.00
08A11048	18101040	Beef, diced, fully-trimmed, raw	0.00
08A10964	18101041	Beef, diced, fully-trimmed, baked, roasted, fried or stir-fried, grilled or BBQ'd, no added fat	0.00
08A11250	18101042	Beef, diced, fully-trimmed, baked, roasted, fried or stir-fried, grilled or BBQ'd, fat not further defined	0.00
08A11240	18101043	Beef, diced, fully-trimmed, boiled, casseroled, microwaved, poached, steamed or stewed, no added fat	0.00
08A11252	18101044	Beef, diced, fully-trimmed, boiled, casseroled, microwaved, poached, steamed or stewed, fat not further defined	0.00
08A10962	18101045	Beef, diced, untrimmed, raw	0.00
08A10965	18101046	Beef, diced, untrimmed, baked, roasted, fried or stir-fried, grilled or BBQ'd, no added fat	0.00
08A11050	18101047	Beef, eye fillet, separable lean, raw	0.00
08A10966	18101048	Beef, eye fillet, separable lean, baked, roasted, fried, grilled or BBQ'd, no added fat	0.00
08A10967	18101049	Beef, fillet steak, separable lean, raw	0.00
08A10969	18101050	Beef, fillet steak, separable lean, grilled or BBQ'd, no added fat	0.00
08A11049	18101051	Beef, fillet steak, fully-trimmed, raw	0.00
08A10970	18101052	Beef, fillet steak, fully-trimmed, baked, roasted, fried, grilled or BBQ'd, no added fat	0.00
08A11318	18101053	Beef, fillet steak, fully-trimmed, baked, roasted, fried, grilled or BBQ'd, butter, dairy blend or margarine	0.00
08A11190	18101054	Beef, fillet steak, fully-trimmed, baked, roasted, fried, grilled or BBQ'd, canola oil	0.00
08A11173	18101055	Beef, fillet steak, fully-trimmed, baked, roasted, fried, grilled or BBQ'd, olive oil	0.00
08A11287	18101056	Beef, fillet steak, fully-trimmed, baked, roasted, fried, grilled or BBQ'd, other oil	0.00
08A11178	18101057	Beef, fillet steak, fully-trimmed, baked, roasted, fried, grilled or BBQ'd, fat not further defined	0.00
08A10968	18101058	Beef, fillet steak, untrimmed, raw	0.00

08A10972	18101059	Beef, fillet steak, untrimmed, baked, roasted, fried, grilled or BBQ'd, no added fat	0.00
08A11267	18101060	Beef, fillet steak, untrimmed, baked, roasted, fried, grilled or BBQ'd, fat not further defined	0.00
08A11329	18101061	Beef, kebab, marinated, baked, roasted, fried, grilled or BBQ'd, fat not further defined	0.00
08A11059	18101062	Beef, loin (fillet, sirloin, scotch fillet, T-bone), separable fat, raw	0.00
08A11065	18101063	Beef, loin (fillet, sirloin, scotch fillet, T-bone), separable fat, grilled or BBQ'd, no added fat	0.00
08A11086	18101064	Beef, mince, <5% fat, raw	0.00
08A11089	18101065	Beef, mince, <5% fat, baked, roasted, fried or stir-fried, grilled or BBQ'd, no added fat	0.00
08A11123	18101066	Beef, mince, <5% fat, baked, roasted, fried or stir-fried, grilled or BBQ'd, canola oil	0.00
08A11122	18101067	Beef, mince, <5% fat, baked, roasted, fried or stir-fried, grilled or BBQ'd, olive oil	0.00
08A11125	18101068	Beef, mince, <5% fat, baked, roasted, fried or stir-fried, grilled or BBQ'd, other oil	0.00
08A11124	18101069	Beef, mince, <5% fat, baked, roasted, fried or stir-fried, grilled or BBQ'd, fat not further defined	0.00
08A11096	18101070	Beef, mince, <5% fat, boiled, casseroled, microwaved, poached, steamed, or stewed, no added fat	0.00
08A11259	18101071	Beef, mince, <5% fat, boiled, casseroled, microwaved, poached, steamed or stewed, fat not further defined	0.00
08A11087	18101072	Beef, mince, ~5-10% fat, raw	0.00
08A11090	18101073	Beef, mince, ~5-10% fat, baked, roasted, fried or stir-fried, grilled or BBQ'd, no added fat	0.00
08A11127	18101074	Beef, mince, ~5-10% fat, baked, roasted, fried or stir-fried, grilled or BBQ'd, canola oil	0.00
08A11126	18101075	Beef, mince, ~5-10% fat, baked, roasted, fried or stir-fried, grilled or BBQ'd, olive oil	0.00
08A11129	18101076	Beef, mince, ~5-10% fat, baked, roasted, fried or stir-fried, grilled or BBQ'd, other oil	0.00
08A11128	18101077	Beef, mince, ~5-10% fat, baked, roasted, fried or stir-fried, grilled or BBQ'd, fat not further defined	0.00
08A11193	18101078	Beef, mince, ~5-10% fat, boiled, casseroled, microwaved, poached, steamed, or stewed, no added fat	0.00
08A11143	18101079	Beef, mince, ~5-10% fat, boiled, casseroled, microwaved, poached, steamed, or stewed, fat not further defined	0.00
08A11088	18101080	Beef, mince, >10% fat, raw	0.00
08A11091	18101081	Beef, mince, >10% fat, baked, roasted, fried or stir-fried, grilled or BBQ'd, no added fat	0.00
08A11132	18101082	Beef, mince, >10% fat, baked, roasted, fried or stir-fried, grilled or BBQ'd, fat not further defined	0.00
08A11135	18101083	Beef, mince, raw, not further defined	0.00

08A11282	18101084	Beef, mince, baked, roasted, fried or stir-fried, grilled or BBQ'd, no added fat	0.00
08A11281	18101085	Beef, mince, baked, roasted, fried or stir-fried, grilled or BBQ'd, fat not further defined	0.00
08A11291	18101086	Beef, mince, boiled, casseroled, microwaved, steamed or stewed, with or without added fat	0.00
08A11109	18101087	Beef, rib cutlet or roast, fully-trimmed, baked, roasted, fried, grilled or BBQ'd, no added fat	0.00
08A11110	18101088	Beef, rib cutlet or roast, fully-trimmed, baked, roasted, fried, grilled or BBQ'd, fat not further defined	0.00
08A11108	18101089	Beef, rib cutlet or roast, semi-trimmed, boiled, casseroled, microwaved, poached, steamed, or stewed, with or without added fat	0.00
08A11330	18101090	Beef, rib, baked, roasted, fried, grilled or BBQ'd, with or without added fat	0.00
08A11074	18101091	Beef, roasting cuts, fully-trimmed, raw	0.00
08A11075	18101092	Beef, roasting cuts, fully-trimmed, baked or roasted, no added fat	0.00
08A11076	18101093	Beef, roasting cuts, semi-trimmed, raw	0.00
08A11077	18101094	Beef, roasting cuts, semi-trimmed, baked or roasted, no added fat	0.00
08A11233	18101095	Beef, roasting cuts, untrimmed, raw	0.00
08A11245	18101096	Beef, roasting cuts, untrimmed, baked or roasted, no added fat	0.00
08A11045	18101097	Beef, round medallion, separable lean, raw	0.00
08A10973	18101098	Beef, round medallion, separable lean, grilled or BBQ'd, no added fat	0.00
08A11057	18101099	Beef, round steak, separable fat, raw	0.00
08A11063	18101100	Beef, round steak, separable fat, grilled or BBQ'd, no added fat	0.00
08A10974	18101101	Beef, round steak, separable lean, raw	0.00
08A10977	18101102	Beef, round steak, separable lean, grilled or BBQ'd, no added fat	0.00
08A11044	18101103	Beef, round steak, fully-trimmed, raw	0.00
08A10978	18101104	Beef, round steak, fully-trimmed, baked, roasted, fried, grilled or BBQ'd, no added fat	0.00
08A11292	18101105	Beef, round steak, fully-trimmed, baked, roasted, fried, grilled or BBQ'd, canola oil	0.00
08A11229	18101106	Beef, round steak, fully-trimmed, baked, roasted, fried, grilled or BBQ'd, olive oil	0.00
08A11293	18101107	Beef, round steak, fully-trimmed, baked, roasted, fried, grilled or BBQ'd, other oil	0.00
08A11296	18101108	Beef, round steak, fully-trimmed, baked, roasted, fried, grilled or BBQ'd, fat not further defined	0.00
08A11263	18101109	Beef, round steak, fully-trimmed, boiled, casseroled, microwaved, poached, steamed or stewed, with or without added fat	0.00
08A10975	18101110	Beef, round steak, semi-trimmed, raw	0.00

08A10979	18101111	Beef, round steak, semi-trimmed, baked, roasted, fried, grilled or BBQ'd, no added fat	0.00
08A10976	18101112	Beef, round steak, untrimmed, raw	0.00
08A10980	18101113	Beef, round steak, untrimmed, baked, roasted, fried, grilled or BBQ'd, no added fat	0.00
08A11297	18101114	Beef, round steak, untrimmed, baked, roasted, fried, grilled or BBQ'd, fat not further defined	0.00
08A11047	18101115	Beef, rump medallion, separable lean, raw	0.00
08A10981	18101116	Beef, rump medallion, separable lean, grilled or BBQ'd, no added fat	0.00
08A11058	18101117	Beef, rump steak, separable fat, raw	0.00
08A11064	18101118	Beef, rump steak, separable fat, grilled or BBQ'd, no added fat	0.00
08A10982	18101119	Beef, rump steak, separable lean, raw	0.00
08A10985	18101120	Beef, rump steak, separable lean, grilled or BBQ'd, no added fat	0.00
08A11046	18101121	Beef, rump steak, fully-trimmed, raw	0.00
08A10986	18101122	Beef, rump steak, fully-trimmed, baked, roasted, fried, grilled or BBQ'd, no added fat	0.00
08A11216	18101123	Beef, rump steak, fully-trimmed, baked, roasted, fried, grilled or BBQ'd, butter, dairy blend or margarine	0.00
08A11184	18101124	Beef, rump steak, fully-trimmed, baked, roasted, fried, grilled or BBQ'd, canola oil	0.00
08A11177	18101125	Beef, rump steak, fully-trimmed, baked, roasted, fried, grilled or BBQ'd, olive oil	0.00
08A11298	18101126	Beef, rump steak, fully-trimmed, baked, roasted, fried, stir-fried, grilled or BBQ'd, other oil	0.00
08A11228	18101127	Beef, rump steak, fully-trimmed, baked, roasted, fried, stir-fried, grilled or BBQ'd, fat not further defined	0.00
08A11226	18101128	Beef, rump steak, fully-trimmed, boiled, casseroled, microwaved, poached, steamed or stewed, with or without added fat	0.00
08A10983	18101129	Beef, rump steak, semi-trimmed, raw	0.00
08A10987	18101130	Beef, rump steak, semi-trimmed, baked, roasted, fried, grilled or BBQ'd, no added fat	0.00
08A11320	18101131	Beef, rump steak, semi-trimmed, baked, roasted, fried, grilled or BBQ'd, butter, dairy blend or margarine	0.00
08A11181	18101132	Beef, rump steak, semi-trimmed, baked, roasted, fried, grilled or BBQ'd, canola oil	0.00
08A11115	18101133	Beef, rump steak, semi-trimmed, baked, roasted, fried, grilled or BBQ'd, olive oil	0.00
08A11208	18101134	Beef, rump steak, semi-trimmed, baked, roasted, fried, grilled or BBQ'd, other oil	0.00
08A11116	18101135	Beef, rump steak, semi-trimmed, baked, roasted, fried, grilled or BBQ'd, fat not further defined	0.00
08A11092	18101136	Beef, rump steak, semi-trimmed, boiled, casseroled, microwaved, poached, steamed, or stewed, with or without added fat	0.00
08A10984	18101137	Beef, rump steak, untrimmed, raw	0.00

08A10988	18101138	Beef, rump steak, untrimmed, baked, roasted, fried, grilled or BBQ'd, no added fat	0.00
08A11300	18101139	Beef, rump steak, untrimmed, baked, roasted, fried, grilled or BBQ'd, butter, dairy blend or margarine	0.00
08A11147	18101140	Beef, rump steak, untrimmed, baked, roasted, fried, grilled or BBQ'd, canola oil	0.00
08A11174	18101141	Beef, rump steak, untrimmed, baked, roasted, fried, grilled or BBQ'd, olive oil	0.00
08A11246	18101142	Beef, rump steak, untrimmed, baked, roasted, fried, grilled or BBQ'd, other oil	0.00
08A11209	18101143	Beef, rump steak, untrimmed, baked, roasted, fried, grilled or BBQ'd, fat not further defined	0.00
08A11301	18101144	Beef, rump steak, untrimmed, boiled, casseroled, microwaved, poached, steamed or stewed, with or without added fat	0.00
08A10989	18101145	Beef, scotch fillet, separable lean, raw	0.00
08A10992	18101146	Beef, scotch fillet, separable lean, grilled or BBQ'd, no added fat	0.00
08A11053	18101147	Beef, scotch fillet, fully-trimmed, raw	0.00
08A10993	18101148	Beef, scotch fillet, fully-trimmed, baked, roasted, fried, grilled or BBQ'd, no added fat	0.00
08A11235	18101149	Beef, scotch fillet, fully-trimmed, baked, roasted, fried, grilled or BBQ'd, butter, dairy blend or margarine	0.00
08A11164	18101150	Beef, scotch fillet, fully-trimmed, baked, roasted, fried, grilled or BBQ'd, canola oil	0.00
08A11167	18101151	Beef, scotch fillet, fully-trimmed, baked, roasted, fried, grilled or BBQ'd, olive oil	0.00
08A11191	18101152	Beef, scotch fillet, fully-trimmed, baked, roasted, fried, grilled or BBQ'd, other oil	0.00
08A11212	18101153	Beef, scotch fillet, fully-trimmed, baked, roasted, fried, grilled or BBQ'd, fat not further defined	0.00
08A11247	18101154	Beef, scotch fillet, fully-trimmed, boiled, casseroled, microwaved, poached, steamed or stewed, with or without added fat	0.00
08A10990	18101155	Beef, scotch fillet, semi-trimmed, raw	0.00
08A10994	18101156	Beef, scotch fillet, semi-trimmed, baked, roasted, fried, grilled or BBQ'd, no added fat	0.00
08A11236	18101157	Beef, scotch fillet, semi-trimmed, baked, roasted, fried, grilled or BBQ'd, butter, dairy blend or margarine	0.00
08A11093	18101158	Beef, scotch fillet, semi-trimmed, baked, roasted, fried, grilled or BBQ'd, canola oil	0.00
08A11117	18101159	Beef, scotch fillet, semi-trimmed, baked, roasted, fried, grilled or BBQ'd, olive oil	0.00
08A11302	18101160	Beef, scotch fillet, semi-trimmed, baked, roasted, fried, grilled or BBQ'd, other oil	0.00
08A11118	18101161	Beef, scotch fillet, semi-trimmed, baked, roasted, fried, grilled or BBQ'd, fat not further defined	0.00
08A10991	18101162	Beef, scotch fillet, untrimmed, raw	0.00

08A10995	18101163	Beef, scotch fillet, untrimmed, baked, roasted, fried, grilled or BBQ'd, no added fat	0.00
08A11188	18101164	Beef, scotch fillet, untrimmed, baked, roasted, fried, grilled or BBQ'd, fat not further defined	0.00
08A11041	18101165	Beef, silverside minute steak, separable lean, raw	0.00
08A10996	18101166	Beef, silverside minute steak, separable lean, baked or roasted, no added fat	0.00
08A10997	18101167	Beef, silverside roast, separable lean, raw	0.00
08A11000	18101168	Beef, silverside roast, separable lean, baked or roasted, no added fat	0.00
08A11042	18101169	Beef, silverside roast, fully-trimmed, raw	0.00
08A11001	18101170	Beef, silverside roast, fully-trimmed, baked or roasted, no added fat	0.00
08A11136	18101171	Beef, silverside roast, fully-trimmed, boiled, casseroled, microwaved, poached, steamed, or stewed, no added fat	0.00
08A10998	18101172	Beef, silverside roast, semi-trimmed, raw	0.00
08A11002	18101173	Beef, silverside roast, semi-trimmed, baked or roasted, no added fat	0.00
08A11138	18101174	Beef, silverside roast, semi-trimmed, boiled, casseroled, microwaved, poached, steamed, or stewed, no added fat	0.00
08A10999	18101175	Beef, silverside roast, untrimmed, raw	0.00
08A11003	18101176	Beef, silverside roast, untrimmed, baked or roasted, no added fat	0.00
08A11221	18101177	Beef, silverside roast, untrimmed, boiled, casseroled, microwaved, poached, steamed or stewed, no added fat	0.00
08A11004	18101178	Beef, sirloin steak, separable lean, raw	0.00
08A11007	18101179	Beef, sirloin steak, separable lean, grilled or BBQ'd, no added fat	0.00
08A11052	18101180	Beef, sirloin steak, fully-trimmed, raw	0.00
08A11008	18101181	Beef, sirloin steak, fully-trimmed, baked, roasted, fried, grilled or BBQ'd, no added fat	0.00
08A11140	18101182	Beef, sirloin steak, fully-trimmed, baked, roasted, fried, grilled or BBQ'd, canola oil	0.00
08A11185	18101183	Beef, sirloin steak, fully-trimmed, baked, roasted, fried, grilled or BBQ'd, olive oil	0.00
08A11237	18101184	Beef, sirloin steak, fully-trimmed, baked, roasted, fried, grilled or BBQ'd, other oil	0.00
08A11121	18101185	Beef, sirloin steak, fully-trimmed, baked, roasted, fried, grilled or BBQ'd, fat not further defined	0.00
08A11227	18101186	Beef, sirloin steak, fully-trimmed, boiled, casseroled, microwaved, poached, steamed or stewed, with or without added fat	0.00
08A11005	18101187	Beef, sirloin steak, semi-trimmed, raw	0.00
08A11009	18101188	Beef, sirloin steak, semi-trimmed, baked, roasted, fried, grilled or BBQ'd, no added fat	0.00
08A11211	18101189	Beef, sirloin steak, semi-trimmed, baked, roasted, fried, grilled or BBQ'd, canola oil	0.00

08A11144	18101190	Beef, sirloin steak, semi-trimmed, baked, roasted, fried, grilled or BBQ'd, olive oil	0.00
08A11207	18101191	Beef, sirloin steak, semi-trimmed, baked, roasted, fried, grilled or BBQ'd, other oil	0.00
08A11219	18101192	Beef, sirloin steak, semi-trimmed, baked, roasted, fried, grilled or BBQ'd, fat not further defined	0.00
08A11006	18101193	Beef, sirloin steak, untrimmed, raw	0.00
08A11010	18101194	Beef, sirloin steak, untrimmed, baked, roasted, fried, grilled or BBQ'd, no added fat	0.00
08A11146	18101195	Beef, sirloin steak, untrimmed, baked, roasted, fried, grilled or BBQ'd, olive oil	0.00
08A11206	18101196	Beef, sirloin steak, untrimmed, baked, roasted, fried, grilled or BBQ'd, fat not further defined	0.00
08A11056	18101197	Beef, stir-fry strips or diced, separable fat, raw	0.00
08A11062	18101198	Beef, stir-fry strips or diced, separable fat, fried or stir-fried, no added fat	0.00
08A11011	18101199	Beef, stir-fry strips, separable lean, raw	0.00
08A11013	18101200	Beef, stir-fry strips, separable lean, fried or stir-fried, no added fat	0.00
08A11043	18101201	Beef, stir-fry strips, fully-trimmed, raw	0.00
08A11014	18101202	Beef, stir-fry strips, fully-trimmed, baked, roasted, fried, stir-fried, grilled or BBQ'd, no added fat	0.00
08A11241	18101203	Beef, stir-fry strips, fully-trimmed, baked, roasted, fried, stir-fried, grilled or BBQ'd, canola oil	0.00
08A11242	18101204	Beef, stir-fry strips, fully-trimmed, baked, roasted, fried, stir-fried, grilled or BBQ'd, olive oil	0.00
08A11244	18101205	Beef, stir-fry strips, fully-trimmed, baked, roasted, fried, stir-fried, grilled or BBQ'd, other oil	0.00
08A11243	18101206	Beef, stir-fry strips, fully-trimmed, baked, roasted, fried, stir-fried, grilled or BBQ'd, fat not further defined	0.00
08A11210	18101207	Beef, stir-fry strips, fully-trimmed, boiled, casseroled, microwaved, poached, steamed or stewed, no added fat	0.00
08A11306	18101208	Beef, stir-fry strips, fully-trimmed, boiled, casseroled, microwaved, poached, steamed or stewed, fat not further defined	0.00
08A11012	18101209	Beef, stir-fry strips, untrimmed, raw	0.00
08A11015	18101210	Beef, stir-fry strips, untrimmed, baked, roasted, fried, stir-fried, grilled or BBQ'd, no added fat	0.00
08A11078	18101211	Beef, stir-fry cuts, fully-trimmed, raw	0.00
08A11079	18101212	Beef, stir-fry cuts, fully-trimmed, fried or stir-fried, no added fat	0.00
08A11080	18101213	Beef, stir-fry cuts, semi-trimmed, raw	0.00
08A11081	18101214	Beef, stir-fry cuts, semi-trimmed, fried or stir-fried, no added fat	0.00
08A11016	18101215	Beef, T-bone steak, separable lean, raw	0.00
08A11019	18101216	Beef, T-bone steak, separable lean, grilled or BBQ'd, no added fat	0.00

08A11051	18101217	Beef, T-bone steak, fully-trimmed, raw	0.00
08A11020	18101218	Beef, T-bone steak, fully-trimmed, baked, roasted, fried, grilled or BBQ'd, no added fat	0.00
08A11094	18101219	Beef, T-bone steak, fully-trimmed, baked, roasted, fried, grilled or BBQ'd, fat not further defined	0.00
08A11017	18101220	Beef, T-bone steak, semi-trimmed, raw	0.00
08A11021	18101221	Beef, T-bone steak, semi-trimmed, baked, roasted, fried, grilled or BBQ'd, no added fat	0.00
08A11278	18101222	Beef, T-bone steak, semi-trimmed, baked, roasted, fried, grilled or BBQ'd, butter, dairy blend or margarine	0.00
08A11280	18101223	Beef, T-bone steak, semi-trimmed, baked, roasted, fried, grilled or BBQ'd, canola oil	0.00
08A11182	18101224	Beef, T-bone steak, semi-trimmed, baked, roasted, fried, grilled or BBQ'd, olive oil	0.00
08A11186	18101225	Beef, T-bone steak, semi-trimmed, baked, roasted, fried, grilled or BBQ'd, other oil	0.00
08A11279	18101226	Beef, T-bone steak, semi-trimmed, baked, roasted, fried, grilled or BBQ'd, fat not further defined	0.00
08A11018	18101227	Beef, T-bone steak, untrimmed, raw	0.00
08A11022	18101228	Beef, T-bone steak, untrimmed, baked, roasted, fried, grilled or BBQ'd, no added fat	0.00
08A11180	18101229	Beef, T-bone steak, untrimmed, baked, roasted, fried, grilled or BBQ'd, fat not further defined	0.00
08A11055	18101230	Beef, topside or silverside, separable fat, raw	0.00
08A11023	18101231	Beef, topside roast, separable lean, raw	0.00
08A11026	18101232	Beef, topside roast, separable lean, baked or roasted, no added fat	0.00
08A11040	18101233	Beef, topside roast, fully-trimmed, raw	0.00
08A11027	18101234	Beef, topside roast, fully-trimmed, baked or roasted, no added fat	0.00
08A11204	18101235	Beef, topside roast, fully-trimmed, boiled, casseroled, microwaved, poached, steamed or stewed, no added fat	0.00
08A11024	18101236	Beef, topside roast, semi-trimmed, raw	0.00
08A11028	18101237	Beef, topside roast, semi-trimmed, baked or roasted, no added fat	0.00
08A11025	18101238	Beef, topside roast, untrimmed, raw	0.00
08A11029	18101239	Beef, topside roast, untrimmed, baked or roasted, no added fat	0.00
08F11030	18101240	Beef, topside roast, untrimmed, breadcrumb coating, baked or roasted, no added fat	0.00
08A11030	18101241	Beef, topside steak, separable lean, raw	0.00
08A11061	18101242	Beef, topside/silverside, separable fat, baked or roasted, no added fat	0.00
08A11033	18101243	Beef, topside steak, separable lean, baked or roasted, no added fat	0.00
08A11039	18101244	Beef, topside steak, fully-trimmed, raw	0.00
08A11034	18101245	Beef, topside steak, fully-trimmed, baked, roasted, fried, grilled or BBQ'd, no added fat	0.00

08A11095	18101246	Beef, topside steak, fully-trimmed, baked, roasted, fried, grilled or BBQ'd, fat not further defined	0.00
08A11322	18101247	Beef, topside steak, fully-trimmed, boiled, casseroled, microwaved, poached, steamed or stewed, with or without added fat	0.00
08A11031	18101248	Beef, topside steak, semi-trimmed, raw	0.00
08A11035	18101249	Beef, topside steak, semi-trimmed, baked, roasted, fried, grilled or BBQ'd, no added fat	0.00
08A11032	18101250	Beef, topside steak, untrimmed, raw	0.00
08A11036	18101251	Beef, topside steak, untrimmed, baked, roasted, fried, grilled or BBQ'd, no added fat	0.00
08F11018	18703001	Beef, schnitzel, breadcrumb coating, baked, roasted, fried, grilled or BBQ'd, no added fat	0.00
08F11026	18703002	Beef, schnitzel, breadcrumb coating, baked, roasted, fried, grilled or BBQ'd, canola oil	0.00
08F11005	18703003	Beef, schnitzel, breadcrumb coating, baked, roasted, fried, grilled or BBQ'd, olive oil	0.00
08F11027	18703004	Beef, schnitzel, breadcrumb coating, baked, roasted, fried, grilled or BBQ'd, other oil	0.00
08F10927	18703005	Beef, schnitzel, breadcrumb coating, baked, roasted, fried, grilled or BBQ'd, fat not further defined	0.00
08A11223	18101252	Beef, steak, fully-trimmed, raw	0.00
08A11220	18101253	Beef, steak, fully-trimmed, baked, roasted, fried, grilled or BBQ'd, no added fat	0.00
08A11232	18101254	Beef, steak, fully-trimmed, baked, roasted, fried, grilled or BBQ'd, canola oil	0.00
08A11231	18101255	Beef, steak, fully-trimmed, baked, roasted, fried, grilled or BBQ'd, olive oil	0.00
08A11268	18101256	Beef, steak, fully-trimmed, baked, roasted, fried, grilled or BBQ'd, other oil	0.00
08A11230	18101257	Beef, steak, fully-trimmed, baked, roasted, fried, grilled or BBQ'd, fat not further defined	0.00
08A11254	18101258	Beef, steak, fully-trimmed, boiled, casseroled, microwaved, poached, steamed or stewed, no added fat	0.00
08A11304	18101259	Beef, steak, fully-trimmed, boiled, casseroled, microwaved, poached, steamed or stewed, fat not further defined	0.00
08F11022	18703006	Beef, steak, fully-trimmed, coated, baked, roasted, fried, grilled or BBQ'd, no added fat	0.00
08F11023	18703007	Beef, steak, fully-trimmed, coated, baked, roasted, fried, grilled or BBQ'd, fat not further defined	0.00
08A11160	18101260	Beef, steak, semi-trimmed, raw	0.00
08A11270	18101261	Beef, steak, semi-trimmed, baked, roasted, fried, grilled or BBQ'd, no added fat	0.00
08A11271	18101262	Beef, steak, semi-trimmed, baked, roasted, fried, grilled or BBQ'd, canola oil	0.00
08A11194	18101263	Beef, steak, semi-trimmed, baked, roasted, fried, grilled or BBQ'd, olive oil	0.00

08A11272	18101264	Beef, steak, semi-trimmed, baked, roasted, fried, grilled or BBQ'd, other oil	0.00
08A11195	18101265	Beef, steak, semi-trimmed, baked, roasted, fried, grilled or BBQ'd, fat not further defined	0.00
08A11238	18101266	Beef, steak, semi-trimmed, boiled, casseroled, microwaved, poached, steamed or stewed, with or without added fat	0.00
08F11130	18703008	Beef, steak, semi-trimmed, coated, baked, roasted, fried, grilled or BBQ'd, with or without added fat	0.00
08A11192	18101267	Beef, steak, untrimmed	0.00
08A11176	18101268	Beef, steak, untrimmed, baked, roasted, fried, grilled or BBQ'd, no added fat	0.00
08A11273	18101269	Beef, steak, untrimmed, baked, roasted, fried, grilled or BBQ'd, olive oil	0.00
08A11274	18101270	Beef, steak, untrimmed, baked, roasted, fried, grilled or BBQ'd, other oil	0.00
08A11277	18101271	Beef, steak, untrimmed, baked, roasted, fried, grilled or BBQ'd, fat not further defined	0.00
08F11029	18101272	Beef, steak, untrimmed, coated, baked, roasted, fried, grilled or BBQ'd, fat not further defined	0.00
08A11161	18101273	Beef, steak, for use on sandwiches, not further defined	0.00
08A11323	18703012	Meatball or rissole, beef, commercial, cooked	0.00
08A11100	18703013	Meatball or rissole, beef mince, <5% fat, homemade, raw	0.00
08A11149	18703014	Meatball or rissole, beef mince, <5% fat, homemade, baked, roasted, fried, grilled or BBQ'd, no added fat	0.00
08A11197	18703015	Meatball or rissole, beef mince, <5% fat, homemade, baked, roasted, fried, grilled or BBQ'd, fat not further defined	0.00
08A11105	18703016	Meatball or rissole, beef mince, ~5-10% fat, homemade, raw	0.00
08A11157	18703017	Meatball or rissole, beef mince, >10% fat, homemade, raw	0.00
08A11150	18703018	Meatball or rissole, beef mince, homemade, raw	0.00
08A11151	18703019	Meatball or rissole, beef mince, homemade, baked, roasted, fried, grilled or BBQ'd, no added fat	0.00
08A11159	18703020	Meatball or rissole, beef mince, homemade, baked, roasted, fried, grilled or BBQ'd, fat not further defined	0.00
08A11134	18703022	Rissole or patty, beef mince, grilled or fried, no added fat, fast food style	0.00
08A20749	18102001	Lamb, all cuts, separable fat, raw	0.00
08A20750	18102002	Lamb, all cuts, separable fat, cooked	0.00
08A20801	18102003	Lamb, BBQ/grill/fry cuts, fully-trimmed, raw	0.00
08A20802	18102004	Lamb, BBQ/grill/fry cuts, fully-trimmed, BBQ'd, grilled or fried, no added fat	0.00
08A20803	18102005	Lamb, BBQ/grill/fry cuts, semi-trimmed, raw	0.00
08A20804	18102006	Lamb, BBQ/grill/fry cuts, semi-trimmed, BBQ'd, grilled or fried, no added fat	0.00
08A20766	18102007	Lamb, butterfly steak, separable lean, raw	0.00

08A20819	18102008	Lamb, butterfly steak, fully-trimmed, raw	0.00
08A20664	18102009	Lamb, butterfly steak, fully-trimmed, baked, roasted, fried, grilled or BBQ'd, no added fat	0.00
08A20820	18102010	Lamb, butterfly steak, fully-trimmed, boiled, casseroled, microwaved, poached, steamed, or stewed, no added fat	0.00
08A20793	18102011	Lamb, casserole cuts, fully-trimmed, raw	0.00
08A20794	18102012	Lamb, casserole cuts, fully-trimmed, boiled, casseroled, microwaved, poached, steamed, or stewed, no added fat	0.00
08A20844	18102013	Lamb, casserole cuts, semi-trimmed, boiled, casseroled, microwaved, poached, steamed or stewed, no added fat	0.00
08A20831	18102014	Lamb, chop, fully-trimmed, raw, not further defined	0.00
08A20838	18102015	Lamb, chop, fully-trimmed, baked, roasted, fried, grilled or BBQ'd, no added fat	0.00
08A20849	18102016	Lamb, chop, fully-trimmed, baked, roasted, fried, grilled or BBQ'd, butter, dairy blend or margarine	0.00
08A20845	18102017	Lamb, chop, fully-trimmed, baked, roasted, fried, grilled or BBQ'd, canola oil	0.00
08A20856	18102018	Lamb, chop, fully-trimmed, baked, roasted, fried, grilled or BBQ'd, olive oil	0.00
08A20855	18102019	Lamb, chop, fully-trimmed, baked, roasted, fried, grilled or BBQ'd, other oil	0.00
08A20887	18102020	Lamb, chop, fully-trimmed, baked, roasted, fried, grilled or BBQ'd, fat not further defined	0.00
08A20878	18102021	Lamb, chop, fully-trimmed, boiled, casseroled, microwaved, poached, steamed or stewed, no added fat	0.00
08A20879	18102022	Lamb, chop, fully-trimmed, boiled, casseroled, microwaved, poached, steamed or stewed, fat not further defined	0.00
08F11032	18102023	Lamb, chop, fully-trimmed, coated, baked, roasted, fried, grilled or BBQ'd, fat not further defined	0.00
08A20830	18102024	Lamb, chop, semi-trimmed, raw, not further defined	0.00
08A20839	18102025	Lamb, chop, semi-trimmed, baked, roasted, fried, grilled or BBQ'd, no added fat	0.00
08A20852	18102026	Lamb, chop, semi-trimmed, baked, roasted, fried, grilled or BBQ'd, canola oil	0.00
08A20840	18102027	Lamb, chop, semi-trimmed, baked, roasted, fried, grilled or BBQ'd, olive oil	0.00
08A20857	18102028	Lamb, chop, semi-trimmed, baked, roasted, fried, grilled or BBQ'd, other oil	0.00
08A20858	18102029	Lamb, chop, semi-trimmed, baked, roasted, fried, grilled or BBQ'd, fat not further defined	0.00
08A20859	18102030	Lamb, chop, semi-trimmed, boiled, casseroled, microwaved, poached, steamed or stewed, no added fat	0.00

08A20888	18102031	Lamb, chop, semi-trimmed, boiled, casseroled, microwaved, poached, steamed or stewed, fat not further defined	0.00
08F11033	18102032	Lamb, chop, semi-trimmed, coated, baked, roasted, fried, grilled or BBQ'd, fat not further defined	0.00
08A20829	18102033	Lamb, chop, untrimmed, raw, not further defined	0.00
08A20837	18102034	Lamb, chop, untrimmed, baked, roasted, fried, grilled or BBQ'd, no added fat	0.00
08A20860	18102035	Lamb, chop, untrimmed, baked, roasted, fried, grilled or BBQ'd, canola oil	0.00
08A20836	18102036	Lamb, chop, untrimmed, baked, roasted, fried, grilled or BBQ'd, olive oil	0.00
08A20861	18102037	Lamb, chop, untrimmed, baked, roasted, fried, grilled or BBQ'd, other oil	0.00
08A20843	18102038	Lamb, chop, untrimmed, baked, roasted, fried, grilled or BBQ'd, fat not further defined	0.00
08F11035	18102039	Lamb, chop, untrimmed, coated, baked, roasted, fried, grilled or BBQ'd, with or without added fat	0.00
08A20665	18102040	Lamb, chump chop, separable lean, raw	0.00
08A20668	18102041	Lamb, chump chop, separable lean, grilled or BBQ'd, no added fat	0.00
08A20773	18102042	Lamb, chump chop, fully-trimmed, raw	0.00
08A20666	18102044	Lamb, chump chop, semi-trimmed, raw	0.00
08A20667	18102047	Lamb, chump chop, untrimmed, raw	0.00
08A20669	18102043	Lamb, chump chop, fully-trimmed, baked, roasted, fried, grilled or BBQ'd, no added fat	0.00
08A20670	18102045	Lamb, chump chop, semi-trimmed, baked, roasted, fried, grilled or BBQ'd, no added fat	0.00
08A20883	18102046	Lamb, chump chop, semi-trimmed, baked, roasted, fried, grilled or BBQ'd, fat not further defined	0.00
08A20671	18102048	Lamb, chump chop, untrimmed, baked, roasted, fried, grilled or BBQ'd, no added fat	0.00
08A20846	18102049	Lamb, chump chop, untrimmed, baked, roasted, fried, grilled or BBQ'd, fat not further defined	0.00
08A20672	18102050	Lamb, diced, separable lean, raw	0.00
08A20674	18102051	Lamb, diced, separable lean, fried or stir-fried, no added fat	0.00
08A20775	18102052	Lamb, diced, fully-trimmed, raw	0.00
08A20675	18102053	Lamb, diced, fully-trimmed, baked, roasted, fried, stir-fried, grilled or BBQ'd, no added fat	0.00
08A20877	18102054	Lamb, diced, fully-trimmed, boiled, casseroled, microwaved, poached, steamed or stewed, no added fat	0.00
08A20889	18102055	Lamb, diced, fully-trimmed, boiled, casseroled, microwaved, poached, steamed or stewed, fat not further defined	0.00
08A20673	18102056	Lamb, diced, untrimmed, raw	0.00
08A20676	18102057	Lamb, diced, untrimmed, baked, roasted, fried or stir-fried, grilled or BBQ'd, no added fat	0.00

08A20677	18102058	Lamb, drumstick, separable lean, raw	0.00
08A20680	18102059	Lamb, drumstick, separable lean, grilled or BBQ'd, no added fat	0.00
08A20786	18102060	Lamb, drumstick, fully-trimmed, baked, roasted, fried, grilled or BBQ'd, no added fat	0.00
08A20681	18102061	Lamb, drumstick, untrimmed, baked, roasted, fried, grilled or BBQ'd, no added fat	0.00
08A20682	18102062	Lamb, easy carve leg roast, separable lean, raw	0.00
08A20685	18102063	Lamb, easy carve leg roast, separable lean, baked or roasted, no added fat	0.00
08A20777	18102064	Lamb, easy carve leg roast, fully-trimmed, raw	0.00
08A20686	18102065	Lamb, easy carve leg roast, fully-trimmed, baked or roasted, no added fat	0.00
08A20683	18102066	Lamb, easy carve leg roast, semi-trimmed, raw	0.00
08A20687	18102067	Lamb, easy carve leg roast, semi-trimmed, baked or roasted, no added fat	0.00
08A20684	18102068	Lamb, easy carve leg roast, untrimmed, raw	0.00
08A20688	18102069	Lamb, easy carve leg roast, untrimmed, baked or roasted, no added fat	0.00
08A20689	18102070	Lamb, easy carve shoulder, separable lean, raw	0.00
08A20692	18102071	Lamb, easy carve shoulder, separable lean, baked or roasted, no added fat	0.00
08A20768	18102072	Lamb, easy carve shoulder, fully-trimmed, raw	0.00
08A20693	18102073	Lamb, easy carve shoulder, fully-trimmed, baked or roasted, no added fat	0.00
08A20690	18102074	Lamb, easy carve shoulder, semi-trimmed, raw	0.00
08A20694	18102075	Lamb, easy carve shoulder, semi-trimmed, baked or roasted, no added fat	0.00
08A20828	18707001	Lamb, easy carve shoulder, semi-trimmed, breadcrumb coating, baked or roasted, no added fat	0.00
08A20691	18102076	Lamb, easy carve shoulder, untrimmed, raw	0.00
08A20695	18102077	Lamb, easy carve shoulder, untrimmed, baked or roasted, no added fat	0.00
08A20767	18102078	Lamb, eye of loin, separable lean, raw	0.00
08A20696	18102079	Lamb, eye of loin, separable lean, grilled or BBQ'd, no added fat	0.00
08A20770	18102080	Lamb, fillet or tenderloin, fully-trimmed, raw	0.00
08A20727	18102081	Lamb, fillet or tenderloin, fully-trimmed, baked, roasted, fried, stir-fried, grilled or BBQ'd, no added fat	0.00
08A20891	18102082	Lamb, fillet or tenderloin, fully-trimmed, baked, roasted, fried, stir-fried, grilled or BBQ'd, fat not further defined	0.00
08A20784	18102083	Lamb, forequarter (easy carve shoulder, forequarter chop), separable fat, raw	0.00
08A20785	18102084	Lamb, forequarter (easy carve shoulder, forequarter chop), separable fat, grilled or BBQ'd, no added fat	0.00
08A20697	18102085	Lamb, forequarter chop, separable lean, raw	0.00
08A20701	18102086	Lamb, forequarter chop, separable lean, grilled or BBQ'd, no added fat	0.00

08A20698	18102087	Lamb, forequarter chop, fully-trimmed, raw	0.00
08A20702	18102088	Lamb, forequarter chop, fully-trimmed, baked, roasted, fried, grilled or BBQ'd, no added fat	0.00
08A20699	18102089	Lamb, forequarter chop, semi-trimmed, raw	0.00
08A20703	18102090	Lamb, forequarter chop, semi-trimmed, baked, roasted, fried, grilled or BBQ'd, no added fat	0.00
08A20867	18102091	Lamb, forequarter chop, semi-trimmed, baked, roasted, fried, grilled or BBQ'd, fat not further defined	0.00
08A20847	18102092	Lamb, forequarter chop, semi-trimmed, boiled, casseroled, microwaved, poached, steamed or stewed, with or without added fat	0.00
08A20700	18102093	Lamb, forequarter chop, untrimmed, raw	0.00
08A20704	18102094	Lamb, forequarter chop, untrimmed, baked, roasted, fried, grilled or BBQ'd, no added fat	0.00
08A20824	18102095	Lamb, forequarter chop, untrimmed, baked, roasted, fried, grilled or BBQ'd, fat not further defined	0.00
08A20705	18102096	Lamb, frenched cutlet/rack, separable lean, raw	0.00
08A20708	18102097	Lamb, frenched cutlet/rack, separable lean, grilled or BBQ'd, no added fat	0.00
08A20769	18102098	Lamb, frenched cutlet/rack, fully-trimmed, raw	0.00
08A20709	18102099	Lamb, frenched cutlet/rack, fully-trimmed, baked, roasted, fried, grilled or BBQ'd, no added fat	0.00
08A20825	18102100	Lamb, frenched cutlet/rack, fully-trimmed, baked, roasted, fried, grilled or BBQ'd, fat not further defined	0.00
08A20812	18102101	Lamb, frenched cutlet/rack, fully-trimmed, boiled, casseroled, microwaved, poached, steamed, or stewed, with or without added fat	0.00
08A20826	18707002	Lamb, frenched cutlet/rack, fully-trimmed, breadcrumb coating, baked, roasted, fried, grilled or BBQ'd, no added fat	0.00
08A20821	18707003	Lamb, frenched cutlet/rack, fully-trimmed, breadcrumb coating, baked, roasted, fried, grilled or BBQ'd, fat not further defined	0.00
08A20706	18102102	Lamb, frenched cutlet/rack, semi-trimmed, raw	0.00
08A20710	18102103	Lamb, frenched cutlet/rack, semi-trimmed, baked, roasted, fried, grilled or BBQ'd, no added fat	0.00
08A20822	18102104	Lamb, frenched cutlet/rack, semi-trimmed, baked, roasted, fried, grilled or BBQ'd, fat not further defined	0.00
08A20868	18707004	Lamb, frenched cutlet/rack, semi-trimmed, breadcrumb coating, baked, roasted, fried, grilled or BBQ'd, fat not further defined	0.00
08A20707	18102105	Lamb, frenched cutlet/rack, untrimmed, raw	0.00
08A20711	18102106	Lamb, frenched cutlet/rack, untrimmed, baked, roasted, fried, grilled or BBQ'd, no added fat	0.00
08A20894	18102107	Lamb, frenched cutlet/rack, untrimmed, baked, roasted, fried, grilled or BBQ'd, fat not further defined	0.00
08A20815	18102108	Lamb, kebab, baked, roasted, fried, grilled or BBQ'd, no added fat, no added marinade	0.00

08A20907	18102109	Lamb, kebab, marinated, baked, roasted, fried, grilled or BBQ'd, fat not further defined	0.00
08A20780	18102110	Lamb, leg (leg roast, mini roast, chump chop), separable fat, raw	0.00
08A20781	18102111	Lamb, leg (leg roast, mini roast, chump chop), separable fat, baked or roasted, no added fat	0.00
08A20712	18102112	Lamb, leg roast, separable lean, raw	0.00
08A20715	18102113	Lamb, leg roast, separable lean, baked or roasted, no added fat	0.00
08A20776	18102114	Lamb, leg roast, fully-trimmed, raw	0.00
08A20716	18102115	Lamb, leg roast, fully-trimmed, baked or roasted, no added fat	0.00
08A20713	18102116	Lamb, leg roast, semi-trimmed, raw	0.00
08A20717	18102117	Lamb, leg roast, semi-trimmed, baked or roasted, no added fat	0.00
08A20714	18102118	Lamb, leg roast, untrimmed, raw	0.00
08A20718	18102119	Lamb, leg roast, untrimmed, baked or roasted, no added fat	0.00
08A20719	18102120	Lamb, loin chop, separable lean, raw	0.00
08A20722	18102121	Lamb, loin chop, separable lean, grilled or BBQ'd, no added fat	0.00
08A20771	18102122	Lamb, loin chop, fully-trimmed, raw	0.00
08A20723	18102123	Lamb, loin chop, fully-trimmed, baked, roasted, fried, grilled or BBQ'd, no added fat	0.00
08A20720	18102124	Lamb, loin chop, semi-trimmed, raw	0.00
08A20724	18102125	Lamb, loin chop, semi-trimmed, baked, roasted, fried, grilled or BBQ'd, no added fat	0.00
08A20862	18102126	Lamb, loin chop, semi-trimmed, baked, roasted, fried, grilled or BBQ'd, fat not further defined	0.00
08A20721	18102127	Lamb, loin chop, untrimmed, raw	0.00
08A20725	18102128	Lamb, loin chop, untrimmed, baked, roasted, fried, grilled or BBQ'd, no added fat	0.00
08A20864	18102129	Lamb, loin chop, untrimmed, baked, roasted, fried, grilled or BBQ'd, fat not further defined	0.00
08A20782	18102130	Lamb, loin, separable fat, raw	0.00
08A20783	18102131	Lamb, loin, separable fat, grilled or BBQ'd, no added fat	0.00
08A20747	18102132	Lamb, mince, raw	0.00
08A20748	18102133	Lamb, mince, baked, roasted, fried, stir-fried, grilled or BBQ'd, no added fat	0.00
08A20871	18102134	Lamb, mince, baked, roasted, fried, stir-fried, grilled or BBQ'd, fat not further defined	0.00
08A20728	18102135	Lamb, mini roast, separable lean, raw	0.00
08A20731	18102136	Lamb, mini roast, separable lean, baked or roasted, no added fat	0.00
08A20779	18102137	Lamb, mini roast, fully-trimmed, raw	0.00
08A20732	18102138	Lamb, mini roast, fully-trimmed, baked or roasted, no added fat	0.00
08A20729	18102139	Lamb, mini roast, semi-trimmed, raw	0.00

08A20733	18102140	Lamb, mini roast, semi-trimmed, baked or roasted, no added fat	0.00
08A20730	18102141	Lamb, mini roast, untrimmed, raw	0.00
08A20734	18102142	Lamb, mini roast, untrimmed, baked or roasted, no added fat	0.00
08A20908	18102143	Lamb, rib, cooked, with or without added fat	0.00
08A20795	18102144	Lamb, roasting cuts, fully-trimmed, raw	0.00
08A20796	18102145	Lamb, roasting cuts, fully-trimmed, baked or roasted, no added fat	0.00
08A20876	18102146	Lamb, roasting cuts, fully-trimmed, boiled, casseroled, microwaved, poached, steamed or stewed, no added fat	0.00
08A20797	18102147	Lamb, roasting cuts, semi-trimmed, raw	0.00
08A20798	18102148	Lamb, roasting cuts, semi-trimmed, baked or roasted, no added fat	0.00
08A20854	18102149	Lamb, roasting cuts, untrimmed, raw	0.00
08A20881	18102150	Lamb, roasting cuts, untrimmed, baked or roasted, no added fat	0.00
08A20882	18102151	Lamb, roasting cuts, untrimmed, boiled, casseroled, microwaved, poached, steamed or stewed, no added fat	0.00
08A20774	18102152	Lamb, rump, separable lean, raw	0.00
08A20726	18102153	Lamb, rump, separable lean, grilled or BBQ'd, no added fat	0.00
08A20678	18102154	Lamb, shank, fully-trimmed, raw	0.00
08A20874	18102155	Lamb, shank, fully-trimmed, baked, roasted, fried, grilled or BBQ'd, no added fat	0.00
08A20872	18102156	Lamb, shank, fully-trimmed, baked, roasted, fried, grilled or BBQ'd, fat not further defined	0.00
08A20834	18102157	Lamb, shank, fully-trimmed, boiled, casseroled, microwaved, poached, steamed, or stewed, with or without added fat	0.00
08A20886	18102158	Lamb, shank, semi-trimmed, raw	0.00
08A20896	18102159	Lamb, shank, semi-trimmed, baked, roasted, fried, grilled or BBQ'd, no added fat	0.00
08A20897	18102160	Lamb, shank, semi-trimmed, boiled, casseroled, microwaved, poached, steamed or stewed, no added fat	0.00
08A20898	18102161	Lamb, shank, semi-trimmed, boiled, casseroled, microwaved, poached, steamed or stewed, fat not further defined	0.00
08A20679	18102162	Lamb, shank, untrimmed, raw	0.00
08A20873	18102163	Lamb, shank, untrimmed, baked, roasted, fried, grilled or BBQ'd, fat not further defined	0.00
08A20813	18102164	Lamb, shank, untrimmed, boiled, casseroled, microwaved, poached, steamed, or stewed, no added fat	0.00
08A20853	18102165	Lamb, shank, untrimmed, boiled, casseroled, microwaved, poached, steamed or stewed, fat not further defined	0.00

08A20735	18102166	Lamb, steak, separable lean, raw	0.00
08A20738	18102167	Lamb, steak, separable lean, baked or roasted, no added fat	0.00
08A20778	18102168	Lamb, steak, fully-trimmed, raw	0.00
08A20739	18102169	Lamb, steak, fully-trimmed, baked, roasted, fried, grilled or BBQ'd, no added fat	0.00
08A20899	18102170	Lamb, steak, fully-trimmed, baked, roasted, fried, grilled or BBQ'd, fat not further defined	0.00
08A20736	18102171	Lamb, steak, semi-trimmed, raw	0.00
08A20740	18102172	Lamb, steak, semi-trimmed, baked, roasted, fried, grilled or BBQ'd, no added fat	0.00
08A20737	18102173	Lamb, steak, untrimmed, raw	0.00
08A20741	18102174	Lamb, steak, untrimmed, baked, roasted, fried, grilled or BBQ'd, no added fat	0.00
08A20742	18102175	Lamb, stir-fry strips, separable lean, raw	0.00
08A20744	18102176	Lamb, stir-fry strips, separable lean, fried or stir fried, no added fat	0.00
08A20799	18102177	Lamb, stir-fry cuts, fully-trimmed, raw	0.00
08A20800	18102178	Lamb, stir-fry cuts, fully-trimmed, cooked, no added fat	0.00
08A20772	18102179	Lamb, stir-fry strips, fully-trimmed, raw	0.00
08A20745	18102180	Lamb, stir-fry strips, fully-trimmed, baked, roasted, fried, stir-fried, grilled or BBQ'd, no added fat	0.00
08A20901	18102181	Lamb, stir-fry strips, fully-trimmed, baked, roasted, fried, stir-fried, grilled or BBQ'd, fat not further defined	0.00
08A20900	18102182	Lamb, stir-fry strips, fully-trimmed, boiled, casseroled, microwaved, poached, steamed or stewed, with or without added fat	0.00
08A20743	18102183	Lamb, stir-fry strips, untrimmed, raw	0.00
08A20746	18102184	Lamb, stir-fry strips, untrimmed, baked, roasted, fried, stir-fried, grilled or BBQ'd, no added fat	0.00
08A20850	18102185	Lamb, stir-fry strips, untrimmed, baked, roasted, fried, stir-fried, grilled or BBQ'd, fat not further defined	0.00
08E10190	18102186	Lamb, for use in hamburgers, cooked	0.00
08C10604	18102187	Lamb, for use in kebabs, cooked	0.00
08A20885	18707005	Meatball or rissole, lamb mince, homemade, raw	0.00
08A20884	18707006	Meatball or rissole, lamb mince, homemade, baked, roasted, fried, grilled or BBQ'd, no added fat	0.00
08A20904	18707007	Meatball or rissole, lamb mince, homemade, baked, roasted, fried, grilled or BBQ'd, fat not further defined	0.00
08A20764	18102188	Mutton, all cuts, separable fat, raw	0.00
08A20765	18102189	Mutton, all cuts, separable fat, cooked	0.00
08A20789	18102190	Mutton, shoulder, separable fat, raw	0.00
08A20790	18102191	Mutton, shoulder, separable fat, boiled, casseroled, microwaved, poached, steamed, or stewed, no added fat	0.00
08A20751	18102192	Mutton, shoulder, separable lean, raw	0.00

08A20754	18102193	Mutton, shoulder, separable lean, boiled, casseroled, microwaved, poached, steamed, or stewed, no added fat	0.00
08A20752	18102194	Mutton, shoulder, fully-trimmed, raw	0.00
08A20755	18102195	Mutton, shoulder, fully-trimmed, boiled, casseroled, microwaved, poached, steamed, or stewed, no added fat	0.00
08A20753	18102196	Mutton, shoulder, untrimmed, raw	0.00
08A20756	18102197	Mutton, shoulder, untrimmed, boiled, casseroled, microwaved, poached, steamed, or stewed, no added fat	0.00
08A20787	18102198	Mutton, leg roast, separable fat, raw	0.00
08A20788	18102199	Mutton, leg roast, separable fat, baked or roasted, no added fat	0.00
08A20757	18102200	Mutton, leg roast, separable lean, raw	0.00
08A20760	18102201	Mutton, leg roast, separable lean, baked or roasted, no added fat	0.00
08A20758	18102202	Mutton, leg roast, fully-trimmed, raw	0.00
08A20761	18102203	Mutton, leg roast, fully-trimmed, baked or roasted, no added fat	0.00
08A20759	18102204	Mutton, leg roast, semi-trimmed, raw	0.00
08A20762	18102205	Mutton, leg roast, semi-trimmed, baked or roasted, no added fat	0.00
08A20791	18102206	Mutton, leg roast, untrimmed, raw	0.00
08A20763	18102207	Mutton, leg roast, untrimmed, baked or roasted, no added fat	0.00
08A40220	18104001	Veal, all cuts, separable fat, raw	0.00
08A40221	18104002	Veal, all cuts, separable fat, cooked	0.00
08A40184	18104003	Veal, cutlet, separable lean, raw	0.00
08A40188	18104004	Veal, cutlet, separable lean, fried, grilled or BBQ'd, no added fat	0.00
08A40185	18104005	Veal, cutlet, fully-trimmed, raw	0.00
08A40189	18104006	Veal, cutlet, fully-trimmed, baked, roasted, fried, grilled or BBQ'd, no added fat	0.00
08F11037	18104007	Veal, cutlet, fully-trimmed, breadcrumb coating, baked, roasted, fried, grilled or BBQ'd, fat not further defined	0.00
08A40186	18104008	Veal, cutlet, semi-trimmed, raw	0.00
08A40190	18104009	Veal, cutlet, semi-trimmed, baked, roasted, fried, grilled or BBQ'd, no added fat	0.00
08A40187	18104010	Veal, cutlet, untrimmed, raw	0.00
08A40191	18104011	Veal, cutlet, untrimmed, baked, roasted, fried, grilled or BBQ'd, no added fat	0.00
08A40192	18104012	Veal, diced, separable lean, raw	0.00
08A40195	18104013	Veal, diced, separable lean, fried, stir-fried, grilled or BBQ'd, no added fat	0.00
08A40193	18104014	Veal, diced, fully-trimmed, raw	0.00
08A40196	18104015	Veal, diced, fully-trimmed, baked, roasted, fried, stir-fried, grilled or BBQ'd, no added fat	0.00

08A40227	18104016	Veal, diced, fully-trimmed, baked, roasted, fried, stir-fried, grilled or BBQ'd, fat not further defined	0.00
08A40225	18104017	Veal, diced, fully-trimmed, boiled, casseroled, microwaved, poached, steamed, or stewed, no added fat	0.00
08A40194	18104018	Veal, diced, untrimmed, raw	0.00
08A40197	18104019	Veal, diced, untrimmed, baked, roasted, fried, stir-fried, grilled or BBQ'd, no added fat	0.00
08A40198	18104020	Veal, leg roast, separable lean, raw	0.00
08A40200	18104021	Veal, leg roast, separable lean, fried, grilled or BBQ'd, no added fat	0.00
08A40199	18104022	Veal, leg roast, fully-trimmed, raw	0.00
08A40201	18104023	Veal, leg roast, fully-trimmed, baked or roasted, no added fat	0.00
08A40202	18104024	Veal, leg steak, separable lean, raw	0.00
08A40205	18104025	Veal, leg steak, separable lean, fried, grilled or BBQ'd, no added fat	0.00
08A40203	18104026	Veal, leg steak, fully-trimmed, raw	0.00
08A40206	18104027	Veal, leg steak, fully-trimmed, baked, roasted, fried, grilled or BBQ'd, no added fat	0.00
08A40232	18104028	Veal, leg steak, fully-trimmed, baked, roasted, fried, grilled or BBQ'd, fat not further defined	0.00
08A40204	18104029	Veal, leg steak, untrimmed, raw	0.00
08A40207	18104030	Veal, leg steak, untrimmed, baked, roasted, fried, grilled or BBQ'd, no added fat	0.00
08A40222	18104031	Veal, loin chop, separable fat, raw	0.00
08A40223	18104032	Veal, loin chop, separable fat, fried, grilled or BBQ'd, no added fat	0.00
08A40208	18104033	Veal, loin chop, separable lean, raw	0.00
08A40211	18104034	Veal, loin chop, separable lean, fried, grilled or BBQ'd, no added fat	0.00
08A40209	18104035	Veal, loin chop, fully-trimmed, raw	0.00
08A40212	18104036	Veal, loin chop, fully-trimmed, baked, roasted, fried, grilled or BBQ'd, no added fat	0.00
08A40229	18104037	Veal, loin chop, semi-trimmed, raw	0.00
08A40210	18104038	Veal, loin chop, untrimmed, raw	0.00
08A40213	18104039	Veal, loin chop, untrimmed, baked, roasted, fried, grilled or BBQ'd, no added fat	0.00
08A40226	18104040	Veal, mince, untrimmed, raw	0.00
08A40214	18104041	Veal, stir-fry strips, separable lean, raw	0.00
08A40217	18104042	Veal, stir-fry strips, separable lean, fried, stir-fried, grilled or BBQ'd, no added fat	0.00
08A40215	18104043	Veal, stir-fry strips, fully-trimmed, raw	0.00
08A40218	18104044	Veal, stir-fry strips, fully-trimmed, baked, roasted, fried, stir-fried, grilled or BBQ'd, no added fat	0.00
08A40216	18104045	Veal, stir-fry strips, untrimmed, raw	0.00
08A40219	18104046	Veal, stir-fry strips, untrimmed, baked, roasted, fried, stir-fried, grilled or BBQ'd, no added fat	0.00

08F11020	18704001	Veal, schnitzel, breadcrumb coating, baked, roasted, fried, grilled or BBQ'd, no added fat	0.00
08F10926	18704002	Veal, schnitzel, breadcrumb coating, baked, roasted, fried, grilled or BBQ'd, canola oil	0.00
08F10915	18704003	Veal, schnitzel, breadcrumb coating, baked, roasted, fried, grilled or BBQ'd, olive oil	0.00
08F11021	18704004	Veal, schnitzel, breadcrumb coating, baked, roasted, fried, grilled or BBQ'd, other oil	0.00
08F11019	18704005	Veal, schnitzel, breadcrumb coating, baked, roasted, fried, grilled or BBQ'd, fat not further defined	0.00
08F11038	18704006	Veal, schnitzel, breadcrumb coating, boiled, casseroled, microwaved, poached, steamed or stewed, no added fat	0.00
08F10991	18704007	Veal, schnitzel, breadcrumb coating, cooked with tomato sauce & cheese (veal parmigiana)	0.00
15A20137	18011012	Pig (pork), wild caught, cooked	0.00
08A30494	18103001	Pork, belly, raw	0.00
08A30496	18103002	Pork, belly, baked, roasted, fried, grilled or BBQ'd, no added fat	0.00
08A30513	18103003	Pork, belly, baked, roasted, fried, grilled or BBQ'd, fat not further defined	0.00
08A30523	18103004	Pork, belly, boiled, casseroled, microwaved, poached, steamed or stewed, no added fat	0.00
08A30403	18103005	Pork, butterfly steak, fully-trimmed, raw	0.00
08A30404	18103006	Pork, butterfly steak, fully-trimmed, baked, roasted, fried, grilled or BBQ'd, no added fat	0.00
08A30510	18103007	Pork, butterfly steak, fully-trimmed, baked, roasted, fried, grilled or BBQ'd, canola oil	0.00
08A30525	18103008	Pork, butterfly steak, fully-trimmed, baked, roasted, fried, grilled or BBQ'd, olive oil	0.00
08A30524	18103009	Pork, butterfly steak, fully-trimmed, baked, roasted, fried, grilled or BBQ'd, other oil	0.00
08A30567	18103010	Pork, butterfly steak, fully-trimmed, baked, roasted, fried, grilled or BBQ'd, fat not further defined	0.00
08A30430	18103011	Pork, butterfly steak, untrimmed, raw	0.00
08A30431	18103012	Pork, butterfly steak, untrimmed, baked, roasted, fried, grilled or BBQ'd, no added fat	0.00
08A30429	18103013	Pork, crackling, roasted, salted	0.00
08A30411	18103014	Pork, diced, fully-trimmed, raw	0.00
08A30412	18103015	Pork, diced, fully-trimmed, baked, roasted, fried, stir-fried, grilled or BBQ'd, no added fat	0.00
08A30526	18103016	Pork, diced, fully-trimmed, baked, roasted, fried, stir-fried, grilled or BBQ'd, butter, dairy blend or margarine	0.00
08A30527	18103017	Pork, diced, fully-trimmed, baked, roasted, fried, stir-fried, grilled or BBQ'd, canola oil	0.00
08A30505	18103018	Pork, diced, fully-trimmed, baked, roasted, fried, stir-fried, grilled or BBQ'd, olive oil	0.00
08A30521	18103019	Pork, diced, fully-trimmed, baked, roasted, fried, stir-fried, grilled or BBQ'd, other oil	0.00

08A30528	18103020	Pork, diced, fully-trimmed, baked, roasted, fried, stir-fried, grilled or BBQ'd, fat not further defined	0.00
08A30515	18103021	Pork, diced, fully-trimmed, boiled, casseroled, microwaved, poached, steamed or stewed, no added fat	0.00
08A30530	18103022	Pork, diced, fully-trimmed, boiled, casseroled, microwaved, poached, steamed or stewed, fat not further defined	0.00
08A30405	18103023	Pork, fillet, fully-trimmed, raw	0.00
08A30406	18103024	Pork, fillet, fully-trimmed, baked, roasted, fried, grilled or BBQ'd, no added fat	0.00
08A30532	18103025	Pork, fillet, fully-trimmed, baked, roasted, fried, grilled or BBQ'd, butter, dairy blend or margarine	0.00
08A30533	18103026	Pork, fillet, fully-trimmed, baked, roasted, fried, grilled or BBQ'd, canola oil	0.00
08A30488	18103027	Pork, fillet, fully-trimmed, baked, roasted, fried, grilled or BBQ'd, olive oil	0.00
08A30506	18103028	Pork, fillet, fully-trimmed, baked, roasted, fried, grilled or BBQ'd, other oil	0.00
08A30490	18103029	Pork, fillet, fully-trimmed, baked, roasted, fried, grilled or BBQ'd, fat not further defined	0.00
08A30534	18103030	Pork, fillet, fully-trimmed, boiled, casseroled, microwaved, poached, steamed or stewed, no added fat	0.00
08A30419	18103031	Pork, forequarter (chop, roast, neck), separable fat, raw	0.00
08A30422	18103032	Pork, forequarter, separable fat, grilled or BBQ'd, no added fat	0.00
08A30407	18103033	Pork, forequarter chop, fully-trimmed, raw	0.00
08A30408	18103034	Pork, forequarter chop, fully-trimmed, baked, roasted, fried, grilled or BBQ'd, no added fat	0.00
08A30535	18103035	Pork, forequarter chop, fully-trimmed, baked, roasted, fried, grilled or BBQ'd, fat not further defined	0.00
08A30445	18103036	Pork, forequarter chop, semi-trimmed, baked, roasted, fried, grilled or BBQ'd, no added fat	0.00
08A30432	18103037	Pork, forequarter chop, untrimmed, raw	0.00
08A30433	18103038	Pork, forequarter chop, untrimmed, baked, roasted, fried, grilled or BBQ'd, no added fat	0.00
08A30536	18103039	Pork, forequarter chop, untrimmed, baked, roasted, fried, grilled or BBQ'd, fat not further defined	0.00
08A30409	18103040	Pork, forequarter shoulder roast, fully-trimmed, raw	0.00
08A30410	18103041	Pork, forequarter shoulder roast, fully-trimmed, baked or roasted, no added fat	0.00
08A30446	18103042	Pork, forequarter shoulder roast, semi-trimmed, baked or roasted, no added fat	0.00
08A30434	18103043	Pork, forequarter shoulder roast, untrimmed, raw	0.00
08A30435	18103044	Pork, forequarter shoulder roast, untrimmed, baked or roasted, no added fat	0.00
08A30569	18103045	Pork, head, cooked, with or without added fat	0.00

08A30566	18103046	Pork, kebab, marinated, baked, roasted, fried, grilled or BBQ'd, fat not further defined	0.00
08A30436	18103047	Pork, leg roast, untrimmed, raw	0.00
08A30437	18103048	Pork, leg roast, untrimmed, baked or roasted, no added fat	0.00
08A30420	18103049	Pork, leg steak (rump), separable fat, raw	0.00
08A30423	18103050	Pork, leg steak (rump), separable fat, fried or stir fried, no added fat	0.00
08A30413	18103051	Pork, leg steak (round, rump, topside, silverside), separable lean, raw	0.00
08A30414	18103052	Pork, leg steak (round, rump, topside, silverside), separable lean, fried or stir fried, no added fat	0.00
08A30438	18103053	Pork, leg steak (round, rump, topside, silverside), as purchased, raw	0.00
08A30439	18103054	Pork, leg steak (round, rump, topside, silverside), as purchased, fried or stir-fried, no added fat	0.00
08A30449	18103055	Pork, loin chop, separable fat, raw	0.00
08A30424	18103056	Pork, loin chop, separable fat, baked, roasted, fried, grilled or BBQ'd, no added fat	0.00
08A30448	18103057	Pork, loin chop, fully-trimmed, raw	0.00
08A30416	18103058	Pork, loin chop, fully-trimmed, baked, roasted, fried, grilled or BBQ'd, no added fat	0.00
08A30556	18103059	Pork, loin chop, fully-trimmed, baked, roasted, fried, grilled or BBQ'd, butter, dairy blend or margarine	0.00
08A30507	18103060	Pork, loin chop, fully-trimmed, baked, roasted, fried, grilled or BBQ'd, canola oil	0.00
08A30538	18103061	Pork, loin chop, fully-trimmed, baked, roasted, fried, grilled or BBQ'd, olive oil	0.00
08A30517	18103062	Pork, loin chop, fully-trimmed, baked, roasted, fried, grilled or BBQ'd, other oil	0.00
08A30537	18103063	Pork, loin chop, fully-trimmed, baked, roasted, fried, grilled or BBQ'd, fat not further defined	0.00
08A30516	18103064	Pork, loin chop, fully-trimmed, boiled, casseroled, microwaved, poached, steamed or stewed, no added fat	0.00
08A30520	18103065	Pork, loin chop, semi-trimmed, raw	0.00
08A30447	18103066	Pork, loin chop, semi-trimmed, baked, roasted, fried, grilled or BBQ'd, no added fat	0.00
08A30522	18103067	Pork, loin chop, semi-trimmed, baked, roasted, fried, grilled or BBQ'd, butter, dairy blend or margarine	0.00
08A30539	18103068	Pork, loin chop, semi-trimmed, baked, roasted, fried, grilled or BBQ'd, canola oil	0.00
08A30557	18103069	Pork, loin chop, semi-trimmed, baked, roasted, fried, grilled or BBQ'd, olive oil	0.00
08A30551	18103070	Pork, loin chop, semi-trimmed, baked, roasted, fried, grilled or BBQ'd, other oil	0.00
08A30514	18103071	Pork, loin chop, semi-trimmed, baked, roasted, fried, grilled or BBQ'd, fat not further defined	0.00

08A30540	18103072	Pork, loin chop, semi-trimmed, boiled, casseroled, microwaved, poached, steamed or stewed, no added fat	0.00
08A30441	18103073	Pork, loin chop, untrimmed, raw	0.00
08A30442	18103074	Pork, loin chop, untrimmed, baked, roasted, fried, grilled or BBQ'd, no added fat	0.00
08A30558	18103075	Pork, loin chop, untrimmed, baked, roasted, fried, grilled or BBQ'd, olive oil	0.00
08A30541	18103076	Pork, loin chop, untrimmed, baked, roasted, fried, grilled or BBQ'd, other oil	0.00
08A30477	18103077	Pork, loin cutlet, untrimmed, raw	0.00
08A30542	18103078	Pork, loin cutlet, untrimmed, baked, roasted, fried, grilled or BBQ'd, no added fat	0.00
08A30472	18103079	Pork, loin roast, separable fat, raw	0.00
08A30475	18103080	Pork, loin roast, separable fat, baked or roasted, no added fat	0.00
08A30471	18103081	Pork, loin roast, fully-trimmed, raw	0.00
08A30474	18103082	Pork, loin roast, fully-trimmed, baked or roasted, no added fat	0.00
08A30486	18103083	Pork, loin roast, fully-trimmed, boiled, casseroled, microwaved, poached, steamed, or stewed, no added fat	0.00
08A30553	18103084	Pork, loin roast, semi-trimmed, raw	0.00
08A30555	18103085	Pork, loin roast, semi-trimmed, baked or roasted, no added fat	0.00
08A30473	18103086	Pork, loin roast, untrimmed, raw	0.00
08A30476	18103087	Pork, loin roast, untrimmed, baked or roasted, no added fat	0.00
08A30421	18103088	Pork, medallion or loin steak, separable fat, raw	0.00
08A30453	18103089	Pork, medallion or loin steak, separable fat, fried, grilled or BBQ'd, no added fat	0.00
08A30427	18103090	Pork, medallion or loin steak, fully-trimmed, raw	0.00
08A30428	18103091	Pork, medallion or loin steak, fully-trimmed, baked, roasted, fried, grilled or BBQ'd, no added fat	0.00
08A30570	18103092	Pork, medallion or loin steak, fully-trimmed, baked, roasted, fried, grilled or BBQ'd, fat not further defined	0.00
08A30452	18103093	Pork, medallion or loin steak, untrimmed, raw	0.00
08A30571	18103094	Pork, medallion or loin steak, untrimmed, baked, roasted, fried, grilled or BBQ'd, fat not further defined	0.00
08A30425	18103095	Pork, mince, raw	0.00
08A30426	18103096	Pork, mince, baked, roasted, fried or stir-fried, grilled or BBQ'd, no added fat	0.00
08A30480	18103097	Pork, mince, baked, roasted, fried or stir-fried, grilled or BBQ'd, canola oil	0.00
08A30479	18103098	Pork, mince, baked, roasted, fried or stir-fried, grilled or BBQ'd, olive oil	0.00
08A30482	18103099	Pork, mince, baked, roasted, fried or stir-fried, grilled or BBQ'd, other oil	0.00

08A30481	18103100	Pork, mince, baked, roasted, fried or stir-fried, grilled or BBQ'd, fat not further defined	0.00
08A30546	18103101	Pork, mince, boiled, casseroled, microwaved, poached, steamed or stewed, no added fat	0.00
08A30491	18103102	Pork, mince, boiled, casseroled, microwaved, poached, steamed or stewed, added fat not further defined	0.00
08A30573	18103103	Pork, pickled, cooked, with or without added fat	0.00
08A30466	18103104	Pork, round mini roast, separable fat, raw	0.00
08A30469	18103105	Pork, round mini roast, separable fat, baked or roasted, no added fat	0.00
08A30465	18103106	Pork, round mini roast, fully-trimmed, raw	0.00
08A30468	18103107	Pork, round mini roast, fully-trimmed, baked or roasted, no added fat	0.00
08A30467	18103108	Pork, round mini roast, untrimmed, raw	0.00
08A30470	18103109	Pork, round mini roast, untrimmed, baked or roasted, no added fat	0.00
08A30456	18103110	Pork, round steak, fully-trimmed, raw	0.00
08A30461	18103111	Pork, round steak, fully-trimmed, baked, roasted, fried, grilled or BBQ'd, no added fat	0.00
08A30454	18103112	Pork, rump steak, fully-trimmed, raw	0.00
08A30460	18103113	Pork, rump steak, fully-trimmed, baked, roasted, fried, grilled or BBQ'd no added fat	0.00
08A30455	18103114	Pork, rump steak, untrimmed, raw	0.00
08A30559	18103115	Pork, rump steak, untrimmed, baked, roasted, fried, grilled or BBQ'd, no added fat	0.00
08A30450	18103116	Pork, scotch roast, separable fat, raw	0.00
08A30451	18103117	Pork, scotch roast, separable fat, baked or roasted, no added fat	0.00
08A30417	18103118	Pork, scotch roast, fully-trimmed, raw	0.00
08A30418	18103119	Pork, scotch roast, fully-trimmed, baked or roasted, no added fat	0.00
08A30443	18103120	Pork, scotch roast, untrimmed, raw	0.00
08A30444	18103121	Pork, scotch roast, untrimmed, baked or roasted, no added fat	0.00
08A30572	18103122	Pork, scotch roast, untrimmed, baked or roasted, fat not further defined	0.00
08A30459	18103123	Pork, silverside steak, fully-trimmed, raw	0.00
08A30464	18103124	Pork, silverside steak, fully-trimmed, baked, roasted, fried, grilled or BBQ'd, no added fat	0.00
08A30457	18103125	Pork, silverside steak, untrimmed, raw	0.00
08A30501	18103126	Pork, spare ribs, fully-trimmed, raw	0.00
08A30519	18103127	Pork, spare ribs, fully-trimmed, baked, roasted, fried, grilled or BBQ'd, no added fat	0.00
08A30568	18103128	Pork, spare ribs, fully-trimmed, baked, roasted, fried, grilled or BBQ'd, fat not further defined	0.00
08A30502	18103129	Pork, spare ribs, fully-trimmed, boiled, casseroled, microwaved, poached, steamed, or stewed, no added fat	0.00

08A30503	18103130	Pork, spare ribs, fully-trimmed, boiled, casseroled, microwaved, poached, steamed, or stewed, fat not further defined	0.00
08A30497	18103131	Pork, spare ribs, semi-trimmed, raw	0.00
08A30498	18103132	Pork, spare ribs, semi-trimmed, baked, roasted, fried, grilled or BBQ'd, no added fat	0.00
08A30518	18103133	Pork, spare ribs, semi-trimmed, baked, roasted, fried, grilled or BBQ'd, fat not further defined	0.00
08A30500	18103134	Pork, spare ribs, semi-trimmed, boiled, casseroled, microwaved, poached, steamed, or stewed, no added fat	0.00
08A30552	18103135	Pork, spare ribs, untrimmed, raw	0.00
08A30554	18103136	Pork, spare ribs, untrimmed, baked, roasted, fried, grilled or BBQ'd, no added fat	0.00
08A30561	18103137	Pork, spare ribs, untrimmed, baked, roasted, fried, grilled or BBQ'd, fat not further defined	0.00
08A30564	18103138	Pork, spare ribs, untrimmed, boiled, casseroled, microwaved, poached, steamed or stewed, no added fat	0.00
08A30563	18103139	Pork, spare ribs, untrimmed, boiled, casseroled, microwaved, poached, steamed or stewed, fat not further defined	0.00
08A30565	18103140	Pork, spare ribs, marinated, baked, roasted, fried, grilled or BBQ'd, fat not further defined	0.00
08A30440	18103141	Pork, strips, fully-trimmed, raw	0.00
08A30415	18103142	Pork, strips, fully-trimmed, baked, roasted, fried, stir-fried, grilled or BBQ'd, no added fat	0.00
08A30458	18103143	Pork, topside steak, fully-trimmed, raw	0.00
08A30462	18103144	Pork, topside steak, fully-trimmed, baked, roasted, fried, grilled or BBQ'd, no added fat	0.00
08A30463	18103145	Pork, topside steak, untrimmed, baked, roasted, fried, grilled or BBQ'd, no added fat	0.00
08F11133	18710001	Pork, coated, baked, roasted, fried, grilled or BBQ'd, no added fat	0.00
08F11129	18710002	Pork, coated, baked, roasted, fried, grilled or BBQ'd, fat not further defined	0.00
08A30492	18710003	Meatball or rissole, pork mince, homemade, raw	0.00
08A30499	18710004	Meatball or rissole, pork mince, homemade, baked, roasted, fried, grilled or BBQ'd, no added fat	0.00
08A30493	18710005	Meatball or rissole, pork mince, homemade, baked, roasted, fried, grilled or BBQ'd, fat not further defined	0.00
08C10462	18301001	Chicken, barbecued, with skin, commercial	0.00
08C10493	18301002	Chicken, barbecued, without skin, commercial	0.00
08C10431	18301003	Chicken, breast, flesh, raw	0.00
08C10432	18301004	Chicken, breast, flesh, baked or roasted, no added fat	0.00
08C10478	18301005	Chicken, breast, flesh, grilled or BBQ'd, no added fat	0.00
08C10449	18301006	Chicken, breast, flesh, fried or stir-fried, no added fat	0.00
08C10566	18301007	Chicken, breast, flesh, baked, roasted, fried, stir-fried, grilled or BBQ'd, butter, dairy blend, margarine spread	0.00

08C10482	18301008	Chicken, breast, flesh, baked, roasted, fried, stir-fried, grilled or BBQ'd, canola oil	0.00
08C10481	18301009	Chicken, breast, flesh, baked, roasted, fried, stir-fried, grilled or BBQ'd, olive oil	0.00
08C10483	18301010	Chicken, breast, flesh, baked, roasted, fried, stir-fried, grilled or BBQ'd, other oil	0.00
08C10522	18301011	Chicken, breast, flesh, baked, roasted, fried, stir-fried, grilled or BBQ'd, fat not further defined	0.00
08C10443	18301012	Chicken, breast, flesh, boiled, casseroled, microwaved, poached, steamed or stewed, no added fat	0.00
08C10555	18301013	Chicken, breast, flesh, boiled, casseroled, microwaved, poached, steamed or stewed, fat not further defined	0.00
08C10615	18301014	Chicken, breast, flesh, smoked, no added fat	0.00
08E10155	18903001	Chicken, breast, flesh, breadcrumb coating, cooked, no added fat	0.00
08E10149	18903002	Chicken, breast, flesh, breadcrumb coating, cooked, fat not further defined	0.00
08E10213	18903003	Chicken, breast, flesh, purchased frozen with breadcrumb coating, cooked, no added fat	0.00
08E10171	18903004	Chicken, breast, flesh, purchased frozen with breadcrumb coating, cooked, fat not further defined	0.00
08E10139	18903005	Chicken, breast, flesh, coated, cooked, no added fat	0.00
08E10170	18903006	Chicken, breast, flesh, coated, cooked, fat not further defined	0.00
08C10550	18606001	Chicken, breast, flesh, canned in water, drained	0.00
08C10466	18301015	Chicken, breast, flesh, skin & fat, raw	0.00
08C10467	18301016	Chicken, breast, flesh, skin & fat, baked, roasted, fried, grilled or BBQ'd, no added fat	0.00
08C10570	18301017	Chicken, breast, flesh, skin & fat, baked, roasted, fried, grilled or BBQ'd, fat not further defined	0.00
08C10464	18301018	Chicken, breast, flesh, skin & fat, boiled, casseroled, microwaved, poached, steamed or stewed, with or without added fat	0.00
08E10168	18903007	Chicken, breast, flesh, skin & fat, coated, baked, roasted, fried, grilled or BBQ'd, fat not further defined	0.00
08E10130	18903027	Chicken, breast, flesh, skin & fat, coated, fast food chain, fried, undefined fat	0.00
08C10437	18301019	Chicken, drumstick, flesh, raw	0.00
08C10438	18301020	Chicken, drumstick, flesh, baked, roasted, fried, grilled or BBQ'd, no added fat	0.00
08C10497	18301021	Chicken, drumstick, flesh, baked, roasted, fried, grilled or BBQ'd, fat not further defined	0.00
08C10446	18301022	Chicken, drumstick, flesh, boiled, casseroled, microwaved, poached, steamed or stewed, no added fat	0.00
08C10558	18301023	Chicken, drumstick, flesh, boiled, casseroled, microwaved, poached, steamed or stewed, fat not further defined	0.00

08E10182	18903008	Chicken, drumstick, flesh, coated, cooked, no added fat	0.00
08E10162	18903009	Chicken, drumstick, flesh, coated, cooked, fat not further defined	0.00
08C10474	18301024	Chicken, drumstick, flesh, skin & fat, raw	0.00
08C10475	18301025	Chicken, drumstick, flesh, skin & fat, baked, roasted, fried, grilled or BBQ'd, no added fat	0.00
08C10531	18301026	Chicken, drumstick, flesh, skin & fat, baked, roasted, fried, grilled or BBQ'd, fat not further defined	0.00
08C10476	18301027	Chicken, drumstick, flesh, skin & fat, boiled, casseroled, microwaved, poached, steamed or stewed, with or without added fat	0.00
08E10152	18903010	Chicken, drumstick, flesh, skin & fat, coated, cooked, no added fat	0.00
08E10176	18903011	Chicken, drumstick, flesh, skin & fat, coated, cooked, fat not further defined	0.00
08E10133	18903029	Chicken, drumstick, flesh, skin & fat, coated, fast food chain, fried, undefined fat	0.00
08C10614	18301028	Chicken, drumstick, flesh, skin & fat, marinated, baked, roasted, fried, grilled or BBQ'd, no added fat	0.00
08C10610	18301029	Chicken, feet, boiled, casseroled, microwaved, poached or steamed, with or without added fat	0.00
08C10508	18301030	Chicken, fillet or kebab, flesh, raw	0.00
08C10513	18301031	Chicken, fillet or kebab, flesh, baked, roasted, fried, stir-fried, grilled or BBQ'd, no added fat, no added marinade	0.00
08C10611	18301032	Chicken, fillet or kebab, flesh, baked, roasted, fried, stir-fried, grilled or BBQ'd, butter or dairy blend, no added marinade	0.00
08C10607	18301033	Chicken, fillet or kebab, flesh, baked, roasted, fried, stir-fried, grilled or BBQ'd, canola oil, no added marinade	0.00
08C10533	18301034	Chicken, fillet or kebab, flesh, baked, roasted, fried, stir-fried, grilled or BBQ'd, olive oil, no added marinade	0.00
08C10504	18301035	Chicken, fillet or kebab, flesh, baked, roasted, fried, stir-fried, grilled or BBQ'd, other oil, no added marinade	0.00
08C10519	18301036	Chicken, fillet or kebab, flesh, baked, roasted, fried, stir-fried, grilled or BBQ'd, fat not further defined, no added marinade	0.00
08C10495	18301037	Chicken, fillet or kebab, flesh, boiled, steamed, poached, stewed, casseroled or microwaved, with or without added fat, no added marinade	0.00
08E10142	18903012	Chicken, fillet or kebab, flesh, coated, cooked, no added fat, no added marinade	0.00
08E10151	18903013	Chicken, fillet or kebab, coated, cooked, fat not further defined, no added marinade	0.00
08C10524	18301038	Chicken, fillet or kebab, flesh, skin & fat, raw	0.00

08C10534	18301039	Chicken, fillet or kebab, flesh, skin & fat, baked, roasted, fried, stir-fried, grilled or BBQ'd, no added fat, no added marinade	0.00
08C10599	18301040	Chicken, fillet or kebab, flesh, skin & fat, baked, roasted, fried, stir-fried, grilled or BBQ'd, fat not further defined, no added marinade	0.00
08C10597	18301041	Chicken, fillet or kebab, flesh, skin & fat, boiled, casseroled, microwaved, poached, steamed or stewed, with or without added fat, no added marinade	0.00
08C10523	18301042	Chicken, fillet or kebab, raw, not further defined	0.00
08C10545	18301043	Chicken kebab, marinated, baked, roasted, fried, grilled or BBQ'd, no added fat	0.00
08C10590	18301044	Chicken kebab, marinated, baked, roasted, fried, grilled or BBQ'd, fat not further defined	0.00
08C10592	18301045	Chicken, maryland, flesh, raw	0.00
08C10593	18301046	Chicken, maryland, flesh, baked, roasted, fried, grilled or BBQ'd, no added fat	0.00
08C10591	18301047	Chicken, maryland, flesh, baked, roasted, fried, grilled or BBQ'd, fat not further defined	0.00
08E10211	18903014	Chicken, maryland, flesh, coated, cooked, no added fat	0.00
08E10210	18903015	Chicken, maryland, flesh, coated, cooked, fat not further defined	0.00
08C10505	18301048	Chicken, maryland, flesh, skin & fat, raw	0.00
08C10594	18301049	Chicken, maryland, flesh, skin & fat, baked, roasted, fried, grilled or BBQ'd, no added fat	0.00
08C10506	18301050	Chicken, maryland, flesh, skin & fat, baked, roasted, fried, grilled or BBQ'd, fat not further defined	0.00
08C10589	18301051	Chicken, maryland, flesh, skin & fat, boiled, casseroled, microwaved, poached, steamed or stewed, with or without added fat	0.00
08E10208	18903016	Chicken, maryland, flesh, skin & fat, coated, cooked, no added fat	0.00
08C10500	18301052	Chicken, mince, raw	0.00
08C10548	18301053	Chicken, mince, baked, roasted, fried, stir-fried, grilled or BBQ'd, no added fat	0.00
08C10578	18301054	Chicken, mince, baked, roasted, fried, stir-fried, grilled or BBQ'd, fat not further defined	0.00
08E10129	18903026	Chicken, bite-size pieces, coated, fast food chain, fried, fat not further defined	0.00
08E10138	18903028	Chicken, breast strip, coated, fast food chain, fried, fat not further defined	0.00
08E10148	18903030	Chicken, finger or chip, purchased frozen, baked, roasted, fried, grilled or BBQ'd, no added fat	0.00
08E10122	18903034	Chicken, nugget, purchased frozen, baked, roasted, fried, grilled or BBQ'd, with or without added fat	0.00
08E10125	18903035	Chicken, nugget, purchased from takeaway chain, fried, canola oil	0.00
08E10123	18903036	Chicken, nugget, purchased from independent & chain takeaway outlets, fried, undefined oil	0.00

08E10124	18903037	Chicken, nugget, purchased from takeaway chain, grilled	0.00
08E10141	18903038	Chicken piece, flesh, skin & fat, coated, fast food outlet, fried, undefined oil	0.00
08C10441	18301079	Chicken, separable fat, composite, raw	0.00
08C10448	18301080	Chicken, separable fat, composite, boiled, casseroled, microwaved, poached, steamed or stewed, no added fat	0.00
08C10442	18301081	Chicken, separable fat, composite, baked or roasted, no added fat	0.00
08C10439	18301082	Chicken, skin, composite, raw	0.00
08C10440	18301083	Chicken, skin, composite, baked or roasted, no added fat	0.00
08C10447	18301084	Chicken, skin, composite, boiled, casseroled, microwaved, poached, steamed or stewed, no added fat	0.00
08C10435	18301055	Chicken, thigh, flesh, raw	0.00
08C10436	18301056	Chicken, thigh, flesh, baked, roasted, fried, grilled or BBQ'd, no added fat	0.00
08C10485	18301057	Chicken, thigh, flesh, baked, roasted, fried, grilled or BBQ'd, fat not further defined	0.00
08C10445	18301058	Chicken, thigh, flesh, boiled, casseroled, microwaved, poached, steamed or stewed, with or without added fat	0.00
08E10199	18903017	Chicken, thigh, flesh, coated, baked, roasted, fried, grilled or BBQ'd, no added fat	0.00
08E10197	18903018	Chicken, thigh, flesh, coated, baked, roasted, fried, grilled or BBQ'd, fat not further defined	0.00
08C10468	18301059	Chicken, thigh, flesh, skin & fat, raw	0.00
08C10469	18301060	Chicken, thigh, flesh, skin & fat, baked, roasted, fried, grilled or BBQ'd, no added fat	0.00
08C10576	18301061	Chicken, thigh, flesh, skin & fat, baked, roasted, fried, grilled or BBQ'd, fat not further defined	0.00
08C10470	18301062	Chicken, thigh, flesh, skin & fat, boiled, casseroled, microwaved, poached, steamed or stewed, with or without added fat	0.00
08E10131	18903045	Chicken, thigh, flesh, skin & fat, coated, fast food chain, fried, undefined fat	0.00
08C10477	18301063	Chicken, thigh fillets, flesh & some fat, raw	0.00
08C10433	18301064	Chicken, wing, flesh, raw	0.00
08C10434	18301065	Chicken, wing, flesh, baked, roasted, fried, grilled or BBQ'd, no added fat	0.00
08C10569	18301066	Chicken, wing, flesh, baked, roasted, fried, grilled or BBQ'd, fat not further defined	0.00
08C10444	18301067	Chicken, wing, flesh, boiled, casseroled, microwaved, poached, steamed or stewed, with or without added fat	0.00
08E10179	18903019	Chicken, wing, flesh, coated, baked, roasted, fried, grilled or BBQ'd, no added fat	0.00
08C10471	18301068	Chicken, wing, flesh, skin & fat, raw	0.00

08C10472	18301069	Chicken, wing, flesh, skin & fat, baked, roasted, fried, grilled or BBQ'd, no added fat	0.00
08C10489	18301070	Chicken, wing, flesh, skin & fat, baked, roasted, fried, grilled or BBQ'd, fat not further defined	0.00
08C10473	18301071	Chicken, wing, flesh, skin & fat, boiled, casseroled, microwaved, poached, steamed or stewed, with or without added fat	0.00
08E10157	18903020	Chicken, wing, flesh, skin & fat, coated, baked, roasted, fried, grilled or BBQ'd, no added fat	0.00
08E10156	18903021	Chicken, wing, flesh, skin & fat, coated, baked, roasted, fried, grilled or BBQ'd, fat not further defined	0.00
08E10132	18903046	Chicken, wing, flesh, skin & fat, coated, fast food chain, fried, undefined fat	0.00
08E10178	18903047	Chicken, wing, flesh, skin & fat, purchased frozen, breadcrumb coating, baked, roasted, fried, grilled or BBQ'd, with or without added fat	0.00
08C10494	18301072	Chicken, whole, lean flesh, baked, roasted, fried, grilled or BBQ'd, no added fat	0.00
08C10525	18301073	Chicken, whole, flesh, skin & fat, raw	0.00
08C10526	18301074	Chicken, whole, flesh, skin & fat, baked, roasted, fried, grilled or BBQ'd, no added fat	0.00
08E10158	18903022	Chicken, whole, flesh, skin & fat, coated, baked, roasted, fried, grilled or BBQ'd, no added fat	0.00
08C10509	18301075	Chicken, flesh, raw	0.00
08C10510	18301076	Chicken, flesh, baked, roasted, fried, grilled or BBQ'd, no added fat	0.00
08C10498	18301077	Chicken, flesh, baked, roasted, fried, grilled or BBQ'd, fat not further defined	0.00
08C10511	18301078	Chicken, flesh, boiled, steamed, poached, stewed, casseroled or microwaved, with or without added fat	0.00
08E10215	18903023	Chicken, flesh, coated, baked, roasted, fried, grilled or BBQ'd, no added fat	0.00
08E10189	18903024	Chicken, for use in hamburgers, cooked	0.00
08C10603	18301085	Chicken, for use in kebabs, cooked	0.00
08C10512	18301086	Chicken, for use as a sandwich filling, not further defined	0.00
08C10605	18301087	Chicken, for use as subway sandwich filling	0.00
08E10212	18903031	Chicken, kiev, purchased frozen with breadcrumb coating, baked, roasted, fried, grilled or BBQ'd, cooked with or without added fat	0.00
08E10137	18903032	Chicken, kiev, homemade from basic ingredients, baked, roasted, fried, grilled or BBQ'd, no added fat	0.00
08E10166	18903033	Chicken, kiev, homemade from basic ingredients, baked, roasted, fried, grilled or BBQ'd, fat not further defined	0.00
08E10143	18903039	Chicken, schnitzel, breadcrumb coating, baked, roasted, fried, grilled or BBQ'd, no added fat	0.00

08E10145	18903040	Chicken, schnitzel, breadcrumb coating, baked, roasted, fried, grilled or BBQ'd, butter, dairy blend or margarine	0.00
08E10128	18903041	Chicken, schnitzel, breadcrumb coating, baked, roasted, fried, grilled or BBQ'd, canola oil	0.00
08E10127	18903042	Chicken, schnitzel, breadcrumb coating, baked, roasted, fried, grilled or BBQ'd, olive oil	0.00
08E10134	18903043	Chicken, schnitzel, breadcrumb coating, baked, roasted, fried, grilled or BBQ'd, other oil	0.00
08E10163	18903044	Chicken, schnitzel, breadcrumb coating, baked, roasted, fried, grilled or BBQ'd, fat not further defined	0.00
08C10501	18903048	Meatball or rissole, chicken mince, homemade from basic ingredients, raw	0.00
08C10507	18903049	Meatball or rissole, chicken mince, homemade, baked, roasted, fried, grilled or BBQ'd, no added fat	0.00
08C10552	18903050	Meatball or rissole, chicken mince, homemade, baked, roasted, fried, grilled or BBQ'd, fat not further defined	0.00
08C10516	18903051	Meatball or rissole, chicken mince, commercial, breadcrumb coating, cooked, fat not further defined	0.00
08C10454	18302011	Turkey, breast, lean, raw	0.00
08C10450	18302012	Turkey, breast, lean, baked, roasted, fried, grilled or BBQ'd, no added fat	0.00
08C10613	18302013	Turkey, breast, lean, baked, roasted, fried, grilled or BBQ'd, fat not further defined	0.00
08C10528	18903054	Turkey, breast, lean, breadcrumb coating, baked, roasted, fried, grilled or BBQ'd, fat not further defined	0.00
08C10456	18302014	Turkey, breast, lean, skin & fat, raw	0.00
08C10455	18302015	Turkey, breast, lean, skin & fat, baked, roasted, fried, grilled or BBQ'd, no added fat	0.00
08C10451	18302016	Turkey, hindquarter, lean, raw	0.00
08C10457	18302017	Turkey, hindquarter, lean, baked, roasted, fried, grilled or BBQ'd, no added fat	0.00
08C10453	18302018	Turkey, hindquarter, lean, skin & fat, raw	0.00
08C10452	18302019	Turkey, hindquarter, lean, skin & fat, baked, roasted, fried, grilled or BBQ'd, no added fat	0.00
08C10606	18302020	Turkey, hindquarter, lean, skin & fat, baked, roasted, fried, grilled or BBQ'd, fat not further defined	0.00
08C10602	18302021	Turkey, hindquarter, lean, skin & fat, boiled, casseroled, microwaved, poached, steamed or stewed, with or without added fat	0.00
08C10529	18302022	Turkey, whole, lean, baked, roasted, fried, grilled or BBQ'd, no added fat	0.00
08C10530	18302023	Turkey, whole, lean, boiled, casseroled, microwaved, poached, steamed or stewed, with or without added fat	0.00
08C10608	18302024	Turkey, whole, lean, skin & fat, baked, roasted, fried, grilled or BBQ'd, no added fat	0.00
15A20142	18011014	Turkey, wild caught, lean, skin & fat, cooked	0.00
08C10459	18302001	Duck, lean, raw	0.00

08C10461	18302002	Duck, lean, baked, roasted, fried, grilled or BBQ'd, no added fat	0.00
08C10612	18302003	Duck, lean, baked, roasted, fried, stir-fried, grilled or BBQ'd, fat not further defined	0.00
08C10543	18302004	Duck, lean, boiled, casseroled, microwaved, poached, steamed or stewed, no added fat	0.00
08C10458	18302005	Duck, skin & fat, raw	0.00
08C10460	18302006	Duck, skin & fat, baked or roasted, fried, grilled or BBQ'd, no added fat	0.00
08C10463	18302007	Duck, lean, skin & fat, raw	0.00
08C10465	18302008	Duck, lean, skin & fat, baked, roasted, fried, grilled or BBQ'd, no added fat	0.00
08C10600	18302009	Duck, lean, skin & fat, baked, roasted, fried, grilled or BBQ'd, fat not further defined	0.00
08E10159	18302010	Duck, lean, skin & fat, coated, baked, roasted, fried, grilled or BBQ'd, with or without added fat	0.00
15A20133	18011006	Goose, wild caught, flesh, raw	0.00
15A20139	18011007	Goose, wild caught, flesh, cooked	0.00
08C20016	18303008	Quail, flesh & skin, raw	0.00
08C20017	18303009	Quail, flesh & skin, baked, roasted, fried, grilled or BBQ'd, no added fat	0.00
08C20023	18303010	Quail, flesh & skin, baked, roasted, fried, grilled or BBQ'd, fat not further defined	0.00
08B10070	18202003	Buffalo, swamp, cube roll, raw	0.00
08B10071	18202004	Buffalo, swamp, topside, raw	0.00
08B10072	18202001	Buffalo, riverine, cube roll, raw	0.00
08B10073	18202002	Buffalo, riverine, topside, raw	0.00
08B10097	18011001	Buffalo, wild caught, cooked	0.00
08B10074	18202005	Camel, cube roll, raw	0.00
08B10075	18202006	Camel, rump, raw	0.00
08B10076	34101001	Crocodile, tail fillet, raw	0.00
08B10077	34101002	Crocodile, back leg, raw	0.00
08B10098	34101003	Crocodile, cooked	0.00
15A20136	18011002	Echidna, wild caught, flesh, raw	0.00
15A20141	18011003	Echidna, wild caught, flesh, cooked	0.00
08C20021	18303001	Emu, fan fillet, raw	0.00
08C20022	18303003	Emu, steak, raw	0.00
08C20024	18303002	Emu, fillet or steak, baked, roasted, fried, stir-fried, grilled or BBQ'd, no added fat	0.00
15A20135	34101004	Goanna, wild caught, flesh, cooked	0.00
08B10092	18202007	Goat, forequarter, separable lean, raw	0.00
08B10093	18202008	Goat, separable fat (composite), raw	0.00
08B10088	18202009	Goat, flesh, raw	0.00
08B10090	18202010	Goat, cooked, with or without added fat	0.00
15A40034	34201001	Insect, wild caught, raw	0.00
08B10066	18201005	Kangaroo, rump, raw	0.00
08B10067	18201006	Kangaroo, rump, baked, roasted, fried, grilled or BBQ'd, no added fat	0.00

08B10068	18201001	Kangaroo, loin fillet, raw	0.00
08B10069	18201002	Kangaroo, loin fillet, baked, roasted, fried, grilled or BBQ'd, no added fat	0.00
08B10086	18201003	Kangaroo, loin fillet, baked, roasted, fried, grilled or BBQ'd, fat not further defined	0.00
08B10087	18201004	Kangaroo, loin fillet, boiled, casseroled, microwaved, poached, steamed or stewed, fat not further defined	0.00
08B10095	18201007	Kangaroo, tail, raw	0.00
08B10094	18201008	Kangaroo, tail, cooked	0.00
15A20143	18011008	Kangaroo, wild caught, flesh, cooked	0.00
15A20147	18011009	Kangaroo, wild caught, flesh & organs, cooked	0.00
15A20145	18011010	Kangaroo, wild caught, tail, cooked	0.00
08B10096	18711006	Meatball or rissole, kangaroo mince, cooked, with or without added fat	0.00
08C20025	18303004	Mutton-bird, cooked	0.00
08C20018	18303005	Ostrich, fan fillet, raw	0.00
08C20019	18303006	Ostrich, moon steak, raw	0.00
08C20020	18303007	Pigeon (squab), whole, raw	0.00
15A20131	18011013	Possum, wild caught, flesh, cooked	0.00
08B10064	18202011	Rabbit, farmed, whole, raw	0.00
08B10065	18202012	Rabbit, flesh, boiled, casseroled, microwaved, poached, steamed, or stewed, no added fat	0.00
08B10079	18202013	Venison, diced, lean, raw	0.00
08B10078	18202014	Venison, diced, lean, baked, roasted, fried, stir-fried, grilled or BBQ'd, no added fat	0.00
08B10081	18202015	Venison, leg medallion, lean, raw	0.00
08B10080	18202016	Venison, leg medallion, lean, baked, roasted, fried, stir-fried, grilled or BBQ'd, no added fat	0.00
08B10091	18202017	Venison, leg medallion, lean, baked, roasted, fried, stir-fried, grilled or BBQ'd, fat not further defined	0.00
08B10083	18202018	Venison, mince, premium, raw	0.00
08B10082	18202019	Venison, mince, premium, baked, roasted, fried, stir-fried, grilled or BBQ'd, no added fat	0.00
08B10085	18202020	Venison, stir fry strips, lean, raw	0.00
08B10084	18202021	Venison, stir fry strips, lean, baked, roasted, fried, stir-fried, grilled or BBQ'd, no added fat	0.00
15A20146	18011011	Meat, wild caught, cooked	0.00
08D10165	18403001	Beef, heart, raw	0.00
08D10166	18403002	Beef, heart, simmered or boiled, no added fat	0.00
08D10167	18401001	Beef, liver, raw	0.00
08D10168	18401002	Beef, liver, simmered or boiled, no added fat	0.00
08D10169	18402001	Beef, kidney, raw	0.00
08D10170	18402002	Beef, kidney, simmered or boiled, no added fat	0.00
08D10171	18403003	Beef, tail, raw	0.00
08D10172	18403004	Beef, tail, simmered or boiled, no added fat	0.00
08D10173	18403005	Beef, tongue, raw	0.00
08D10174	18403006	Beef, tongue, simmered or boiled, no added fat	0.00
08D10175	18403007	Beef, tripe, raw	0.00

08D10176	18403008	Beef, tripe, simmered or boiled, no added fat	0.00
08D10193	18405001	Black pudding, raw	0.00
08D10196	18405002	Black pudding, fried, grilled, BBQ'd or baked, fat not further defined	0.00
08D10194	18401003	Chicken, liver, raw	0.00
08D10195	18401004	Chicken, liver, fried, baked, grilled or BBQ'd, added fat	0.00
08D10177	18403009	Lamb, brain, raw	0.00
08D10178	18403010	Lamb, brain, simmered or boiled, no added fat	0.00
08D10179	18403011	Lamb, heart, raw	0.00
08D10180	18403012	Lamb, heart, baked or roasted, no added fat	0.00
08D10199	18403017	Lamb, intestine, simmered or boiled, no added fat	0.00
08D10181	18402003	Lamb, kidney, raw	0.00
08D10182	18402004	Lamb, kidney, simmered or boiled, no added fat	0.00
08D10185	18401005	Lamb, liver, raw	0.00
08D10186	18401006	Lamb, liver, fried, grilled or BBQ'd, no added fat	0.00
08D10197	18401007	Lamb, liver, fried, grilled or BBQ'd, fat not further defined	0.00
08D10183	18403013	Lamb, tongue, raw	0.00
08D10184	18403014	Lamb, tongue, simmered or boiled, no added fat	0.00
08D10187	18403015	Veal, heart, raw	0.00
08D10188	18403016	Veal, heart, baked or roasted, no added fat	0.00
08D10189	18402005	Veal, kidney, raw	0.00
08D10190	18402006	Veal, kidney, fried, grilled or BBQ'd, no added fat	0.00
08D10192	18401008	Veal, liver, raw	0.00
08D10191	18401009	Veal, liver, fried, grilled or BBQ'd, no added fat	0.00
08E30346	18601003	Bacon, breakfast rasher, raw	0.00
08E30345	18601004	Bacon, breakfast rasher, baked, roasted or fried, no added fat	0.00
08E30344	18601005	Bacon, breakfast rasher, grilled or BBQ'd, no added fat	0.00
08E30386	18601001	Bacon, 97% fat free, raw	0.00
08E30391	18601002	Bacon, 97% fat free, baked, roasted, fried, grilled or BBQ'd, with or without added fat	0.00
08E30337	18601006	Bacon, middle rasher or shortcut, fully-trimmed, raw	0.00
08E30339	18601007	Bacon, middle rasher or shortcut, fully-trimmed, fried or stir-fried, no added fat	0.00
08E30342	18601008	Bacon, middle rasher or shortcut, fully-trimmed, baked, roasted, grilled or BBQ'd, no added fat	0.00
08E30366	18601009	Bacon, middle rasher or shortcut, fully-trimmed, baked, roasted, fried, grilled or BBQ'd, butter, dairy blend or margarine spread	0.00
08E30387	18601010	Bacon, middle rasher or shortcut, fully-trimmed, baked, roasted, fried, stir-fried, grilled or BBQ'd, canola oil	0.00
08E30368	18601011	Bacon, middle rasher or shortcut, fully-trimmed, baked, roasted, fried, grilled or BBQ'd, olive oil	0.00
08E30388	18601012	Bacon, middle rasher or shortcut, fully-trimmed, baked, roasted, fried, stir-fried, grilled or BBQ'd, other oil	0.00

08E30370	18601013	Bacon, middle rasher or shortcut, fully-trimmed, baked, roasted, fried, grilled or BBQ'd, fat not further defined	0.00
08E30336	18601014	Bacon, middle rasher, semi-trimmed, raw	0.00
08E30340	18601015	Bacon, middle rasher, semi-trimmed, fried or stir-fried, no added fat	0.00
08E30338	18601016	Bacon, middle rasher, semi-trimmed, baked, roasted, grilled or BBQ'd, no added fat	0.00
08E30373	18601017	Bacon, middle rasher, semi-trimmed, baked, roasted, fried, grilled or BBQ'd, butter, dairy blend & margarine	0.00
08E30371	18601018	Bacon, middle rasher, semi-trimmed, baked, roasted, fried, grilled or BBQ'd, canola oil	0.00
08E30363	18601019	Bacon, middle rasher, semi-trimmed, baked, roasted, fried, grilled or BBQ'd, olive oil	0.00
08E30365	18601020	Bacon, middle rasher, semi-trimmed, baked, roasted, fried, stir-fried, grilled or BBQ'd, other oil	0.00
08E30372	18601021	Bacon, middle rasher, semi-trimmed, baked, roasted, fried, grilled or BBQ'd, fat not further defined	0.00
08E30380	18601022	Bacon, middle rasher, semi-trimmed, boiled, casseroled, microwaved, poached, steamed or stewed, no added fat	0.00
08E30343	18601023	Bacon, middle rasher, untrimmed, raw	0.00
08E30382	18601024	Bacon, middle rasher, untrimmed, baked, roasted, fried, grilled or BBQ'd, no added fat	0.00
08E30383	18601025	Bacon, middle rasher, untrimmed, baked, roasted, fried, grilled or BBQ'd, fat not further defined	0.00
08E30385	18601026	Bacon, middle rasher, untrimmed, boiled, casseroled, microwaved, poached, steamed or stewed, no added fat	0.00
08E30341	18601027	Bacon, middle rasher, fat only, grilled or BBQ'd	0.00
08E30362	18601028	Bacon, raw, used in baked products, not further defined	0.00
08E30355	18604001	Beef, corned, lean, cooked	0.00
08E30359	18604002	Beef, corned, 50% trimmed, cooked	0.00
08E30360	18604003	Beef, corned, 75% trimmed, cooked	0.00
08E30358	18604004	Beef, corned, lean & fat, cooked	0.00
08E30335	18606002	Beef, corned, canned	0.00
08E30357	18606003	Beef, corned, with cereal, canned	0.00
08E30321	18604005	Berliner	0.00
08F10890	18606004	Braised steak & onions, canned, heated	0.00
08E30333	18604006	Brawn	0.00
08E30334	18606005	Camp pie, canned	0.00
13B10258	18606006	Casserole or curry, meat & vegetables, canned, heated	0.00
08E30364	18605001	Chicken, processed luncheon meat, regular fat	0.00
08E30356	18605002	Chicken, processed luncheon meat, low or reduced fat	0.00
08E30367	18605003	Chicken, processed luncheon meat, not further defined	0.00
08E30323	18604007	Devon, processed luncheon meat	0.00

08E20136	18606007	Frankfurt, canned, heated, drained	0.00
08E20125	18502001	Frankfurt, cooked	0.00
08E30331	18604008	Garlic roll	0.00
08E30349	18602001	Ham, leg, lean	0.00
08E30350	18602002	Ham, leg, lean & fat	0.00
08E30351	18602003	Ham, leg, lean & fat, canned	0.00
08E30352	18602004	Ham, shoulder, lean & fat	0.00
08E30353	18602005	Ham, shoulder, lean & fat, canned	0.00
08E30347	18602006	Ham steak, raw	0.00
08E30348	18602007	Ham steak, baked, roasted, fried, grilled or BBQ'd, no added fat	0.00
08E30389	18602008	Ham steak, baked, roasted, fried, grilled or BBQ'd, fat not further defined	0.00
08E30330	18604009	Ham & chicken roll, processed luncheon meat	0.00
08E30378	18604013	Ham & salami, for use as subway sandwich filling	0.00
08E30381	18607001	Jerky, beef, all flavours	0.00
08E30332	18603001	Kabana or cabanossi	0.00
08E40024	18606008	Meat paste, commercial	0.00
08E30324	18604010	Mortadella, processed meat	0.00
08E40023	18404001	Pate de foie (chicken liver pate)	0.00
08E40022	18404002	Pate, liverwurst, commercial	0.00
02E60370	13405034	Pie, steak & kidney, canned	0.00
08E20127	18604011	Polish sausage	0.00
08E30392	18604014	Processed meat, for use in garden salads	0.00
08E30361	18602009	Prosciutto	0.00
08E30325	18603002	Salami, danish	0.00
08E30326	18603003	Salami, hungarian	0.00
08E30327	18603004	Salami, mettwurst	0.00
08E30328	18603005	Salami, milano	0.00
08E30320	18603006	Salami, pepperoni	0.00
08E30329	18603007	Salami, not further defined	0.00
08E20128	18501001	Sausage, beef, raw	0.00
08E20130	18501002	Sausage, beef, fried	0.00
08E20129	18501003	Sausage, beef, grilled, BBQ'd or baked	0.00
08E20139	18501004	Sausage, beef, flavoured, fried, grilled, BBQ'd or baked	0.00
08E20144	18501005	Sausage, beef, plain or flavoured, boiled, casseroled, microwaved, poached, steamed, or stewed	0.00
08E20175	18503001	Sausage, beef, plain or flavoured, reduced fat, raw	0.00
08E20176	18503002	Sausage, beef, plain or flavoured, reduced fat, fried, grilled, BBQ'd or baked	0.00
08E20177	18503003	Sausage, beef, plain or flavoured, reduced fat, boiled, casseroled, microwaved, poached, steamed or stewed	0.00
08E20137	18503004	Sausage, chicken, plain, fried, grilled, BBQ'd or baked, with or without added fat	0.00
08E20142	18503005	Sausage, chicken, flavoured, fried, grilled, BBQ'd or baked, with or without fat	0.00
08E20150	18503006	Sausage, chicken, reduced fat, raw	0.00

08E20151	18503007	Sausage, chicken, reduced fat, fried, grilled, BBQ'd or baked, no added fat	0.00
08E20163	18503008	Sausage, chicken, reduced fat, fried, grilled, BBQ'd or baked, fat not further defined	0.00
08E30375	18603008	Sausage, chorizo, uncooked	0.00
08E30377	18603009	Sausage, chorizo, cooked	0.00
08E20179	18503009	Sausage, kangaroo, plain or flavoured, fried, grilled, BBQ'd or baked	0.00
08E20140	18501006	Sausage, lamb, plain, fried, grilled, BBQ'd or baked	0.00
08E20141	18501007	Sausage, lamb, flavoured, fried, grilled, BBQ'd or baked	0.00
08E20131	18501008	Sausage, pork, raw	0.00
08E20133	18501009	Sausage, pork, plain, fried	0.00
08E20132	18501010	Sausage, pork, plain, grilled, BBQ'd or baked	0.00
08E20138	18501011	Sausage, pork, flavoured, fried, grilled, BBQ'd or baked	0.00
08E20169	18501012	Sausage, pork, plain or flavoured, boiled, casseroled, microwaved, poached, steamed or stewed	0.00
08E20172	18501014	Sausage, fried, grilled, BBQ'd or baked	0.00
08E20178	18501015	Sausage, boiled, casseroled, microwaved, poached, steamed or stewed	0.00
08E20135	18501013	Sausage, deep fried, commercial	0.00
08E20154	18501016	Sausage, for use on sandwiches, not further defined	0.00
08E20126	18801004	Saveloy, battered, deep fried, oil not further defined	0.00
08E30354	18606009	Spam, canned	0.00
08F11012	18606010	Stew, casserole or curry, chicken & vegetable, canned	0.00
08E30322	18604012	Strasburg	0.00
08E30319	18605004	Turkey, roast, deli-sliced	0.00
08E30318	18605005	Turkey, processed luncheon meat	0.00
13B10256	18606011	Vegetables & sausages, canned	0.00
13B10257	18606012	Vegetables & steak, canned	0.00
11B10216	22203006	Coconut, cream, regular fat	0.00
11B10210	22203001	Coconut, fresh, mature fruit, flesh	0.00
11B10213	22203002	Coconut, fresh, young or immature, flesh	0.00
11B10212	22203003	Coconut, fresh, mature, water or juice	0.00
11B10214	22203004	Coconut, fresh, young or immature, water or juice	0.00
11B10215	22203005	Coconut, grated & desiccated	0.00
11B10219	22203007	Coconut, milk, dried, powder	0.00
11B10217	22203008	Coconut, milk, canned, regular fat	0.00
11B10218	22203009	Coconut, milk, canned, reduced fat	0.00
11B10233	22203010	Coconut, milk, canned, not further defined	0.00
11B10232	22204002	Nut, almond meal	0.00
11B10238	22204003	Nut, almond, with or without skin, roasted, salted	0.00
11B10197	22204004	Nut, almond, with or without skin, raw, unsalted	0.00
11B10225	22204005	Nut, almond, with or without skin, roasted, unsalted	0.00
11B10198	22204006	Nut, almond, without skin, blanched, unsalted	0.00
11B10205	22204007	Nut, brazil, with or without skin, raw, unsalted	0.00
11B10199	22204008	Nut, cashew, raw, unsalted	0.00
11B10200	22204009	Nut, cashew, roasted, salted	0.00

11B10224	22204010	Nut, cashew, roasted, unsalted	0.00
11B10258	22204011	Nut, cashew, roasted, coated in honey	0.00
11B10209	22204012	Nut, chestnut, raw, unsalted	0.00
11B10211	22204013	Nut, chestnut, roasted, unsalted	0.00
11B10202	22204014	Nut, hazelnut, with or without skin, raw, unsalted	0.00
11B10206	22204015	Nut, macadamia, raw, unsalted	0.00
11B10227	22204016	Nut, macadamia, roasted, salted	0.00
11B10243	22204017	Nut, macadamia, roasted, coated in honey	0.00
11B10194	22201001	Nut, peanut, with skin, raw or dry roasted, unsalted	0.00
11B10196	22201002	Nut, peanut, with skin, roasted, with oil, salted	0.00
11B10201	22201003	Nut, peanut, without skin, roasted, with oil, salted	0.00
11B10220	22201004	Nut, peanut, without skin, roasted, with oil, unsalted	0.00
11B10244	22202001	Nut, peanut, roasted, coated in honey	0.00
11B10203	22204018	Nut, pecan, raw, unsalted	0.00
11B10207	22204019	Nut, pine, raw, unsalted	0.00
11B10239	22204020	Nut, pistachio, roasted, salted	0.00
11B10204	22204021	Nut, pistachio, raw, unsalted	0.00
11B10195	22204022	Nut, walnut, raw, unsalted	0.00
11A10039	22101001	Seed, chia, dried	0.00
11A10038	22101003	Seed, linseed or flaxseed	0.00
11A10042	22101004	Seeds, mixed	0.00
11A10037	22101005	Seed, poppy	0.00
11A10036	22101006	Seed, pumpkin, hulled & dried, unsalted	0.00
11A10033	22101007	Seed, sesame, unsalted	0.00
11A10034	22101008	Seed, sunflower, unsalted	0.00
11A10041	22101010	Mixed seeds, for use in muesli recipes, unsalted	0.00
11A10035	22102004	Tahini, sesame seed pulp	0.00
11B10240	22205017	Mixed nuts, for use in muesli recipes, unsalted	0.00
11B10229	22205013	Mixed nuts, without peanuts, raw, unsalted	0.00
11B10231	22205004	Mixed nuts, cashew & macadamia, salted	0.00
11B10251	22205001	Mixed nuts, almond, cashew, peanut, salted	0.00
11B10252	22205002	Mixed nuts, almond, cashew, peanut, unsalted	0.00
11B10253	22205003	Mixed nuts, almond, cashew, walnut, unsalted	0.00
11B10254	22205005	Mixed nuts, peanut & 2 or fewer other nuts, salted	0.00
11B10255	22205006	Mixed nuts, peanut & 2 or fewer other nuts, unsalted	0.00
11B10256	22205007	Mixed nuts, three or fewer, without peanuts, salted	0.00
11B10257	22205008	Mixed nuts, three or fewer, without peanuts, unsalted	0.00
11B10247	22205009	Mixed nuts, peanut & 3 or more other nuts, salted	0.00
11B10248	22205010	Mixed nuts, peanut & 3 or more other nuts, unsalted	0.00
11B10246	22205011	Mixed nuts, four or more nuts, without peanuts, salted	0.00
11B10249	22205012	Mixed nuts, four or more nuts, without peanuts, unsalted	0.00
10D10175	16803001	Mixed nuts & dried fruit, with peanuts	0.00
10D10174	16803002	Mixed nuts & dried fruit, without peanuts	0.00
11A10044	22205014	Mixed nuts & seeds, with peanut	0.00
11A10043	22205015	Mixed nuts & seeds, without peanut	0.00

11A10040	22205016	Mixed nuts & seeds, almond, linseed & sunflower seed (LSA), unsalted	0.00
11B10242	22205018	Mixed nuts & seeds, unsalted, for use in muesli bar recipes	0.00
11B10259	22205019	Mixed nuts & seeds, for use in garden salad recipes	0.00
10D10176	16803003	Mixed nuts, dried fruit & seeds (trail mix), with peanuts	0.00
10D10164	16803004	Mixed nuts, dried fruit & seeds (trail mix), without peanuts	0.00
08F11039	18701002	Casserole, homemade, beef, homemade gravy	0.00
08F11138	18701036	Casserole, homemade, beef, homemade gravy	0.00
08F11040	18701003	Casserole, homemade, beef, homemade tomato based sauce	0.00
08F11091	18702001	Casserole, homemade, beef, homemade tomato based sauce, with rice or pasta	0.00
08F11096	18701004	Casserole, homemade, beef, kidney & vegetable, homemade gravy	0.00
08F10965	18701005	Casserole, homemade, beef & mushroom, homemade cream based sauce (stroganoff)	0.00
08F11042	18702002	Casserole, homemade or commercial, beef & mushroom, homemade cream based sauce, with rice or pasta	0.00
08F10966	18701001	Casserole, commercial, beef & vegetable	0.00
08F11044	18701006	Casserole, homemade, beef & vegetable, commercial sauce	0.00
08F11009	18701007	Casserole, homemade, beef & vegetable, homemade cream based sauce	0.00
08F11010	18702003	Casserole, homemade, beef & vegetable, homemade cream based sauce, with rice or pasta	0.00
08F10948	18701008	Casserole, homemade, beef & vegetables, homemade gravy	0.00
08F11139	18701037	Casserole or curry, homemade, beef & vegetable, homemade gravy	0.00
08F11008	18702004	Casserole, homemade, beef & vegetable, homemade gravy, with rice or pasta	0.00
08F11151	18702020	Casserole or curry, homemade, beef & vegetable, homemade gravy, with rice or pasta	0.00
08F10947	18701009	Casserole, homemade, beef & vegetable, homemade tomato based sauce	0.00
08F11131	18702005	Casserole, homemade, beef & vegetable, homemade tomato based sauce, with rice or pasta	0.00
08F10969	18701010	Casserole, homemade, beef, vegetable & legume, homemade sauce	0.00
08F11041	18702006	Casserole, homemade, beef, vegetable & legume, homemade sauce, with rice or pasta	0.00
08F11140	18803002	Casserole or curry, homemade, canned meat & vegetable, homemade gravy	0.00
08F11088	18901001	Casserole, commercial, chicken	0.00
08F11141	18901032	Casserole or curry, homemade, chicken, homemade gravy	0.00

08F11067	18901002	Casserole, commercial, chicken & vegetable	0.00
08F11068	18902001	Casserole, commercial, chicken & vegetable, with rice or pasta	0.00
08F10951	18901003	Casserole, homemade, chicken & vegetable, homemade gravy	0.00
08F11142	18901033	Casserole or curry, homemade, chicken & vegetable, homemade gravy	0.00
08F11003	18902002	Casserole, homemade, chicken & vegetable, homemade gravy, with rice or pasta	0.00
08F11152	18902026	Casserole or curry, homemade, chicken & vegetable, homemade gravy, with rice or pasta	0.00
08F11069	18901004	Casserole, homemade, chicken & vegetable, homemade cream based sauce	0.00
08F11070	18902003	Casserole, homemade, chicken & vegetable, homemade cream based sauce, with rice or pasta	0.00
08F11071	18901005	Casserole, homemade, chicken & vegetable, homemade fruit sauce	0.00
08F11092	18902004	Casserole, homemade, chicken & vegetable, homemade fruit sauce, with rice or pasta	0.00
08F10950	18901006	Casserole, homemade, chicken & vegetable, homemade tomato based sauce	0.00
08F11099	18902005	Casserole, homemade, chicken & vegetable, homemade tomato based sauce, with rice or pasta	0.00
08F11072	18901007	Casserole, homemade, duck, homemade sauce	0.00
13B20290	24901001	Casserole, homemade, eggplant, homemade tomato based sauce	0.00
05D10411	15601001	Casserole, homemade, fish, homemade tomato & cream sauce	0.00
05D10430	15601012	Casserole or curry, homemade, fish, homemade gravy	0.00
05D10431	15601013	Casserole or curry, homemade, fish & vegetables, homemade gravy	0.00
08B10089	18711001	Casserole, homemade, kangaroo, homemade sauce	0.00
08B10099	18012001	Casserole, homemade, kangaroo & vegetable, homemade sauce	0.00
08F10954	18705001	Casserole, homemade, lamb, homemade gravy	0.00
08F11144	18705022	Casserole or curry, homemade, lamb, homemade gravy	0.00
08F10974	18705002	Casserole, homemade, lamb & eggplant, baked with homemade creamy sauce (moussaka)	0.00
08F11137	18705021	Casserole, homemade, lamb, offal & vegetable, homemade gravy	0.00
08F11046	18705003	Casserole, commercial, lamb & vegetables	0.00
08F11045	18705004	Casserole, homemade, lamb & vegetable, homemade gravy	0.00
08F11145	18705023	Casserole or curry, homemade, lamb & vegetable, homemade gravy	0.00
08F10953	18705005	Casserole, homemade, lamb & vegetable, homemade tomato based sauce	0.00
08F11047	18706001	Casserole, homemade, lamb & vegetable, tomato based sauce, with rice or pasta	0.00

08F11048	18705006	Casserole, homemade, lamb, vegetable & legume, homemade sauce	0.00
05D20134	15603001	Casserole, homemade, mussel & vegetable, homemade sauce	0.00
08F11146	18708012	Casserole or curry, homemade, pork & vegetable, homemade gravy	0.00
08F10956	18708001	Casserole, homemade, pork & vegetable, homemade tomato based sauce	0.00
08F11095	18711002	Casserole, homemade, rabbit, vegetable & legume, homemade sauce	0.00
05D10393	15601003	Casserole, homemade, salmon or tuna & vegetable, homemade sauce	0.00
05D10417	15602001	Casserole, homemade, salmon or tuna & vegetable, homemade sauce, with rice or pasta	0.00
05D10418	15601002	Casserole, homemade, salmon or tuna & vegetable, commercial sauce	0.00
05D10424	15602002	Casserole, homemade, salmon or tuna & vegetable, commercial sauce, with rice or pasta	0.00
05D10392	15601004	Casserole, homemade, salmon or tuna & vegetable, homemade dairy based sauce	0.00
08F10957	18801001	Casserole, homemade, sausage & vegetable, homemade tomato based sauce	0.00
08F11123	18802001	Casserole, homemade, sausage & vegetable, homemade sauce, with rice or pasta	0.00
08F10990	18701011	Casserole, homemade, veal & vegetable, homemade gravy	0.00
08F11089	24901002	Casserole, commercial, vegetable	0.00
13B10317	24901003	Casserole, homemade, vegetable, homemade gravy	0.00
13B10338	24901027	Casserole or curry, homemade, vegetable, homemade gravy	0.00
13B10288	24901004	Casserole, homemade, vegetable, homemade tomato based sauce	0.00
13B20267	25202002	Casserole, homemade, vegetable & legumes, homemade tomato based sauce	0.00
13B20291	25202003	Casserole, homemade, vegetable & tofu, homemade tomato based sauce	0.00
13B20292	25202004	Casserole, homemade, vegetable & tofu, homemade tomato based sauce, with rice or pasta	0.00
08F11097	18711003	Casserole, homemade, venison & vegetable, homemade gravy	0.00
15A20149	18012002	Casserole or stew, homemade, wild caught meat & vegetable, homemade gravy	0.00
08F10988	18903025	Chicken, battered, takeaway, restaurant or cafe style, with honey & lemon sauce	0.00
02F40373	13515003	Couscous, homemade, cooked, with roast vegetables	0.00
02F40415	13515004	Couscous, homemade, cooked with roast vegetables & meat	0.00
08F11001	18702009	Curry or casserole, homemade, beef & pasta, not further defined	0.00

08F10970	18702010	Curry or casserole, homemade, beef & rice, not further defined	0.00
08F11006	18701020	Curry or casserole, commercial, beef & vegetables, not further defined	0.00
08F11007	18702011	Curry or casserole, commercial, beef & vegetables, with rice, not further defined	0.00
13B20271	25202008	Curry or casserole, homemade, chick pea, homemade sauce	0.00
08F10933	18701012	Curry, commercial, beef, coconut milk based sauce	0.00
08F10932	18701013	Curry, commercial, beef, tomato based sauce	0.00
08F10908	18701014	Curry, commercial, beef, vindaloo sauce	0.00
08F10930	18701015	Curry, homemade, beef, commercial simmer sauce, Indian style	0.00
08F10928	18701016	Curry, homemade, beef, homemade sauce	0.00
08F11015	18701017	Curry, homemade, beef & vegetable, commercial sauce	0.00
08F11101	18702007	Curry, homemade, beef & vegetable, commercial sauce, with rice or noodles	0.00
08F10931	18701018	Curry, homemade, beef & vegetable, homemade coconut milk based sauce	0.00
08F11049	18702008	Curry, homemade, beef & vegetable, homemade coconut milk based sauce, with rice or noodles	0.00
08F10929	18701019	Curry, homemade, beef & vegetable, homemade tomato based sauce	0.00
08F10942	18901008	Curry, commercial, chicken, dairy based sauce, Indian	0.00
08F10941	18901009	Curry, homemade, chicken, commercial sauce	0.00
08F11073	18902007	Curry, homemade, chicken, commercial sauce, with rice or noodles	0.00
08F11106	18901010	Curry, homemade, chicken, homemade sauce	0.00
08F11074	18901011	Curry, homemade, chicken, homemade coconut milk sauce	0.00
08F11075	18902008	Curry, homemade, chicken, homemade coconut milk sauce, with rice or noodles	0.00
08F10985	18902009	Curry, homemade, chicken & rice	0.00
08F10852	18901012	Curry, commercial, chicken & vegetable, coconut milk sauce	0.00
08F11076	18901013	Curry, homemade, chicken & vegetable, commercial sauce	0.00
08F11105	18902010	Curry, homemade, chicken & vegetable, commercial sauce, with rice or noodles	0.00
08F10939	18901014	Curry, homemade, chicken & vegetable, homemade coconut milk sauce	0.00
08F11143	18901034	Curry, homemade, chicken & vegetable, homemade coconut milk sauce	0.00
08F11077	18902011	Curry, homemade, chicken & vegetable, homemade coconut milk sauce, with rice or noodles	0.00
08F10940	18901015	Curry, homemade, chicken & vegetable, homemade dairy based sauce	0.00

08F11002	18902012	Curry, homemade, chicken & vegetable, homemade dairy based sauce, with rice or noodles	0.00
08F11078	18901016	Curry, homemade, chicken & vegetable, homemade tomato based sauce	0.00
08F11079	18902013	Curry, homemade, chicken & vegetable, homemade tomato based sauce, with rice or noodles	0.00
08F11108	18901017	Curry, homemade, chicken, vegetable & legume, homemade sauce	0.00
08F11109	18902014	Curry, homemade, chicken, vegetable & legume, homemade sauce, with rice or noodles	0.00
13B20293	25202005	Curry, homemade, chick pea & vegetable, homemade sauce, with rice or noodles	0.00
05D10410	15601005	Curry, homemade, fish, homemade sauce	0.00
05D10413	15602003	Curry, homemade, fish, homemade sauce, with rice or noodles	0.00
05D10395	15601006	Curry, homemade, fish, homemade coconut milk sauce	0.00
05D10397	15602004	Curry, homemade, fish & rice	0.00
05D10396	15601007	Curry, commercial, fish & vegetable, coconut milk sauce	0.00
05D10391	15601008	Curry, homemade, fish & vegetable, homemade coconut milk sauce	0.00
05D10412	15601009	Curry, homemade, fish & vegetable, homemade tomato based sauce	0.00
05D10416	15602005	Curry, homemade, fish & vegetable, homemade tomato based sauce, with rice or noodles	0.00
08F11100	18711004	Curry, homemade, goat, homemade sauce	0.00
08F11128	18711005	Curry, homemade, goat, homemade sauce, with rice or noodles	0.00
08F10901	18705007	Curry, commercial, lamb, dairy based sauce	0.00
08F10934	18705008	Curry, homemade, lamb, homemade sauce	0.00
08F10975	18706002	Curry, homemade, lamb & rice	0.00
08F10936	18705009	Curry, commercial, lamb & vegetable, dairy based sauce	0.00
08F11111	18705010	Curry, homemade, lamb & vegetable, homemade coconut milk sauce	0.00
08F10935	18705011	Curry, homemade, lamb & vegetable, homemade dairy based sauce	0.00
08F11112	18706003	Curry, homemade, lamb & vegetable, homemade dairy based sauce, with rice or noodles	0.00
08F11050	18705012	Curry, homemade, lamb & vegetable, homemade tomato based sauce	0.00
08F10971	18705013	Curry, homemade, lamb, vegetable & legume, homemade tomato based sauce	0.00
08F10905	25202006	Curry, commercial, legume (dhal)	0.00
13B20270	25202007	Curry, homemade, legume (dhal)	0.00
13B10323	24901005	Curry, homemade, okra, homemade sauce	0.00
13B10329	24901006	Curry, homemade, okra, with rice or noodles	0.00
08F10937	18708002	Curry, commercial, pork, coconut milk sauce	0.00

08F11052	18708003	Curry, homemade, pork, coconut milk sauce	0.00
08F11053	18708004	Curry, homemade, pork & vegetable, coconut milk sauce	0.00
08F10903	24901007	Curry, commercial, potato & pea (aloo muttar)	0.00
13A12557	24901008	Curry, homemade, potato, dairy based sauce	0.00
05D20137	15604001	Curry, commercial, prawn & vegetable, coconut milk sauce, with rice	0.00
05D20136	15603002	Curry, commercial, prawn & vegetable, tomato based sauce	0.00
05D20132	15603003	Curry, homemade, prawn & vegetable, homemade coconut milk sauce	0.00
05D20133	15603004	Curry, homemade, prawn & vegetable, homemade tomato based sauce	0.00
05D20144	15604002	Curry, homemade, prawn & vegetable, homemade tomato based sauce, with rice or noodles	0.00
13A12639	24901028	Curry, homemade, root vegetable, homemade coconut milk sauce	0.00
08F10938	18801002	Curry, homemade, sausage, homemade sauce	0.00
08F10904	24901009	Curry, commercial, spinach & cheese (palak paneer)	0.00
13B20262	20701001	Curry, commercial, tofu & vegetable	0.00
13A12221	24901010	Curry, commercial, vegetable, cream based sauce	0.00
13B10322	24901011	Curry, homemade, vegetables, commercial sauce	0.00
13A12219	24901012	Curry, homemade, vegetable, homemade sauce	0.00
13A12350	24901013	Curry, homemade, vegetable, homemade coconut milk sauce	0.00
13A12351	24901014	Curry, homemade, vegetable, homemade dairy based sauce	0.00
13A12567	24901015	Curry, homemade, vegetable & egg, homemade dairy based sauce	0.00
13B10318	24901016	Curry, homemade, vegetable, homemade sauce, with rice or noodles	0.00
13B10282	24901017	Curry, homemade, vegetable & legume, homemade sauce	0.00
08F10999	13509004	Frozen meal, beef lasagne, energy controlled portion	0.00
08F10995	18702012	Frozen meal, beef & noodles or pasta, energy controlled portion	0.00
08F11085	18702013	Frozen meal, beef & rice, energy controlled portion	0.00
08F10949	18701021	Frozen meal, beef & vegetables, energy controlled portion	0.00
08F10952	18901018	Frozen meal, chicken & vegetables, energy controlled portion	0.00
08F10998	18902015	Frozen meal, chicken, vegetables & noodles or pasta, energy controlled portion	0.00
08F10946	18902016	Frozen meal, curry, chicken & rice, coconut milk sauce	0.00
08F10945	18902017	Frozen meal, curry, chicken & rice, Indian style	0.00
08F10944	18902018	Frozen meal, curry, chicken & rice, energy controlled portion	0.00
08F11000	18705014	Frozen meal, lamb & vegetables, energy controlled portion	0.00

02F40347	13507004	Frozen meal, Mexican wrap, beef, rice & vegetables, energy controlled portion	0.00
08F10996	13509005	Frozen meal, pasta with bolognese sauce	0.00
08F11014	18709001	Frozen meal, pork & vegetables with rice	0.00
05C10201	15604003	Frozen meal, prawn & rice	0.00
08F11084	18701022	Frozen meal, roast beef & vegetables	0.00
08F10997	18901019	Frozen meal, roast chicken with vegetables	0.00
08F11087	18801003	Frozen meal, sausage & vegetables	0.00
02F40277	15602006	Frozen meal, tuna mornay, with pasta & white sauce	0.00
08F11013	18703009	Frozen meal, veal schnitzel with cheese & potato	0.00
08F11086	18902019	Frozen meal, vegetables & pasta or noodles, energy controlled portion	0.00
08F11055	18712001	Meatloaf, commercial, all meats	0.00
08F10992	18703021	Meatloaf, homemade, beef	0.00
08F11066	18903052	Meatloaf, homemade, chicken	0.00
02F30327	13505059	Mixed dish, cheeseburger, potato fries & soft drink, fast food style	0.00
08F11054	18709002	Mixed dish, homemade, pork & rice, not further defined	0.00
08F11125	18712002	Mixed dish, meat & egg, grilled or fried	0.00
02A10504	13515005	Mixed dish, polenta, with added tomato based sauce & cheese	0.00
08F11124	18712003	Mixed dish, not further defined	0.00
02F40416	13509012	Noodles, fried or stir-fried, not further defined, with sauce	0.00
08F11098	18903053	Roast chicken, with stuffing & homemade gravy	0.00
08F11056	18705015	Roast dinner, commercial or homemade, lamb, vegetables & gravy	0.00
08F11057	18706004	Roast dinner, commercial or homemade, lamb, vegetables, gravy & grains	0.00
08F11058	18708005	Roast dinner, commercial or homemade, pork, vegetables & gravy	0.00
08F11114	18709003	Roast pork, with rice, homemade	0.00
13B10306	24901020	Stir-fry, homemade, green vegetable, homemade sauce	0.00
08F11059	18701027	Stir-fry, commercial, beef	0.00
08F11126	18702014	Stir-fry, commercial, beef, with noodles	0.00
08F10886	18701028	Stir-fry, commercial, beef, satay sauce	0.00
08F11061	18701031	Stir-fry, homemade, beef	0.00
08F11132	18702016	Stir-fry, homemade, beef, with rice or noodles	0.00
08F10968	18701035	Stir-fry, homemade, beef, commercial or homemade satay sauce	0.00
08F10876	18701029	Stir-fry, commercial, beef & vegetable	0.00
08F11060	18702015	Stir-fry, commercial, beef & vegetable, with noodles	0.00
08F10961	18701032	Stir-fry, homemade, beef & vegetable	0.00
08F11147	18701038	Stir-fry, homemade, beef & vegetable	0.00
08F10963	18701034	Stir-fry, homemade, beef & vegetable, commercial simmer sauce	0.00

08F10964	18702017	Stir-fry, homemade, beef & vegetable, with noodles	0.00
08F11011	18702018	Stir-fry, homemade, beef & vegetable, with rice	0.00
08F11153	18702021	Stir-fry, homemade, beef & vegetable, with rice	0.00
08F11062	18701033	Stir-fry, homemade, beef, vegetable & egg	0.00
08F11063	18702019	Stir-fry, homemade, beef, vegetable & egg, with rice or noodles	0.00
08F11016	18701030	Stir-fry, commercial, beef, vegetable & nut	0.00
08F11115	18901020	Stir-fry, commercial, chicken	0.00
08F10979	18901021	Stir-fry, commercial, chicken, satay sauce	0.00
08F11116	18902020	Stir-fry, commercial, chicken, with rice or noodles	0.00
08F11117	18901022	Stir-fry, homemade, chicken	0.00
08F10980	18901023	Stir-fry, homemade, chicken, commercial or homemade satay sauce	0.00
08F10982	18902021	Stir-fry, commercial, chicken, with noodles	0.00
08F10981	18902022	Stir-fry, homemade, chicken, with rice or noodles	0.00
08F10983	18901024	Stir-fry, commercial, chicken & vegetable	0.00
08F11150	18901035	Stir-fry, commercial, chicken & vegetable, satay sauce	0.00
02F40238	13509075	Stir-fry, commercial, noodles, added chicken & vegetable	0.00
08F10987	18901025	Stir-fry, homemade, chicken & vegetable, commercial simmer sauce	0.00
08F10958	18901026	Stir-fry, homemade, chicken & vegetable	0.00
08F11148	18901036	Stir-fry, homemade, chicken & vegetable	0.00
08F11103	18902023	Stir-fry, homemade, chicken & vegetable, commercial or homemade satay sauce, with rice or noodles	0.00
08F10960	18902024	Stir-fry, homemade, chicken & vegetable, with noodles	0.00
08F11081	18902025	Stir-fry, homemade, chicken & vegetable, with rice	0.00
08F10984	18901027	Stir-fry, commercial, chicken, vegetable & nut	0.00
08F10986	18901028	Stir-fry, homemade, chicken, vegetable & nut	0.00
08F11080	18901029	Stir-fry, commercial, chicken, vegetable & seaweed	0.00
05D20143	15603007	Stir-fry, homemade, crab	0.00
13B10307	24901018	Stir-fry, homemade, egg & vegetable	0.00
13B10309	24901019	Stir-fry, homemade, egg & vegetable, with rice or noodles	0.00
05D10421	15601010	Stir-fry, commercial, fish	0.00
05D10414	15601011	Stir-fry, homemade, fish & vegetable	0.00
05D10432	15601014	Stir-fry, homemade, fish & vegetable	0.00
05D10422	15602009	Stir-fry, homemade, fish & vegetable, with rice or noodles	0.00
08F10972	18705016	Stir-fry, commercial, lamb, mongolian sauce	0.00
08F11065	18705017	Stir-fry, commercial, lamb, satay sauce	0.00
08F11118	18705018	Stir-fry, homemade, lamb	0.00
08F11064	18705019	Stir-fry, commercial, lamb & vegetable	0.00
08F11120	18706005	Stir-fry, homemade, lamb & vegetable, with rice or noodles	0.00
05D20141	15603013	Stir-fry, homemade, seafood & vegetable	0.00
05D20150	15604005	Stir-fry, homemade, seafood & vegetable, with rice or noodles	0.00

08F10885	18708006	Stir-fry, commercial, pork, plum sauce	0.00
08F10889	18708007	Stir-fry, commercial, pork, sweet & sour sauce	0.00
08F10978	18708008	Stir-fry, commercial, pork & vegetable	0.00
08F11122	18708009	Stir-fry, homemade, pork	0.00
08F10977	18708010	Stir-fry, homemade, pork & vegetable, commercial simmer sauce	0.00
08F10976	18708011	Stir-fry, homemade, pork & vegetable	0.00
08F11149	18708013	Stir-fry, homemade, pork & vegetable	0.00
08F11121	18709004	Stir-fry, homemade, pork & vegetable, with rice or noodles	0.00
05D20124	15603008	Stir-fry, commercial, prawn, garlic sauce	0.00
05D20145	15603009	Stir-fry, commercial, prawn, satay sauce	0.00
05D20131	15603010	Stir-fry, homemade, prawn, garlic sauce	0.00
05D20140	15603011	Stir-fry, homemade, prawn & vegetable	0.00
05D20135	15604004	Stir-fry, homemade, prawn & vegetable, with rice or noodles	0.00
02F40225	13509077	Stir-fry, commercial, rice noodles, added prawn, egg & vegetable (pad Thai)	0.00
02F40226	13509078	Stir-fry, commercial, rice noodles, added prawn, pork, egg & vegetable (mee grob)	0.00
05D20139	15603012	Stir-fry, commercial, seafood (prawn, squid & fish) & vegetable	0.00
05D20142	15603014	Stir-fry, homemade, squid or calamari	0.00
13B20296	20701003	Stir-fry, homemade, tofu, commercial or homemade satay sauce	0.00
13B20294	20701004	Stir-fry, homemade, tofu & vegetable	0.00
02F40237	24901021	Stir-fry, commercial, vegetable	0.00
13B10287	13509076	Stir-fry, commercial, noodles, added vegetable	0.00
13B10285	24901022	Stir-fry, homemade, vegetable	0.00
13B10286	24901023	Stir-fry, homemade, vegetable, with noodles	0.00
13B10308	24901024	Stir-fry, homemade, vegetable, with rice	0.00
13B10324	24901025	Stir-fry, homemade, vegetable & nut or legume	0.00
13B10325	24901026	Stir-fry, homemade, vegetable & nut or legume, with rice or noodles	0.00
07C10132	32302001	Mixed dish, infant food, meat & vegetable, puree or mash	0.00
08F10902	18901030	Tandoori chicken, commercial	0.00
08F10989	18901031	Tandoori chicken, homemade, with commercial paste & yoghurt	0.00
08F10900	18705020	Tandoori lamb, commercial	0.00
05D20138	15603015	Tandoori prawn, homemade or commercial, with commercial paste & yoghurt	0.00
13B10310	23502003	Dip, beetroot, commercial	0.00
13B10326	23502004	Dip, beetroot, homemade	0.00
13B10327	23502005	Dip, carrot, commercial	0.00
13B20287	23502006	Dip, carrot, homemade	0.00
13B10312	23502007	Dip, capsicum, commercial, regular	0.00
13B10313	23502008	Dip, chilli & nut, commercial, regular	0.00

13B10314	23501001	Dip, crab, commercial, regular	0.00
09B30020	23501002	Dip, cream cheese based, flavoured, commercial	0.00
13B20280	23501003	Dip, cream cheesed based, flavoured, homemade	0.00
08F10916	23501004	Dip, cucumber & yoghurt, commercial	0.00
08F10917	23501005	Dip, cucumber & yoghurt, homemade	0.00
13B10274	23502009	Dip, eggplant, commercial	0.00
13B20245	23502010	Dip, eggplant, homemade	0.00
13B20238	23503001	Dip, hummus, commercial	0.00
13B20239	23503002	Dip, hummus, homemade	0.00
13B10277	23502011	Dip, olive, commercial	0.00
13B10275	23502012	Dip, pumpkin or sweet potato, commercial	0.00
13B10315	23501006	Dip, salmon, commercial, regular	0.00
09A50050	23501007	Dip, sour cream-based, commercial	0.00
13B20247	23501008	Dip, sour cream based, homemade	0.00
13B20275	23501009	Dip, spinach & fetta, commercial	0.00
13B10328	23502013	Dip, sundried tomato, commercial	0.00
13B20281	23501010	Dip, tomato &/or capsicum, homemade	0.00
05D10370	23504001	Dip, taramosalata, commercial	0.00
05D10371	23504002	Dip, taramosalata, homemade	0.00
02F40433	13514002	Sushi, beef, with seaweed	0.00
05D10427	13514001	Sushi, bacon & vegetable, with seaweed	0.00
05D10381	13514004	Sushi, chicken, with seaweed	0.00
02F40434	13514007	Sushi, crab stick, with seaweed	0.00
05D10426	13514008	Sushi, egg, with seaweed	0.00
05D10376	13514009	Sushi, fish, with seaweed	0.00
05D10387	13514010	Sushi, fish, without seaweed	0.00
05D10425	13514011	Sushi, fried bean curd (inari)	0.00
05D10378	13514012	Sushi, prawn, with seaweed	0.00
05D10394	13514013	Sushi, prawn, without seaweed	0.00
05D10389	13514014	Sushi, prawn tempura, with seaweed	0.00
05D10385	13514016	Sushi, salmon, with seaweed	0.00
05D10423	13514017	Sushi, salmon, without seaweed	0.00
05D10379	13514018	Sushi, tuna, with seaweed	0.00
05D10386	13514019	Sushi, tuna, without seaweed	0.00
13B10302	24902001	Cabbage roll, stuffed with meat & rice	0.00
13B10303	24902002	Cabbage roll, stuffed with tomato & rice	0.00
13B10300	24902003	Capsicum, stuffed with meat & rice	0.00
13B10301	24902004	Capsicum, stuffed with tomato & rice	0.00
13B10331	24902005	Mixed vegetables, stuffed with meat & rice	0.00
13B10305	24902006	Mushroom, stuffed with cheese & bacon	0.00
13B10304	24902007	Pepper, stuffed with cheese	0.00
13B10299	24902008	Vine leaf, stuffed with rice & meat, homemade from basic ingredients	0.00
13B10270	24902009	Vine leaf, stuffed with rice & tomato, commercial	0.00
13B10271	24902010	Vine leaf, stuffed with rice & tomato, homemade from basic ingredients	0.00
05A10644	15101001	Barramundi, raw	0.00

05A10739	15101002	Barramundi, baked, roasted, grilled, BBQ'd or fried, no added fat	0.00
05A10983	15101003	Barramundi, baked, roasted, grilled, BBQ'd, fried or deep fried, butter, dairy blend or margarine	0.00
05A10740	15101004	Barramundi, baked, roasted, grilled, BBQ'd, fried or deep fried, canola oil	0.00
05A10804	15101005	Barramundi, baked, roasted, grilled, BBQ'd, fried or deep fried, olive oil	0.00
05A10833	15101006	Barramundi, baked, roasted, grilled, BBQ'd, fried or deep fried, other oil	0.00
05A10785	15101007	Barramundi, baked, roasted, grilled, BBQ'd, fried or deep fried, fat not further defined	0.00
05A10719	15101008	Barramundi, boiled, microwaved, steamed or poached, no added fat	0.00
05A10742	15501001	Barramundi, coated, baked, roasted, fried, deep fried, grilled or BBQ'd, fat not further defined	0.00
05A10764	15501002	Barramundi, coated, takeaway outlet, deep fried	0.00
05A10715	15101009	Bassa (basa), raw	0.00
05A10805	15101010	Bassa (basa), baked, roasted, grilled, BBQ'd or fried, no added fat	0.00
05A10875	15101011	Bassa (basa), baked, roasted, grilled, BBQ'd, fried or deep fried, fat not further defined	0.00
05A10726	15101012	Bassa (basa), boiled, microwaved, steamed or poached, with or without added fat	0.00
05A10876	15501003	Bassa (basa), coated, baked, roasted, fried, deep fried, grilled or BBQ'd, fat not further defined	0.00
05A10744	15101013	Blue grenadier (hoki), raw	0.00
05A10688	15101014	Blue grenadier (hoki), baked, roasted, fried, grilled or BBQ'd, no added fat	0.00
05A10885	15101015	Blue grenadier (hoki), baked, roasted, grilled, BBQ'd, fried or deep fried, fat not further defined	0.00
05A10887	15101016	Blue grenadier (hoki), boiled, microwaved, steamed or poached, with or without added fat	0.00
05A10743	15501004	Blue grenadier (hoki), coated, baked, roasted, grilled, BBQ'd, fried or deep fried, fat not further defined	0.00
05A10774	15501006	Blue grenadier (hoki), coated, takeaway outlet, deep fried	0.00
05A10779	15501005	Blue grenadier (hoki), coated, packaged frozen, baked, roasted, fried, grilled or BBQ'd, with or without added fat	0.00
05A10928	15101017	Blue-eye trevalla, flesh, raw	0.00
05A10931	15101018	Blue-eye trevalla, baked, roasted, grilled, BBQ'd, fried or deep fried, fat not further defined	0.00
05A10932	15501007	Blue-eye trevalla, coated, takeaway outlet, deep fried	0.00
05A10646	15101019	Bream, raw	0.00
05A10720	15101020	Bream, baked, roasted, fried, grilled or BBQ'd, no added fat	0.00
05A10867	15101021	Bream, baked, roasted, grilled, BBQ'd, fried or deep fried, fat not further defined	0.00

05A10645	15101022	Bream, boiled, microwaved, steamed or poached, with or without added fat	0.00
05A10750	15101023	Cod, Atlantic, flesh, raw	0.00
05A10751	15101024	Cod, Pacific, flesh, raw	0.00
05A10752	15101025	Cod or hake, raw	0.00
05A10853	15101026	Cod or hake, baked, roasted, fried, grilled or BBQ'd, no added fat	0.00
05A10848	15101027	Cod or hake, baked, roasted, grilled, BBQ'd, fried or deep fried, fat not further defined	0.00
05A10854	15101028	Cod or hake, boiled, microwaved, steamed or poached, with or without added fat	0.00
05A10753	15501008	Cod or hake, coated, baked, roasted, grilled, BBQ'd, fried or deep fried, fat not further defined	0.00
05A10846	15501010	Cod or hake, coated, takeaway outlet, deep fried	0.00
05A10844	15501009	Cod or hake, coated, packaged frozen, baked, roasted, fried, grilled or BBQ'd, with or without added fat	0.00
15A20134	18011004	Dugong, wild caught, flesh, raw	0.00
15A20140	18011005	Dugong, wild caught, flesh, cooked	0.00
05A10648	15101029	Flathead, raw	0.00
05A10827	15101030	Flathead, baked, roasted, fried, grilled or BBQ'd, no added fat	0.00
05A10829	15101031	Flathead, baked, roasted, grilled, BBQ'd, fried or deep fried, fat not further defined	0.00
05A10647	15101032	Flathead, boiled, microwaved, steamed or poached, with or without added fat	0.00
05A10894	15501011	Flathead, coated, baked, roasted, fried, deep-fried, grilled or BBQ'd, fat not further defined	0.00
05A10889	15501012	Flathead, coated, takeaway outlet, deep fried	0.00
05A10949	15101033	Flounder, raw	0.00
05A10950	15101034	Flounder, baked, roasted, fried, grilled or BBQ'd, no added fat	0.00
05A10952	15101035	Flounder, baked, roasted, grilled, BBQ'd, fried or deep fried, fat not further defined	0.00
05A10943	15101036	Garfish, raw	0.00
05A10944	15501013	Garfish, coated, takeaway outlet, deep fried	0.00
05A10649	15101037	Gemfish, raw	0.00
05A10682	15101038	Gemfish, boiled, microwaved, steamed or poached, no added fat	0.00
05A10937	15101039	Grouper, raw	0.00
05A10938	15101040	Grouper, baked, roasted, fried, grilled or BBQ'd, no added fat	0.00
05A10939	15101041	Grouper, baked, roasted, fried, grilled or BBQ'd, fat not further defined	0.00
05A10942	15501014	Grouper, coated, baked, roasted, grilled, or BBQ'd, fat not further defined	0.00
05A10945	15101042	John dory, raw	0.00
05A10946	15101043	John dory, baked, roasted, fried, grilled or BBQ'd, no added fat	0.00

05A10947	15101044	John dory, baked, roasted, grilled, BBQ'd, fried or deep fried, fat not further defined	0.00
05A10948	15501015	John dory, coated, baked, roasted, fried, or BBQ'd, fat not further defined	0.00
05A10926	15101045	Ling, raw	0.00
05A10929	15101046	Ling, baked, roasted, fried, grilled or BBQ'd, no added fat	0.00
05A10930	15101047	Ling, baked, roasted, grilled, BBQ'd, fried or deep fried, fat not further defined	0.00
05A10984	15501016	Ling, coated, baked, roasted, grilled, BBQ'd, fried or deep fried, fat not further defined	0.00
05A10755	15101048	Mackerel, raw	0.00
05A10906	15101049	Mackerel, baked, roasted, fried, grilled or BBQ'd, no added fat	0.00
05A10955	15101050	Mackerel, baked, roasted, grilled, BBQ'd, fried or deep fried, fat not further defined	0.00
05A10806	15501017	Mackerel, coated, takeaway outlet, deep fried	0.00
05A10987	15401003	Mackerel, canned	0.00
05A10717	15101051	Milkfish, raw	0.00
05A10729	15101052	Milkfish, boiled, microwaved, steamed or poached, with or without added fat	0.00
05A10683	15101053	Morwong, raw	0.00
05A10684	15101054	Morwong, boiled, microwaved, steamed or poached, with or without added fat	0.00
05A10651	15101055	Mullet, raw	0.00
05A10818	15101056	Mullet, baked, roasted, grilled, BBQ'd, fried or deep fried, fat not further defined	0.00
05A10685	15101057	Mullet, boiled, microwaved, steamed or poached, with or without added fat	0.00
05A10954	15501018	Mullet, coated, takeaway outlet, deep fried	0.00
05A10650	15101058	Mulloway, raw	0.00
05A10721	15101059	Mulloway, baked, roasted, grilled, BBQ'd, fried or deep fried, fat not further defined	0.00
05A10686	15101060	Mulloway, boiled, microwaved, steamed or poached, with or without added fat	0.00
05A10925	15501019	Mulloway, coated, fried or deep fried, fat not further defined	0.00
05A10915	15101061	Nile perch, baked, roasted, fried, grilled or BBQ'd, no added fat	0.00
05A10960	15101062	Nile perch, baked, roasted, grilled, BBQ'd, fried or deep fried, fat not further defined	0.00
05A10961	15101063	Nile perch, boiled, microwaved, steamed or poached, with or without added fat	0.00
05A10985	15501020	Nile perch, coated, baked, roasted, grilled, BBQ'd, fried or deep fried, fat not further defined	0.00
05A10986	15501021	Nile perch, coated, takeaway outlet, deep fried	0.00
05A10988	15101064	Orange roughy, raw	0.00
05A10989	15101065	Orange roughy, boiled, microwaved, steamed or poached, no added fat	0.00

05A10653	15101066	Salmon, Atlantic, raw	0.00
05A10733	15101067	Salmon, Pacific king, raw	0.00
05A10748	15101068	Salmon, raw, not further defined	0.00
05A10772	15101069	Salmon, baked, roasted, fried, grilled or BBQ'd, no added fat	0.00
05A10825	15101070	Salmon, baked, roasted, fried, grilled or BBQ'd, butter, dairy blend or margarine	0.00
05A10773	15101071	Salmon, baked, roasted, grilled, BBQ'd, fried or deep fried, canola oil	0.00
05A10777	15101072	Salmon, baked, roasted, grilled, BBQ'd, fried or deep fried, olive oil	0.00
05A10834	15101073	Salmon, baked, roasted, grilled, BBQ'd, fried or deep fried, other oil	0.00
05A10835	15101074	Salmon, baked, roasted, grilled, BBQ'd, fried or deep fried, fat not further defined	0.00
05A10782	15101075	Salmon, boiled, microwaved, steamed or poached, with or without added fat	0.00
05A10880	15501022	Salmon, coated, baked, roasted, fried, grilled or BBQ'd, no added fat	0.00
05A10879	15501023	Salmon, coated, baked, roasted, fried, grilled or BBQ'd, fat not further defined	0.00
05A10707	15101076	Salmon, sashimi style, raw	0.00
05A10927	15101077	Sardine, raw	0.00
05A10982	15101078	Sardine, cooked, not further defined	0.00
05A10655	15101079	Shark (flake), raw	0.00
05A10808	15101080	Shark (flake), baked, roasted, fried, grilled or BBQ'd, no added fat	0.00
05A10813	15101081	Shark (flake), baked, roasted, grilled, BBQ'd, fried or deep fried, fat not further defined	0.00
05A10654	15101082	Shark (flake), boiled, microwaved, steamed or poached, with or without added fat	0.00
05A10705	15501026	Shark (flake), coated, takeaway outlet, deep fried	0.00
05A10864	15501024	Shark (flake), coated, baked, roasted, grilled or BBQ'd, fat not further defined	0.00
05A10862	15501025	Shark (flake), coated, packaged frozen, baked, roasted, fried, grilled or BBQ'd, with or without added fat	0.00
05A10718	15101083	Silver perch, raw	0.00
05A10914	15101084	Silver perch, baked, roasted, fried, grilled or BBQ'd, no added fat	0.00
05A10913	15101085	Silver perch, baked, roasted, grilled, BBQ'd, fried or deep fried, fat not further defined	0.00
05A10730	15101086	Silver perch, boiled, microwaved, steamed or poached, with or without added fat	0.00
05A10657	15101087	Snapper, raw	0.00
05A10809	15501027	Snapper, baked, roasted, fried, grilled or BBQ'd, no added fat	0.00
05A10821	15101088	Snapper, baked, roasted, fried, grilled or BBQ'd, butter, dairy blend or margarine	0.00

05A10817	15101089	Snapper, baked, roasted, fried, grilled or BBQ'd, canola oil	0.00
05A10722	15101090	Snapper, baked, roasted, grilled, BBQ'd, fried or deep fried, olive oil	0.00
05A10767	15101091	Snapper, baked, roasted, grilled, BBQ'd, fried or deep fried, other oil	0.00
05A10836	15101092	Snapper, baked, roasted, grilled, BBQ'd, fried or deep fried, fat not further defined	0.00
05A10656	15101093	Snapper, boiled, microwaved, steamed or poached, with or without added fat	0.00
05A10911	15501028	Snapper, coated, baked, roasted, fried, deep fried, grilled or BBQ'd, fat not further defined	0.00
05A10807	15501029	Snapper, coated, takeaway outlet, deep fried	0.00
05A10990	15701001	Stingray, wild caught, flesh, baked, roasted, fried, grilled or BBQ'd, no added fat	0.00
05A10840	15101094	Swordfish, raw	0.00
05A10841	15101095	Swordfish, baked, roasted, fried, grilled or BBQ'd, no added fat	0.00
05A10842	15101096	Swordfish, baked, roasted, grilled, BBQ'd, fried or deep fried, fat not further defined	0.00
05A10716	15101097	Tilapia, raw	0.00
05A10727	15101098	Tilapia, boiled, microwaved, steamed or poached, no added fat	0.00
05A10856	15101099	Trevally or kingfish, raw	0.00
05A10981	15101100	Trevally or kingfish, cooked, no fat added	0.00
05A10859	15101101	Trevally or kingfish, baked, roasted, grilled, BBQ'd, fried or deep fried, fat not further defined	0.00
05A10858	15501030	Trevally or kingfish, coated, baked, roasted, fried, deep fried, grilled or BBQ'd, fat not further defined	0.00
05A10991	15101122	Trout, coral, cooked, with or without added fat	0.00
05A10658	15101102	Trout, rainbow, raw	0.00
05A10919	15101103	Trout, rainbow, baked, roasted, fried, grilled or BBQ'd, no added fat	0.00
05A10921	15101104	Trout, rainbow, baked, roasted, grilled, BBQ'd, fried or deep fried, fat not further defined	0.00
05A10922	15501031	Trout, rainbow, coated, baked, roasted, fried, deep fried, grilled or BBQ'd, fat not further defined	0.00
05A10723	15101105	Trout, rainbow, boiled, microwaved, steamed or poached, with or without added fat	0.00
05A10708	15101106	Tuna, raw	0.00
05A10802	15101107	Tuna, baked, roasted, fried, grilled or BBQ'd, no added fat	0.00
05A10830	15101108	Tuna, baked, roasted, grilled, BBQ'd, fried or deep fried, fat not further defined	0.00
05A10801	15101109	Tuna, boiled, microwaved, steamed or poached, with or without added fat	0.00
05A10706	15101110	Tuna, sashimi style, raw	0.00
15A20132	34101005	Turtle, wild caught, flesh, raw	0.00
15A20138	34101006	Turtle, wild caught, flesh, cooked	0.00

05A10967	15101112	Whitebait, floured, fried, not further defined	0.00
05A10660	15101113	Whiting, raw	0.00
05A10769	15101114	Whiting, baked, roasted, fried, grilled or BBQ'd, no added fat	0.00
05A10787	15101115	Whiting, baked, roasted, grilled, BBQ'd, fried or deep fried, fat not further defined	0.00
05A10659	15101116	Whiting, boiled, microwaved, steamed or poached, with or without added fat	0.00
05A10901	15501034	Whiting, coated, takeaway outlet, deep fried	0.00
05A10838	15501032	Whiting, coated, baked, roasted, fried, deep fried, grilled, or BBQ'd, fat not further defined	0.00
05A10745	15501033	Whiting, coated, packaged frozen, baked, roasted, fried, grilled, or BBQ'd, with or without added fat	0.00
05A10757	15302001	Fish, eel, raw	0.00
05A10758	15302002	Fish, eel, baked, roasted, fried, stir-fried, grilled or BBQ'd, no added fat	0.00
05A10759	15302003	Fish, eel, baked, roasted, fried, stir-fried, deep fried, grilled or BBQ'd, fat not further defined	0.00
05A10760	15101117	Fish, sashimi, raw, not further defined	0.00
05A10738	15101118	Fish, white flesh, raw, not further defined	0.00
05A10923	15101119	Fish, white flesh, baked, roasted, fried, grilled or BBQ'd, no added fat	0.00
05A10831	15101120	Fish, white flesh, baked, roasted, grilled, BBQ'd, fried or deep fried, fat not further defined	0.00
05A10832	15101121	Fish, white flesh, boiled, microwaved, steamed or poached, with or without added fat	0.00
05D10355	15501036	Fish, white flesh, battered, packaged frozen, baked, roasted, fried, grilled or BBQ'd, with or without added fat	0.00
05A10735	15501037	Fish, white flesh, battered, takeaway outlet, deep fried	0.00
05A10776	15501035	Fish, white flesh, coated, baked, roasted, fried, deep fried, grilled or BBQ'd, fat not further defined	0.00
05D10356	15501038	Fish, white flesh, crumbed, packaged frozen, baked, roasted, fried, grilled or BBQ'd, with or without added fat	0.00
05A10737	15501039	Fish, white flesh, crumbed, takeaway outlet, deep fried	0.00
05C10143	15201001	Crab, flesh, purchased steamed, poached or boiled	0.00
05C10192	15201002	Crab, flesh, cooked, fat not further defined	0.00
05C10194	15502001	Crab, flesh, coated, fried or deep fried, fat not further defined	0.00
05C10224	15503001	Jellyfish, coated, baked, roasted, fried, grilled or BBQ'd, fat not further defined	0.00
05C10144	15201003	Lobster or crayfish, flesh, purchased steamed, poached or boiled, no added fat	0.00
05C10225	15502005	Lobster or crayfish, flesh, coated, fried or deep-fried, fat not further defined	0.00
05C10166	15201004	Moreton bay bug or balmain bug, flesh, raw	0.00
05C10222	15201005	Moreton bay bug, cooked, with or without fat	0.00

05C10161	15202001	Mussel, blue, boiled, microwaved, steamed or poached, no added fat	0.00
05C10149	15202002	Mussel, green, boiled, microwaved, steamed or poached, no added fat	0.00
05C10165	15202003	Mussel, cooked, with or without added fat	0.00
05C10197	15202004	Octopus, raw	0.00
05C10218	15202005	Octopus, cooked, with or without added fat	0.00
05C10198	15202006	Octopus, marinated, baked, grilled, fried or BBQ'd, fat not further defined	0.00
05C10200	15503002	Octopus, coated, takeaway outlet, fried, fat not further defined	0.00
05C10150	15202007	Oyster, raw	0.00
05C10220	15202008	Oyster, cooked, with or without added fat	0.00
05C10226	15702002	Pipi, wild harvested, cooked	0.00
05C10145	15201006	Prawn, king or medium, raw (green)	0.00
05C10181	15201007	Prawn, king or medium, flesh, baked, roasted, fried, grilled or BBQ'd, no added fat	0.00
05C10184	15201008	Prawn, king or medium, flesh, baked, roasted, fried, grilled or BBQ'd, fat not further defined	0.00
05C10147	15201009	Prawn, king or medium, flesh, boiled, microwaved, steamed or poached, no added fat	0.00
05C10183	15201010	Prawn, king or medium, flesh, boiled, microwaved, steamed or poached, fat not further defined	0.00
05C10146	15201011	Prawn, school, flesh, boiled, microwaved, steamed or poached, no added fat	0.00
05C10221	15502003	Prawn, coated, packaged frozen, baked, roasted, fried, grilled or BBQ'd, fat not further defined	0.00
05C10178	15502002	Prawn, coated, fried or deep fried, fat not further defined	0.00
05D20118	15502004	Prawn, coated, takeaway outlet, deep fried	0.00
05C10152	15202009	Scallop, with roe, raw	0.00
05C10168	15202010	Scallop with or without roe, baked, roasted, grilled, fried or BBQ'd, no added fat	0.00
05C10187	15202011	Scallop with or without roe, baked, roasted, grilled, fried, deep fried or BBQ'd, fat not further defined	0.00
05C10151	15202012	Scallop with or without roe, boiled, microwaved, steamed or poached, with or without added fat	0.00
05C10188	15503003	Scallop with or without roe, coated, baked, roasted, grilled or BBQ'd, fat not further defined	0.00
05C10190	15503004	Scallop with or without roe, coated, takeaway outlet, deep fried	0.00
05C10148	15202013	Squid or calamari, raw	0.00
05C10185	15202014	Squid or calamari, baked, roasted, fried, stir-fried, grilled or BBQ'd, no added fat	0.00
05C10164	15202015	Squid or calamari, baked, roasted, fried, stir-fried, deep fried, grilled or BBQ'd, fat not further defined	0.00
05C10158	15202016	Squid or calamari, boiled, microwaved, steamed or poached, with or without added fat	0.00

05C10172	15503005	Squid or calamari, battered, takeaway outlet, deep fried	0.00
05C10157	15503006	Squid or calamari, crumbed, takeaway outlet, deep fried	0.00
05C10169	15503007	Squid or calamari, coated, baked, roasted, fried, grilled or BBQ'd, fat not further defined	0.00
05C10176	15503008	Squid or calamari, coated, packaged frozen, baked, roasted, fried, grilled or BBQ'd, with or without added fat	0.00
05C10228	15201012	Yabby (yabbie), flesh, cooked, no added fat	0.00
05D20130	15303001	Mixed seafood, contains fish & shellfish, raw	0.00
05D20147	15303002	Mixed seafood, contains fish & shellfish, baked, roasted, fried, grilled or BBQ'd, with or without added fat	0.00
05D20129	15303003	Mixed seafood, contains fish & shellfish, boiled, microwaved, steamed or poached, with or without added fat	0.00
05D20148	15303004	Mixed seafood, contains fish & shellfish, coated, baked, roasted, grilled, BBQ'd, fried or deep fried, fat not further defined	0.00
05D10420	15504009	Mixed seafood, tempura coating, fried or deep fried, fat not further defined	0.00
05D20149	15303005	Mixed seafood, contains fish & shellfish, cooked, not further defined	0.00
05C10227	15702001	Mixed seafood (except finfish), wild-harvested, raw	0.00
05D20151	15504010	Mixed seafood, for use in garden salad recipes	0.00
05A10666	15401001	Anchovy, canned	0.00
05A10661	15504001	Cod, Atlantic, dried, salted	0.00
05A10662	15102001	Cod, smoked, raw	0.00
05A10663	15102002	Cod or hake, smoked, boiled, microwaved, steamed or poached, no added fat	0.00
05C10155	15402001	Crabmeat, canned in brine, undrained	0.00
05D10372	15504002	Fish ball, Asian style, purchased cooked	0.00
05D10390	15504003	Fish ball, packaged frozen, boiled, steamed, poached, microwaved, no fat added	0.00
05A10935	15504004	Fish patty or cake, fried, fat not further defined	0.00
05A10933	15504005	Fish patty or cake, crumbed, fried, fat not further defined	0.00
05D10357	15504006	Fish patty or cake, packaged frozen, baked, roasted, fried, grilled or BBQ'd, fat not further defined	0.00
05D10358	15504007	Fish patty or cake, takeaway outlet, deep fried	0.00
05D10366	15501041	Fish finger, crumbed, packaged frozen, raw	0.00
05D10364	15501042	Fish finger, crumbed, packaged frozen, baked, roasted, fried or grilled, no added fat	0.00
05D10401	15501043	Fish finger, crumbed, packaged frozen, baked, roasted, fried, grilled or BBQ'd, fat not further defined	0.00
05D10404	15501040	Fish bite or cocktail, crumbed, takeaway outlet, deep fried	0.00

05A10798	15504012	Salmon patty or cake, crumbed, fried, fat not further defined	0.00
05D10360	15504008	Fish paste or spread	0.00
05D10352	15301001	Fish roe (caviar), black	0.00
05D10353	15301002	Fish roe (caviar), red	0.00
05A10665	15401002	Herring, Atlantic, pickled	0.00
05C10153	15402002	Mussel, smoked, canned in oil, drained	0.00
05C10154	15402003	Oyster, smoked, canned in oil, drained	0.00
05A10671	15401004	Salmon, pink, unflavoured, canned in brine, drained	0.00
05A10673	15401005	Salmon, pink, unflavoured, canned in water, drained	0.00
05A10669	15401006	Salmon, red, unflavoured, canned in brine, drained	0.00
05A10672	15401007	Salmon, red, unflavoured, canned in water, drained	0.00
05A10957	15401008	Salmon, unflavoured, canned in brine, drained	0.00
05A10979	15401009	Salmon, unflavoured, canned in oil	0.00
05A10775	15401010	Salmon, unflavoured, canned in water, drained	0.00
05A10795	15401011	Salmon, unflavoured, canned, drained	0.00
05A10796	15401012	Salmon, flavoured, canned, drained	0.00
05A10797	15401013	Salmon, canned, drained, not further defined	0.00
05A10664	15102003	Salmon, smoked, sliced	0.00
05A10674	15401014	Sardine, canned in oil, undrained	0.00
05A10675	15401015	Sardine, canned in oil, drained	0.00
05A10710	15401016	Sardine, canned in tomato sauce, undrained	0.00
05A10676	15401017	Sardine, canned in water, drained	0.00
05D10362	15504013	Seafood or fish stick (surimi), packaged frozen, raw	0.00
05D10361	15504014	Seafood or fish stick (surimi), packaged frozen, fried, peanut oil	0.00
05D10408	15504015	Seafood or fish stick (surimi), packaged frozen, boiled, microwaved, steamed or poached, no added fat	0.00
05D10409	15504016	Seafood or fish stick (surimi), coated, takeaway outlet, deep fried	0.00
05A10709	15102004	Smoked fish (including eel & trout), smoked	0.00
05A10678	15401018	Tuna, unflavoured, canned in brine, drained	0.00
05A10680	15401019	Tuna, unflavoured, canned in vegetable oil, drained	0.00
05A10681	15401020	Tuna, unflavoured, canned in water, drained	0.00
05A10714	15401021	Tuna, flavoured, canned, drained	0.00
05A10970	15401022	Tuna, canned, not further defined	0.00
12C10415	28302001	Bar, muesli or snack, plain or with 10% dried fruit	0.00
12C10496	28302002	Bar, muesli or snack, plain or with 10% dried fruit, added vitamins B1, B2, B3, folate & Fe	0.00
12C10418	28303001	Bar, muesli or snack, plain or with 10% dried fruit, chocolate-coated	0.00
12C10542	28302003	Bar, muesli or snack, plain or with 10% dried fruit, high fibre, added vitamins B1, B2, B3, B6, E, & folate, Fe & Zn	0.00
12C10416	28303002	Bar, muesli or snack, plain or with 10% dried fruit, yoghurt-coated	0.00
12C10501	28303003	Bar, muesli or snack, plain or with 10% dried fruit, yoghurt-coated, added vitamins B1, B2, B3, folate & Fe	0.00

12C10494	28302004	Bar, muesli or snack, with 10% dried fruit & 5% nuts	0.00
12C10495	28302005	Bar, muesli or snack, with 10% dried fruit & 10% nuts	0.00
12C10519	28302006	Bar, muesli or snack, with 10% dried fruit & 45% nuts, chocolate-coated	0.00
12C10520	28302007	Bar, muesli or snack, with 10% dried fruit & 60% nuts	0.00
12C10537	28302008	Bar, muesli or snack, with 10% dried fruit & 60% nuts, yoghurt-coated	0.00
12C10480	28303004	Bar, muesli or snack, with 15% dried fruit & 15% nuts, yoghurt-coated	0.00
12C10543	28302009	Bar, muesli or snack, with 15% dried fruit & 25% nuts, added vitamins B1, B2, B3, C & folate, Fe, & Zn	0.00
12C10498	28302011	Bar, muesli or snack, with 20% dried fruit & 20% nuts, chocolate base	0.00
12C10499	28302010	Bar, muesli or snack, with 20% dried fruit & 5% nuts	0.00
12C10497	28302012	Bar, muesli or snack, with 30% dried fruit & 30% nuts	0.00
12C10538	28303005	Bar, muesli or snack, with 30% dried fruit, yoghurt-coated, added vitamins B1, B2, B3, folate & Fe	0.00
12C10420	28302013	Bar, muesli or snack, with 10% nuts	0.00
12C10482	28302014	Bar, muesli or snack, with 10% nuts, added flaxseeds	0.00
12C10544	28303006	Bar, muesli or snack, with 10% nuts, chocolate-coated	0.00
12C10531	28302015	Bar, muesli or snack, with 70% nuts	0.00
12C10515	28302016	Bar, muesli or snack, with 70% nuts, added vitamins B1, B2, B3, C & folate, Fe, & Zn	0.00
12C10514	28202002	Bar, muesli or snack, with caramel & sesame seeds	0.00
12C10414	28303007	Bar, muesli or snack, with chocolate chips or coating	0.00
12C10443	28304001	Bar, muesli or snack, fruit filled, baked	0.00
12C10506	28304002	Bar, muesli or snack, fruit filled, high fibre, baked	0.00
12C10500	28302017	Bar, muesli or snack, gluten free, with 20% dried fruit & 20% seeds	0.00
12C10464	28301001	Bar, muesli or snack, made from breakfast cereal	0.00
12C10463	28302018	Bar, muesli or snack, made from breakfast cereal with dried fruit	0.00
12C10439	28301002	Bar, muesli or snack, made from breakfast cereal with milk solids	0.00
12C10441	28302019	Bar, muesli or snack, made from breakfast cereal with nuts	0.00
12C10541	28302020	Bar, muesli or snack, made from breakfast cereal, with chocolate & nuts, added vitamins B1, B2, B3, C & folate, Fe & Zn	0.00
12C10529	28301003	Bar, muesli or snack, made from breakfast cereal, with chocolate coating, added vitamins B1, B2, B3, B6 & folate, Ca, Fe & Zn	0.00
12C10516	28301004	Bar, muesli or snack, made from puffed rice, added vitamins B1, B2, B3, C & folate, Fe, & Zn	0.00
12C10554	28301005	Bar, muesli or snack, made from puffed rice, with chocolate flavour, chips or coating	0.00
12C10517	28303008	Bar, muesli or snack, made from puffed rice, with chocolate flavour, chips or coating, added vitamins B1, B2, B3, C & folate, Fe, & Zn	0.00

12C10518	28303009	Bar, muesli or snack, made from puffed rice, with yoghurt flavour coating, added vitamins B1, B2, B3, C & folate, Fe, & Zn	0.00
12C10564	28305001	Bar, snack style, chocolate flavour, space stick	0.00
12C10455	28301006	Bar, snack style, chocolate fortified cereal, milk solids	0.00
12C10559	28302021	Bar, muesli or snack, not further defined	0.00
01B10545	30105001	Amino acid or creatine powder	0.00
01B10546	30104001	Amino acid or creatine drink, prepared from dry powder with water or milk	0.00
02C20379	28305002	Bar, high protein, chocolate flavour, reduced carbohydrate, commercial	0.00
02C20381	28305003	Bar, high protein, reduced carbohydrate, fortified, caffeinated, commercial	0.00
12C10481	30101001	Bar, meal replacement, fortified	0.00
12C10539	28302022	Bar, nutrition, energy or snack	0.00
02C20378	28305004	Bar or biscuit, high protein, chocolate flavour, commercial	0.00
01B20487	30106001	Beverage, medical or special purpose, added vitamins & minerals	0.00
01B40007	30102019	Beverage, water based, thickened, with caffeine	0.00
01B40006	30104002	Gel, energy, caffeinated, all flavours	0.00
01B10449	30103001	Meal replacement powder, coffee flavour	0.00
01B10505	30103002	Meal replacement powder, coffee flavour, added fibre	0.00
01B10495	30102001	Meal replacement drink, coffee flavour, prepared with milk, any type	0.00
01B10463	30102002	Meal replacement drink, coffee flavour, prepared with water	0.00
01B10499	30102003	Meal replacement drink, coffee flavour (Optislim), prepared with water	0.00
01B10507	30102004	Meal replacement drink, coffee flavour, added fibre, prepared with milk, any type	0.00
01B10506	30102005	Meal replacement drink, coffee flavour, added fibre, prepared with water	0.00
01B10502	30103003	Meal replacement powder, non-coffee flavour	0.00
01B10450	30103004	Meal replacement powder, non-coffee flavours, added fibre	0.00
01B10498	30103005	Meal replacement powder, non-coffee flavour, fortified (Optislim)	0.00
01B10504	30102006	Meal replacement drink, non-coffee flavour, prepared with milk, any type	0.00
01B10503	30102007	Meal replacement drink, non-coffee flavour, prepared with water	0.00
01B10500	30102008	Meal replacement drink, non-coffee flavour (Optislim), prepared with water	0.00
01B10497	30102009	Meal replacement drink, non-coffee flavours, added fibre, prepared with milk, any type	0.00
01B10496	30102010	Meal replacement drink, non-coffee flavours, added fibre, prepared with water	0.00
01B10440	30107001	Oral supplement powder, chocolate flavour, fortified (Sustagen Hospital)	0.00

01B10519	30107002	Oral supplement powder, chocolate flavour, fortified (Sustagen Everyday)	0.00
01B10459	30106002	Oral supplement beverage, chocolate flavour, fortified (Sustagen Hospital), prepared with regular fat milk	0.00
01B10458	30106003	Oral supplement beverage, chocolate flavour, fortified (Sustagen Hospital), prepared with water	0.00
01B10529	30106004	Oral supplement beverage, chocolate flavour, fortified (Sustagen Everyday), prepared with cow's milk	0.00
01B10439	30107003	Oral supplement powder, vanilla flavour, fortified (Sustagen Hospital)	0.00
01B10441	30107004	Oral supplement powder, vanilla flavour, children's formulation, fortified (Sustagen Kid Essentials)	0.00
01B10457	30106005	Oral supplement beverage, vanilla flavour, fortified (Sustagen Hospital), prepared with regular fat milk	0.00
01B10460	30106006	Oral supplement beverage, vanilla flavour, fortified (Sustagen Kid Essentials), prepared with regular fat milk	0.00
01B10456	30106007	Oral supplement beverage, vanilla flavour, fortified (Sustagen Hospital), prepared with water	0.00
01B10548	30107005	Oral supplement powder, Ensure	0.00
01B10549	30106008	Oral supplement beverage, Ensure, prepared with water or milk	0.00
01B10448	30105002	Protein powder, whey based, protein > 70%, reduced carbohydrate, fortified, caffeinated	0.00
01B10509	30104003	Protein drink, whey based, protein >70%, reduced carbohydrate, fortified, prepared with water	0.00
01B10510	30105003	Protein powder, protein >70%, caffeinated	0.00
01B10533	30104004	Protein drink, protein >70%, caffeinated, prepared with water	0.00
01B10511	30105004	Protein powder, protein 45%, unfortified	0.00
01B10526	30104005	Protein drink, protein 45%, unfortified, prepared with milk, any type	0.00
01B10512	30104006	Protein drink, protein 45%, unfortified, prepared with water	0.00
01B10513	30105005	Protein powder, protein 45%, fortified	0.00
01B10530	30104007	Protein drink, protein 45%, fortified, prepared with milk, any type	0.00
01B10514	30104008	Protein drink, protein 45%, fortified, prepared with water	0.00
01B10516	30105006	Protein powder, protein 45%, reduced sugars, fortified	0.00
01B10536	30104009	Protein drink, protein 45%, reduced sugars, fortified, prepared with water	0.00
01B10515	30105007	Protein powder, protein 45%, fortified, caffeinated	0.00
01B10537	30104010	Protein drink, protein 45%, fortified, caffeinated, prepared with milk, any type	0.00
01B10528	30104011	Protein drink, protein 45%, fortified, caffeinated, prepared with water	0.00
01B10517	30105008	Protein powder, protein 30%, fortified	0.00

01B10534	30104012	Protein drink, protein 30%, fortified, prepared with milk, any type	0.00
01B10538	30104013	Protein drink, protein 30%, fortified, prepared with water	0.00
01B10552	30105009	Protein powder, protein 25%, unfortified	0.00
01B10565	30105010	Protein powder, protein 20%, fortified, caffeinated	0.00
01B10567	30104024	Protein drink, protein 20%, fortified, caffeinated, prepared with water	0.00
01B20516	30107006	Powder, medical or special purpose, added vitamins & minerals	0.00
01B10446	30105011	Protein powder, soy based, protein > 70%, unfortified	0.00
01B10462	30104014	Protein drink, soy based, protein >70%, unfortified, prepared from dry powder with milk, any type	0.00
01B10436	30105012	Protein powder, whey based, protein > 70%, unfortified	0.00
01B10452	30104015	Protein drink, whey based, protein >70%, unfortified, prepared from dry powder with milk, any type	0.00
01B10461	30104016	Protein drink, whey based, protein >70%, unfortified, prepared from dry powder with soy milk	0.00
01B10451	30104017	Protein drink, whey based, protein >70%, unfortified, prepared from dry powder with water	0.00
01B10447	30105013	Protein powder, whey based, protein > 70%, fortified	0.00
01B10531	30104018	Protein drink, whey based, protein >70%, fortified, prepared with milk, any type	0.00
01B10527	30104019	Protein drink, whey based, protein >70%, fortified, prepared with juice	0.00
01B10508	30104020	Protein drink, whey based, protein >70%, fortified, prepared with water	0.00
01B10541	30105014	Protein or meal replacement powder, not further defined, dry powder only	0.00
01B10543	30104021	Protein or meal replacement powder, not further defined, prepared with milk, any type	0.00
01B10542	30104022	Protein or meal replacement powder, not further defined, prepared with water	0.00
09A10227	30104023	Sports supplement, cows milk, flavoured, chocolate, reduced fat, added protein	0.00
13A12631	24803001	Vegetable and/or fruit blend, dry powder	0.00
13A12644	11308002	Vegetable &/or fruit blend, prepared from dry powder with water	0.00
01B10464	30103006	Very low energy diet powder, chicken or vegetable soup, fortified (Optifast)	0.00
01B10465	30102011	Very low energy diet soup, chicken or vegetable fortified (Optifast), prepared with water	0.00
01B10437	30103007	Very low energy diet powder, chocolate flavour, fortified (Optifast)	0.00
01B10539	30102012	Very low energy diet drink, chocolate flavour, fortified (Optifast), prepared with milk, any type	0.00
01B10453	30102013	Very low energy diet drink, chocolate flavour, fortified (Optifast), prepared with water	0.00

01B10444	30103008	Very low energy diet powder, coffee flavour, fortified (Optifast)	0.00
01B10445	30103009	Very low energy diet powder, coffee flavour, fortified (Optislim)	0.00
01B10550	30102014	Very low energy diet drink, coffee flavour, fortified (Optifast), prepared with water or milk, any type	0.00
01B10535	30102015	Very low energy diet drink, coffee flavour, fortified (Opitfast), prepared with water	0.00
01B10438	30103010	Very low energy diet powder, vanilla flavour, fortified (Optifast)	0.00
01B10455	30102016	Very low energy diet drink, vanilla flavour, fortified (Optifast), prepared with milk, any type	0.00
01B10454	30102017	Very low energy diet drink, vanilla flavour, fortified (Optifast), prepared with water	0.00
01B10547	30103011	Very low energy diet powder, strawberry flavour, fortified (Optifast)	0.00
01B10551	30102018	Very low energy diet drink, strawberry flavour, fortified (Optifast), prepared with water or milk, any type	0.00
10D10170	26102001	Beetroot crisps or chips, plain, salted	0.00
10D10134	26301001	Chip or crisp, soy	0.00
10D10137	26201001	Corn chips, cheese flavoured, salted	0.00
10D10160	26201002	Corn chips, other flavours, salted	0.00
10D10138	26201003	Corn chips, plain, toasted, no added salt	0.00
10D10136	26201004	Corn chips, plain toasted, salted	0.00
10D10165	26201005	Corn chips, puffed, plain, salted	0.00
10D10172	26201006	Corn chips, not further defined	0.00
10D10140	26301002	Extruded snack, cheese flavoured	0.00
10D10141	26301003	Extruded snack, non-cheese flavoured	0.00
12C10438	28201002	Fruit, leather	0.00
10D10149	26301004	Grain waves wheat snack, plain, original	0.00
10D10159	26301005	Grain waves wheat snack, cheese or sour cream & chives	0.00
10D10150	26301006	Grain waves wheat snack, other flavours	0.00
02A10395	26401003	Noodle snack, wheat, flavoured, dry	0.00
13B20259	26401004	Pappadam, raw	0.00
13B20232	26401005	Pappadam, deep fried	0.00
13B20260	26401006	Pappadam, microwaved without oil or salt	0.00
10D10139	26202001	Popcorn, air-popped, no added fat or salt	0.00
10D10135	26202002	Popcorn, commercial, butter flavoured, salted	0.00
10D10168	26202006	Popcorn, commercial, sugar coating	0.00
10D10151	26202003	Popcorn, microwaved, butter flavoured, salted	0.00
10D10169	26202004	Popcorn, regular, unflavoured, salted, vegetable oil	0.00
10D10166	26202005	Popcorn, regular, unflavoured, unsalted, vegetable oil	0.00
10D10132	26401007	Pork rind snack	0.00
10D10152	26101001	Potato crisps or chips, cheese based flavours	0.00
10D10142	26101003	Potato crisps or chips, plain, salted	0.00
10D10146	26101004	Potato crisps or chips, plain, unsalted	0.00

10D10147	26101002	Potato crisps or chips, flavoured, reduced fat	0.00
10D10145	26101005	Potato crisps or chips, salt & vinegar flavoured	0.00
10D10153	26101006	Potato crisps or chips, sour cream based flavours	0.00
10D10154	26101007	Potato crisps or chips, other flavours	0.00
10D10167	26101015	Potato straws, French fries, salt & vinegar flavoured	0.00
10D10161	26101008	Potato crisps or chips, reformed, cheese flavour, salted	0.00
10D10155	26101009	Potato crisps or chips, reformed, plain, salted	0.00
10D10156	26101010	Potato crisps or chips, reformed, salt & vinegar flavoured	0.00
10D10157	26101011	Potato crisps or chips, reformed, sour cream & chives flavour, salted	0.00
10D10158	26101012	Potato crisps or chips, reformed, other flavours, salted	0.00
10D10163	26101013	Potato crisps or chips, not further defined	0.00
10D10130	26101014	Potato straws, French fries, plain	0.00
10D10133	26401008	Prawn cracker, Asian, commercial, fried	0.00
10D10131	26401009	Pretzels, salted	0.00
02C10158	26301007	Rice crisps or chips, extruded, flavoured	0.00
10D10177	26401010	Snack mix, Bhuja	0.00
02F40306	26201007	Taco shell, from corn flour, plain	0.00
10D10171	26102002	Vegetable crisps or chips, plain, salted	0.00
10C10572	21102001	Soup, broth, Asian style, with egg & rice	0.00
10C10499	21201001	Soup, Asian style meat & vegetable, with noodles, cup of soup, instant dry mix	0.00
10C10498	21201002	Soup, Asian style seafood & vegetable, with noodles, cup of soup, instant dry mix	0.00
10C10519	21301001	Soup, Asian style seafood & vegetable, with noodles, cup of soup, prepared from instant dry mix with water	0.00
10C10525	21601001	Soup, beef & vegetable, from cafe or restaurant	0.00
10C10532	21101001	Soup, beef & vegetable, prepared with stock or water, homemade from basic ingredients	0.00
10C10535	21101002	Soup, beef & vegetable, with pasta or grains, prepared with stock or water, homemade from basic ingredients	0.00
10C10534	21101003	Soup, beef, vegetable & legume, prepared with stock or water, homemade from basic ingredients	0.00
10C10536	21101004	Soup, beef, vegetable & legume, with pasta or grains, prepared with stock or water, homemade	0.00
10C10517	21101005	Soup, broth with meat & noodles	0.00
10C10482	21201003	Soup, broth style with meat, cup of soup, instant dry mix	0.00
10C10471	21301002	Soup, broth style with meat, prepared from instant dry mix with water	0.00
10C10466	21201004	Soup, chicken & noodle, cup of soup, instant dry mix	0.00
10C10467	21301003	Soup, chicken & noodle, cup of soup, prepared from instant dry mix with water	0.00
10C10550	21501001	Soup, chicken & noodle, ready to eat, canned	0.00
10C10555	21101006	Soup, chicken & sweetcorn, prepared with stock or water, homemade from basic ingredients	0.00
10C10510	21501002	Soup, chicken & sweetcorn, ready to eat, canned	0.00

10C10479	21201005	Soup, chicken, with or without vegetables, cup of soup, instant dry mix	0.00
10C10523	21301004	Soup, chicken, with or without vegetables, cup of soup, prepared from instant dry mix with water	0.00
10C10544	21201006	Soup, chicken, with or without vegetables, cup of soup, instant dry mix, reduced salt	0.00
10C10545	21301005	Soup, chicken, with or without vegetables, cup of soup, reduced salt, prepared from instant dry mix with water	0.00
10C10480	21201007	Soup, chicken & vegetable, with pasta or croutons, cup of soup, instant dry mix	0.00
10C10548	21501003	Soup, chicken & vegetable, ready to eat, canned	0.00
10C10526	21601002	Soup, chicken & vegetable, from cafe or restaurant	0.00
10C10537	21101007	Soup, chicken & vegetable, prepared with stock or water, homemade from basic ingredients	0.00
10C10538	21101008	Soup, chicken & vegetable, with grains or pasta, prepared with stock or water, homemade from basic ingredients	0.00
10C10539	21101009	Soup, chicken, vegetable & legume, prepared with stock or water, homemade from basic ingredients	0.00
10C10540	21101010	Soup, chicken, vegetable & legume, with grains or pasta, prepared with stock or water, homemade	0.00
10C10453	21401001	Soup, cream of chicken, condensed, canned	0.00
10C10458	21501004	Soup, cream of chicken, condensed, canned, prepared with regular fat cows milk & water	0.00
10C10455	21501005	Soup, cream of seafood, condensed, canned, prepared with regular fat cows milk & water	0.00
10C10454	21402001	Soup, cream of vegetables, condensed, canned	0.00
10C10459	21502001	Soup, cream of vegetables, condensed, canned, prepared with regular fat cows milk & water	0.00
10C10553	21502002	Soup, cream of vegetable, ready to eat, canned	0.00
10C10567	21102002	Soup, cream of vegetable, prepared with cream or milk, homemade from basic ingredients	0.00
10C10464	21202001	Soup, cream variety, instant dry mix	0.00
10C10456	21302001	Soup, cream variety, prepared from dry mix with water	0.00
10C10460	21302002	Soup, cup of soup style with croutons, prepared from instant dry mix with water	0.00
10C10568	21101011	Soup, fish, prepared with stock or water, homemade from basic ingredients	0.00
10C10486	21202002	Soup, French onion, instant dry mix	0.00
10C10514	21202003	Soup, French onion, instant dry mix, reduced salt	0.00
10C10513	21302003	Soup, French onion, prepared from instant dry mix with water	0.00
10C10569	21102003	Soup, French onion, homemade from basic ingredients	0.00
10C10542	21101012	Soup, ham & vegetable, prepared with stock or water, homemade from basic ingredients	0.00
10C10543	21101013	Soup, ham & vegetable, with pasta or grains, prepared with stock or water, homemade from basic ingredients	0.00

10C10571	21601003	Soup, hot & sour (tom yum), prepared with stock or water	0.00
10C10546	21601004	Soup, laksa, beef, chicken or pork, with noodles, prepared with coconut milk	0.00
10C10562	21601005	Soup, laksa, prawn or seafood, with noodles, prepared with coconut milk	0.00
10C10556	21101014	Soup, lamb & vegetable, prepared with stock or water, homemade from basic ingredients	0.00
10C10557	21101015	Soup, lamb & vegetable, with grains or pasta, prepared with stock or water, homemade from basic ingredients	0.00
10C10558	21101016	Soup, lamb, vegetable & legume, prepared with stock or water, homemade from basic ingredients	0.00
10C10527	21602001	Soup, lentil or other legumes, from cafe or restaurant	0.00
10C10564	21101017	Soup, offal, prepared with stock or water, homemade from basic ingredients	0.00
10C10472	21501006	Soup, meat & vegetable, canned, ready-to-eat, heated	0.00
10C10512	21102004	Soup, minestrone, prepared, from cafe or restaurant	0.00
10C10476	21102005	Soup, minestrone, prepared with stock or water, homemade from basic ingredients	0.00
10C10515	21102006	Soup, miso, with tofu & seaweed	0.00
10C10473	21302004	Soup, mixed vegetables, prepared from instant dry mix with water	0.00
10C10484	21202004	Soup, mixed vegetables, reduced energy, cup of soup, instant dry mix	0.00
10C10524	21602002	Soup, mixed vegetable, from cafe or restaurant	0.00
10C10529	21102007	Soup, mixed vegetable, prepared with stock or water, homemade from basic ingredients	0.00
10C10533	21102008	Soup, mixed vegetable & legume, with pasta or grains, prepared with stock or water, homemade from basic ingredients	0.00
10C10530	21102009	Soup, mixed vegetable & pasta or grains, prepared with stock or water, homemade from basic ingredients	0.00
10C10474	21102010	Soup, mixed vegetable & legume, prepared with stock or water, homemade from basic ingredients	0.00
10C10565	21101018	Soup, mussel, oyster or scallop, homemade from basic ingredients	0.00
10C10520	21201008	Soup, pea & ham, cup of soup, instant dry mix	0.00
10C10521	21301006	Soup, pea & ham, cup of soup, prepared from instant dry mix with water	0.00
10C10475	21501007	Soup, pea & ham, condensed, canned, prepared with water	0.00
10C10509	21501008	Soup, pea & ham, ready to eat, canned	0.00
10C10505	21101019	Soup, pea & ham, prepared with stock or water, homemade from basic ingredients	0.00
10C10560	21101020	Soup, pork & vegetable, prepared with stock or water, homemade from basic ingredients	0.00
10C10561	21101021	Soup, pork & vegetable, with grains or pasta, prepared with stock or water, homemade from basic ingredients	0.00

10C10563	21101022	Soup, pork, vegetable & legume, prepared with stock or water, homemade from basic ingredients	0.00
10C10507	21102011	Soup, potato, prepared with stock or water, homemade from basic ingredients	0.00
10C10506	21102012	Soup, potato, prepared with stock or water & cream, homemade from basic ingredients	0.00
10C10511	21102013	Soup, pumpkin, prepared, from cafe or restaurant	0.00
10C10522	21102014	Soup, pumpkin, prepared with stock or water, homemade from basic ingredients	0.00
10C10502	21102015	Soup, pumpkin, prepared with stock or water & coconut milk, homemade from basic ingredients	0.00
10C10491	21102016	Soup, pumpkin, prepared with stock or water & milk, homemade from basic ingredients	0.00
10C10508	21502003	Soup, pumpkin, ready to eat, canned	0.00
10C10570	21101023	Soup, seaweed, with or without meat, vegetables & grains or pasta	0.00
10C10566	21101024	Soup, shark's fin	0.00
10C10481	21202005	Soup, tomato, with or without noodles, cup of soup, instant dry mix	0.00
10C10495	21302005	Soup, tomato, with or without noodles, cup of soup, prepared from instant dry mix with water	0.00
10C10452	21402002	Soup, tomato, condensed, canned	0.00
10C10518	21502004	Soup, tomato, condensed, canned, prepared with water	0.00
10C10457	21502005	Soup, tomato, condensed, canned, prepared with cows milk & water	0.00
10C10551	21502006	Soup, tomato, ready to eat, canned	0.00
10C10528	21102017	Soup, tomato, prepared with stock or water, homemade from basic ingredients	0.00
10C10477	21202006	Soup, vegetable, cup of soup, instant dry mix	0.00
10C10478	21302006	Soup, vegetable, cup of soup, prepared from instant dry mix with water	0.00
10C10547	21502007	Soup, vegetable, ready to eat, canned	0.00
10C10516	21101025	Soup, wonton, clear broth with dumplings	0.00
12C10404	28104001	Bar, carob	0.00
12C10437	28102001	Bar, cherry & coconut centre, dark chocolate-coated	0.00
12C10491	28103001	Bar, chocolate nougat centre, milk chocolate-coated	0.00
12C10433	28102002	Bar, coconut cream centre, milk chocolate-coated	0.00
12C10431	28202003	Bar, fruit, nut & seed	0.00
12C10435	28103002	Bar, honeycomb centre, milk chocolate-coated	0.00
12C10442	13304001	Muffin bar, fruit flavoured	0.00
12C10434	28103003	Bar, nougat & caramel centre, milk chocolate-coated	0.00
12C10565	28103004	Bar, nougat & caramel centre, milk chocolate coated, battered, deep fried	0.00
12C10484	28102003	Bar, nougat, caramel & peanut centre, milk chocolate-coated	0.00
12C10549	28102004	Bar, nougat & nut centre, chocolate-coated	0.00
12C10487	28103005	Bar, Turkish delight centre, milk chocolate-coated	0.00

12C10488	28103006	Bar, wafer & cream layers, chocolate-coated	0.00
12C10556	28103007	Bar, wafer & fudge, milk chocolate-coated	0.00
12C10555	28102005	Bar, wafer & hazelnut, milk chocolate-coated	0.00
12C10490	28103008	Bar, wafer or biscuit & caramel, milk chocolate-coated	0.00
12C10528	28102006	Bar, wafer or biscuit, caramel & peanut, milk chocolate-coated	0.00
12C10413	28101001	Chocolate, compound, cooking	0.00
12C10406	28101002	Chocolate, dark, high cocoa solids, less than 60% cocoa solids	0.00
12C10503	28101003	Chocolate, dark, high cocoa solids, 60% cocoa solids or greater	0.00
12C10460	28103010	Chocolate, dark, fondant filled	0.00
12C10459	28103011	Chocolate, dark, peppermint filled	0.00
12C10533	28103012	Chocolate, dark, with dried fruit	0.00
12C10483	28102007	Chocolate, dark, with dried fruit & nuts	0.00
12C10466	28102008	Chocolate, dark, with nuts	0.00
12C10558	28103013	Chocolate, liqueur-filled	0.00
12C10407	28101004	Chocolate, milk	0.00
12C10462	28103014	Chocolate, milk, caramel filled	0.00
12C10461	28103015	Chocolate, milk, fondant filled	0.00
12C10471	28102009	Chocolate, milk, with coconut	0.00
12C10477	28102010	Chocolate, milk, with dried fruit & nuts	0.00
12C10512	28102011	Chocolate, milk, with hazelnut paste	0.00
12C10472	28102012	Chocolate, milk, with nuts	0.00
12C10486	28103016	Chocolate, milk, with toffee or nougat pieces	0.00
12C10412	28101005	Chocolate, white	0.00
12C10469	28102013	Chocolate, white, with macadamias	0.00
12C10510	28101006	Chocolate, milk & white	0.00
12C10535	28101008	Chocolate, reduced sugar	0.00
12C10522	28103017	Chocolate, favourites	0.00
12C10534	28103018	Chocolate, boxed assorted, not further defined	0.00
12C10502	28101007	Chocolate, plain, not further defined	0.00
12C10545	28103019	Chocolate, not further defined	0.00
12C10445	28102014	Confectionery, almond, chocolate-coated	0.00
12C10504	28103009	Chocolate, chocolate cream centre	0.00
12C10530	28102015	Confectionery, chocolate, hazelnut & wafer centre	0.00
12C10410	28103020	Confectionery, chocolate centre, sugar-coated	0.00
12C10532	28102016	Confectionery, chocolate & peanut centre, sugar-coated	0.00
12C10561	28103021	Confectionery, coffee beans, chocolate-coated	0.00
12C10448	28102017	Confectionery, dried fruit & nuts, chocolate-coated	0.00
12C10547	28401004	Confectionery, fudge, caramel or vanilla	0.00
12C10548	28401005	Confectionery, fudge, chocolate or coffee	0.00
12C10560	28103022	Confectionery, ginger, chocolate-coated	0.00
12C10489	28103023	Confectionery, malt centre, chocolate coated	0.00
12C10550	28102018	Confectionery, mixed nuts, chocolate-coated	0.00
12C10562	28102019	Confectionery, peanut brittle	0.00

12C10444	28102020	Confectionery, peanut, chocolate-coated	0.00
12C10449	28103025	Confectionery, sultana, chocolate-coated	0.00
02E50309	28202004	Confectionery, sesame & toffee	0.00
12C10552	28101009	Confectionery, truffle	0.00
12C10505	28103026	Easter egg, milk, fondant filled	0.00
12C10446	28103027	Licorice, chocolate-coated	0.00
12C10451	28103024	Confectionery, marshmallow-filled, chocolate & coconut coated (snowball)	0.00
12C10475	27103002	Sauce, sweet, caramel, liquid, filling (as used in chocolates, biscuits and ice creams), commercial	0.00
12B10128	25202001	Bean paste	0.00
10B10068	23201002	Chutney or relish, commercial	0.00
10B10076	23201003	Chutney or relish, homemade	0.00
12B10086	27201001	Jam, apricot, regular	0.00
12B10094	27201002	Jam, blackberry, regular	0.00
12B10112	27201003	Jam, fig, regular	0.00
12B10074	27201004	Jam, mixed berry, regular	0.00
12B10076	27201009	Jam, other fruit, regular	0.00
12B10080	27201005	Jam, plum, regular	0.00
12B10092	27201006	Jam, pumpkin & cinnamon, homemade	0.00
12B10084	27201007	Jam, raspberry, regular	0.00
12B10085	27201008	Jam, strawberry, regular	0.00
12B10121	27202001	Jam, all flavours, reduced sugar	0.00
12B10108	27202002	Jam, all flavours, no added sugar (100% fruit)	0.00
12B10082	27202003	Jam, all flavours, intense sweetened	0.00
12B10087	27201010	Jam, not further defined	0.00
12B10083	27203001	Lemon butter, homemade	0.00
12B10098	27201011	Marmalade, ginger, regular	0.00
12B10123	27202004	Marmalade, ginger, reduced sugar	0.00
12B10114	27201012	Marmalade, cumquat (kumquat), regular	0.00
12B10097	27201013	Marmalade, lime or lemon, regular	0.00
12B10104	27202005	Marmalade, lime or lemon, reduced sugar	0.00
12B10075	27201014	Marmalade, orange, regular	0.00
12B10116	27202006	Marmalade, orange, reduced sugar	0.00
12B10100	27202007	Marmalade, orange, no added sugar (100% fruit)	0.00
12B10099	27201015	Marmalade, regular, not further defined	0.00
12B10117	27201016	Paste, quince	0.00
10B10075	23202009	Relish, corn, commercial	0.00
10B10077	23202010	Relish, onion, commercial	0.00
12B10125	27103001	Sauce, sweet, caramel, homemade	0.00
10A10475	27204002	Sauce, sweet, chocolate, homemade	0.00
10A10471	27203002	Sauce, sweet, lemon & butter, homemade	0.00
12B10115	27205003	Sauce, sweet, mixed berry coulis	0.00
12B10079	27204001	Spread, hazelnut & chocolate flavoured	0.00
12B10088	27103003	Topping, caramel	0.00
12B10077	27103004	Topping, chocolate	0.00
12B10126	27103005	Topping, chocolate, hard	0.00

12B10078	27103006	Topping, fruit-flavoured, regular	0.00
12A10079	27102001	Glucose, liquid or syrup	0.00
12A10076	27102002	Honey	0.00
14A10045	31201001	Intense sweetener, containing aspartame, powdered formulation	0.00
14A10046	31201002	Intense sweetener, containing aspartame, tablet formulation	0.00
14A10047	31201003	Intense sweetener, containing aspartame/acesulfame-potassium, tablet	0.00
14A10050	31201004	Intense sweetener, containing saccharin, liquid	0.00
14A10048	31201005	Intense sweetener, containing saccharin, tablet	0.00
14A10044	31201006	Intense sweetener, containing sucralose, powdered formulation	0.00
14A10049	31201007	Intense sweetener, containing sucralose, tablet	0.00
14A10051	31201008	Intense sweetener, containing stevia, powdered formulation	0.00
14A10052	31201009	Intense sweetener, containing stevia, tablet	0.00
14A10053	31201010	Intense sweetener, powder formulation, not further defined	0.00
14A10054	31201011	Intense sweetener, tablet, not further defined	0.00
14B10178	31503011	Pectin, powder, unsweetened	0.00
12A10075	27101001	Sugar, brown	0.00
12A10101	27101002	Sugar, cinnamon	0.00
12A10074	27101003	Sugar, raw	0.00
12A10097	27101004	Sugar, raw, low GI (glucose index)	0.00
12A10102	27101005	Sugar, white, fruit sugar (fructose), granulated or lump	0.00
12A10073	27101006	Sugar, white, granulated or lump	0.00
12A10096	27101007	Sugar, white, with added stevia, granulated	0.00
12A10080	27101008	Sugar, white, icing	0.00
12A10081	27101009	Sugar, white, icing mixture	0.00
14A10055	27102003	Syrup, agave, light & dark, liquid	0.00
12A10077	27102004	Syrup, golden	0.00
12A10078	27102005	Syrup, maple, pure (100% maple)	0.00
12C10470	27304004	Chocolate glaze, commercial	0.00
12A10091	27304005	Icing, chocolate ganache, commercial	0.00
12A10086	27304006	Icing, chocolate ganache, homemade	0.00
12A10085	27304007	Icing, cream cheese, homemade	0.00
12A10089	27304008	Icing, cream style, plain & flavoured (non-chocolate & coffee), commercial	0.00
12A10100	27304009	Icing, egg white based, non-chocolate & coffee, homemade	0.00
12A10093	27304010	Icing, sugar based, chocolate, cocoa powder, commercial	0.00
12A10082	27304011	Icing, sugar based, chocolate, cocoa powder, homemade	0.00
12A10084	27304013	Icing, sugar based, coffee, homemade	0.00
12A10083	27304014	Icing, sugar based, non-chocolate & coffee, homemade	0.00

12A10090	27304012	Icing, sugar based, plain & flavoured (non-chocolate & coffee), commercial	0.00
12A10095	27304015	Icing, homemade, not further defined (non-chocolate & coffee)	0.00
12C10424	28202001	Almond, sugar-coated	0.00
12C10457	28401001	Caramels, soft	0.00
12C10458	28401002	Caramels, hard	0.00
12C10492	28403001	Chewing gum, regular	0.00
12C10493	28404001	Chewing gum, intense sweetened	0.00
12C10450	28202005	Coconut ice, homemade	0.00
12C10540	28405001	Confectionery, chocolate crackle	0.00
12C10527	28405002	Confectionery, honey joy	0.00
02E50263	28102021	Confectionery, rocky road	0.00
02E50293	13306002	Confectionery, rum ball (rumball)	0.00
02E50250	28201001	Confectionery, white Christmas, homemade	0.00
12C10521	28405003	Ginger, crystallised	0.00
12C10454	28201003	Fruit bar, apricot, snack or confectionery style	0.00
06D10558	28201004	Fruit bar, 95% fruit, snack or confectionery style, added fibre & vitamin C & folate	0.00
12C10430	28202006	Halvah, plain	0.00
12C10425	28401006	Honeycomb, plain	0.00
12C10429	28401003	Confectionery, cake decoration, sugar based (hundreds & thousands)	0.00
12D10051	27301001	Jelly crystals, sugar sweetened, all flavours	0.00
12D10053	27301002	Jelly, sugar sweetened, all flavours, prepared	0.00
12D10061	27301003	Jelly, sugar sweetened, all flavours, prepared, added berries	0.00
12D10060	27301004	Jelly, sugar sweetened, all flavours, prepared, added pears	0.00
12D10064	27301005	Jelly, sugar sweetened, all flavours, prepared, added peach, apricot, mango or nectarine	0.00
12D10063	27301006	Jelly, sugar sweetened, all flavours, prepared, added peach & pears	0.00
12D10062	27301007	Jelly, sugar sweetened, all flavours, prepared, added other fruits	0.00
12D10066	27302001	Jelly crystals, intense sweetened, all flavours	0.00
12D10067	27302002	Jelly, intense sweetened, all flavours, prepared	0.00
12D10069	27302003	Jelly, intense sweetened, all flavours, prepared, added peach, apricot, mango or nectarine	0.00
12D10068	27302004	Jelly, intense sweetened, all flavours, prepared, added other fruits	0.00
12C10432	28401007	Licorice, black	0.00
12C10523	28401008	Licorice, flavoured	0.00
12C10452	28401009	Licorice, allsorts	0.00
12C10428	28401010	Lolly, boiled	0.00
12C10453	28401011	Lolly, boiled, chocolate filled	0.00
12C10465	28401012	Lolly, butterscotch	0.00
12C10546	28401013	Lolly, fruit flavoured, chewy	0.00

12C10427	28401014	Lolly, hard varieties	0.00
12C10423	28401015	Lolly, jelly varieties	0.00
12C10511	28401016	Lolly, jelly varieties, natural colours & flavours	0.00
12C10507	28401017	Lolly, jelly varieties, with cocoa	0.00
12C10509	28401018	Lolly, medicated cough lolly or lozenge	0.00
12C10436	28401019	Lolly, mint flavoured, sugar sweetened	0.00
12C10508	28402001	Lolly, mint flavoured, intense sweetened	0.00
12C10557	28402002	Lolly, non-mint flavours, intense sweetened	0.00
12C10563	28401020	Lolly, sour, all varieties	0.00
12C10479	28401021	Lollipop or chupa chup	0.00
12C10422	28401022	Marshmallow, plain or flavoured	0.00
11B10208	28202007	Marzipan, almond paste, added sugar	0.00
12D10052	27301008	Meringue, all flavours, commercial	0.00
12D10059	27301009	Meringue, plain, homemade from basic ingredients	0.00
12C10485	28202008	Nougat, honey & nuts, traditional	0.00
12D10056	27301010	Pavlova, plain, commercial	0.00
12D10055	27301011	Pavlova, plain, homemade from basic ingredients	0.00
12D10057	27301012	Pavlova, plain, topped with whipped cream	0.00
12D10058	27301013	Pavlova, plain, topped with whipped cream & fresh fruit	0.00
12C10421	28401023	Sherbet powder	0.00
12D10054	28401024	Turkish delight, plain	0.00
13A11668	24402001	Artichoke, globe, raw	0.00
13A11806	24402002	Artichoke, globe, boiled, microwaved or steamed, drained	0.00
13A12356	24402003	Artichoke, globe, boiled, microwaved or steamed, drained, added fat not further defined	0.00
13A11669	24302001	Artichoke, jerusalem, peeled, raw	0.00
13A11807	24302002	Artichoke, jerusalem, peeled, boiled, microwaved or steamed, drained	0.00
13A11772	24402004	Artichoke heart, canned in brine, drained	0.00
13A11673	24402005	Asparagus, green, raw	0.00
13A12004	24402006	Asparagus, green, fresh or frozen, baked, roasted, stir-fried or fried, grilled or BBQ'd, no added fat	0.00
13A12005	24402007	Asparagus, green, fresh or frozen, baked, roasted, stir-fried or fried, grilled or BBQ'd, fat not further defined	0.00
13A11916	24402008	Asparagus, green, fresh or frozen, boiled, microwaved or steamed, drained	0.00
13A12208	24402009	Asparagus, green, fresh or frozen, boiled, microwaved or steamed, drained, added fat not further defined	0.00
13A11773	24402010	Asparagus, canned in brine, drained or undrained	0.00
13A11774	24402011	Bamboo shoot, canned in water, heated, drained	0.00
13A12430	24402012	Bamboo shoot, fresh, cooked, with or without fat	0.00
13A11684	24403001	Basil, green, raw	0.00
13A20147	25101001	Bean, black, dried, boiled, microwaved or steamed, drained	0.00
13A11776	24502001	Bean, broad, fresh or frozen, raw	0.00

13A11873	24502002	Bean, broad, fresh or frozen, boiled, microwaved or steamed, drained	0.00
13A12575	25102001	Bean, broad, canned, drained	0.00
13A11777	24502003	Bean, butter, fresh, raw	0.00
13A11874	24502004	Bean, butter, fresh, boiled, microwaved or steamed, drained	0.00
13A12442	24502011	Bean, green, canned, cooked, no added fat	0.00
13A11675	24502005	Bean, green, fresh or frozen, raw	0.00
13A12355	24502006	Bean, green, fresh or frozen, baked, roasted, fried, stir-fried, grilled or BBQ'd, no added fat	0.00
13A12358	24502007	Bean, green, fresh or frozen, baked, roasted, fried, stir-fried, grilled or BBQ'd, fat not further defined	0.00
13A11678	24502008	Bean, green, fresh, boiled, microwaved or steamed, drained	0.00
13A11679	24502009	Bean, green, frozen, boiled, microwaved or steamed, drained	0.00
13A12364	24502010	Bean, green, fresh or frozen, boiled, microwaved or steamed, added fat not further defined	0.00
13A12642	24502014	Bean, green, cooked, not further defined	0.00
13A20148	25101006	Bean, lupin, raw	0.00
13A20150	25102003	Bean, lupin, canned, drained	0.00
13A11778	24502012	Bean, red, fresh, raw	0.00
13A11875	24502013	Bean, red, fresh, boiled, drained	0.00
13A11670	24302003	Beetroot, purple, peeled, fresh or frozen, raw	0.00
13A11990	24302004	Beetroot, purple, peeled or unpeeled, fresh or frozen, baked, roasted, fried, stir-fried, grilled or BBQ'd, no added fat	0.00
13A12366	24302005	Beetroot, purple, peeled or unpeeled, fresh or frozen, baked, roasted, stir-fried, grilled or BBQ'd, fat not further defined	0.00
13A11809	24302006	Beetroot, purple, peeled, fresh or frozen, boiled, microwaved or steamed, drained	0.00
13A12368	24302007	Beetroot, purple, peeled or unpeeled, fresh or frozen, steamed, added fat not further defined	0.00
13A11680	24302008	Beetroot, canned, drained	0.00
13A11683	24201001	Bok choy or choy sum, raw	0.00
13A11812	24201002	Bok choy or choy sum, baked, roasted, fried, stir-fried, grilled or BBQ'd, no added fat	0.00
13A12006	24201003	Bok choy or choy sum, baked, roasted, fried, stir-fried, grilled or BBQ'd, olive oil	0.00
13A12210	24201004	Bok choy or choy sum, baked, roasted, fried, stir-fried, grilled or BBQ'd, other oil	0.00
13A12376	24201005	Bok choy or choy sum, baked, roasted, fried, stir-fried, grilled or BBQ'd, fat not further defined	0.00
13A11912	24201006	Bok choy or choy sum, boiled, microwaved or steamed, drained	0.00
13A12377	24201007	Bok choy or choy sum, boiled, casseroled microwaved, poached, steamed or stewed, added fat not further defined	0.00

13A11681	24202001	Broccoli, fresh or frozen, raw	0.00
13A11934	24202002	Broccoli, fresh or frozen, baked, roasted, fried, stir-fried, grilled or BBQ'd, no added fat	0.00
13A11935	24202003	Broccoli, fresh or frozen, baked, roasted, fried, stir-fried, grilled or BBQ'd, canola oil	0.00
13A11933	24202004	Broccoli, fresh or frozen, baked, roasted, fried, stir-fried, grilled or BBQ'd, olive oil	0.00
13A12370	24202005	Broccoli, fresh or frozen, baked, roasted, fried, stir-fried, grilled or BBQ'd, other oil	0.00
13A12369	24202006	Broccoli, fresh or frozen, baked, roasted, fried, stir-fried, grilled or BBQ'd, fat not further defined	0.00
13A11885	24202007	Broccoli, fresh, boiled, microwaved or steamed, drained	0.00
13A11779	24202008	Broccoli, frozen, boiled, microwaved or steamed, drained	0.00
13A12372	24202009	Broccoli, fresh or frozen, boiled, microwaved or steamed, drained, added fat not further defined	0.00
13A11892	24202010	Broccolini, fresh or frozen, raw	0.00
13A11893	24202011	Broccolini, fresh or frozen, boiled, microwaved or steamed, drained	0.00
13A11682	24201008	Brussels sprout, fresh or frozen, raw	0.00
13A12576	24201009	Brussels sprout, fresh or frozen, baked, roasted, stir-fried, fried, grilled or BBQ'd, fat not further defined	0.00
13A11811	24201010	Brussels sprout, fresh, boiled, microwaved or steamed, drained	0.00
13A11780	24201011	Brussels sprout, frozen, boiled, microwaved or steamed , drained	0.00
13A11782	24201012	Cabbage, Chinese, raw	0.00
13A12327	24201013	Cabbage, Chinese, baked, roasted, fried, stir-fried, grilled or BBQ'd, no added fat	0.00
13A12577	24201014	Cabbage, Chinese, baked, roasted, fried, stir-fried, grilled or BBQ'd, fat not further defined	0.00
13A12027	24201015	Cabbage, Chinese, boiled, microwaved or steamed, drained	0.00
13A12328	24201016	Cabbage, Chinese, boiled, microwaved or steamed, drained, added fat not further defined	0.00
13A11781	24201017	Cabbage, Chinese flowering, raw	0.00
13A11783	24201018	Cabbage, mustard, raw	0.00
13A12619	24201019	Cabbage, mustard, cooked	0.00
13A11784	24201020	Cabbage, red, raw	0.00
13A12211	24201021	Cabbage, red, baked, roasted, fried, stir-fried, grilled or BBQ'd, fat not further defined	0.00
13A11876	24201022	Cabbage, red, boiled, microwaved or steamed, drained, with or without added fat	0.00
13A11785	24201023	Cabbage, red, canned, heated, drained	0.00
13A11786	24201024	Cabbage, savoy, raw	0.00
13A12043	24201025	Cabbage, savoy, baked, roasted, fried, stir-fried, grilled or BBQ'd, with or without added fat	0.00

13A11877	24201026	Cabbage, savoy, boiled, microwaved or steamed, drained, with or without added fat	0.00
13A11685	24201027	Cabbage, white, raw	0.00
13A12330	24201028	Cabbage, white, baked, roasted, fried, stir-fried, grilled or BBQ'd, fat not further defined	0.00
13A11813	24201029	Cabbage, white, boiled, microwaved or steamed, drained, with and without added fat	0.00
13A11908	24201030	Cabbage, raw, not further defined	0.00
13A12323	24201031	Cabbage, baked, roasted, fried, stir-fried, grilled or BBQ'd, no added fat	0.00
13A12373	24201032	Cabbage, baked, roasted, fried, stir-fried, grilled or BBQ'd, butter, dairy blend or margarine	0.00
13A12374	24201033	Cabbage, baked, roasted, fried, stir-fried, grilled or BBQ'd, canola oil	0.00
13A12212	24201034	Cabbage, baked, roasted, fried, stir-fried, grilled or BBQ'd, olive oil	0.00
13A12375	24201035	Cabbage, baked, roasted, fried, stir-fried, grilled or BBQ'd, other oil	0.00
13A12056	24201036	Cabbage, boiled, microwaved or steamed, drained, with or without added fat	0.00
13A12559	24201037	Cabbage, pickled, canned, drained	0.00
10B10073	23201001	Capers, pickled, canned, drained	0.00
13A11686	24705003	Capsicum, green, fresh or frozen, raw	0.00
13A11814	24705004	Capsicum, green, fresh or frozen, baked, roasted, fried, stir-fried, grilled or BBQ'd, no added fat	0.00
13A11959	24705005	Capsicum, green, fresh or frozen, baked, roasted, fried, stir-fried, grilled or BBQ'd, fat not further defined	0.00
13A12333	24705006	Capsicum, green, fresh or frozen, boiled, microwaved or steamed, drained	0.00
13A12380	24705007	Capsicum, green, fresh or frozen, boiled, microwaved or steamed, added fat not further defined	0.00
13A11687	24705008	Capsicum, red, fresh or frozen, raw	0.00
13A11815	24705009	Capsicum, red, fresh or frozen, baked, roasted, fried, stir-fried, grilled or BBQ'd, no added fat	0.00
13A12334	24705010	Capsicum, red, fresh or frozen, baked, roasted, fried, stir-fried, grilled or BBQ'd, fat not further defined	0.00
13A12164	24705011	Capsicum, red, fresh or frozen, boiled, microwaved or steamed, drained	0.00
13A12335	24705012	Capsicum, red, fresh or frozen, boiled, microwaved or steamed, drained, added fat not further defined	0.00
13A11898	24705013	Capsicum, fresh or frozen, raw, not further defined	0.00
13A11968	24705014	Capsicum, fresh or frozen, baked, roasted, fried, stir-fried, grilled or BBQ'd, no added fat	0.00
13A11969	24705015	Capsicum, fresh or frozen, baked, roasted, fried, stir-fried, grilled or BBQ'd, canola oil	0.00
13A11970	24705016	Capsicum, fresh or frozen, baked, roasted, fried, stir-fried, grilled or BBQ'd, olive oil	0.00

13A12332	24705017	Capsicum, fresh or frozen, baked, roasted, fried, stir-fried, grilled or BBQ'd, fat not further defined	0.00
13A12213	24705018	Capsicum, fresh or frozen, boiled, microwaved or steamed, drained	0.00
13A12379	24705019	Capsicum, fresh or frozen, boiled, microwaved or steamed, added fat not further defined	0.00
13A11688	24301001	Carrot, baby, peeled or unpeeled, fresh or frozen, raw	0.00
13A12214	24301002	Carrot, baby, peeled or unpeeled, fresh or frozen, baked, roasted, fried, stir-fried, grilled or BBQ'd, fat not further defined	0.00
13A11817	24301003	Carrot, baby, peeled or unpeeled, fresh or frozen, boiled, microwaved or steamed, drained	0.00
13A11787	24301004	Carrot, baby, canned in brine, boiled or microwaved , drained	0.00
13A11671	24301005	Carrot, mature, peeled or unpeeled, fresh or frozen, raw	0.00
13A11913	24301006	Carrot, mature, peeled or unpeeled, fresh or frozen, baked, roasted, fried, stir-fried, grilled or BBQ'd, no added fat	0.00
13A12382	24301007	Carrot, mature, peeled or unpeeled, fresh or frozen, baked, roasted, fried, stir-fried, grilled or BBQ'd, butter, dairy blend or margarine	0.00
13A12165	24301008	Carrot, mature, peeled or unpeeled, fresh or frozen, baked, roasted, fried, stir-fried, grilled or BBQ'd, canola oil	0.00
13A11936	24301009	Carrot, mature, peeled or unpeeled, fresh or frozen, baked, roasted, fried, stir-fried, grilled or BBQ'd, olive oil	0.00
13A12216	24301010	Carrot, mature, peeled or unpeeled, fresh or frozen, baked, roasted, fried, stir-fried, grilled or BBQ'd, other oil	0.00
13A12215	24301011	Carrot, mature, peeled or unpeeled, fresh or frozen, baked, roasted, fried, stir-fried, grilled or BBQ'd, fat not further defined	0.00
13A11816	24301012	Carrot, mature, peeled or unpeeled, fresh or frozen, boiled, microwaved or steamed, drained	0.00
13A12217	24301013	Carrot, mature, peeled or unpeeled, fresh or frozen, boiled, microwaved or steamed, drained, added fat not further defined	0.00
13A12081	24301014	Carrot, peeled or unpeeled, fresh or frozen, raw, not further defined	0.00
13A11691	24302009	Cassava, peeled, fresh or frozen, raw	0.00
13A11820	24302010	Cassava, peeled, fresh or frozen, boiled, microwaved or steamed, drained	0.00
13A11690	24302011	Cassava, white flesh, peeled, fresh or frozen, raw	0.00
13A11819	24302012	Cassava, white flesh, peeled, fresh or frozen, boiled, microwaved or steamed, drained	0.00
13A11689	24302013	Cassava, yellow flesh, peeled, fresh or frozen, raw	0.00
13A11818	24302014	Cassava, yellow flesh, peeled, fresh or frozen, boiled, microwaved or steamed, drained	0.00

13A11692	24202012	Cauliflower, fresh or frozen, raw	0.00
13A11995	24202013	Cauliflower, fresh or frozen, baked, roasted, fried, stir-fried, grilled or BBQ'd, no added fat	0.00
13A11991	24202014	Cauliflower, fresh or frozen, baked, roasted, fried, stir-fried, grilled or BBQ'd, fat not further defined	0.00
13A11821	24202015	Cauliflower, fresh or frozen, boiled, microwaved or steamed, drained	0.00
13A12222	24202016	Cauliflower, fresh or frozen, boiled, microwaved or steamed, drained, added fat not further defined	0.00
13A11822	24202017	Cauliflower, fresh or frozen, boiled, microwaved or steamed, added homemade cheese sauce	0.00
13A11797	24302015	Celeriac, peeled, raw	0.00
13A11880	24302016	Celeriac, peeled, boiled, microwaved or steamed, drained	0.00
13A11693	24402013	Celery, fresh or frozen, raw	0.00
13A11823	24402014	Celery, fresh or frozen, baked, roasted, fried, stir-fried, grilled or BBQ'd, no added fat	0.00
13A12383	24402015	Celery, fresh or frozen, baked, roasted, fried, stir-fried, grilled or BBQ'd, fat not further defined	0.00
13A11997	24402016	Celery, fresh or frozen, boiled, microwaved or steamed, drained	0.00
13A12336	24402017	Celery, fresh or frozen, boiled, microwaved or steamed, drained, added fat not further defined	0.00
13A11798	24302017	Chicory, raw	0.00
13A11881	24302018	Chicory, boiled, microwaved or steamed, drained	0.00
13A11695	24705020	Chilli (chili), green, raw	0.00
13A11825	24705021	Chilli (chili), green, cooked with or without added fat	0.00
13A12531	24705022	Chilli (chili), green, pickled	0.00
13A11694	24705023	Chilli (chili), red, raw	0.00
13A11824	24705024	Chilli (chili), red, cooked with or without added fat	0.00
13A12337	24705025	Chilli (chili), raw, not further defined	0.00
13A12339	24705026	Chilli (chili), baked, roasted, fried, stir-fried, grilled or BBQ'd, with or without fat	0.00
13A12338	24705027	Chilli (chili), boiled, microwaved or steamed, drained, with or without fat	0.00
13A11788	24403002	Chives, raw	0.00
13A11799	24705028	Choko, peeled, fresh or frozen, raw	0.00
13A11882	24705029	Choko, peeled, fresh or frozen, boiled, microwaved or steamed, drained	0.00
13A11698	24403003	Coriander, fresh, leaves & stems	0.00
13A11701	24705030	Cucumber, apple crystal, unpeeled, raw	0.00
13A11696	24705031	Cucumber, common, peeled, raw	0.00
13A11697	24705032	Cucumber, common, unpeeled, raw	0.00
13A11699	24705033	Cucumber, Lebanese, unpeeled, raw	0.00
13A11700	24705034	Cucumber, telegraph, unpeeled, raw	0.00
13A11904	24705035	Cucumber, peeled or unpeeled, raw, not further defined	0.00
13A12433	24705036	Cucumber, peeled or unpeeled, cooked, no added fat	0.00

13A11897	24403004	Dill, raw	0.00
13A11702	24705037	Eggplant, peeled or unpeeled, fresh or frozen, raw	0.00
13A11827	24705038	Eggplant, peeled or unpeeled, fresh or frozen, baked, roasted, fried, stir-fried, grilled or BBQ'd, no added fat	0.00
13A12045	24705039	Eggplant, peeled or unpeeled, fresh or frozen, baked, roasted, fried, stir-fried, grilled or BBQ'd, olive oil	0.00
13A12184	24705040	Eggplant, peeled or unpeeled, fresh or frozen, baked, roasted, fried, stir-fried, grilled or BBQ'd, other oil	0.00
13A11826	24705041	Eggplant, peeled or unpeeled, fresh or frozen, boiled, microwaved or steamed, drained	0.00
13A12615	24705042	Eggplant, peeled or unpeeled, fresh or frozen, boiled, microwaved or steamed, drained, added fat not further defined	0.00
13A11789	24401001	Endive, raw	0.00
13A11800	24801001	Fennel, fresh or frozen, raw	0.00
13A11883	24801002	Fennel, fresh or frozen, boiled, microwaved or steamed, drained	0.00
13A11667	24802001	Garlic, peeled or unpeeled, fresh or frozen, raw	0.00
13A11829	24802002	Garlic, peeled or unpeeled, fresh or frozen, baked, roasted, fried, stir-fried, grilled, BBQ'd, deep-fried, with or without added fat	0.00
13A12028	24802003	Garlic, peeled or unpeeled, fresh or frozen, boiled, microwaved or steamed, drained, with or without added fat	0.00
10B10072	23202001	Gherkin, pickled, drained, commercial	0.00
13A11704	24302019	Ginger, peeled, fresh or frozen, raw	0.00
13A11830	24302020	Ginger, peeled, fresh or frozen, baked, roasted, fried, stir-fried, grilled or BBQ'd, with or without added fat	0.00
13A12030	24302021	Ginger, peeled, fresh or frozen, boiled, microwaved or steamed, with or without added fat	0.00
13A12569	23202002	Ginger, pickled, drained	0.00
13A12623	24201038	Kale, raw	0.00
13A12624	24201039	Kale, cooked	0.00
13A11801	24201040	Kohlrabi, peeled, fresh or frozen, raw	0.00
13A11884	24201041	Kohlrabi, peeled, fresh or frozen, boiled, microwaved or steamed, drained	0.00
13A11706	24802004	Leek, raw	0.00
13A11992	24802005	Leek, baked, roasted, fried, stir-fried, grilled or BBQ'd, no added fat	0.00
13A12434	24802006	Leek, baked, roasted, fried, stir-fried, grilled or BBQ'd, fat not further defined	0.00
13A12341	24802007	Leek, boiled, microwaved or steamed, drained	0.00
13A12435	24802008	Leek, boiled, casseroled, microwaved, poached, steamed or stewed, added fat not further defined	0.00
13A11709	24401002	Lettuce, cos, raw	0.00
13A11708	24401003	Lettuce, iceberg, raw	0.00
13A11710	24401004	Lettuce, mignonette, raw	0.00
13A11909	24401005	Lettuce, raw, not further defined	0.00

13A12025	24401006	Lettuce, baked, roasted, fried, stir-fried, grilled or BBQ'd, with or without added fat	0.00
13A12396	24401007	Lettuce, boiled, casseroled, microwaved, poached, steamed or stewed, with or without added fat	0.00
13A11802	24705043	Melon, bitter, fresh or frozen, raw	0.00
13A12446	24705044	Melon, bitter, fresh or frozen, baked, roasted, fried, stir-fried, grilled or BBQ'd, no added fat	0.00
13A12447	24705045	Melon, bitter, fresh or frozen, baked, roasted, fried, stir-fried, grilled or BBQ'd, fat not further defined	0.00
13A11803	24705046	Melon, hairy, fresh or frozen, raw	0.00
13A11895	24403007	Mint, raw	0.00
13A12084	24403006	Herbs, mixed, raw	0.00
13A12283	24803018	Mixed green vegetables, for omelette recipes	0.00
13A12095	24401008	Mixed leafy greens, for salad recipes, lettuce, spinach & rocket, raw	0.00
13A12590	24904033	Mixed salad vegetables, common mix, for use in garden salads	0.00
13A12552	24803019	Mixed vegetables, cooked, for use in casserole recipes	0.00
13A12553	24803020	Mixed vegetables, cooked, for use in coconut-based curry recipes	0.00
13A12555	24803021	Mixed vegetables, cooked, for use in all other curry recipes	0.00
13A12640	24803035	Mixed vegetables, cooked, for use in casserole and curry recipes	0.00
13A12556	24803023	Mixed vegetables, cooked, for use in stir fry recipes	0.00
13A12641	24803036	Mixed vegetables, cooked, for use in stir fry recipes	0.00
13A12562	24803022	Mixed vegetables, for use in cream & cheese based pasta dishes	0.00
13A12564	24803024	Mixed vegetables, for use in tomato based pasta dishes	0.00
13A12558	24803025	Mixed vegetables, for use in quiche recipes	0.00
13A12560	24803026	Mixed vegetables, for use in zucchini slice recipes	0.00
13A12074	24803002	Mixed vegetables, Asian greens, raw	0.00
13A12177	24803003	Mixed vegetables, Asian greens, stir-fried or fried, no added fat	0.00
13A12180	24803004	Mixed vegetables, Asian greens, stir-fried or fried, fat not further defined	0.00
13A12176	24803005	Mixed vegetables, Asian greens, boiled, microwaved or steamed, drained	0.00
13A12284	24803006	Mixed vegetables, Asian greens, boiled, microwaved or steamed, drained, added fat not further defined	0.00
13A12090	24803027	Mixed vegetables, for homemade mixed dishes, asparagus & celery	0.00
13A12088	24803028	Mixed vegetables, for homemade mixed dishes, bok choy, spinach, cabbage & silverbeet, raw	0.00
13A12076	24803029	Mixed vegetables, for homemade mixed dishes, broccoli, cauliflower & broccolini, raw	0.00
13A12051	24803030	Mixed vegetables, for homemade mixed dishes, butter beans, green peas, green beans & snow peas, raw	0.00

13A12091	24803031	Mixed vegetables, for homemade mixed dishes, carrot, parsnip, radish, swede & turnip, raw	0.00
13A12079	24803032	Mixed vegetables, for homemade mixed dishes, onion, shallot, leek, spring onion & garlic, raw	0.00
13A12075	24803033	Mixed vegetables, for homemade mixed dishes, pumpkin & sweet potato, raw	0.00
13A12089	24803034	Mixed vegetables, for homemade mixed dishes, zucchini, squash & choko, raw	0.00
13A12621	24803007	Mixed vegetables, fresh or frozen, raw	0.00
13A12592	24803008	Mixed vegetables, fresh or frozen, with carrot, pumpkin or sweet potato, cooked, no added fat	0.00
13A12595	24803009	Mixed vegetables, fresh or frozen, with carrot, pumpkin or sweet potato, cooked, fat not further defined	0.00
13A12594	24803010	Mixed vegetables, fresh or frozen, without carrot, pumpkin or sweet potato, cooked, no added fat	0.00
13A12596	24803011	Mixed vegetables, fresh or frozen, without carrot, pumpkin or sweet potato, cooked, fat not further defined	0.00
13A12296	24803012	Mixed vegetables, fresh or frozen, broccoli & carrot, cooked	0.00
13A12114	24803013	Mixed vegetables, fresh or frozen, broccoli, carrot & pea/bean, cooked, with or without fat	0.00
13A12125	24803014	Mixed vegetables, fresh or frozen, carrot & pea/bean, cooked, with or without fat	0.00
13A12578	24803015	Mixed vegetables, carrot, corn & pea/bean, canned, cooked, no added fat	0.00
13A12289	24803016	Mixed vegetables, fresh, carrot, corn & pea/bean, cooked, with or without fat	0.00
13A11713	24803017	Mixed vegetables, purchased frozen, carrot, corn & pea/bean, cooked, with or without fat	0.00
13A12643	24803037	Mixed vegetables, cooked, not further defined	0.00
13A12613	23202003	Mixed vegetables, pickled, drained	0.00
13B10319	24903003	Mixed vegetable, tempura coating, fried or deep fried, fat not further defined	0.00
13A11715	24703001	Mushroom, common, fresh or frozen, raw	0.00
13A11836	24703002	Mushroom, common, fresh or frozen, baked, roasted, fried, stir-fried, grilled or BBQ'd, no added fat	0.00
13A12342	24703003	Mushroom, common, fresh or frozen, baked, roasted, fried, stir-fried, grilled or BBQ'd, butter, dairy blend or margarine	0.00
13A11951	24703004	Mushroom, common, fresh or frozen, baked, roasted, fried, stir-fried, grilled or BBQ'd, canola oil	0.00
13A11925	24703005	Mushroom, common, fresh or frozen, baked, roasted, fried, stir-fried, grilled or BBQ'd, olive oil	0.00
13A12343	24703006	Mushroom, common, fresh or frozen, baked, roasted, fried, stir-fried, grilled or BBQ'd, other oil	0.00

13A12062	24703007	Mushroom, common, fresh or frozen, baked, roasted, fried, stir-fried, grilled or BBQ'd, fat not further defined	0.00
13A12060	24703008	Mushroom, common, fresh or frozen, boiled, microwaved or steamed, drained	0.00
13A12344	24703009	Mushroom, common, fresh or frozen, boiled, microwaved or steamed, drained, added fat not further defined	0.00
13A12046	24703010	Mushroom, oriental, fresh or frozen, raw, not further defined	0.00
13A12047	24703011	Mushroom, oriental, fresh or frozen, baked, roasted, fried, stir-fried, grilled or BBQ'd, fat not further defined	0.00
13A12519	24703012	Mushroom, oriental, fresh or frozen, boiled, microwaved or steamed, drained	0.00
13A12048	24703013	Mushroom, oriental, fresh or frozen, boiled, microwaved or steamed, drained, added fat not further defined	0.00
13A12058	24703014	Mushroom, fresh or frozen, raw, not further defined	0.00
13A11790	24703015	Mushroom, canned in brine, drained	0.00
13A11791	24703016	Mushroom, straw, Asian, canned in brine, drained	0.00
13A12563	24705047	Okra, raw	0.00
13A12579	24705048	Okra, cooked, with or without added fat	0.00
10B10071	23202004	Olive, green or black, drained	0.00
10B10070	23202005	Olive, green, pimento stuffed, drained	0.00
13A11672	24802009	Onion, mature, brown skinned, peeled, raw	0.00
13A11903	24802010	Onion, mature, red skinned, peeled, fresh or frozen, raw	0.00
13A11717	24802011	Onion, mature, white skinned, peeled, fresh or frozen, raw	0.00
13A11899	24802012	Onion, mature, peeled, fresh or frozen, raw, not further defined	0.00
13A12167	24802013	Onion, mature, peeled, fresh or frozen, baked, roasted, fried, stir-fried, grilled or BBQ'd, no added fat	0.00
13A12565	24802014	Onion, mature, peeled, fresh or frozen, baked, roasted, fried, stir-fried, grilled or BBQ'd, butter, dairy blend or margarine	0.00
13A11966	24802015	Onion, mature, peeled, fresh or frozen, baked, roasted, fried, stir-fried, grilled, BBQ'd, canola oil	0.00
13A11967	24802016	Onion, mature, peeled, fresh or frozen, baked, roasted, fried, stir-fried, grilled, BBQ'd, olive oil	0.00
13A12166	24802017	Onion, mature, peeled, fresh or frozen, baked, roasted, fried, stir-fried, grilled or BBQ'd, other oil	0.00
13A12566	24802018	Onion, mature, peeled, fresh or frozen, baked, roasted, fried, stir-fried, grilled or BBQ'd, fat not further defined	0.00
13A12032	24802019	Onion, mature, peeled, fresh or frozen, boiled, microwaved or steamed, drained	0.00

13A12033	24802020	Onion, mature, peeled, fresh or frozen, boiled, microwaved or steamed, drained, added fat not further defined	0.00
13A12101	24802027	Onion, for salad recipes, onion (red, white & brown), spring onion, shallots & leeks, raw	0.00
10B10067	23202006	Onion, pickled, drained, commercial	0.00
13B10291	24903004	Onion ring, breadcrumb coating, fried	0.00
13A11718	24802021	Onion, spring, raw	0.00
13A11839	24802022	Onion, spring, baked, roasted, fried, stir-fried, grilled or BBQ'd, no added fat	0.00
13A12233	24802023	Onion, spring, baked, roasted, fried, stir-fried, grilled or BBQ'd, fat not further defined	0.00
13A12022	24802024	Onion, spring, boiled, microwaved or steamed, drained, with or without added fat	0.00
13A11722	24403008	Parsley, continental, raw	0.00
13A11721	24403009	Parsley, curly, raw	0.00
13A11901	24403010	Parsley, not further defined, raw	0.00
13A11724	24302022	Parsnip, peeled, fresh or frozen, raw	0.00
13A11842	24302023	Parsnip, peeled or unpeeled, fresh or frozen, baked, roasted, fried, stir-fried, grilled or BBQ'd, no added fat	0.00
13A12234	24302024	Parsnip, peeled or unpeeled, fresh or frozen, baked, roasted, fried, stir-fried, grilled or BBQ'd, fat not further defined	0.00
13A11841	24302025	Parsnip, peeled or unpeeled, fresh or frozen, boiled, microwaved or steamed, drained, with or without added fat	0.00
13A11726	24501001	Pea, green, fresh or frozen, raw	0.00
13A11727	24501002	Pea, green, fresh, cooked, no added fat	0.00
13A11728	24501003	Pea, green, frozen, cooked, no added fat	0.00
13A11972	24501004	Pea, green, cooked, no added fat	0.00
13A12580	24501005	Pea, green, cooked, added fat not further defined	0.00
13A11792	24501006	Pea, green, canned in brine, cooked	0.00
10B10069	23202007	Pickles, mustard, sweet, commercial	0.00
10B10074	23202008	Pickles, for use on sandwiches, not further defined	0.00
13A11769	24102002	Potato, chips, regular, fast food outlet, deep fried, blended oil, salted	0.00
13A11890	24102003	Potato, chips, regular, fast food outlet, deep fried, monounsaturated oil, salted	0.00
13A12018	24102004	Potato, chips, regular, independent takeaway outlet, cafe or restaurant, deep fried, blended oil, no added salt	0.00
13A12016	24102005	Potato, chips, regular, independent takeaway outlet, cafe or restaurant, deep fried, blended oil, salted	0.00
13A11771	24102006	Potato, chips, regular, purchased frozen, baked or roasted, no added fat	0.00
13A12390	24102007	Potato, chips, regular, purchased frozen, baked or roasted, fat not further defined	0.00
13A12389	24102008	Potato, chips, regular, purchased frozen, deep fried or fried, fat not further defined	0.00

13A12407	24102009	Potato, chips, reduced fat, purchased frozen, baked or roasted, no added fat	0.00
13A12039	24102010	Potato, chips, homemade from fresh potato, deep fried or fried, canola oil	0.00
13A12388	24102011	Potato, chips, homemade from fresh potato, deep fried or fried, olive oil	0.00
13A12036	24102012	Potato, chips, homemade from fresh potato, deep fried or fried, other oil	0.00
13A12413	24102013	Potato, chips, homemade from fresh potato, deep fried or fried, fat not further defined	0.00
13A11760	24101001	Potato, coliban, peeled, raw	0.00
13A11864	24101002	Potato, coliban, peeled, baked, roasted, fried, stir-fried, grilled or BBQ'd, no added fat	0.00
13A11888	24101003	Potato, coliban, peeled, boiled, microwaved or steamed, drained	0.00
13A11761	24101004	Potato, desiree, peeled, raw	0.00
13A11867	24101005	Potato, desiree, peeled, baked, roasted, fried, stir-fried, grilled or BBQ'd, no added fat	0.00
13A12235	24101006	Potato, desiree, peeled, baked, roasted, fried, stir-fried, grilled or BBQ'd, fat not further defined	0.00
13A11866	24101007	Potato, desiree, peeled, boiled, microwaved or steamed, drained	0.00
13A12064	24101008	Potato, desiree, unpeeled, raw	0.00
13A12082	24101009	Potato, desiree, unpeeled, baked, roasted, fried, stir-fried, grilled or BBQ'd, no added fat	0.00
13A11889	24102014	Potato, fries, fast food outlet, deep fried, blended oil, salted	0.00
13A12020	24102015	Potato, fries, fast food outlet, deep fried, monounsaturated oil, no added salt	0.00
13A11770	24102016	Potato, fries, fast food outlet, deep fried, monounsaturated oil, salted	0.00
13A12019	24102017	Potato, fries, independent takeaway outlet, cafe or restaurant, deep fried, blended oil, no added salt	0.00
13A12017	24102018	Potato, fries, independent takeaway outlet, cafe or restaurant, deep fried, blended oil, salted	0.00
13A12392	24102019	Potato, fries, regular, purchased frozen, par-fried in canola oil, raw	0.00
13A12393	24102020	Potato, fries, regular, purchased frozen, baked or roasted, no added fat	0.00
13A12394	24102021	Potato, fries, regular, purchased frozen, deep fried or fried, fat not further defined	0.00
13A12040	24102022	Potato, fries, homemade from fresh potato, peeled, deep-fried, fat not further defined	0.00
13A12454	24102023	Potato, gem, nugget or royal, independent takeaway outlet, cafe or restaurant, deep fried, fat not further defined	0.00
13A12185	24102024	Potato, gem, nugget or royal, regular, purchased frozen, par-fried in canola oil, raw	0.00
13A12186	24102025	Potato, gem, nugget or royal, regular, purchased frozen, baked or roasted, with or without added fat	0.00

13A12416	24102026	Potato, gem, nugget or royal, regular, purchased frozen, deep fried or fried, fat not further defined	0.00
13B10245	24102027	Potato, hash brown, McDonalds	0.00
13A12203	24102028	Potato, hash brown, independent takeaway outlet, cafe or restaurant, deep fried, oil not further defined	0.00
13A12201	24102029	Potato, hash brown, purchased frozen, par-fried in canola oil, raw	0.00
13A12202	24102030	Potato, hash brown, purchased frozen, baked, roasted, grilled or BBQ'd, no added fat	0.00
13A12415	24102031	Potato, hash brown, purchased frozen, baked or roasted, fat not further defined	0.00
13A11764	24103001	Potato, mashed, dried powder	0.00
13A11765	24103002	Potato, mashed, prepared from dried powder with cows milk or water	0.00
13A12349	24103003	Potato, mashed, with gravy, as purchased from a fast food outlet	0.00
13A11752	24101010	Potato, new, peeled or unpeeled, raw	0.00
13A11859	24101011	Potato, new, peeled or unpeeled, baked, roasted, fried, stir-fried, grilled or BBQ'd, no added fat	0.00
13A11753	24101012	Potato, new, peeled or unpeeled, boiled, microwaved or steamed, drained	0.00
13A11762	24101020	Potato, pale skin, peeled, raw	0.00
13A11871	24101021	Potato, pale skin, peeled, baked, roasted, fried, stir-fried, grilled or BBQ'd, no added fat	0.00
13A12522	24101022	Potato, pale skin, peeled, baked, roasted, fried, stir-fried, grilled or BBQ'd, butter, dairy blend or margarine	0.00
13A11930	24101023	Potato, pale skin, peeled, baked, roasted, fried, stir-fried, grilled or BBQ'd, canola oil	0.00
13A11929	24101024	Potato, pale skin, peeled, baked, roasted, fried, stir-fried, grilled or BBQ'd, olive oil	0.00
13A12532	24101025	Potato, pale skin, peeled, baked, roasted, fried, stir-fried, grilled or BBQ'd, other oil	0.00
13A12523	24101026	Potato, pale skin, peeled, baked, roasted, fried, stir-fried, grilled or BBQ'd, fat not further defined	0.00
13A11887	24101027	Potato, pale skin, peeled, boiled, microwaved or steamed, drained	0.00
13A12236	24101028	Potato, pale skin, peeled, boiled, microwaved or steamed, drained, added butter, dairy blend or margarine	0.00
13A12238	24101029	Potato, pale skin, peeled, boiled, microwaved or steamed, drained, added oil	0.00
13A12237	24101030	Potato, pale skin, peeled, boiled, microwaved or steamed, drained, added fat not further defined	0.00
13A11979	24101031	Potato, pale skin, unpeeled, raw	0.00
13A11988	24101032	Potato, pale skin, unpeeled, baked, roasted, fried, stir-fried, grilled or BBQ'd, no added fat	0.00
13A12533	24101033	Potato, pale skin, unpeeled, baked, roasted, fried, stir-fried, grilled or BBQ'd, butter, dairy blend or margarine	0.00

13A12024	24101034	Potato, pale skin, unpeeled, baked, roasted, fried, stir-fried, grilled or BBQ'd, canola oil	0.00
13A12065	24101035	Potato, pale skin, unpeeled, baked, roasted, fried, stir-fried, grilled or BBQ'd, olive oil	0.00
13A12239	24101036	Potato, pale skin, unpeeled, baked, roasted, fried, stir-fried, grilled or BBQ'd, other oil	0.00
13A12063	24101037	Potato, pale skin, unpeeled, baked, roasted, fried, stir-fried, grilled or BBQ'd, fat not further defined	0.00
13A12023	24101038	Potato, pale skin, unpeeled, boiled, microwaved or steamed, drained	0.00
13A12534	24101039	Potato, pale skin, unpeeled, boiled, microwaved or steamed, added fat not further defined	0.00
13A11872	24103005	Potato, pale skin, peeled or unpeeled, mashed with cows milk & butter or dairy blend	0.00
13A12188	24103006	Potato, pale skin, peeled or unpeeled, mashed with cows milk & margarine spread	0.00
13A11759	24101013	Potato, pontiac, peeled, raw	0.00
13A11862	24101014	Potato, pontiac, peeled, baked, roasted, fried, stir-fried, grilled or BBQ'd, no added fat	0.00
13A11861	24101015	Potato, pontiac, peeled, boiled, microwaved or steamed, drained	0.00
13A11763	24101040	Potato, red skin, peeled, raw	0.00
13A11869	24101041	Potato, red skin, peeled, baked, roasted, fried, stir-fried, grilled or BBQ'd, no added fat	0.00
13A11941	24101042	Potato, red skin, peeled, baked, roasted, fried, stir-fried, grilled or BBQ'd, fat not further defined	0.00
13A11886	24101043	Potato, red skin, peeled, boiled, microwaved or steamed, drained	0.00
13A11980	24101044	Potato, red skin, unpeeled, raw	0.00
13A11989	24101045	Potato, red skin, unpeeled, baked, roasted, fried, stir-fried, grilled or BBQ'd, no added fat	0.00
13A11985	24101046	Potato, red skin, unpeeled, baked, roasted, fried, stir-fried, grilled or BBQ'd, fat not further defined	0.00
13A12352	24101047	Potato, red skin, unpeeled, boiled, microwaved or steamed, drained	0.00
13A12536	24101048	Potato, red skin, unpeeled, boiled, microwaved or steamed, added fat not further defined	0.00
13A11870	24103007	Potato, red skin, peeled or unpeeled, mashed with cows milk & butter or dairy blend	0.00
13A11905	24101049	Potato, peeled, raw, not further defined	0.00
13A11907	24101050	Potato, peeled, baked, roasted, fried, stir-fried, grilled or BBQ'd, no added fat	0.00
13A12541	24101051	Potato, peeled, baked, roasted, fried, stir-fried, grilled or BBQ'd, butter, dairy blend or margarine	0.00
13A11927	24101052	Potato, peeled, baked, roasted, fried, stir-fried, grilled or BBQ'd, canola oil	0.00
13A11928	24101053	Potato, peeled, baked, roasted, fried, stir-fried, grilled or BBQ'd, olive oil	0.00
13A12204	24101054	Potato, peeled, baked, roasted, fried, stir-fried, grilled or BBQ'd, other oil	0.00

13A12241	24101055	Potato, peeled, baked, roasted, fried, stir-fried, grilled or BBQ'd, fat not further defined	0.00
13A11906	24101056	Potato, peeled, boiled, microwaved or steamed, drained	0.00
13A12242	24101057	Potato, peeled, boiled, microwaved or steamed, drained, added butter, dairy spread or margarine	0.00
13A12535	24101058	Potato, peeled, boiled, microwaved or steamed, added fat not further defined	0.00
13B10247	24102032	Potato scallop, deep fried, saturated frying fat, salted	0.00
13A11754	24101016	Potato, sebago, peeled, raw	0.00
13A11756	24101017	Potato, sebago, peeled, baked, roasted, fried, stir-fried, grilled or BBQ'd, animal fat	0.00
13A11757	24103004	Potato, sebago, peeled, boiled & mashed without added ingredients	0.00
13A11758	24101018	Potato, sebago, unpeeled, baked, roasted, fried, stir-fried, grilled or BBQ'd, no added fat	0.00
13A11755	24101019	Potato, sebago, unpeeled, boiled, microwaved or steamed, drained	0.00
13A11981	24101059	Potato, unpeeled, raw, not further defined	0.00
13A11987	24101060	Potato, unpeeled, baked, roasted, fried, stir-fried, grilled or BBQ'd, no added fat	0.00
13A12543	24101061	Potato, unpeeled, baked, roasted, fried, stir-fried, grilled or BBQ'd, butter, dairy blend or margarine	0.00
13A12066	24101062	Potato, unpeeled, baked, roasted, fried, stir-fried, grilled or BBQ'd, olive oil	0.00
13A12073	24101063	Potato, unpeeled, baked, roasted, fried, stir-fried, grilled or BBQ'd, other oil	0.00
13A12542	24101064	Potato, unpeeled, baked, roasted, fried, stir-fried, grilled or BBQ'd, fat not further defined	0.00
13A12067	24101065	Potato, unpeeled, boiled, microwaved or steamed, drained, with or without added fat	0.00
13A11926	24103008	Potato, peeled or unpeeled, mashed with cows milk & butter or dairy blend	0.00
13A12187	24103009	Potato, peeled or unpeeled, mashed with cows milk & margarine spread	0.00
13A12614	24103010	Potato, peeled or unpeeled, mashed with cows milk & oil	0.00
13A12011	24103011	Potato, peeled, mashed, prepared, from cafe or restaurant	0.00
15A10939	24001002	Potato, wild harvested, cooked	0.00
13A12385	24102033	Potato, wedges, independent takeaway outlet, cafe or restaurant, deep fried, blended oil, salted	0.00
13A12386	24102034	Potato, wedges, regular, purchased frozen, par-fried in canola oil, raw	0.00
13A12387	24102035	Potato, wedges, regular, purchased frozen, baked or roasted, no added fat	0.00
13A12404	24102036	Potato, wedges, regular, purchased frozen, deep fried or fried, fat not further defined	0.00
13A12172	24103012	Potato bake, made with bacon, cheese &/or cream	0.00
13A12173	24103013	Potato bake, made with cheese	0.00

13A12169	24103014	Potato bake, made with cream	0.00
13A12170	24103015	Potato bake, made with cheese & cream	0.00
13A12618	24103016	Potato bake, made with mixed vegetables & cream &/or cheese	0.00
13A12525	24103017	Potato, for stuffed potato recipes	0.00
13A12529	24103018	Potato, filled with bacon, cheese &/or sour cream	0.00
13A12526	24103019	Potato, filled with cheese &/or sour cream	0.00
13A12617	24103020	Potato, filled with legumes &/or vegetables	0.00
13A12616	24103021	Potato, filled with meat & cheese &/or sour cream	0.00
13A11734	24701001	Pumpkin, butternut, peeled, fresh or frozen, raw	0.00
13A11847	24701002	Pumpkin, butternut, peeled, fresh or frozen, baked, roasted, fried, stir-fried, grilled or BBQ'd, no added fat	0.00
13A12193	24701003	Pumpkin, butternut, peeled, fresh or frozen, baked, roasted, fried, stir-fried, grilled or BBQ'd, fat not further defined	0.00
13A11846	24701004	Pumpkin, butternut, peeled, fresh or frozen, boiled, microwaved or steamed, drained, with or without added fat	0.00
13A11732	24701005	Pumpkin, golden nugget, peeled, fresh or frozen, raw	0.00
13A11843	24701006	Pumpkin, golden nugget, peeled, fresh or frozen, baked or roasted, no added fat	0.00
13A11737	24701007	Pumpkin, jarrahdale, peeled, fresh or frozen, raw	0.00
13A11851	24701008	Pumpkin, jarrahdale, peeled, fresh or frozen, baked or roasted, no added fat	0.00
13A11850	24701009	Pumpkin, jarrahdale, peeled, fresh or frozen, boiled, microwaved or steamed, drained	0.00
13A11738	24701013	Pumpkin, peeled, fresh or frozen, raw	0.00
13A11853	24701014	Pumpkin, peeled, fresh or frozen, baked, roasted, fried, stir-fried, grilled or BBQ'd, no added fat	0.00
13A11958	24701015	Pumpkin, peeled, fresh or frozen, baked, roasted, fried, stir-fried, grilled or BBQ'd, canola oil	0.00
13A11957	24701016	Pumpkin, peeled, fresh or frozen, baked, roasted, fried, stir-fried, grilled or BBQ'd, olive oil	0.00
13A12189	24701017	Pumpkin, peeled, fresh or frozen, baked, roasted, fried, stir-fried, grilled or BBQ'd, other oil	0.00
13A12068	24701018	Pumpkin, peeled, fresh or frozen, baked, roasted, fried, stir-fried, grilled or BBQ'd, fat not further defined	0.00
13A11852	24701019	Pumpkin, peeled, fresh or frozen, boiled, microwaved or steamed, drained	0.00
13A12247	24701020	Pumpkin, peeled, fresh or frozen, boiled, microwaved or steamed, drained, added butter, dairy blend or margarine	0.00
13A12537	24701021	Pumpkin, peeled, fresh or frozen, boiled, microwaved or steamed, added fat not further defined	0.00
13A12194	24701022	Pumpkin, unpeeled, fresh or frozen, raw	0.00
13A12195	24701023	Pumpkin, unpeeled, fresh or frozen, baked, roasted, fried, stir-fried, grilled or BBQ'd, no added fat	0.00

13A12244	24701024	Pumpkin, unpeeled, fresh or frozen, baked, roasted, fried, stir-fried, grilled or BBQ'd, canola oil	0.00
13A12196	24701025	Pumpkin, unpeeled, fresh or frozen, baked, roasted, fried, stir-fried, grilled or BBQ'd, olive oil	0.00
13A12246	24701026	Pumpkin, unpeeled, fresh or frozen, baked, roasted, fried, stir-fried, grilled or BBQ'd, other oil	0.00
13A12245	24701027	Pumpkin, unpeeled, fresh or frozen, baked, roasted, fried, stir-fried, grilled or BBQ'd, fat not further defined	0.00
13A12250	24701028	Pumpkin, unpeeled, fresh or frozen, boiled, microwaved or steamed, drained, with or without added fat	0.00
13A11733	24701010	Pumpkin, queensland blue, peeled, fresh or frozen, raw	0.00
13A11845	24701011	Pumpkin, queensland blue, peeled, fresh or frozen, baked or roasted, no added fat	0.00
13A11844	24701012	Pumpkin, queensland blue, peeled, fresh or frozen, boiled, microwaved or steamed, drained	0.00
13A12083	24401009	Rocket, raw	0.00
13A12574	24401010	Rocket, cooked, with or without added fat	0.00
13A11804	24302026	Radish, red skinned, unpeeled, raw	0.00
13A11998	24302027	Radish, red skinned, unpeeled, baked, roasted, fried, stir-fried, grilled or BBQ'd, no added fat	0.00
13A11805	24302028	Radish, white skinned, peeled or unpeeled, raw	0.00
13A11999	24302029	Radish, white skinned, peeled or unpeeled, baked, roasted, fried, stir-fried, grilled or BBQ'd, no added fat	0.00
13A12163	24302030	Radish, peeled or unpeeled, raw	0.00
13A11896	24403011	Rosemary, raw	0.00
13A12436	24904035	Salad, for use in hamburger recipes	0.00
13A12510	24904036	Salad, for use in Mexican recipes, beef wraps	0.00
13A12511	24904037	Salad, for use in Mexican recipes, chicken wraps	0.00
13B10334	24905001	Antipasto, pickled vegetables, cured meat & cheese	0.00
13A12151	24905002	Salad, Asian style, made with cabbage, carrot, onion, fresh herbs, peanuts & poached chicken, no added dressing	0.00
13A12152	24905003	Salad, Asian style, made with cabbage, carrot, onion, fresh herbs, peanuts & poached chicken, with dressing	0.00
13A12589	24904001	Salad, broccoli, no added dressing	0.00
13A12155	24905004	Salad, caesar, made with lettuce, bacon, parmesan & croutons, no added dressing	0.00
13A12156	24905005	Salad, caesar, made with lettuce, bacon, parmesan, croutons & egg, no added dressing	0.00
13A12160	24904002	Salad, caprese, made with tomato, basil & mozzarella cheese, no added dressing	0.00
13A12584	24904003	Salad, carrot, no added dressing	0.00
13A12157	24905006	Salad, chicken caesar, made with lettuce, bacon, parmesan, croutons & chicken, no added dressing	0.00

13A12158	24905007	Salad, chicken caesar, made with lettuce, bacon, parmesan, croutons, egg & chicken, no added dressing	0.00
13B10336	25202014	Salad, chickpea, with vegetables	0.00
13B10266	24904004	Salad, coleslaw, commercial, added dressing	0.00
13B10267	24904005	Salad, coleslaw, homemade from basic ingredients, added dressing	0.00
13B10333	24904006	Salad, coleslaw, with cheese, homemade from basic ingredients, added dressing	0.00
02F40473	13515006	Salad, couscous with cheese & vegetables	0.00
13A12583	24904007	Salad, cucumber, no added dressing	0.00
13A12605	24905008	Salad, garden, added bacon, no added dressing	0.00
13A12606	24905009	Salad, garden, added bacon & cheese, no added dressing	0.00
13A12612	24904011	Salad, garden, added beef, lamb or pork, no added dressing	0.00
13A12601	24904012	Salad, garden, added cheese, no added dressing	0.00
13A12625	24905010	Salad, garden, added cheese & beef, lamb or pork, no added dressing	0.00
13A12602	24905011	Salad, garden, added cheese & egg, no added dressing	0.00
13A12603	24904013	Salad, garden, added cheese & fruit, no added dressing	0.00
13A12604	24904014	Salad, garden, added cheese & nuts &/or seeds, no added dressing	0.00
13A12610	24905012	Salad, garden, added chicken, no added dressing	0.00
13A12611	24905013	Salad, garden, added chicken & cheese, no added dressing	0.00
13A12626	24905014	Salad, garden, added chicken & egg, no added dressing	0.00
13A12598	24905015	Salad, garden, added egg, no added dressing	0.00
13A12599	24904015	Salad, garden, added fruit, no added dressing	0.00
13A12629	24904016	Salad, garden, added grains, no added dressing	0.00
13A12600	24904017	Salad, garden, added nuts &/or seeds, no added dressing	0.00
13A12609	24905016	Salad, garden, added seafood, no added dressing	0.00
13A12607	24905017	Salad, garden, added salmon or tuna, no added dressing	0.00
13A12608	24905018	Salad, garden, added salmon or tuna & cheese, no added dressing	0.00
13A12159	24905019	Salad, garden, added salmon or tuna & egg, no added dressing	0.00
13A12627	24905020	Salad, garden, added tofu, no added dressing	0.00
13A12120	24904018	Salad, garden, made from leafy greens, capsicum, cucumber & tomato, no added dressing	0.00
13A12099	24904019	Salad, garden, made from leafy greens, carrot, cucumber & tomato, no added dressing	0.00
13A12127	24904020	Salad, garden, made from leafy greens, cucumber, onion & tomato, no added dressing	0.00
13A12097	24904021	Salad, garden, made from leafy greens, cucumber & tomato, no added dressing	0.00

13A12098	24904022	Salad, garden, made from leafy greens & tomato, no added dressing	0.00
13A12587	24904023	Salad, greek, no added dressing	0.00
13A12586	24904024	Salad, green bean, no added dressing	0.00
13A12161	24904025	Salad, made with rocket & parmesan, no added dressing	0.00
02A30021	13509079	Salad, noodle, with chicken, vegetables & nuts	0.00
02A30020	13509080	Salad, noodle, with vegetables	0.00
02F40421	13509081	Salad, pasta with vegetables, added dressing	0.00
02F40471	13509082	Salad, pasta with vegetables & cheese, added dressing	0.00
13B10268	24904026	Salad, potato, commercial, added dressing	0.00
13B10269	24904027	Salad, potato, homemade from basic ingredients, added dressing	0.00
13B10335	24905021	Salad, potato, with bacon/ham, homemade from basic ingredients, regular fat dressing	0.00
02A10502	13511030	Salad, rice, with vegetables, added dressing	0.00
13A12585	24904028	Salad, roast vegetable, no added dressing	0.00
13A12628	24904029	Salad, roast vegetable & cheese, no added dressing	0.00
05D10428	15603006	Salad, seafood, commercial, creamy dressing	0.00
13B10263	24904030	Salad, tabouleh, commercial	0.00
13B10272	24904031	Salad, tabouleh, homemade from basic ingredients	0.00
13A12153	24905022	Salad, Thai beef, made with lettuce, onion, cucumber, herbs, chilli & beef, no added dressing	0.00
13A12154	24905023	Salad, Thai beef, made with lettuce, onion, cucumber, herbs, chilli & beef, with dressing	0.00
13B10264	25202015	Salad, three bean, commercial	0.00
13B10265	25202016	Salad, three bean, homemade from basic ingredients	0.00
13A11891	24404001	Seaweed, nori, dried	0.00
13A12581	24404002	Seaweed, boiled, microwaved or steamed, drained	0.00
13A11720	24802025	Shallot, peeled, raw	0.00
13A11840	24802026	Shallot, peeled, baked, roasted, fried, stir-fried, grilled or BBQ'd, no added fat	0.00
13A11750	24401011	Silverbeet, fresh or frozen, raw	0.00
13A11856	24401012	Silverbeet, fresh or frozen, boiled, microwaved or steamed, drained	0.00
13A11751	24501007	Snow pea, fresh or frozen, raw	0.00
13A11857	24501008	Snow pea, fresh or frozen, baked, roasted, fried, stir-fried, grilled or BBQ'd, no added fat	0.00
13A12545	24501009	Snow pea, fresh or frozen, baked, roasted, fried, stir-fried, grilled or BBQ'd, fat not further defined	0.00
13A11858	24501010	Snow pea, fresh or frozen, boiled, microwaved or steamed, drained	0.00
13A12538	24501011	Snow pea, fresh or frozen, boiled, microwaved or steamed, added fat not further defined	0.00
13A11703	24401013	Spinach, fresh, raw	0.00
13A12197	24401014	Spinach, fresh, baked, roasted, fried, stir-fried, grilled or BBQ'd , no added fat	0.00

13A12198	24401015	Spinach, fresh, baked, roasted, fried, stir-fried, grilled or BBQ'd, fat not further defined	0.00
13A11832	24401016	Spinach, fresh, boiled, microwaved or steamed, drained	0.00
13A11794	24401018	Spinach, frozen, boiled, microwaved or steamed, drained	0.00
13A12539	24401017	Spinach, fresh, boiled, microwaved or steamed, added fat not further defined	0.00
13A12200	24401019	Spinach, boiled, microwaved or steamed, drained, not further defined	0.00
13A11705	24401020	Spinach, water, raw	0.00
13A12622	24401021	Spinach, water, cooked, with or without added fat	0.00
13A11666	24503001	Sprout, alfalfa, raw	0.00
13A11775	24503002	Sprout, bean, raw	0.00
13A12431	24503003	Sprout, bean, cooked, no added fat	0.00
13A12432	24503004	Sprout, bean, cooked, fat not further defined	0.00
13A12207	24503005	Sprout, for use in salad recipes, alfalfa & mung bean	0.00
13A11707	24702001	Squash, button, fresh or frozen, raw	0.00
13A11831	24702002	Squash, button, fresh or frozen, boiled, microwaved or steamed, drained	0.00
13A11793	24702003	Squash, scallopini, fresh or frozen, raw	0.00
13A11878	24702004	Squash, scallopini, fresh or frozen, boiled, microwaved or steamed, drained	0.00
13A11711	24302031	Swede, peeled, fresh or frozen, raw	0.00
13A12620	24302032	Swede, peeled, fresh or frozen, baked, roasted, fried, stir-fried, grilled or BBQ'd, fat not further defined	0.00
13A11833	24302033	Swede, peeled, fresh or frozen, boiled, microwaved or steamed, drained	0.00
13A11712	24302034	Sweet potato, orange flesh, peeled or unpeeled, fresh or frozen, raw	0.00
13A11835	24302035	Sweet potato, orange flesh, peeled or unpeeled, fresh or frozen, baked, roasted, fried, stir-fried, grilled or BBQ'd, no added fat	0.00
13A11932	24302036	Sweet potato, orange flesh, peeled or unpeeled, fresh or frozen, baked, roasted, fried, stir-fried, grilled or BBQ'd, canola oil	0.00
13A11931	24302037	Sweet potato, orange flesh, peeled or unpeeled, fresh or frozen, baked, roasted, fried, stir-fried, grilled or BBQ'd, olive oil	0.00
13A11965	24302038	Sweet potato, orange flesh, peeled or unpeeled, fresh or frozen, baked, roasted, fried, stir-fried, grilled or BBQ'd, other oil	0.00
13A12530	24302039	Sweet potato, orange flesh, peeled or unpeeled, fresh or frozen, baked, fried, grilled, fat not further defined	0.00
13A11834	24302040	Sweet potato, orange flesh, peeled or unpeeled, fresh or frozen, boiled, microwaved or steamed, drained	0.00
13A12540	24302041	Sweet potato, orange flesh, peeled or unpeeled, fresh or frozen, boiled, microwaved or steamed, added fat not further defined	0.00

13A12572	24302042	Sweet potato, orange flesh, mashed with fat not further defined	0.00
13A12015	24302043	Sweet potato, white flesh, peeled or unpeeled, fresh or frozen, raw	0.00
13A12550	24302044	Sweet potato, white flesh, peeled or unpeeled, fresh or frozen, baked, roasted, fried, stir-fried, grilled or BBQ'd, no added fat	0.00
13A12549	24302045	Sweet potato, white flesh, peeled or unpeeled, fresh or frozen, stir-fried, fat not further defined	0.00
13A12021	24302046	Sweet potato, white flesh, peeled or unpeeled, fresh or frozen, boiled, microwaved or steamed, drained	0.00
13A12570	24302047	Sweet potato, chips, regular, purchased frozen, par-fried in canola oil, raw	0.00
13A12571	24302048	Sweet potato, chips, regular, purchased frozen, baked or roasted, with or without added fat	0.00
13A11735	24704001	Sweetcorn, baby, canned in brine, heated, drained	0.00
13A11731	24704002	Sweetcorn, creamed, canned, heated	0.00
13A11714	24704003	Sweetcorn, fresh or frozen on cob, raw	0.00
13A12181	24704004	Sweetcorn, fresh or frozen on cob, baked, roasted, fried, stir-fried, grilled or BBQ'd, no added fat	0.00
13A12182	24704005	Sweetcorn, fresh or frozen on cob, baked, roasted, fried, stir-fried, grilled or BBQ'd, added fat not further defined	0.00
13A11922	24704006	Sweetcorn, fresh or frozen on cob, boiled, microwaved or steamed, drained	0.00
13A12183	24704007	Sweetcorn, fresh or frozen on cob, boiled, microwaved or steamed, drained, added fat not further defined	0.00
13A11716	24704008	Sweetcorn, fresh or frozen on cob, boiled or microwaved in brine, drained	0.00
13A11729	24704009	Sweetcorn, kernels, canned in brine, drained	0.00
13A11730	24704010	Sweetcorn, kernels, canned in brine, heated, drained	0.00
13A11719	24704011	Sweetcorn, kernels, fresh or frozen, raw	0.00
13A12551	24704012	Sweetcorn, kernels, baked, roasted, fried, stir-fried, grilled or BBQ'd, fat not further defined	0.00
13A11725	24704013	Sweetcorn, kernels, fresh or frozen, boiled, microwaved or steamed, drained	0.00
13A12049	24704014	Sweetcorn, kernels, fresh or frozen, boiled, microwaved or steamed, drained, added fat not further defined	0.00
13A11723	24704015	Sweetcorn, kernels, fresh or frozen, boiled or microwaved in brine, drained	0.00
13A12174	24704016	Sweetcorn, fresh or frozen, raw, not further defined	0.00
13A12175	24704017	Sweetcorn, fresh or frozen, boiled, microwaved or steamed, drained	0.00
13A11740	24302049	Taro, peeled, fresh or frozen, raw	0.00
13A11854	24302050	Taro, peeled, fresh or frozen, boiled, microwaved or steamed, drained	0.00
13A11741	24601001	Tomato, cherry or grape, raw	0.00
13A11739	24601002	Tomato, common, raw	0.00

13A11767	24601003	Tomato, common, boiled with salt, drained	0.00
13A11742	24601004	Tomato, hydroponic, raw	0.00
13A12044	24601005	Tomato, roma, raw	0.00
13A11900	24601006	Tomato, raw, not further defined	0.00
13A12162	24601007	Tomato, fresh, baked, roasted, fried, stir-fried, grilled or BBQ'd, no added fat	0.00
13A12253	24601008	Tomato, fresh, baked, roasted, fried, stir-fried, grilled or BBQ'd, butter, dairy blend or margarine	0.00
13A12256	24601009	Tomato, fresh, baked, roasted, fried, stir-fried, grilled or BBQ'd, canola oil	0.00
13A12070	24601010	Tomato, fresh, baked, roasted, fried, stir-fried, grilled or BBQ'd, olive oil	0.00
13A12258	24601011	Tomato, fresh, baked, roasted, fried, stir-fried, grilled or BBQ'd, other oil	0.00
13A12257	24601012	Tomato, fresh, baked, roasted, fried, stir-fried, grilled or BBQ'd, fat not further defined	0.00
13B10337	24902011	Tomato, stuffed with breadcrumbs, cheese & vegetables, cooked	0.00
13A12346	24601013	Tomato, fresh, boiled, microwaved or steamed, drained	0.00
13A12345	24601014	Tomato, fresh, boiled, microwaved or steamed, drained, added fat not further defined	0.00
13A11743	24602001	Tomato, paste, with added salt	0.00
13A11977	24602002	Tomato, paste, no added salt	0.00
13A12050	24602003	Tomato, paste, not further defined	0.00
13A11975	24602004	Tomato, puree, commercial	0.00
13A11745	24602005	Tomato, sundried or semi-sundried	0.00
13A11748	24602006	Tomato, whole, canned in tomato juice, undrained	0.00
13A11746	24602007	Tomato, whole, canned in tomato juice, drained	0.00
13A11749	24602008	Tomato, whole, canned in tomato juice, boiled or microwaved, undrained	0.00
13A11747	24602009	Tomato, whole, canned in tomato juice, boiled or microwaved, drained	0.00
13A11744	24302051	Turnip, white, peeled, fresh or frozen, raw	0.00
13A12347	24302052	Turnip, white, peeled or unpeeled, fresh or frozen, baked, roasted, fried, stir-fried, grilled or BBQ'd, with or without added fat	0.00
13A11855	24302053	Turnip, white, peeled or unpeeled, fresh or frozen, boiled, microwaved or steamed, drained, with or without added fat	0.00
13A11894	24401022	Vine leaf, grape, canned	0.00
13A12041	24302054	Wasabi, root, raw	0.00
13A12554	24801003	Water chestnut, peeled, canned, drained	0.00
13A11795	24401023	Watercress, raw	0.00
13A12007	24401024	Watercress, boiled, microwaved or steamed, drained	0.00
13A12010	24401025	Watercress, boiled, microwaved or steamed, drained, added fat not further defined	0.00
15A10943	24001003	Yam, wild harvested, cooked	0.00

13A11796	24702005	Zucchini, golden, fresh or frozen, peeled or unpeeled, raw	0.00
13A11879	24702006	Zucchini, golden, fresh or frozen, peeled or unpeeled, boiled, microwaved or steamed, drained	0.00
13A11736	24702007	Zucchini, green skin, fresh or frozen, peeled or unpeeled, raw	0.00
13A11849	24702008	Zucchini, green skin, fresh or frozen, peeled or unpeeled, baked, roasted, fried, stir-fried, grilled or BBQ'd, no added fat	0.00
13A12573	24702009	Zucchini, green skin, fresh or frozen, peeled or unpeeled, stir-fried, butter, dairy blend or margarine	0.00
13A11950	24702010	Zucchini, green skin, fresh or frozen, peeled or unpeeled, baked, roasted, fried, stir-fried, grilled or BBQ'd, canola oil	0.00
13A11949	24702011	Zucchini, green skin, fresh or frozen, peeled or unpeeled, baked, roasted, fried, stir-fried, grilled or BBQ'd, olive oil	0.00
13A12055	24702012	Zucchini, green skin, fresh or frozen, peeled or unpeeled, baked, roasted, fried, stir-fried, grilled or BBQ'd, fat not further defined	0.00
13A11848	24702013	Zucchini, green skin, fresh or frozen, peeled or unpeeled, boiled, microwaved or steamed, drained	0.00
13A12582	24702014	Zucchini, green skin, fresh or frozen, peeled or unpeeled, boiled, microwaved or steamed, drained, added fat not further defined	0.00
